# Supplementary material for: Functional profiling and visualization of the sphingolipid metabolic network in vivo
Source: EMBO Rep. 2025 Nov 10;26(24):6380–417. doi: 10.1038/s44319-025-00632-0 (PMC12714868; doi:10.1038/s44319-025-00632-0)
Supplement: Supplementary file 2 — Appendix [file 44319_2025_632_MOESM2_ESM.pdf]

Appendix for

Functional profiling and visualization of the sphingolipid metabolic network *in vivo*

Page	Item	Description
2	Appendix Fig. S1	Cell-type expression patterns of pre-Cre HG, post-Cre HG, and CRIMIC-GAL4s.
4	Appendix Table S1	The summary of cell-type expression patterns of pre-Cre HG, post-Cre HG, and CRIMIC-GAL4s.
5	Appendix Fig. S2	mCD8GFP expression driven by Pre-Cre and Post-Cre HG lines of CDase, aSMase, wun2, Cpes.
7	Appendix Fig. S3	mCD8GFP expression driven by Pre-Cre and Post-Cre HG lines of ifc, nSMase, ORMDL, Glct.
9	Appendix Fig. S4	Anti-HA immunoblots of the adult fly lysate from HG lines.
11	Appendix Fig. S5	nls-mCherry expression in the central brain driven by HG lines of aSMase, CG6299, and dSMPD4.
13	Appendix Fig. S6	nls-mCherry expression in the central brain driven by HG lines of CG8536, CG14517, and CG17223.
15	Appendix Fig. S7	nls-mCherry expression in the central brain driven by HG lines of kdsr, CG11425, and CG11426.
17	Appendix Fig. S8	nls-mCherry expression in the central brain driven by HG lines of CG11437, CG11438, and laza.
19	Appendix Fig. S9	nls-mCherry expression in the central brain driven by HG lines of CG15533, CG15534, and gba1a.
21	Appendix Fig. S10	nls-mCherry expression in the central brain driven by HG lines of CG30392, CG33090, and Cpes.
23	Appendix Fig. S11	nls-mCherry expression in the central brain driven by HG lines of Dgat2, ect-3, and fdl.
25	Appendix Fig. S12	nls-mCherry expression in the central brain driven by HG lines of egh, brn, and fa2h.
27	Appendix Fig. S13	nls-mCherry expression in the central brain driven by HG lines of eloF, elovl7, and Gal.
29	Appendix Fig. S14	nls-mCherry expression in the central brain driven by HG lines of gba1b, ghi, and ifc.
31	Appendix Fig. S15	nls-mCherry expression in the central brain driven by HG lines of hexo2, lace, and mdy.
33	Appendix Fig. S16	nls-mCherry expression in the central brain driven by HG lines of nSMase, ORMDL, and Sap-r.
35	Appendix Fig. S17	nls-mCherry expression in the central brain driven by HG lines of schlank, sk1, and sk2.
37	Appendix Fig. S18	nls-mCherry expression in the central brain driven by HG lines of smsr, spin, and sply.
39	Appendix Fig. S19	nls-mCherry expression in the central brain driven by HG lines of spt-I, wun, and wun2.
41	Appendix Fig. S20	nls-mCherry expression in the central brain driven by HG lines of acsl, bwa, and cerk.
43	Appendix Fig. S21	nls-mCherry expression in the central brain driven by HG lines of cert and CG1318.
45	Appendix Fig. S22	mCD8GFP expression driven by HG lines of lace, spt-I, ORMDL, kdsr, schlank, and ifc
47	Appendix Fig. S23	mCD8GFP expression driven by HG lines of Glct, egh, brn, CG14517, CG8536, CG17223
49	Appendix Fig. S24	mCD8GFP expression driven by HG lines of CG6962, nSMase, aSMase, CG15533, CG15534
51	Appendix Fig. S25	mCD8GFP expression driven by HG lines of gba1b, gba1a, CG33090, fdl, Gal, and Ect-3
53	Appendix Fig. S26	mCD8GFP expression driven by HG lines of hexo2, hexo1, CG30392, cerk, ghi, and CG6299
55	Appendix Fig. S27	mCD8GFP expression driven by HG lines of fa2h, Dgat2, acsl, mdy, elovl7, and eloF
57	Appendix Fig. S28	mCD8GFP expression driven by HG lines of CDase, bwa, sk1, sk2, spin, and sply
59	Appendix Fig. S29	mCD8GFP expression driven by HG lines of wun, wun2, CG11425, CG11426, CG11437, and CG11438
61	Appendix Fig. S30	mCD8GFP expression driven by HG lines of Smsr, cert, Cpes, laza, and Sap-r
63	Appendix Fig. S31	Brain warping representative figure.
65	Appendix Fig. S32	Anti-HA immunostainings of adult brains from HG lines of aSMase, CG6299, and Glct
67	Appendix Fig. S33	Anti-HA immunostainings of adult brains from HG lines of CG8536, CG14517, and CG17223
69	Appendix Fig. S34	Anti-HA immunostainings of adult brains from HG lines of kdsr, CG11425, and CG11426
71	Appendix Fig. S35	Anti-HA immunostainings of adult brains from HG lines of CG11437, CG11438, and laza
73	Appendix Fig. S36	Anti-HA immunostainings of adult brains from HG lines of CG30392, CG33090, and Cpes
75	Appendix Fig. S37	Anti-HA immunostainings of adult brains from HG lines of Dgat2, ect-3, and fdl
77	Appendix Fig. S38	Anti-HA immunostainings of adult brains from HG lines of egh, brn, and fa2h
79	Appendix Fig. S39	Anti-HA immunostainings of adult brains from HG lines of eloF, elovl7, and Gal
81	Appendix Fig. S40	Anti-HA immunostainings of adult brains from HG lines of gba1b, ghi, and ifc
83	Appendix Fig. S41	Anti-HA immunostainings of adult brains from HG lines of hexo2, lace, and mdy
85	Appendix Fig. S42	Anti-HA immunostainings of adult brains from HG lines of nSMase, ORMDL, and Sap-r
87	Appendix Fig. S43	Anti-HA immunostainings of adult brains from HG lines of Schlank, sk1, and sk2
89	Appendix Fig. S44	Anti-HA immunostainings of adult brains from HG lines of SMSr, spin, and sply
91	Appendix Fig. S45	Anti-HA immunostainings of adult brains from HG lines of spt-I, wun, and wun2
93	Appendix Fig. S46	Anti-HA immunostainings of adult brains from HG lines of acsl, bwa, and cerk
95	Appendix Fig. S47	Anti-HA immunostainings of adult brains from HG lines of cert and CG1318
97	Appendix Fig. S48	Prediction of signaling peptides on CDase, Gba1a, CG15533, and CG15534.
99	Appendix Fig. S49	Subcellular localization of OlyAw in L3 salivary glands.
101	Appendix Fig. S50	Subcellular localization of OlyAw in adult brain.
103	Appendix Fig. S51	OlyAw live imaging reveals lipid raft dynamic.
105	Appendix Fig. S52	CRISPR-Knockout of dSMPD4 and nSMase.
106	Appendix Fig. S53	RT-qPCR analysis of nSMase RNAi knockdown efficiency in neurons of the adult brain.
107	Appendix Fig. S54	Lipidomic analysis of dSMPD4KO and nSMaseKO brains.
109	Appendix Fig. S55	Subcellular localization of dSMPD4-3XHA, nSMase-3XHA, and aSMase-3XHA in the adult brain.
111	Appendix Fig. S56	dSMPD4 loss-of-function phenotypes.

Appendix Fig. S1

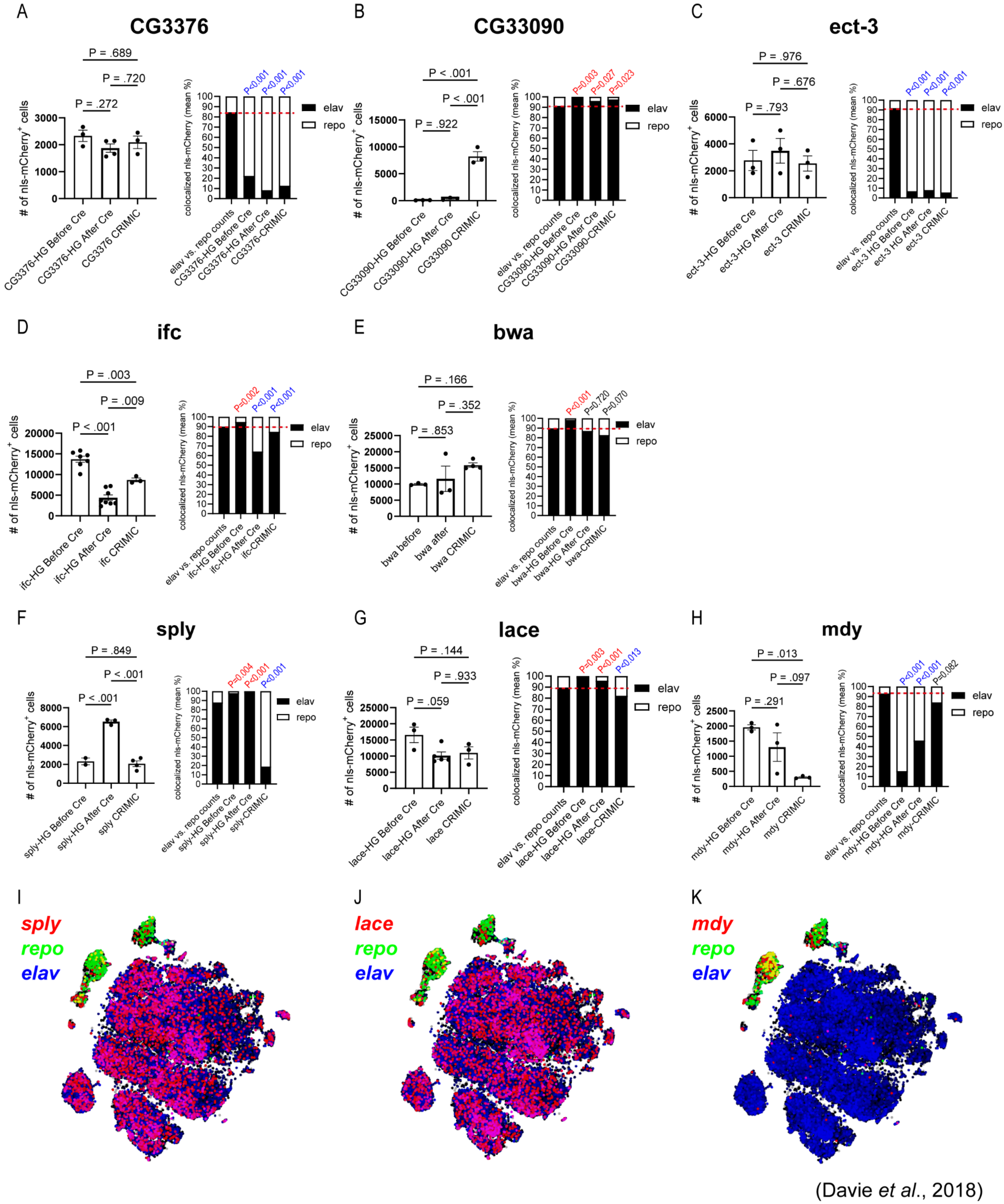

Appendix Figure S1 Cell-type expression patterns of pre-Cre HG, post-Cre HG, and CRIMIC-GAL4s

A-H (Left) Quantification of nls-mCherry positive cells driven by pre-Cre HG, post-Cre HG, and CRIMIC-GAL4s. (Right) Quantification of nls-mCherry colocalized to neuronal and glia markers. Bar graphs showed the percentage of nls-mCherry spots colocalizing to neuronal nuclei (anti-Elav; black bars) and glial nuclei (anti-Repo; white bars) in the total number of labeled nls-mCherry (colocalizing with Elav or Repo). **A**: *CG3376/aSMase*, **B**: *CG33090,* **C**: *ect3*, **D**: *ifc*, **E**: *bwa*, **F**: *sply*, **G**: *lace*, **H**: *mdy*.

I-K Visualization of cellular expression using SCENIC t-SNEs of the 57K dataset from Davie et al. 2018. targeted gene expression (red); *repo* expression represents glia (green); *elav* expression represents neurons (blue). **I**: *sply*, **J**: *lace*, **K**: *mdy.*

Appendix Table S1

	HG-Before Cre	HG-After Cre	CRIMIC	HG Before vs After Cre	HG-After Cre vs CRIMIC
sk1	Glial enrichment	Glial enrichment	Glial enrichment	consistent	consistent
gba1b	Glial enrichment	Glial enrichment	Glial enrichment	consistent	consistent
CDase	Glial enrichment	Glial enrichment	Glial enrichment	consistent	consistent
CG3376	Glial enrichment	Glial enrichment	Glial enrichment	consistent	consistent
CG33090	Neuronal enrichment	Neuronal enrichment	Neuronal enrichment	consistent	consistent
ect-3	Glial enrichment	Glial enrichment	Glial enrichment	consistent	consistent
ifc	Neuronal enrichment	Glial enrichment	Glial enrichment	inconsistent	consistent
lace	Neuronal enrichment	Neuronal enrichment	Glial enrichment	consistent	inconsistent
mdy	Glial enrichment	Glial enrichment	No enrichment	consistent	inconsistent
sply	Neuronal enrichment	Neuronal enrichment	Glial enrichment	consistent	inconsistent
bwa	Neuronal enrichment	No enrichment	No enrichment	inconsistent	consistent
				Consistency 9/11	Consistency 8/11

Appendix Table S1 The summary of cell-type expression patterns of pre-Cre HG, post-Cre HG, and CRIMIC-GAL4s
This table described the cell-type enrichment of GAL4 expression from HG lines (before and after cre excision) and CRIMIC lines.

Appendix Fig. S2

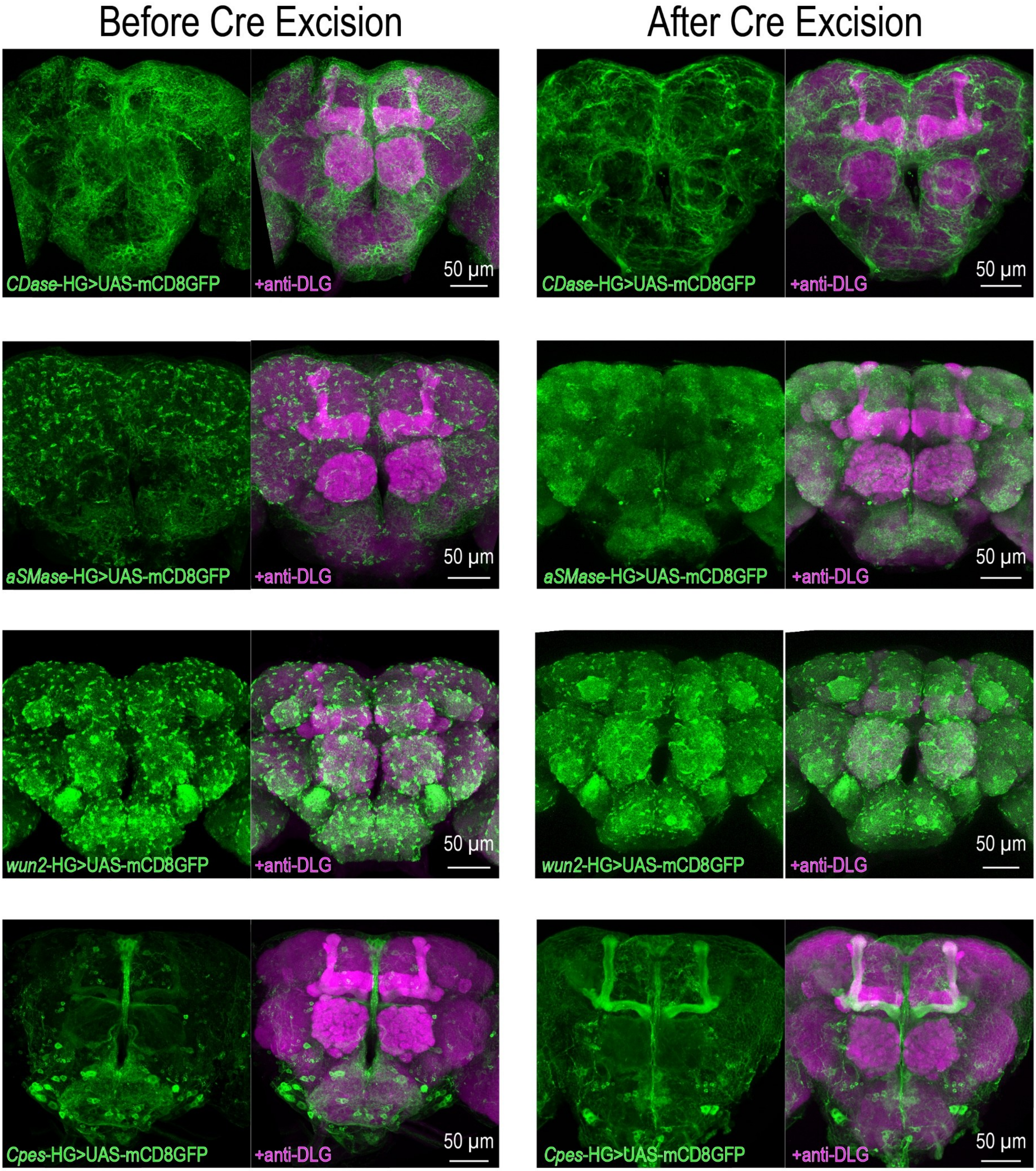

Appendix Figure S2 mCD8GFP expression driven by Pre-Cre and Post-Cre HG lines of *CDase, aSMase, wun2, Cpes*

The expression pattern of targeted genes in the central brain of young adult flies (1-week-old) is visualized with UAS-mCD8GFP (green) and neuropil staining (magenta; anti-DLG)

Appendix Fig. S3

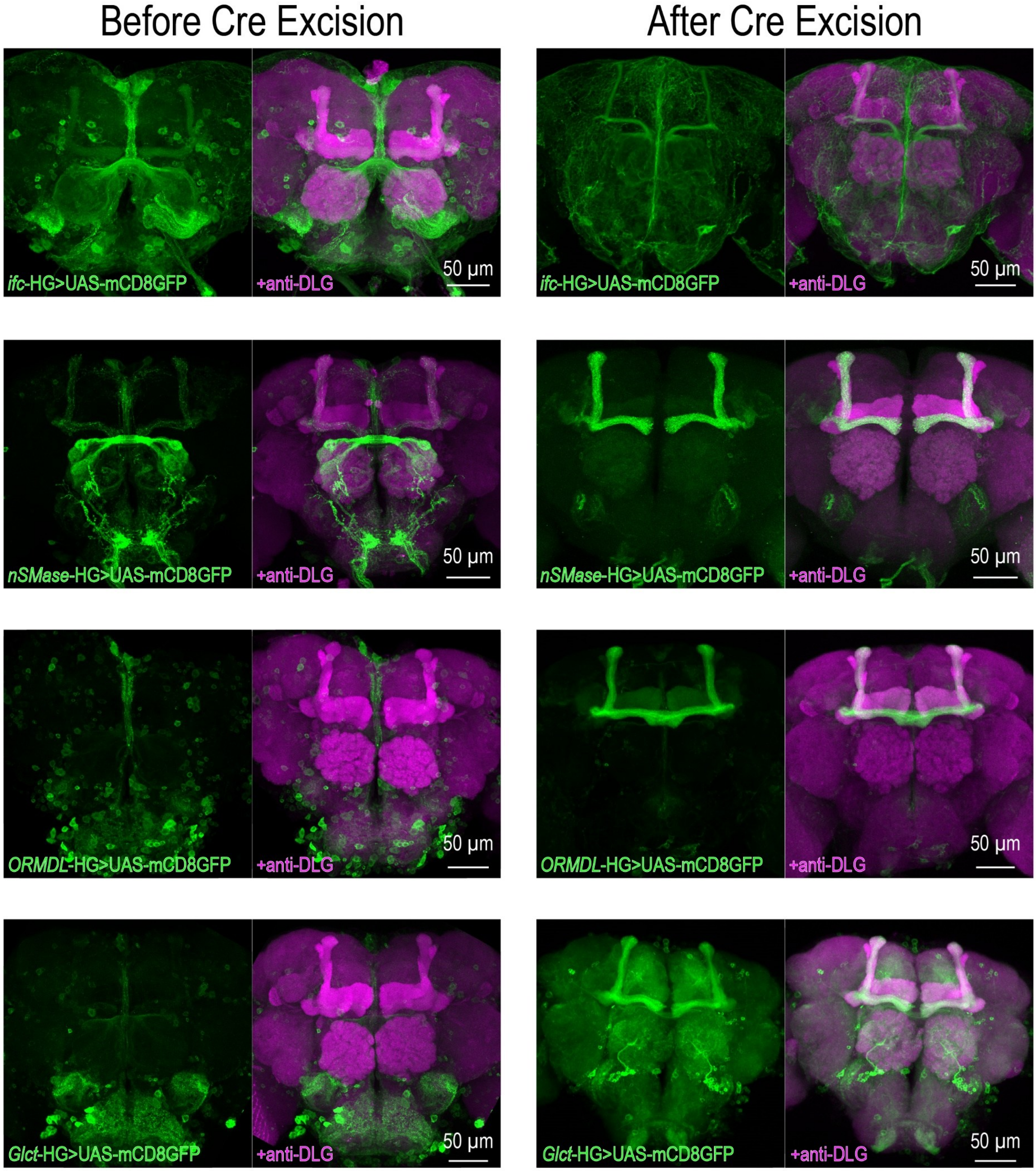

Appendix Figure S3 mCD8GFP expression driven by Pre-Cre and Post-Cre HG lines of *ifc, nSMase, ORMDL, Glct*

The expression pattern of targeted genes in the central brain of young adult flies (1-week-old) is visualized with UAS-mCD8GFP (green) and neuropil staining (magenta; anti-DLG)

Appendix Fig. S4

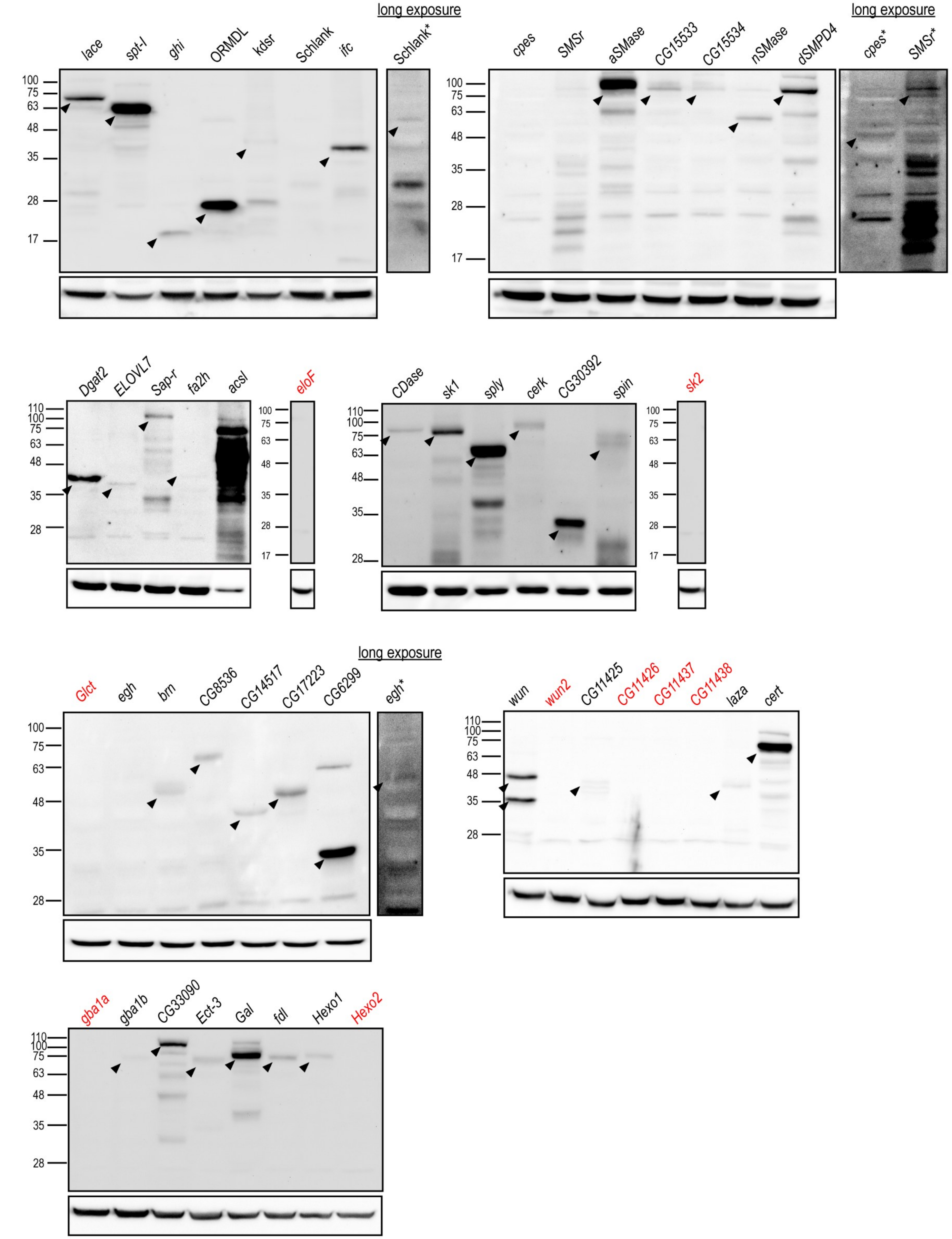

Appendix Figure S4 Anti-HA immunoblots of the adult fly lysate from HG lines

Anti HA immunoblots of whole animal lysates from heterozygous young adult HG flies (1 week of age).

Appendix Fig. S5

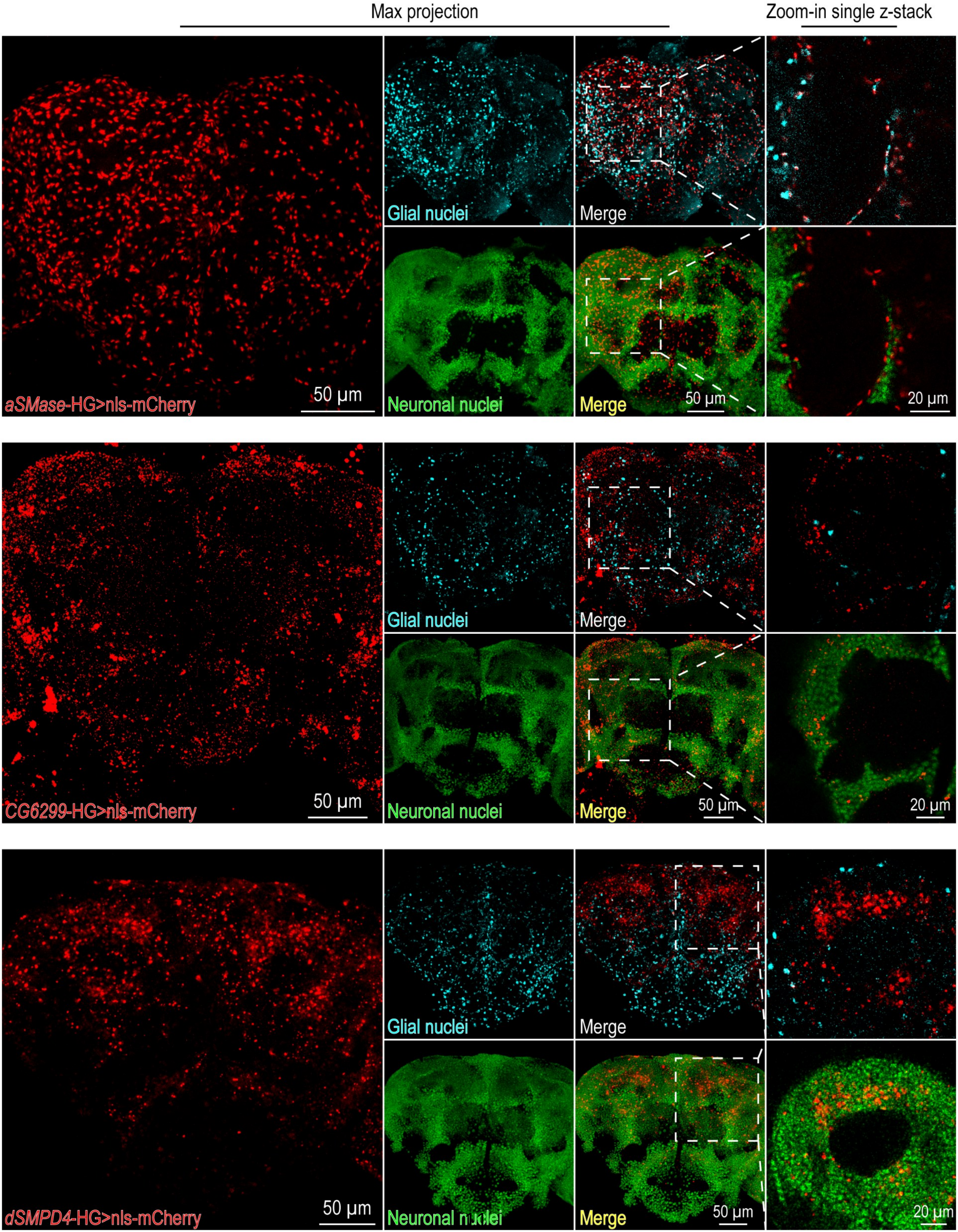

Appendix Figure S5 nls-mCherry expression in the central brain driven by HG lines of *aSMase, CG6299,* and *dSMPD4*

The cell-type expression pattern of targeted genes in the central brain of 1-week-old flies was visualized with UAS-nls-mCherry and co-stainings of neuronal (anti-Elav, green) and glial nuclei (anti-Repo; cyan).

Appendix Fig. S6

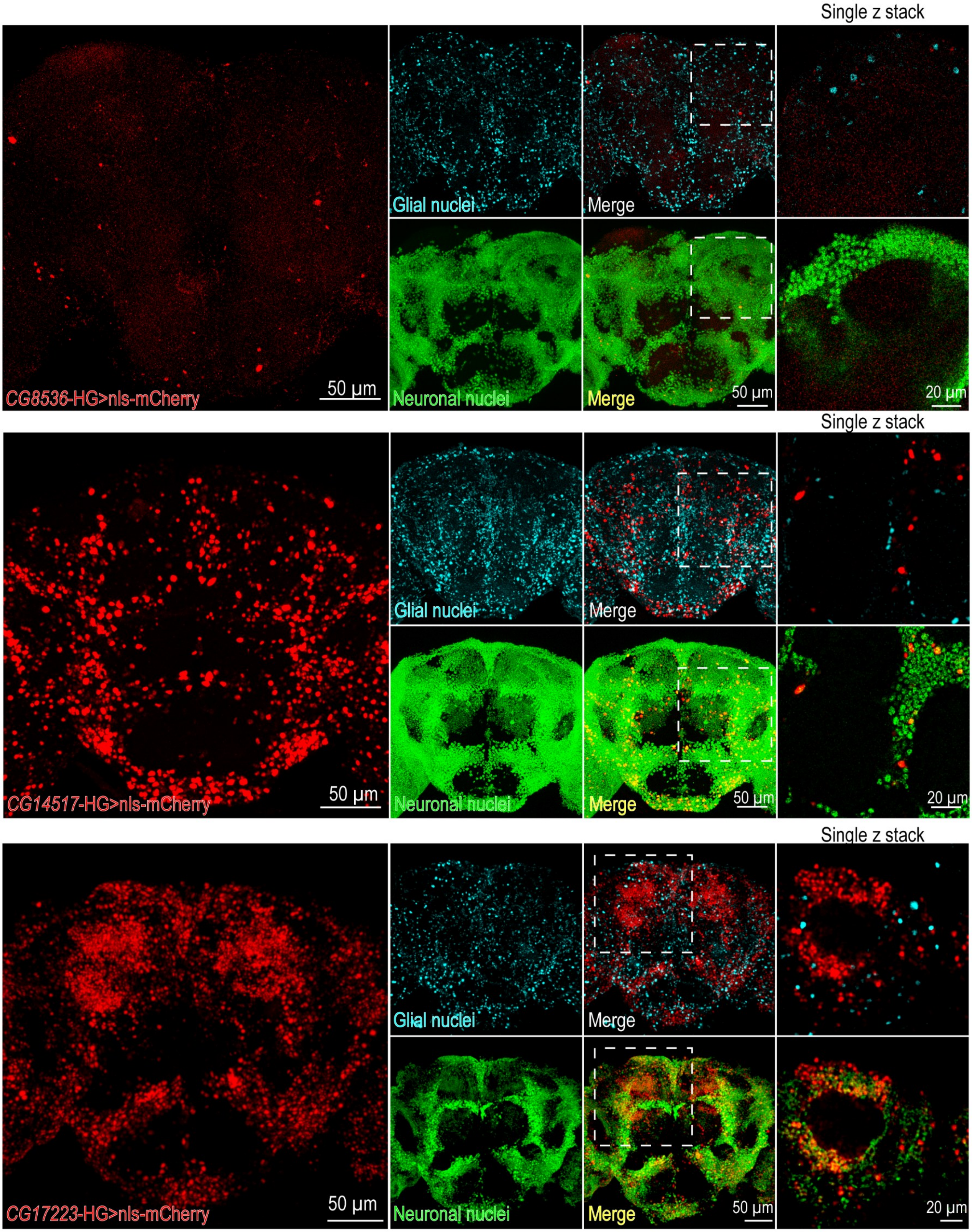

Appendix Figure S6 nls-mCherry expression in the central brain driven by HG lines of *CG8536, CG14517,* and *CG17223*

The cell-type expression pattern of targeted genes in the central brain of 1-week-old flies was visualized with UAS-nls-mCherry and co-stainings of neuronal (anti-Elav, green) and glial nuclei (anti-Repo; cyan).

Appendix Fig. S7

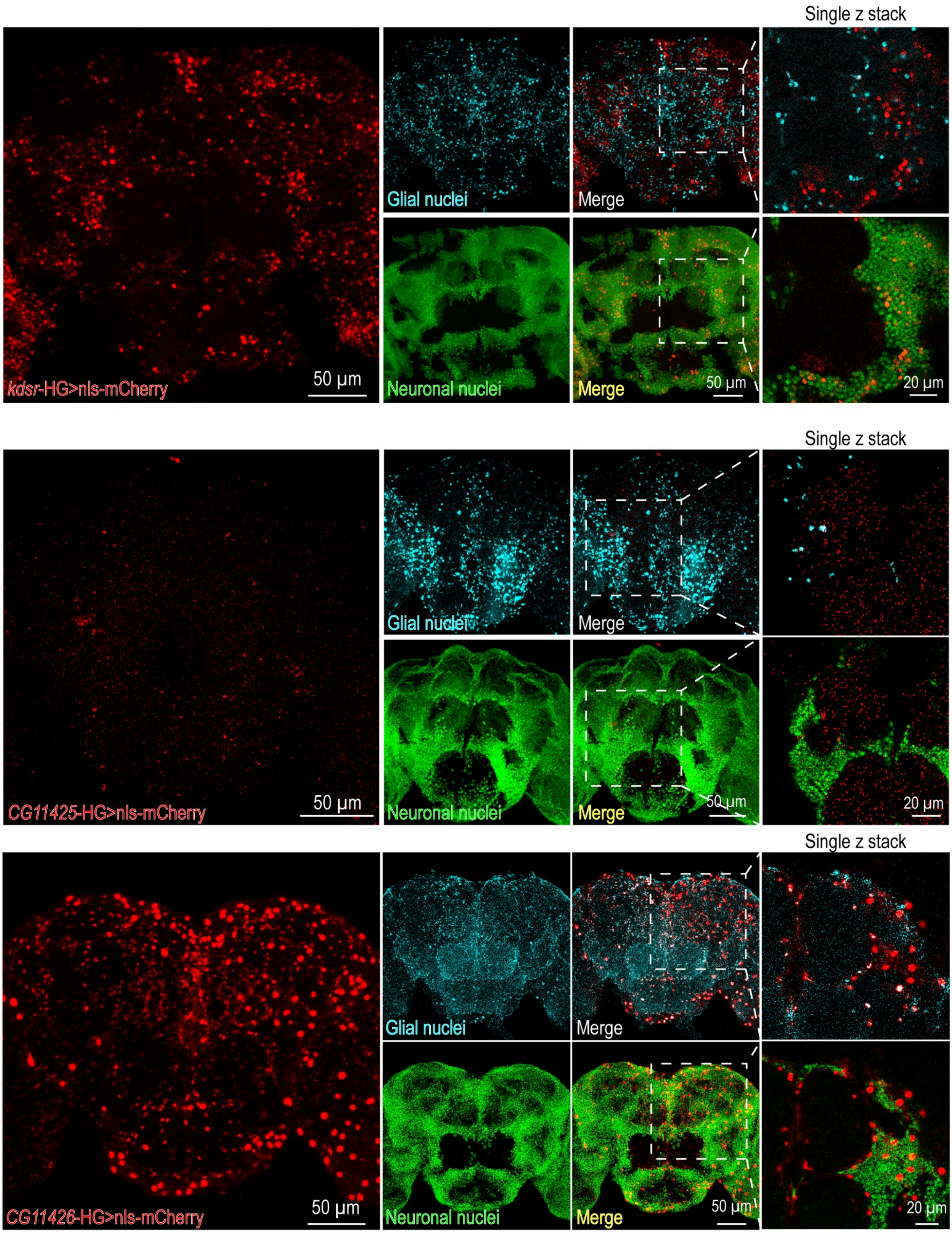

Appendix Figure S7 nls-mCherry expression in the central brain driven by HG lines of *kdsr, CG11425,* and *CG11426*

The cell-type expression pattern of targeted genes in the central brain of 1-week-old flies was visualized with UAS-nls-mCherry and co-stainings of neuronal (anti-Elav, green) and glial nuclei (anti-Repo; cyan).

Appendix Fig. S8

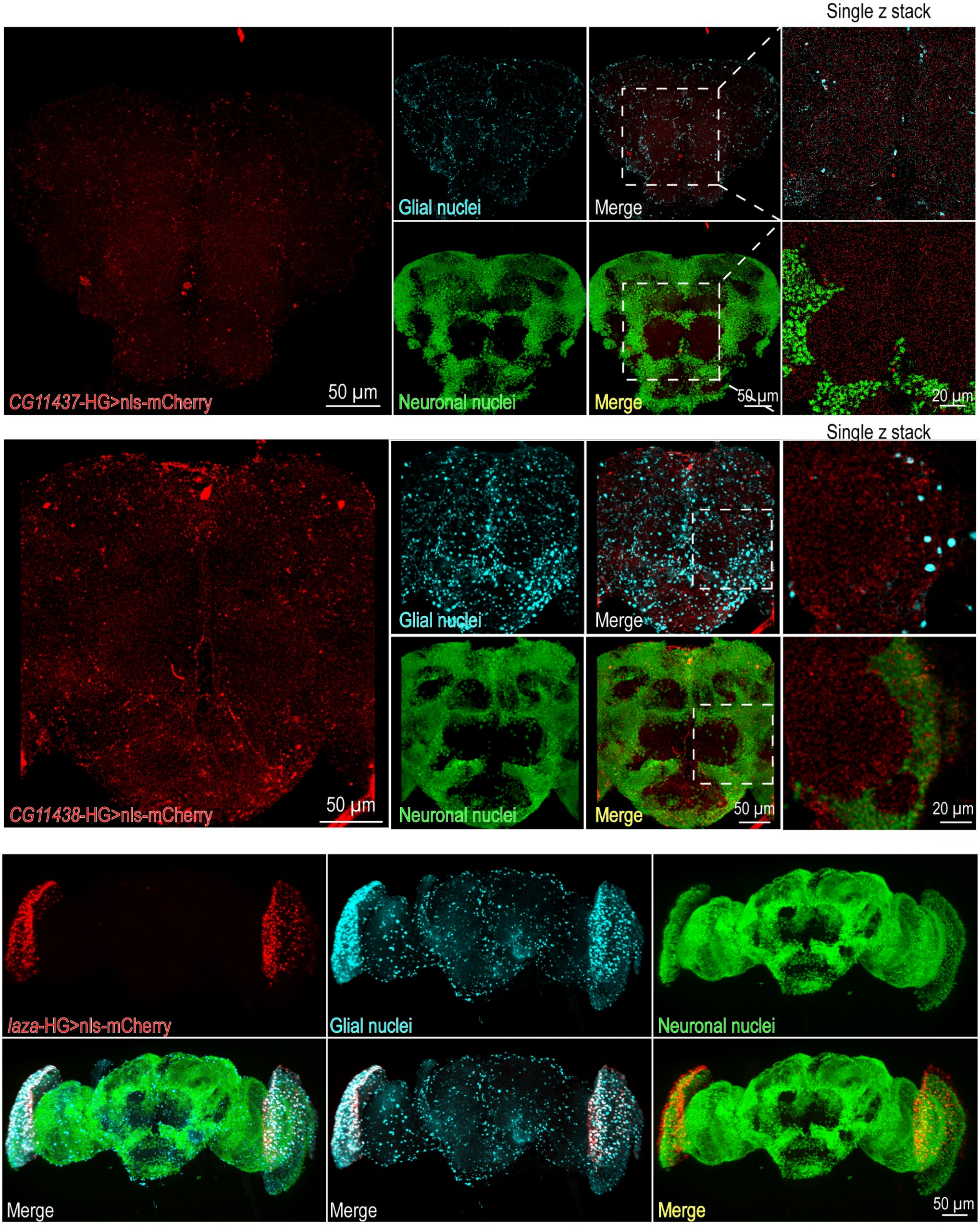

*no central brain expression
expressed in glia in the optic lobe

Appendix Figure S8 nls-mCherry expression in the central brain driven by HG lines of *CG11437, CG11438,* and *laza*

The cell-type expression pattern of targeted genes in the central brain of 1-week-old flies was visualized with UAS-nls-mCherry and co-stainings of neuronal (anti-Elav, green) and glial nuclei (anti-Repo; cyan).

Appendix Fig. S9

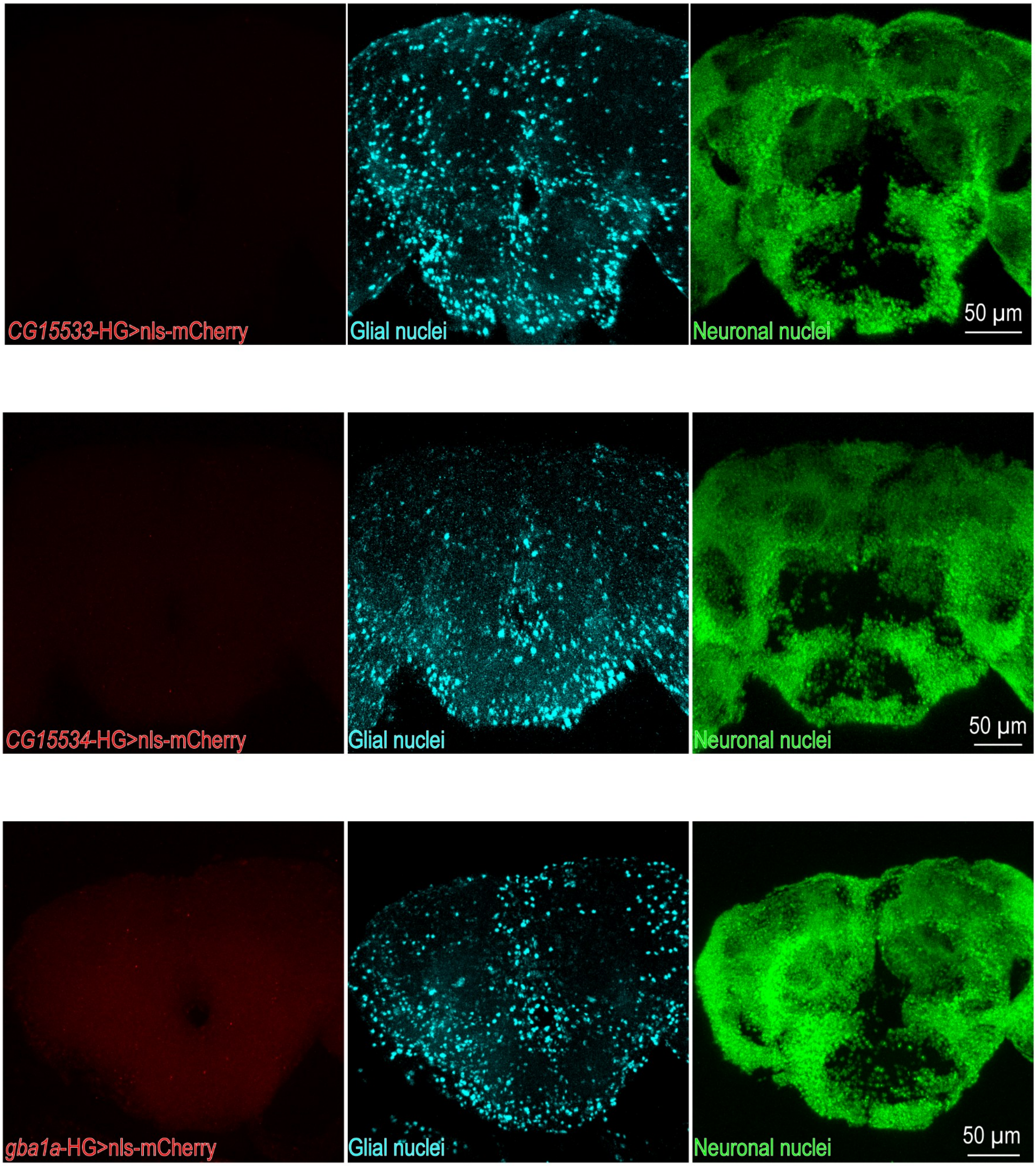

Appendix Figure S9 nls-mCherry expression in the central brain driven by HG lines of *CG15533, CG15534,* and *gba1a*

The cell-type expression pattern of targeted genes in the central brain of 1-week-old flies was visualized with UAS-nls-mCherry and co-stainings of neuronal (anti-Elav, green) and glial nuclei (anti-Repo; cyan).

Appendix Fig. S10

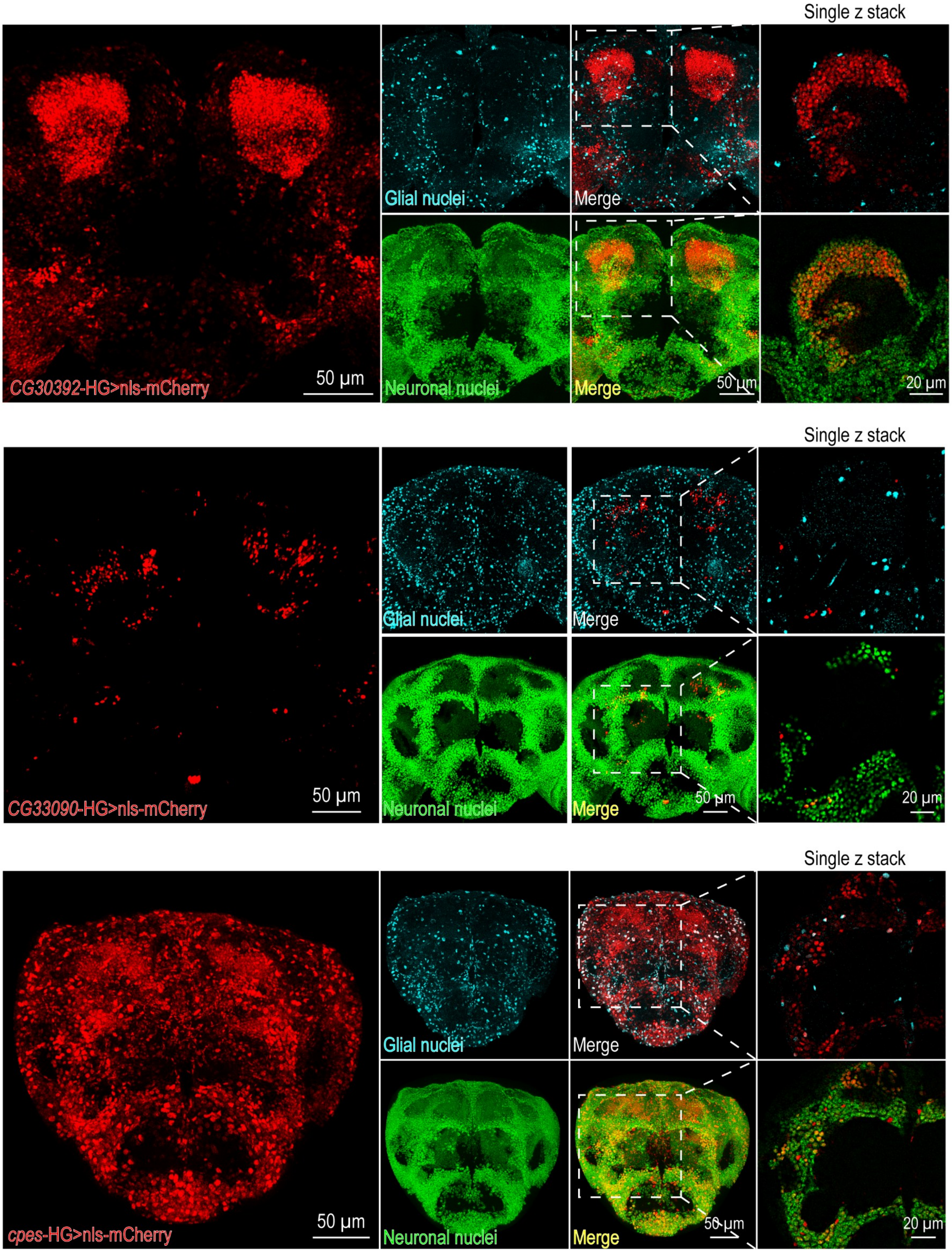

Appendix Figure S10 nls-mCherry expression in the central brain driven by HG lines of *CG30392, CG33090,* and *Cpes*

The cell-type expression pattern of targeted genes in the central brain of 1-week-old flies was visualized with UAS-nls-mCherry and co-stainings of neuronal (anti-Elav, green) and glial nuclei (anti-Repo; cyan).

Appendix Fig. S11

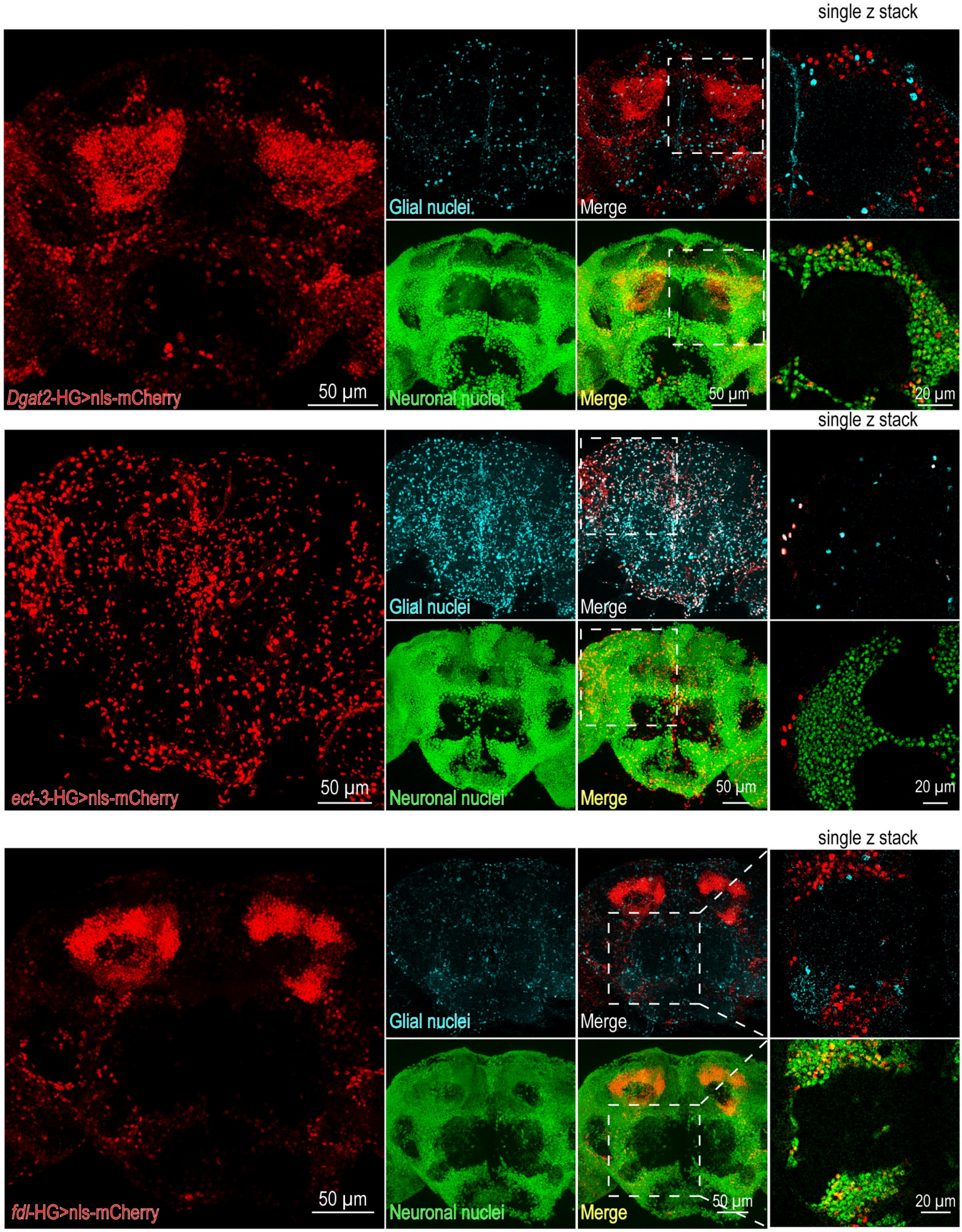

Appendix Figure S11 nls-mCherry expression in the central brain driven by HG lines of *Dgat2*, *ect-3*, and *fdl*

The cell-type expression pattern of targeted genes in the central brain of 1-week-old flies was visualized with UAS-nls-mCherry and co-stainings of neuronal (anti-Elav, green) and glial nuclei (anti-Repo; cyan).

Appendix Fig. S12

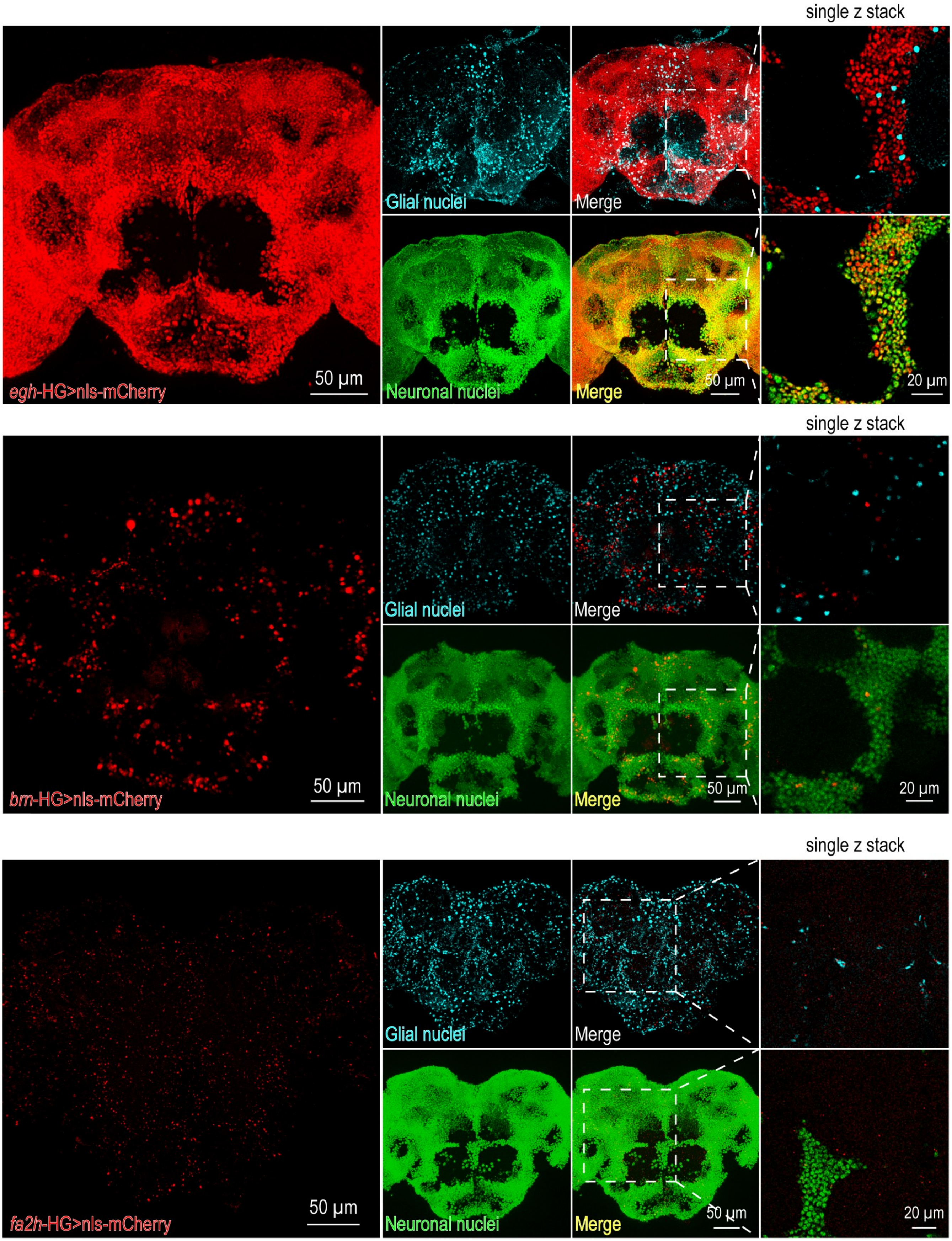

Appendix Figure S12 nls-mCherry expression in the central brain driven by HG lines of *egh*, *brn*, and *fa2h*

The cell-type expression pattern of targeted genes in the central brain of 1-week-old flies was visualized with UAS-nls-mCherry and co-stainings of neuronal (anti-Elav, green) and glial nuclei (anti-Repo; cyan).

Appendix Fig. S13

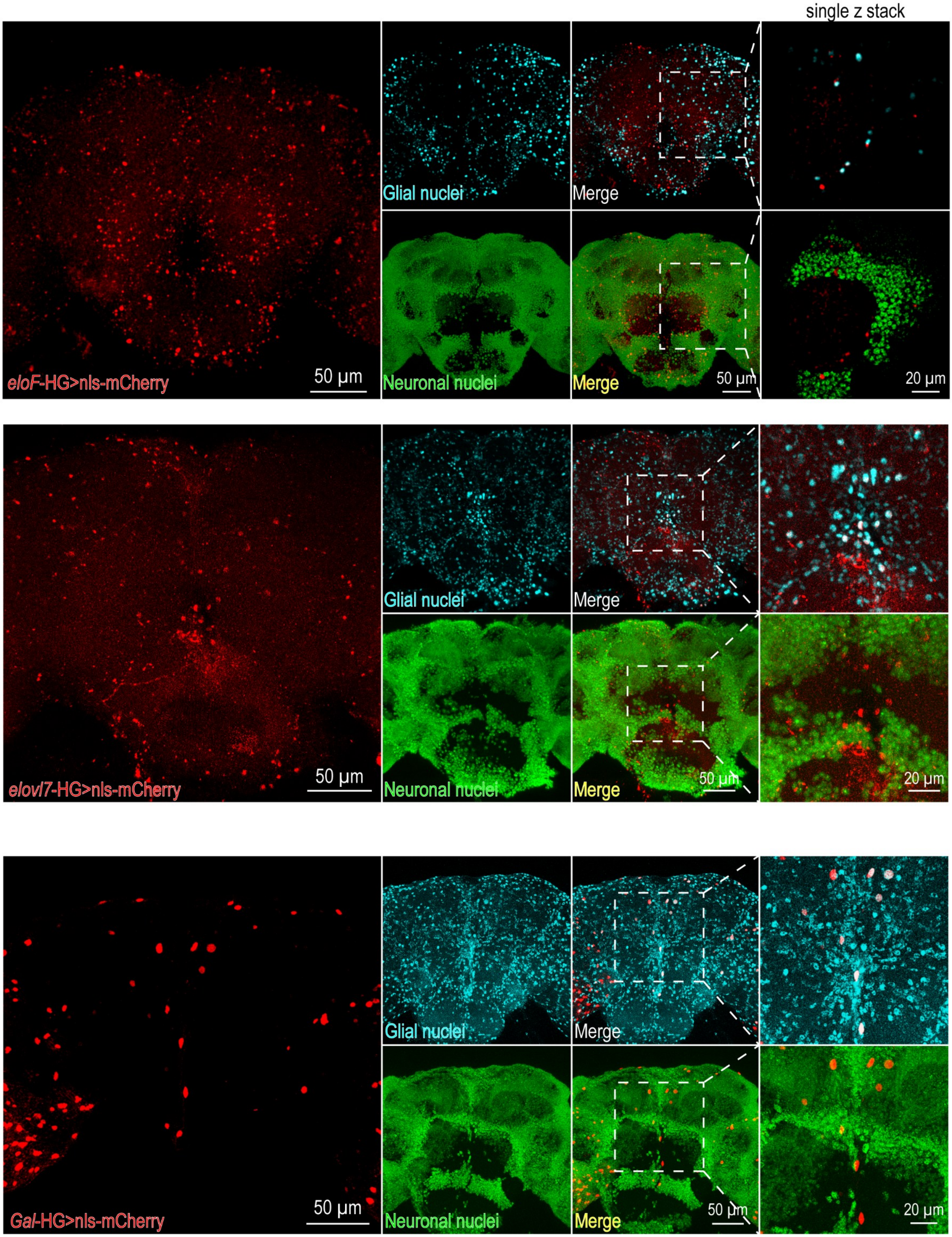

Appendix Figure S13 nls-mCherry expression in the central brain driven by HG lines of *eloF*, *elovl7*, and *Gal*

The cell-type expression pattern of targeted genes in the central brain of 1-week-old flies was visualized with UAS-nls-mCherry and co-stainings of neuronal (anti-Elav, green) and glial nuclei (anti-Repo; cyan).

Appendix Fig. S14

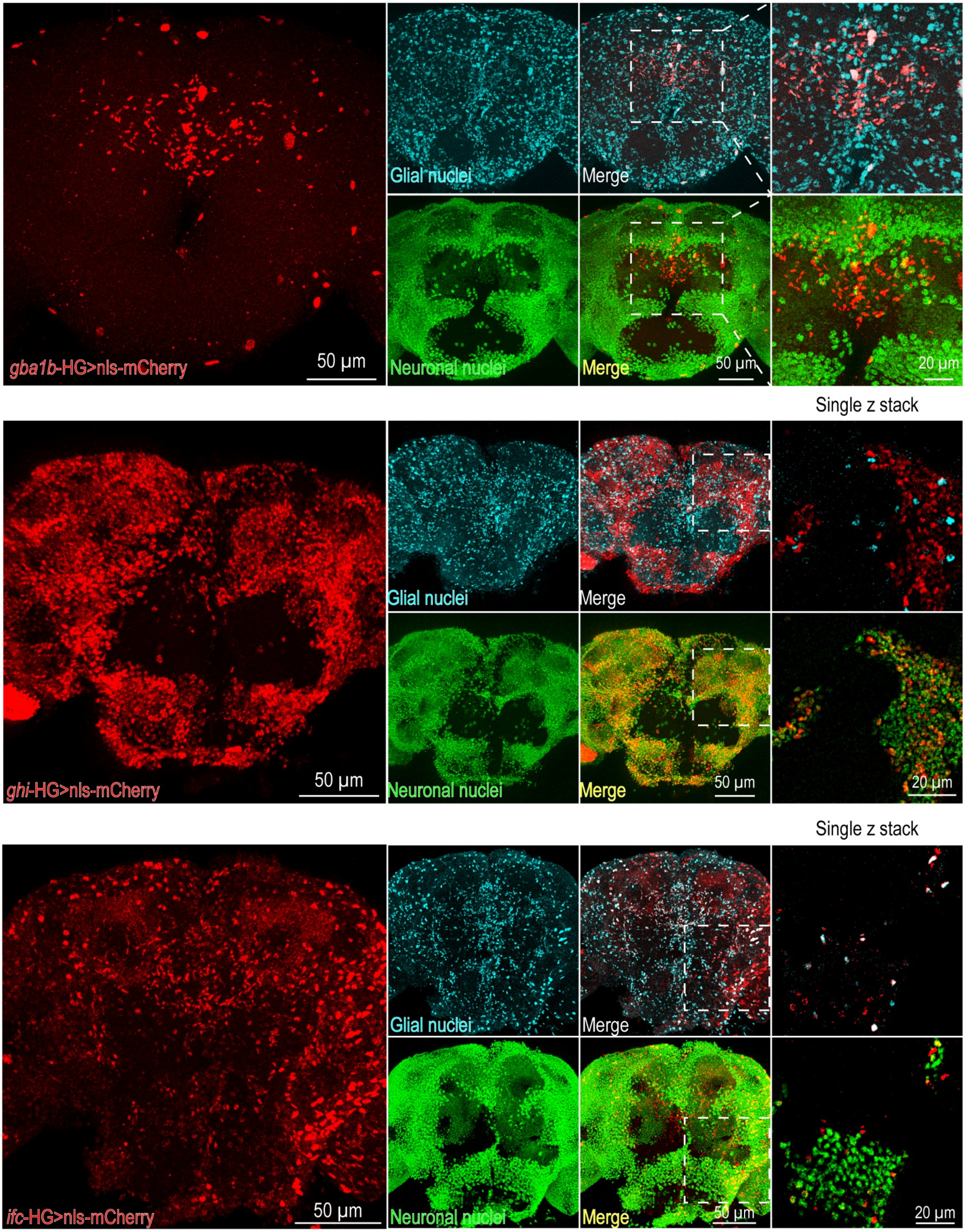

Appendix Figure S14 nls-mCherry expression in the central brain driven by HG lines of *gba1b*, *ghi*, and *ifc*

The cell-type expression pattern of targeted genes in the central brain of 1-week-old flies was visualized with UAS-nls-mCherry and co-stainings of neuronal (anti-Elav, green) and glial nuclei (anti-Repo; cyan).

Appendix Fig. S15

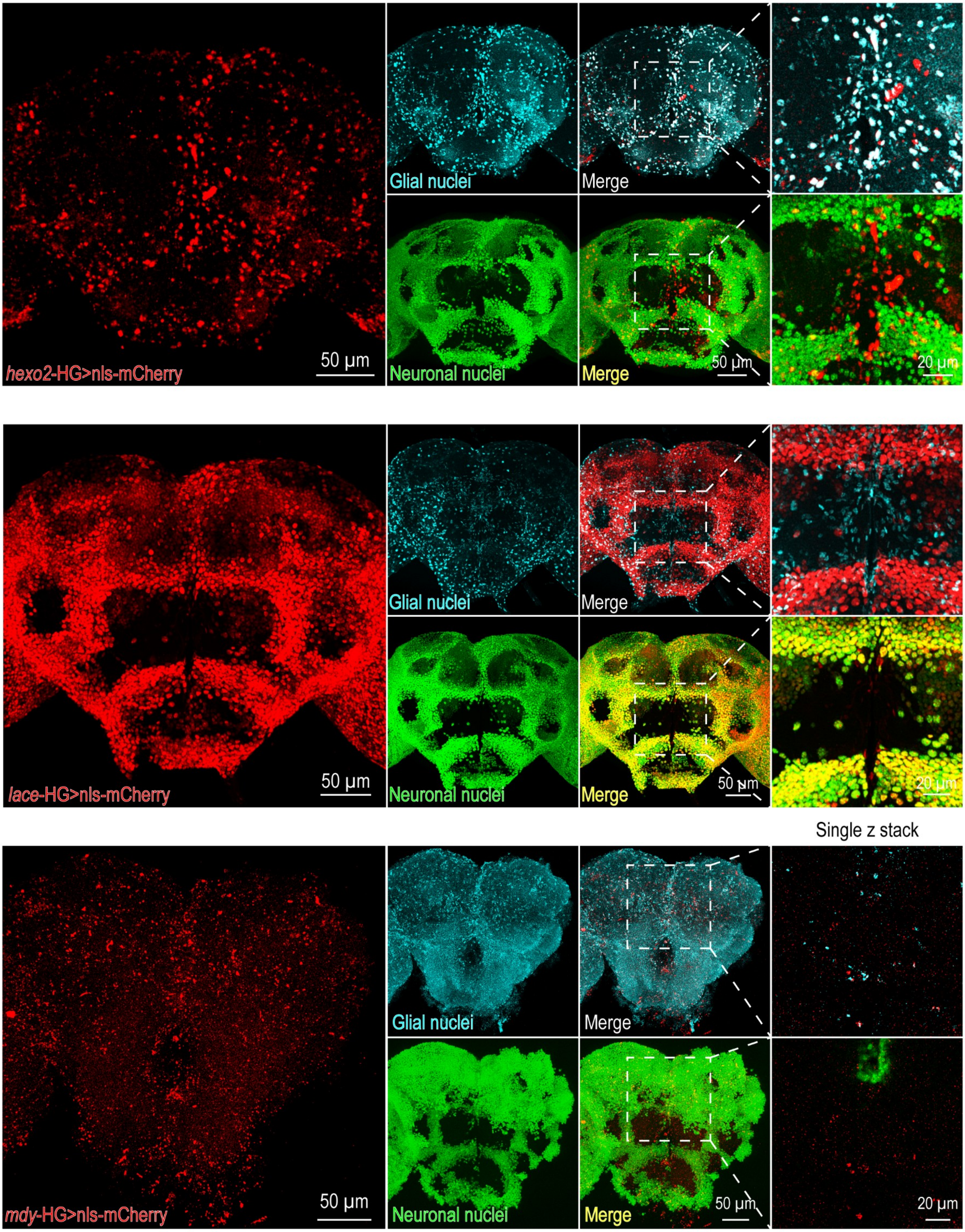

Appendix Figure S15 nls-mCherry expression in the central brain driven by HG lines of *hexo2*, *lace*, and *mdy*

The cell-type expression pattern of targeted genes in the central brain of 1-week-old flies was visualized with UAS-nls-mCherry and co-stainings of neuronal (anti-Elav, green) and glial nuclei (anti-Repo; cyan).

Appendix Fig. S16

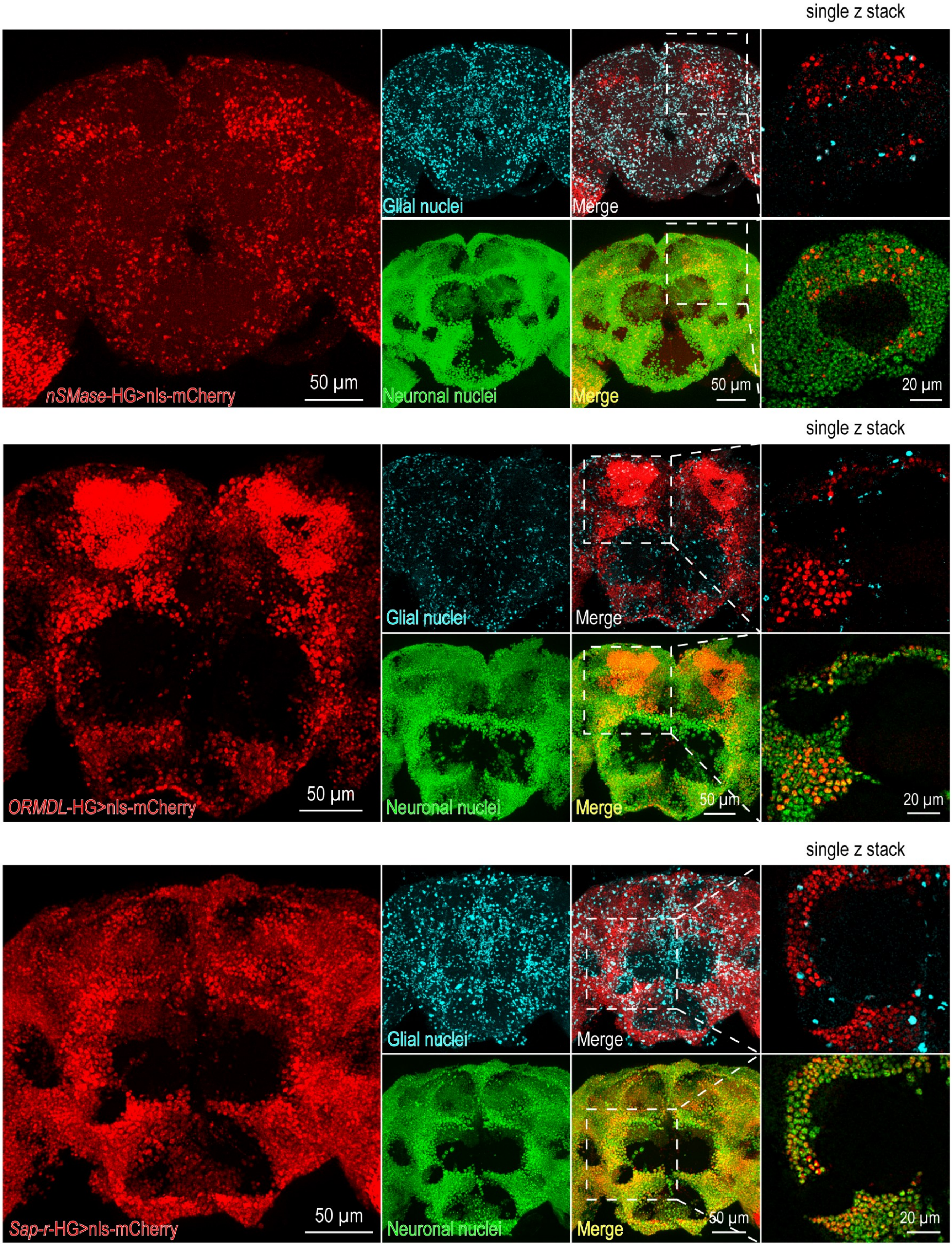

Appendix Figure S16 nls-mCherry expression in the central brain driven by HG lines of *nSMase*, *ORMDL*, and *Sap-r*

The cell-type expression pattern of targeted genes in the central brain of 1-week-old flies was visualized with UAS-nls-mCherry and co-stainings of neuronal (anti-Elav, green) and glial nuclei (anti-Repo; cyan).

Appendix Fig. S17

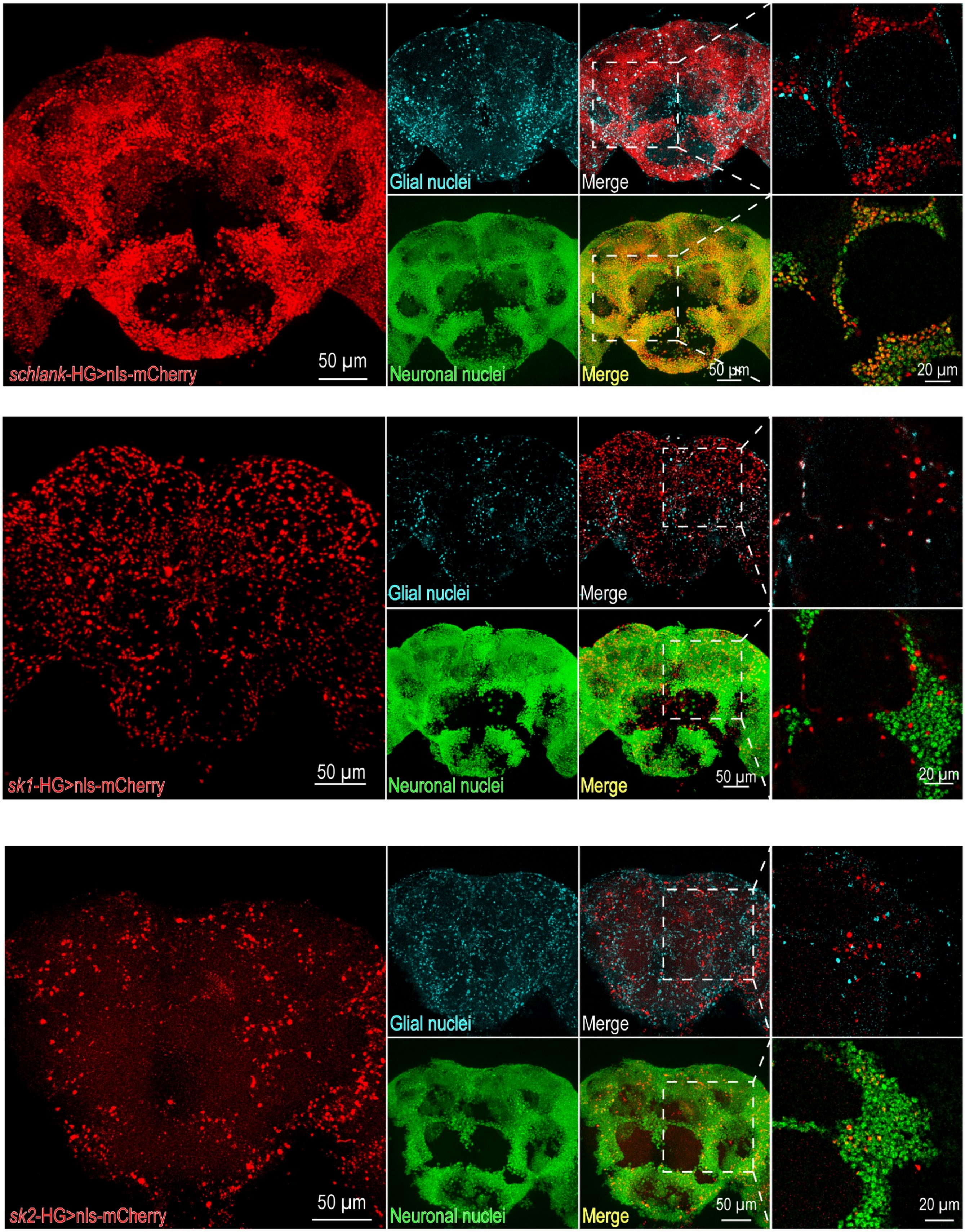

Appendix Figure S17 nls-mCherry expression in the central brain driven by HG lines of *schlank, sk1,* and *sk2*.

The cell-type expression pattern of targeted genes in the central brain of 1-week-old flies was visualized with UAS-nls-mCherry and co-stainings of neuronal (anti-Elav, green) and glial nuclei (anti-Repo; cyan).

Appendix Fig. S18

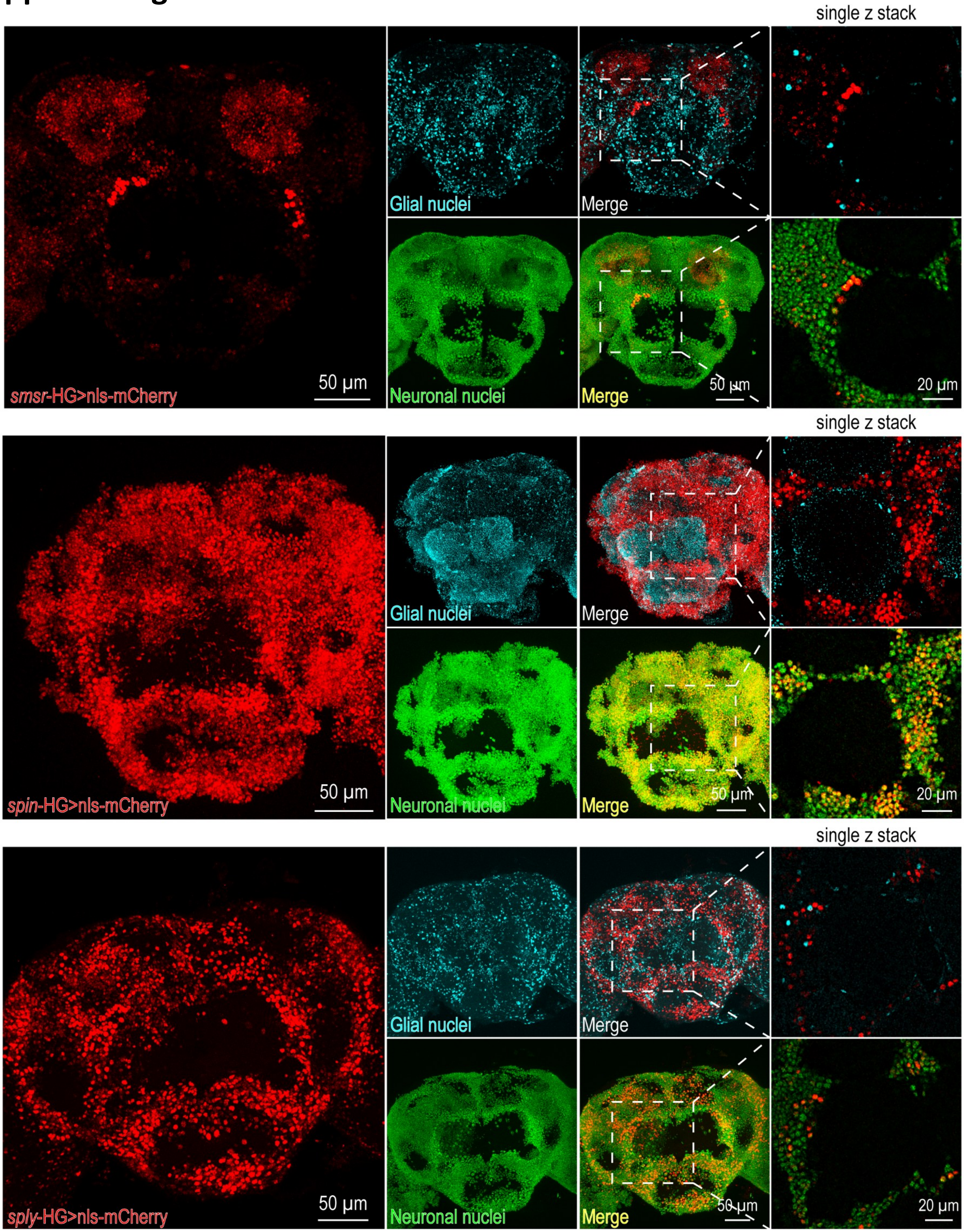

Appendix Figure S18 nls-mCherry expression in the central brain driven by HG lines of *smsr*, *spin*, and *sply*

The cell-type expression pattern of targeted genes in the central brain of 1-week-old flies was visualized with UAS-nls-mCherry and co-stainings of neuronal (anti-Elav, green) and glial nuclei (anti-Repo; cyan).

Appendix Fig. S19

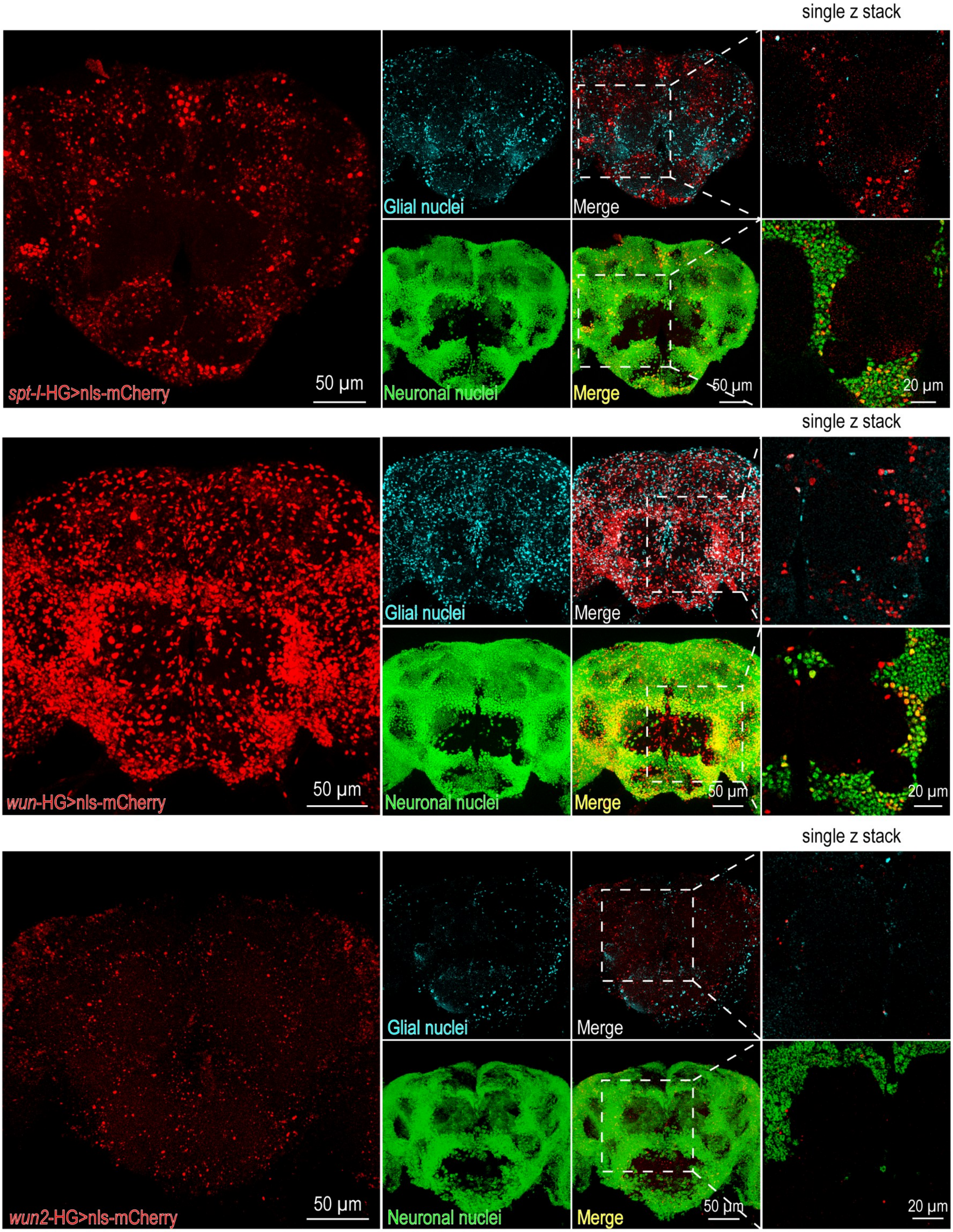

Appendix Figure S19 nls-mCherry expression in the central brain driven by HG lines of *spt-I*, *wun*, and *wun2*

The cell-type expression pattern of targeted genes in the central brain of 1-week-old flies was visualized with UAS-nls-mCherry and co-stainings of neuronal (anti-Elav, green) and glial nuclei (anti-Repo; cyan).

Appendix Fig. S20

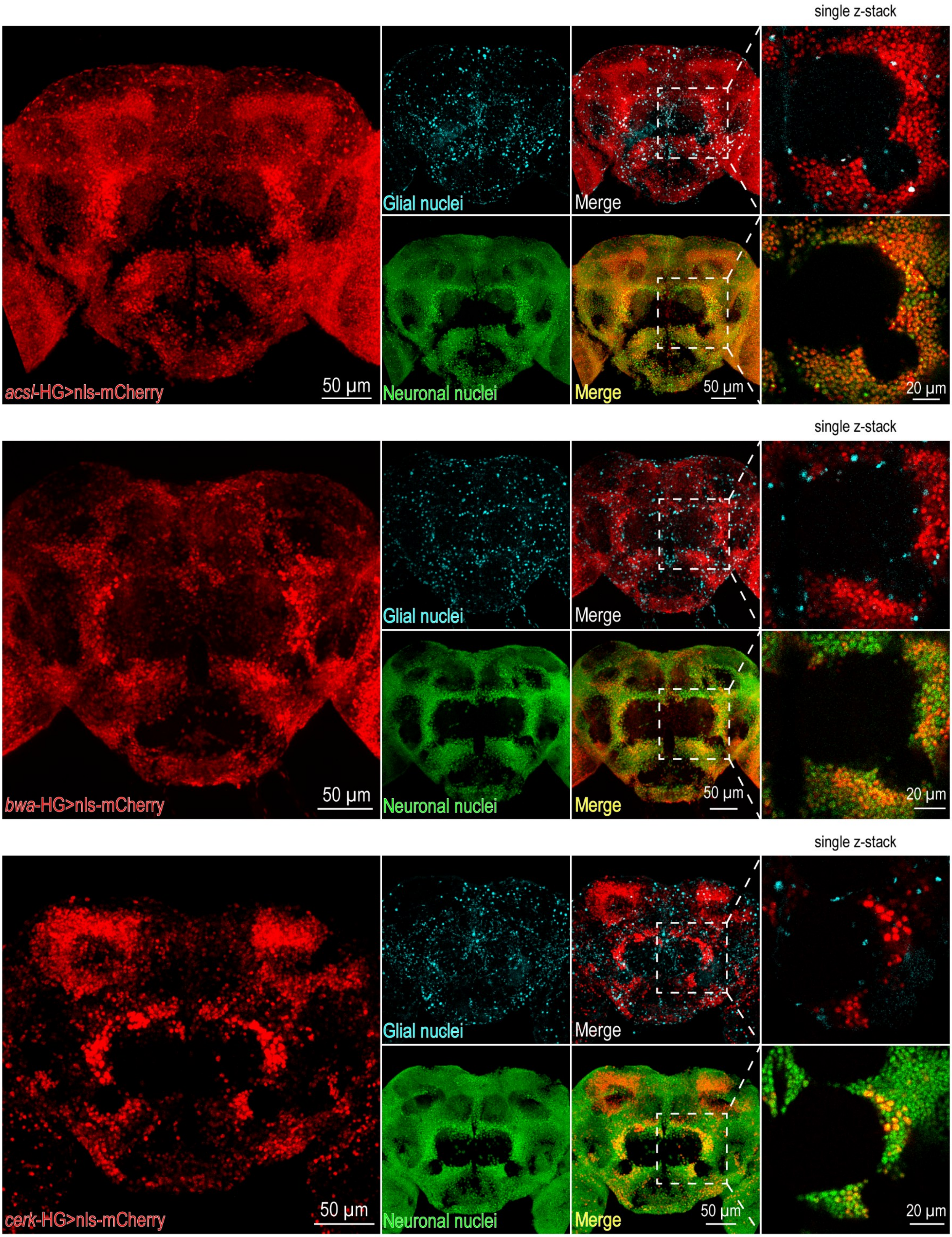

Appendix Figure S20 nls-mCherry expression in the central brain driven by HG lines of *acsl*, *bwa*, and *cerk*

The cell-type expression pattern of targeted genes in the central brain of 1-week-old flies was visualized with UAS-nls-mCherry and co-stainings of neuronal (anti-Elav, green) and glial nuclei (anti-Repo; cyan).

Appendix Fig. S21

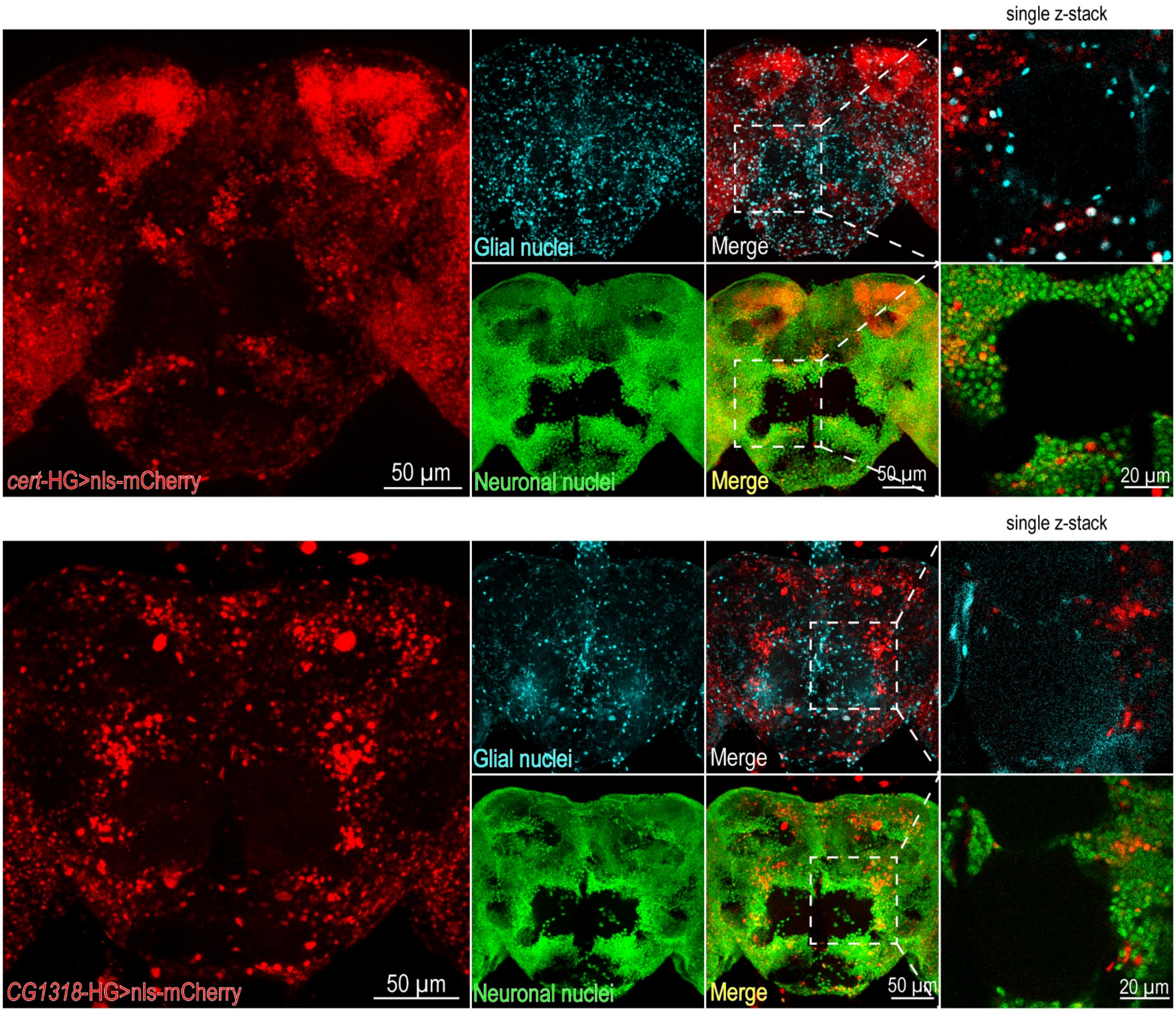

Appendix Figure S21 nls-mCherry expression in the central brain driven by HG lines of *cert* and *CG1318*

The cell-type expression pattern of targeted genes in the central brain of 1-week-old flies was visualized with UAS-nls-mCherry and co-stainings of neuronal (anti-Elav, green) and glial nuclei (anti-Repo; cyan).

Appendix Fig. S22

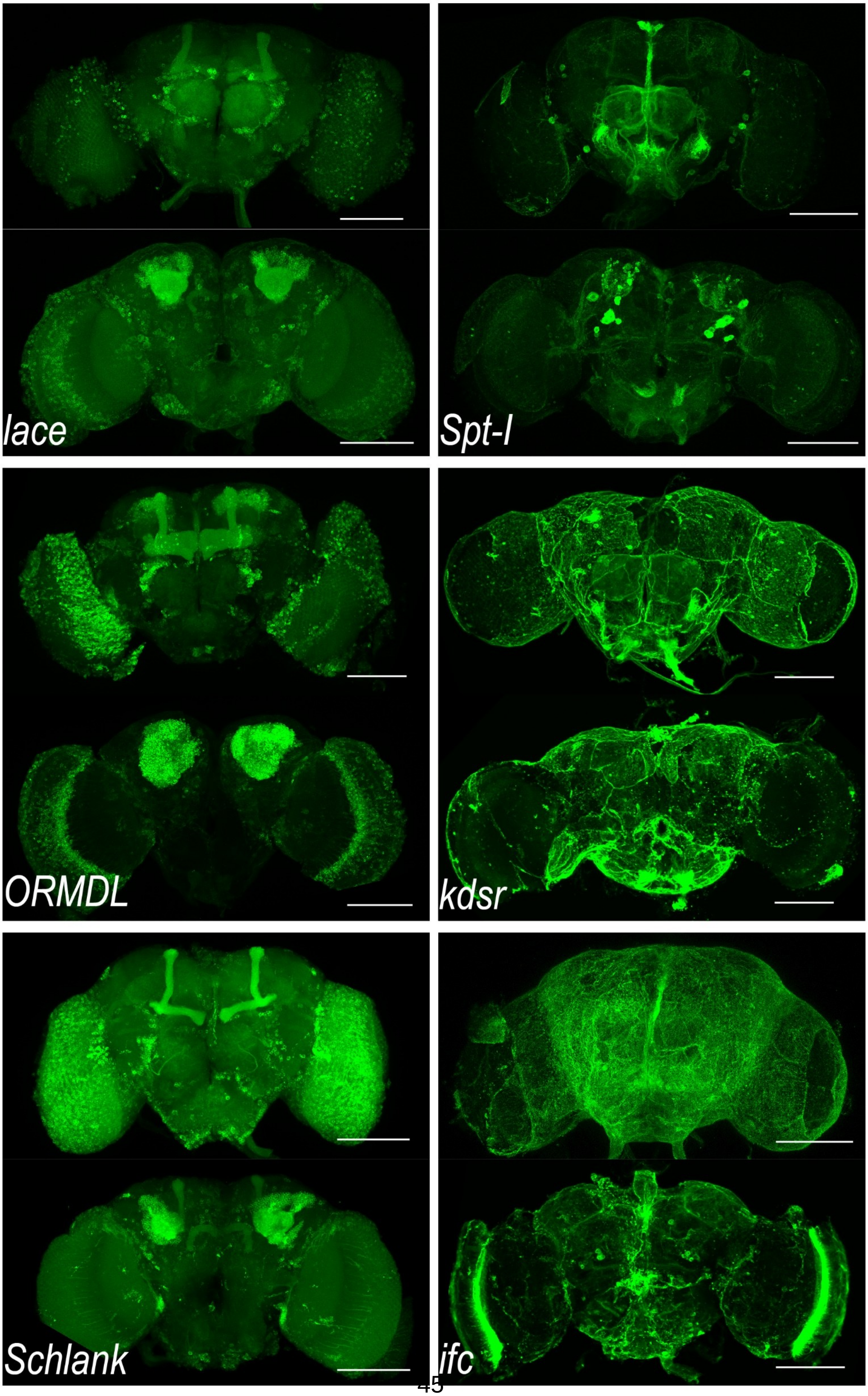

Appendix Figure S22 mCD8GFP expression driven by HG lines
Expression patterns of SPL regulators in adult brains were observed with mCD8GFP driven by HG. The top is the anterior, and the bottom is the posterior view of max projections of z-stack confocal images. The targeted gene is indicated at the bottom left. Scale bar: 100 µm.

Appendix Fig. S23

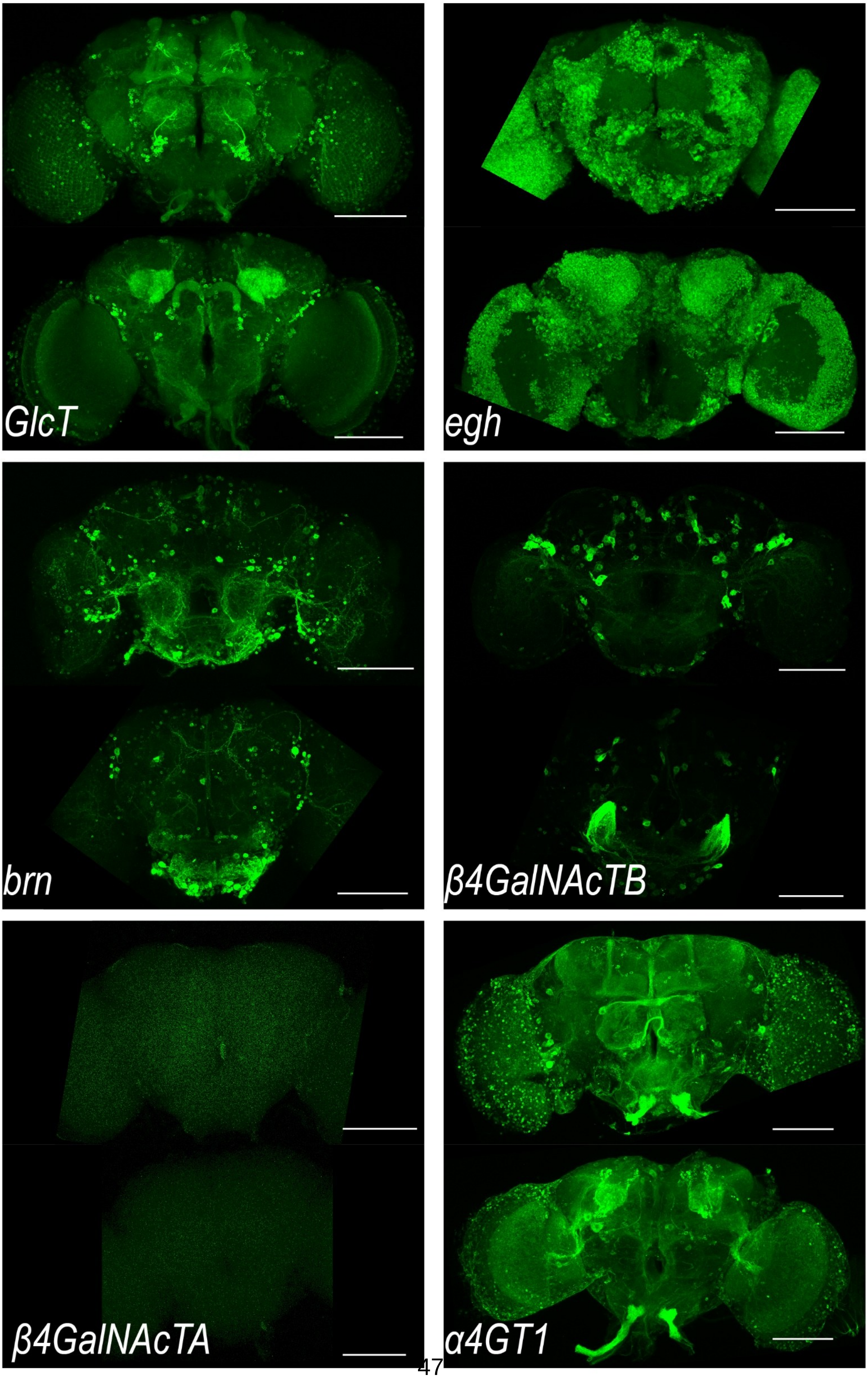

Appendix Figure S23 mCD8GFP expression driven by HG lines
Expression patterns of SPL regulators in adult brains were observed with mCD8GFP driven by HG. The top is the anterior, and the bottom is the posterior view of max projections of z-stack confocal images. The targeted gene is indicated at the bottom left. Scale bar: 100 µm.

Appendix Fig. S24

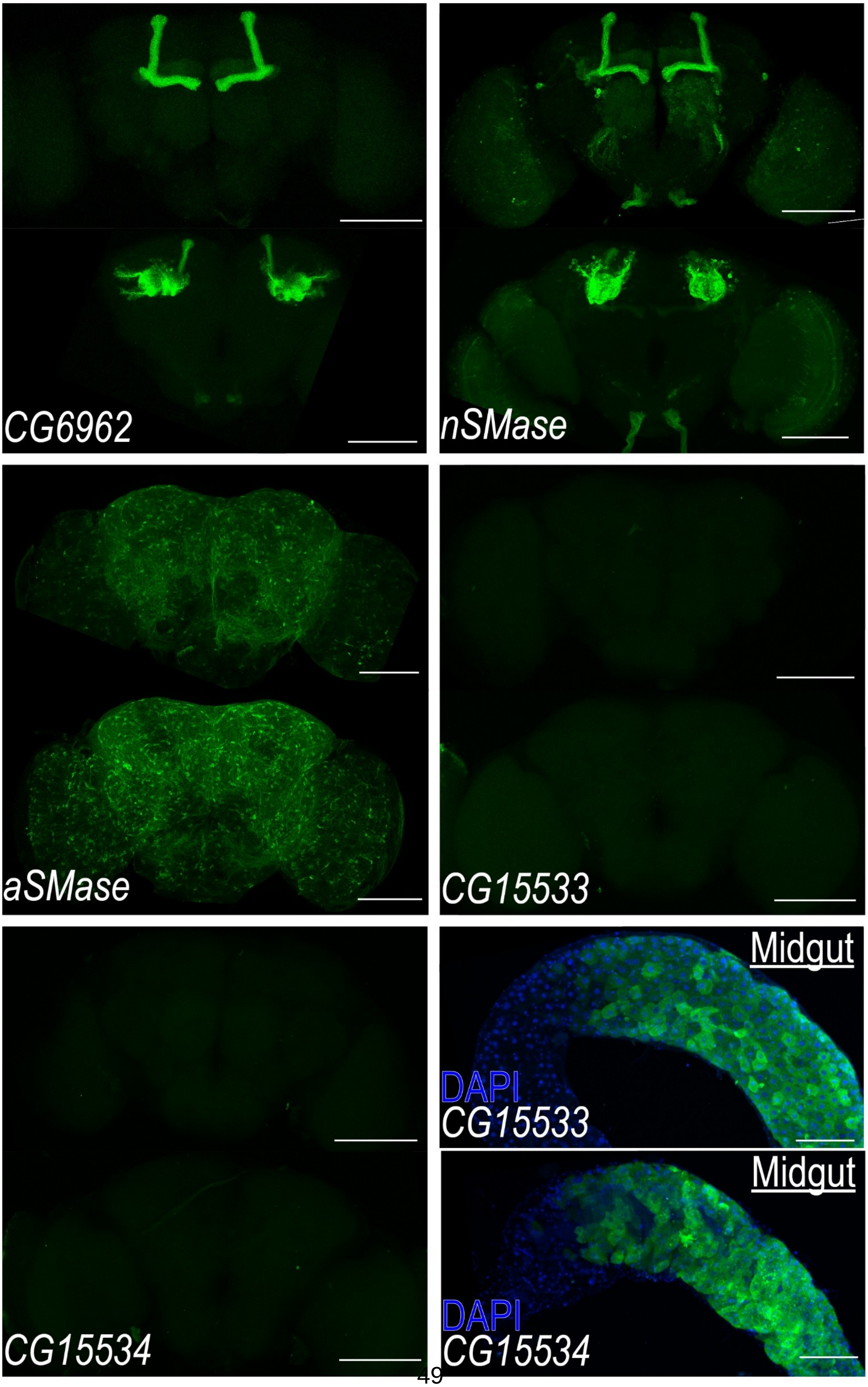

Appendix Figure S24 mCD8GFP expression driven by HG lines
Expression patterns of SPL regulators in adult brains were observed with mCD8GFP driven by HG. The top is the anterior, and the bottom is the posterior view of max projections of z-stack confocal images. The targeted gene is indicated at the bottom left. Scale bar: 100 µm. Midgut expressions of CG15534 and CG15534 were observed with mCD8GFP driven by HG. Scale bar: 100 µm.

Appendix Fig. S25

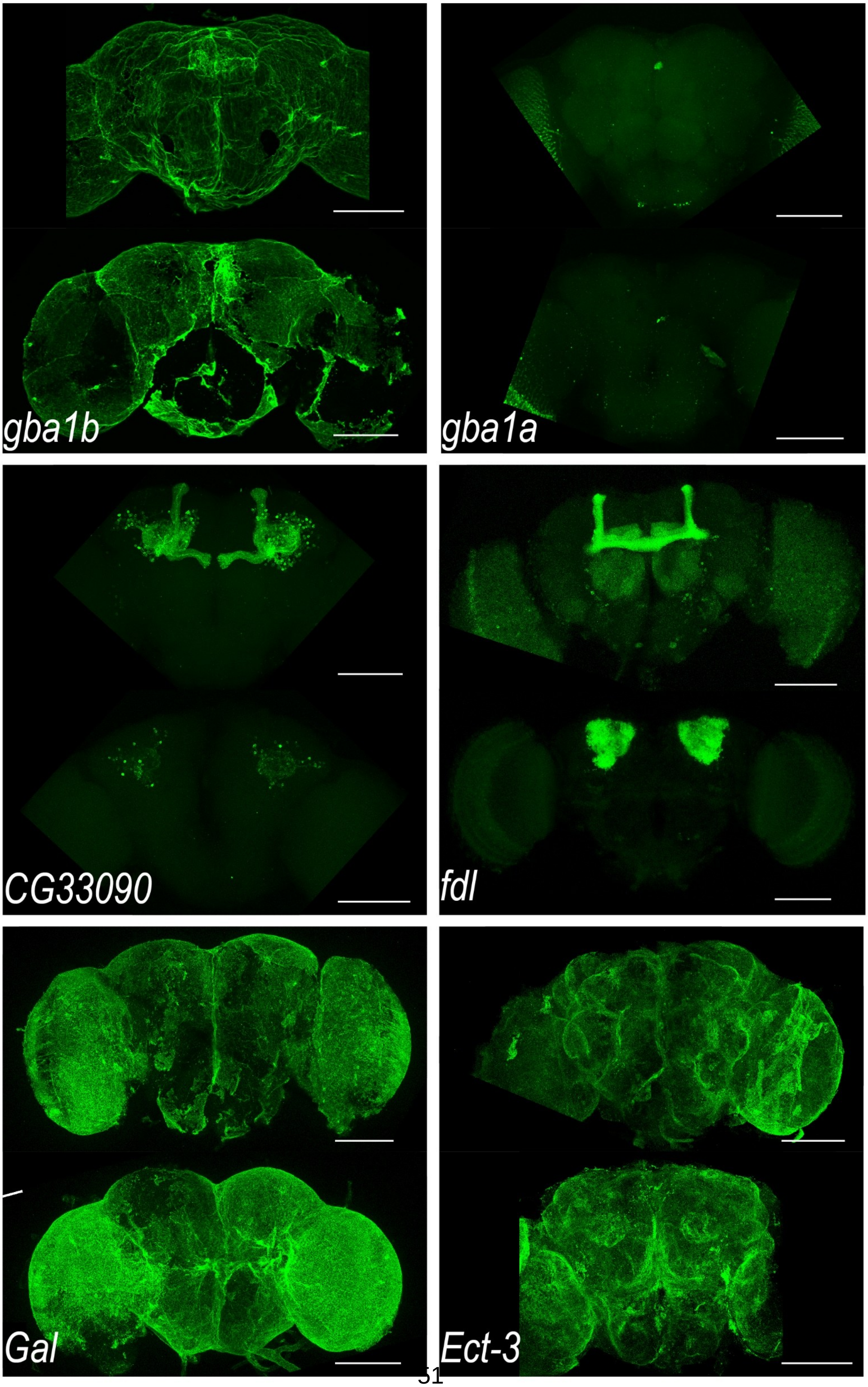

Appendix Figure S25 mCD8GFP expression driven by HG lines
Expression patterns of SPL regulators in adult brains were observed with mCD8GFP driven by HG. The top is the anterior, and the bottom is the posterior view of max projections of z-stack confocal images. The targeted gene is indicated at the bottom left. Scale bar: 100 µm.

Appendix Fig. S26

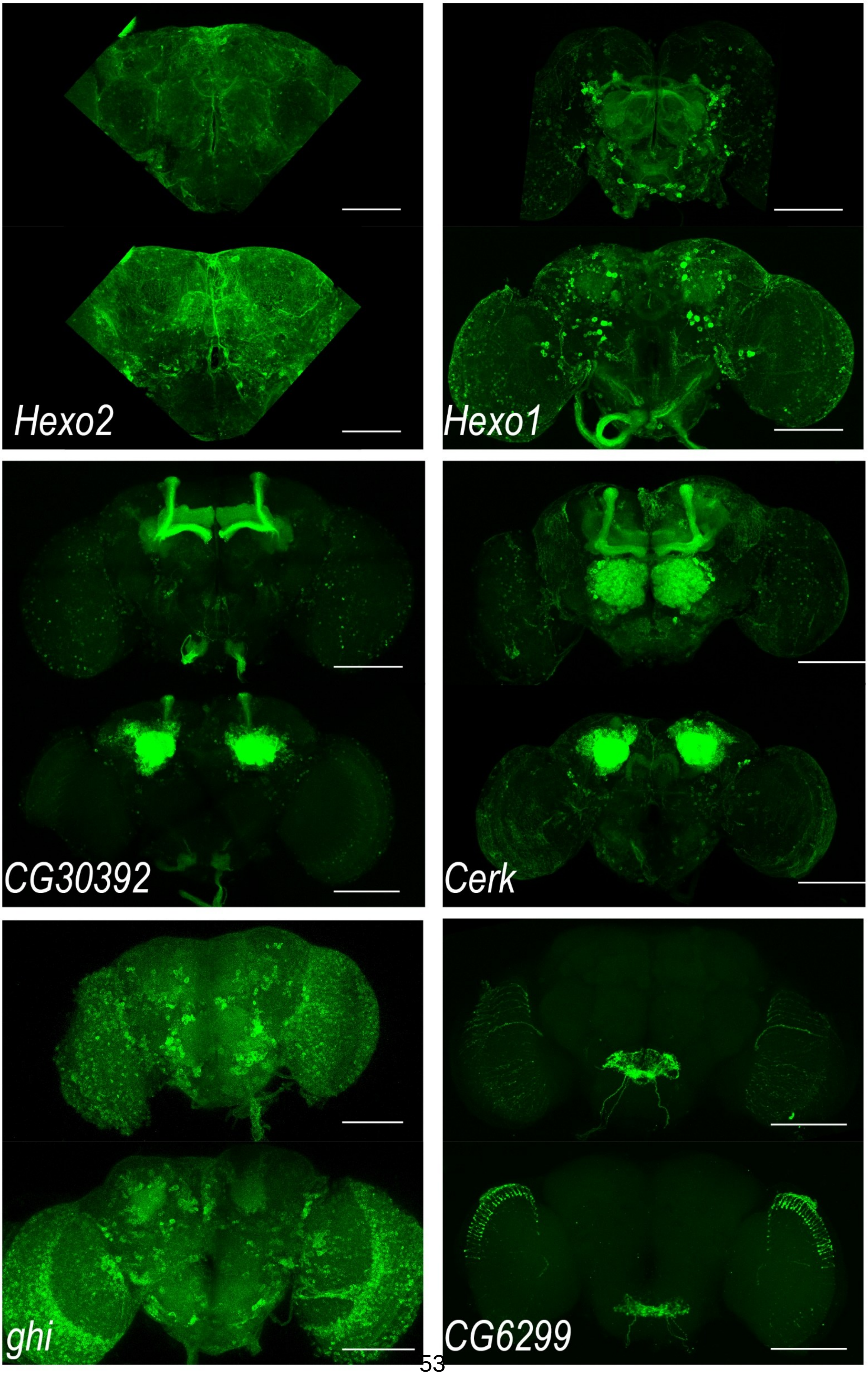

Appendix Figure S26 mCD8GFP expression driven by HG lines
Expression patterns of SPL regulators in adult brains were observed with mCD8GFP driven by HG. The top is the anterior, and the bottom is the posterior view of max projections of z-stack confocal images. The targeted gene is indicated at the bottom left. Scale bar: 100 µm.

Appendix Fig. S27

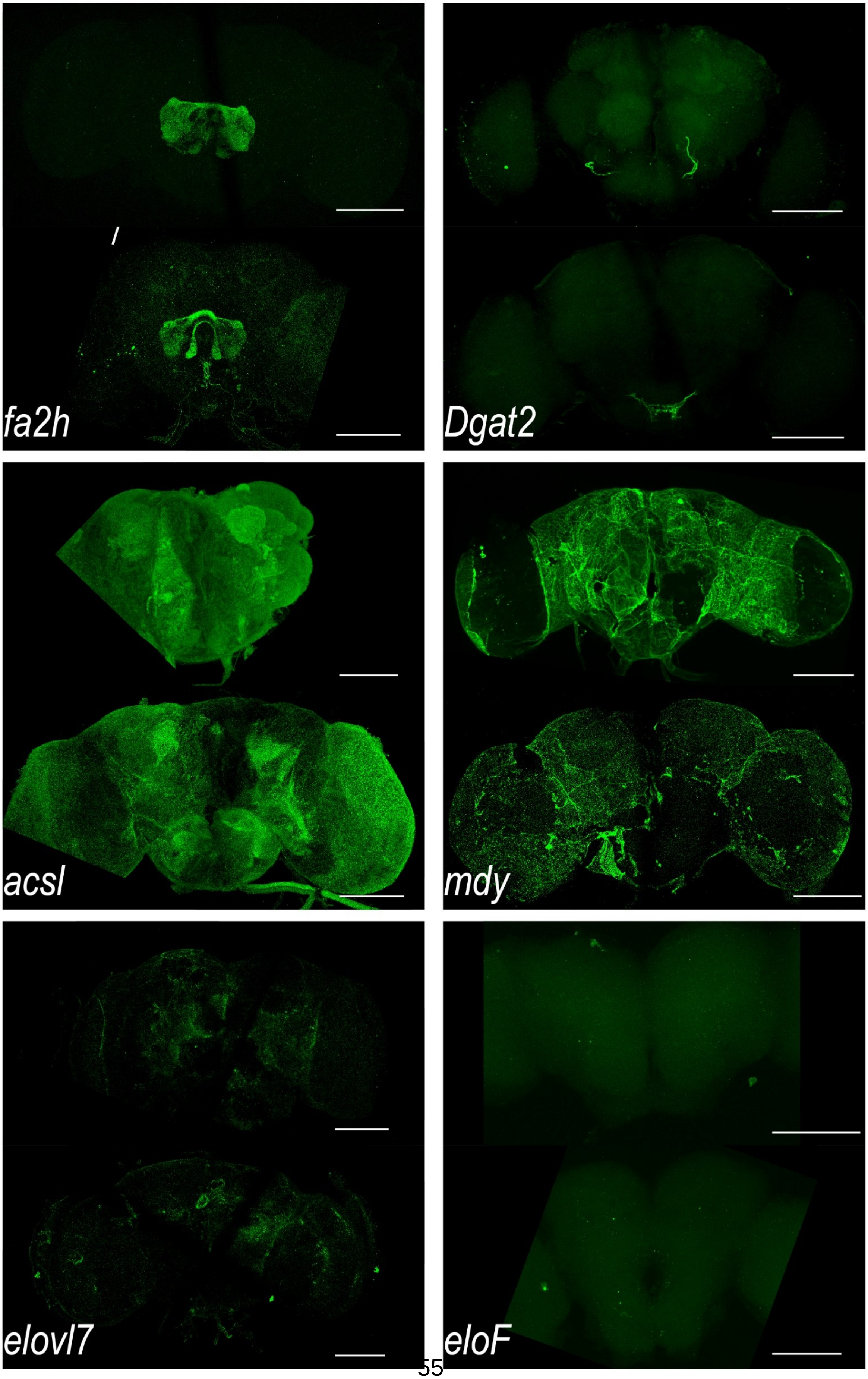

Appendix Figure S27 mCD8GFP expression driven by HG lines
Expression patterns of SPL regulators in adult brains were observed with mCD8GFP driven by HG. The top is the anterior, and the bottom is the posterior view of max projections of z-stack confocal images. The targeted gene is indicated at the bottom left. Scale bar: 100 µm.

Appendix Fig. S28

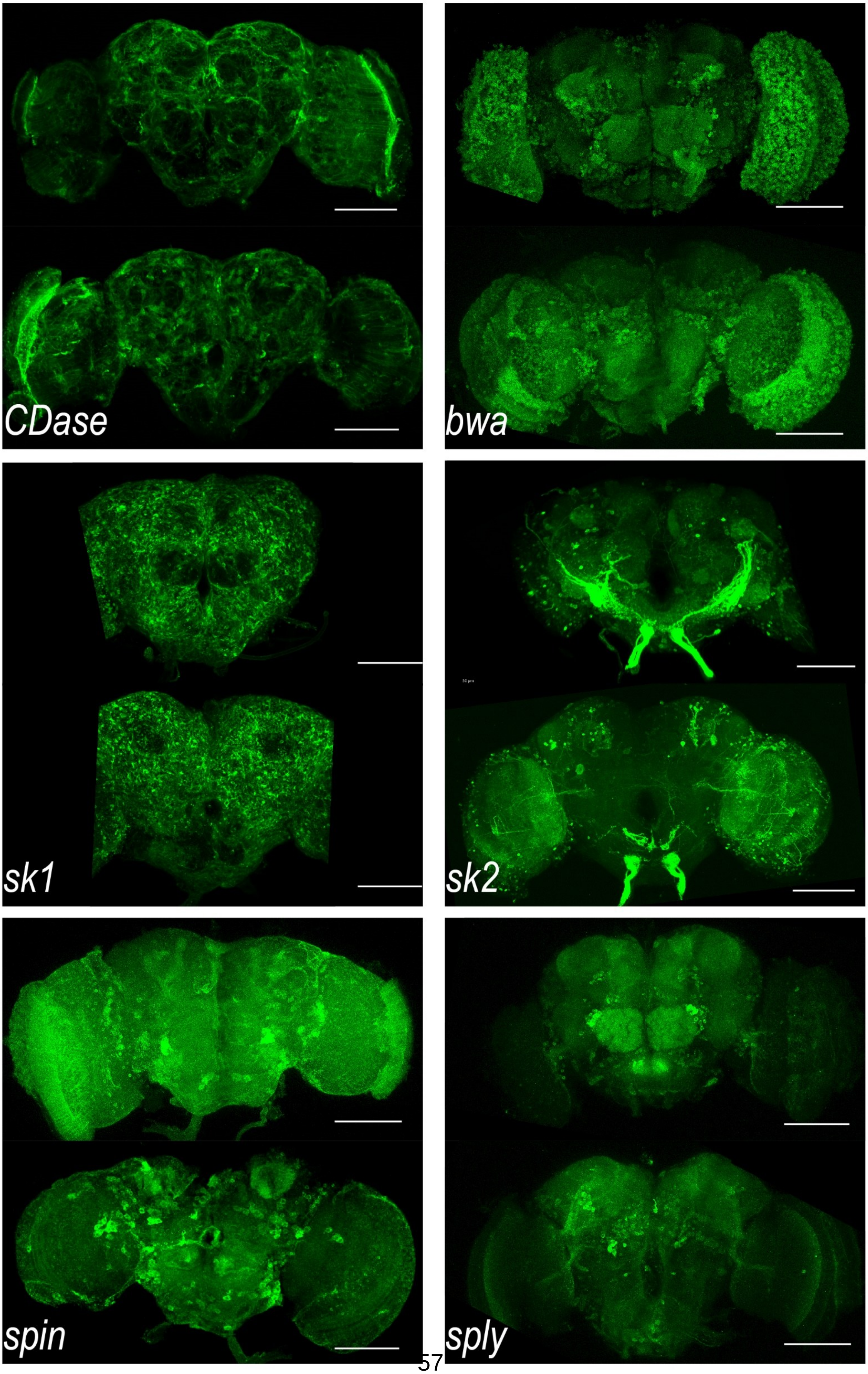

Appendix Figure S28 mCD8GFP expression driven by HG lines
Expression patterns of SPL regulators in adult brains were observed with mCD8GFP driven by HG. The top is the anterior, and the bottom is the posterior view of max projections of z-stack confocal images. The targeted gene is indicated at the bottom left. Scale bar: 100 µm.

Appendix Fig. S29

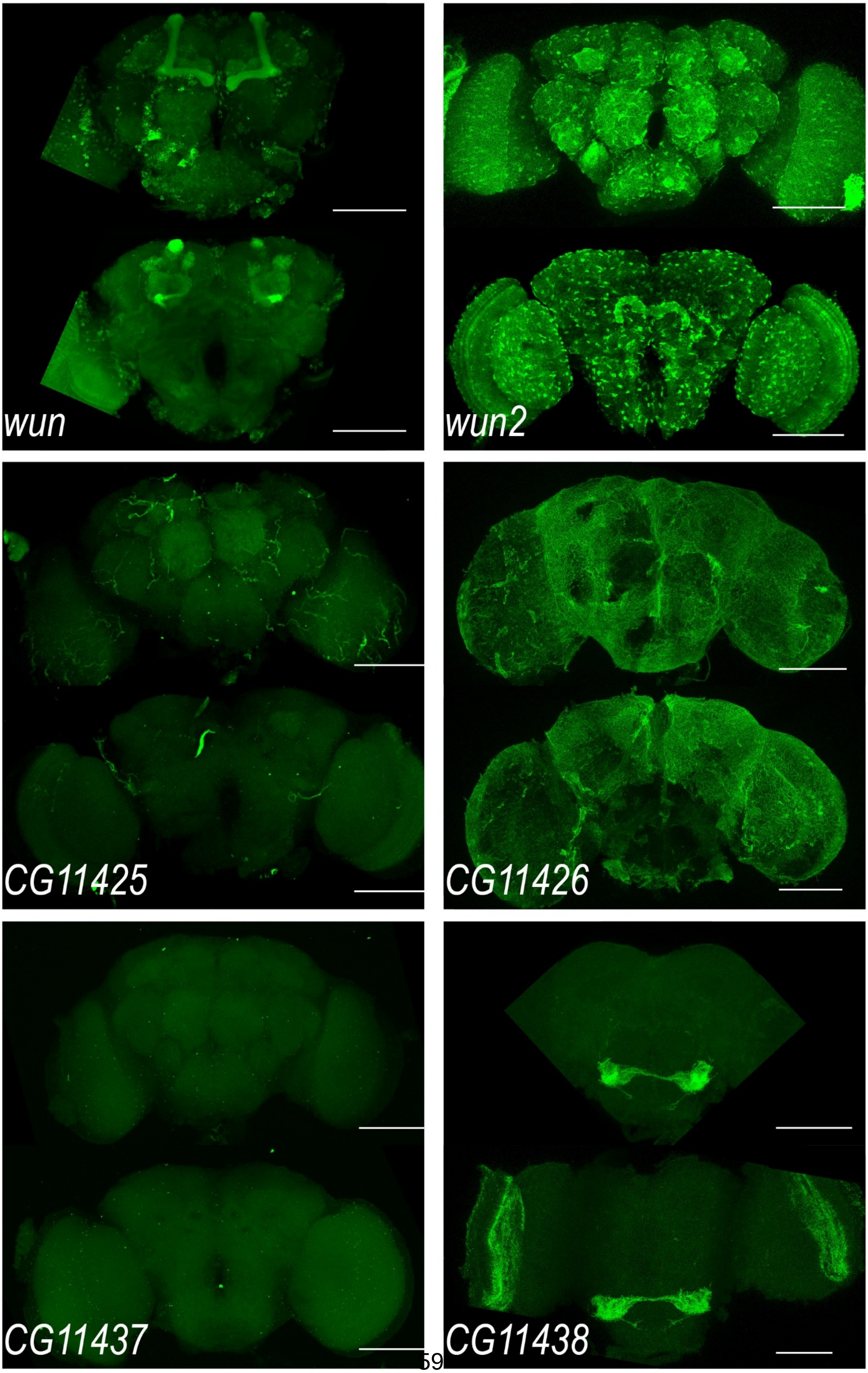

Appendix Figure S29 mCD8GFP expression driven by HG lines
Expression patterns of SPL regulators in adult brains were observed with mCD8GFP driven by HG. The top is the anterior, and the bottom is the posterior view of max projections of z-stack confocal images. The targeted gene is indicated at the bottom left. Scale bar: 100 µm.

Appendix Fig. S30

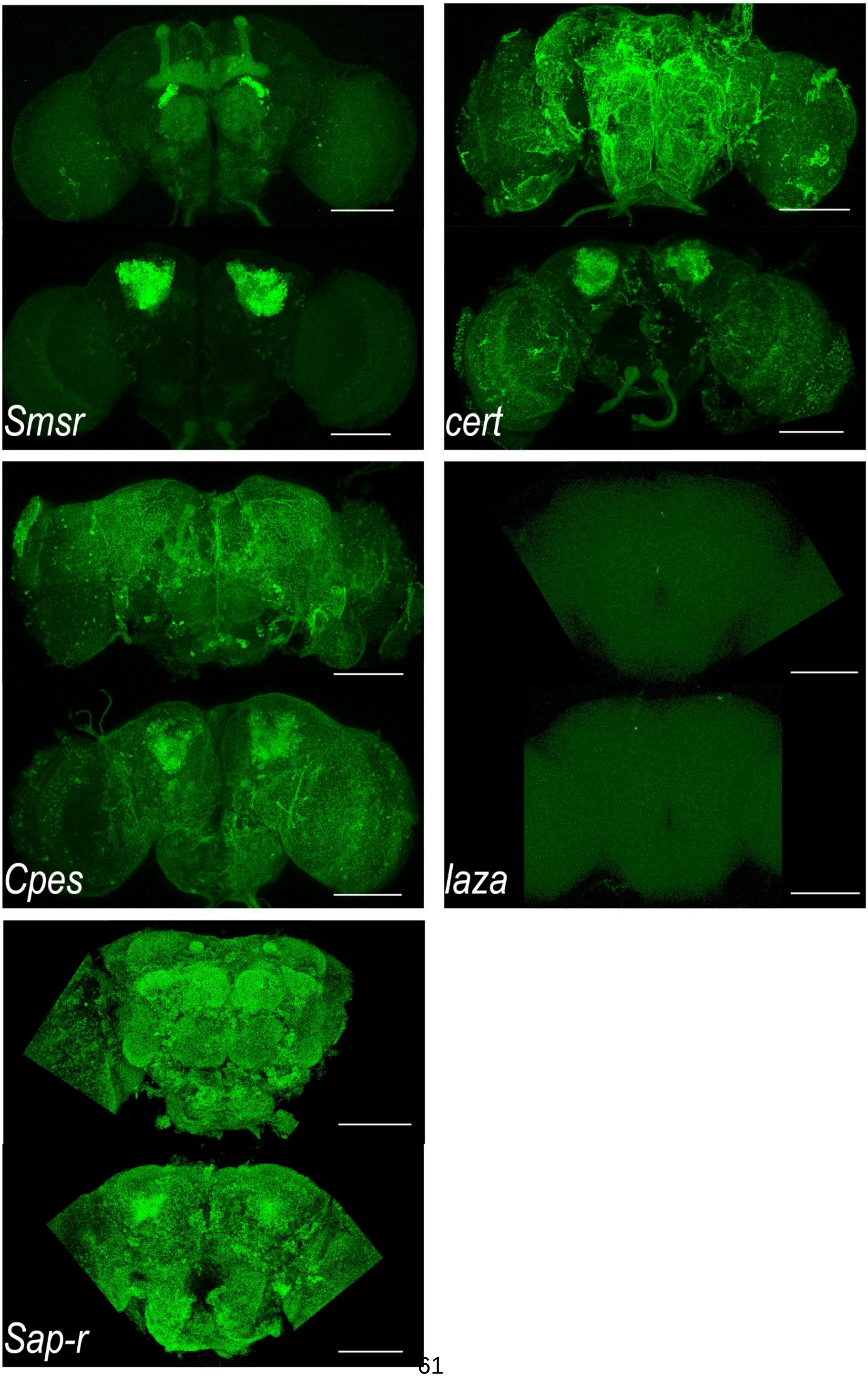

Appendix Figure S30 mCD8GFP expression driven by HG lines
Expression patterns of SPL regulators in adult brains were observed with mCD8GFP driven by HG. The top is the anterior, and the bottom is the posterior view of max projections of z-stack confocal images. The targeted gene is indicated at the bottom left. Scale bar: 100 µm.

Appendix Fig. S31

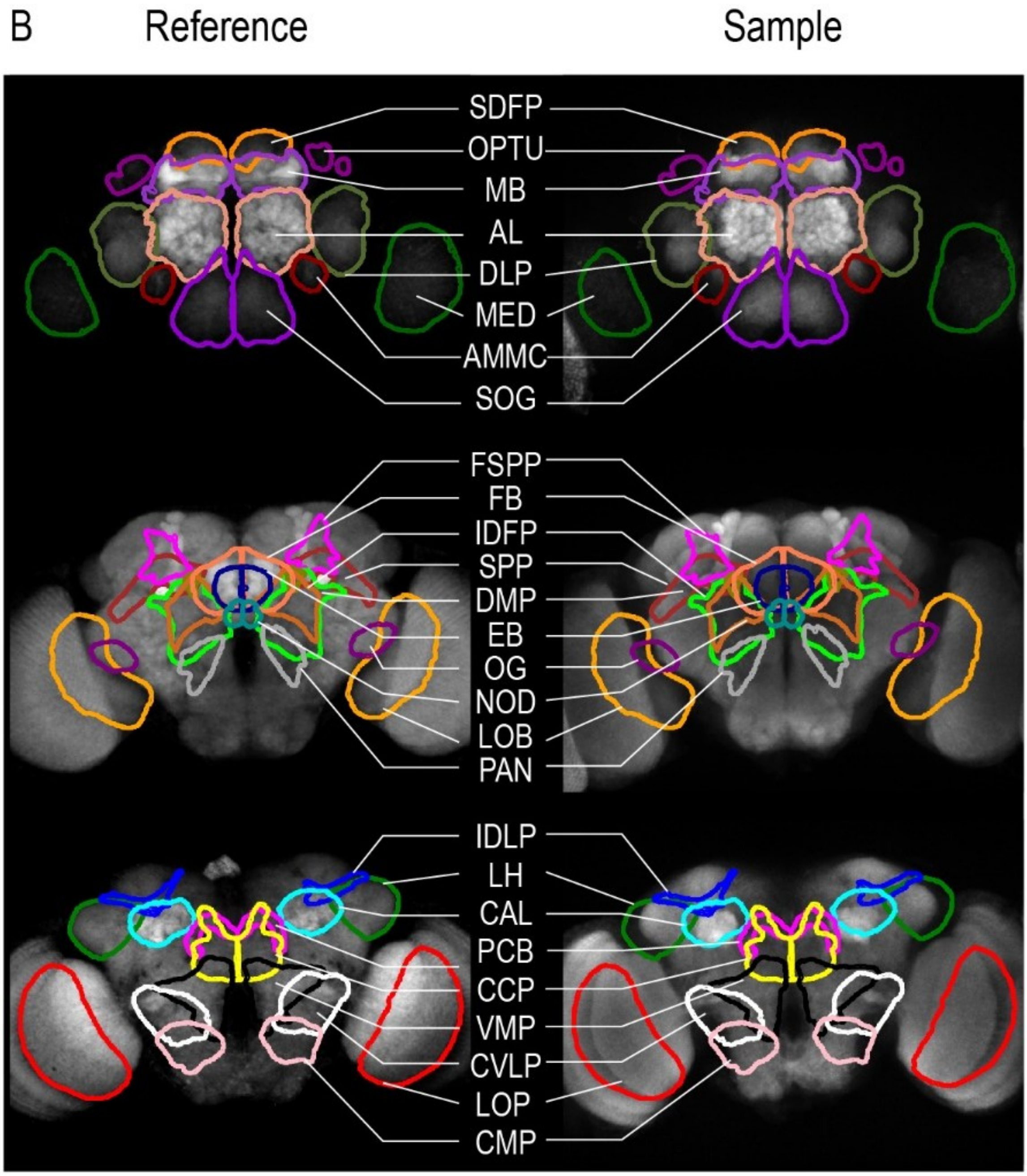

Appendix Figure S31 Brain warping representative figure
Representative confocal images of neuropil alignment of the reference brain (Chiang et al. 2011) and a sample brain. The neuropil alignment is based on anti-DLG staining.
Abbreviations**:** AL, Antennal Lobe; AMMC, Antennal Mechanosensory and Motor Center; CAL, Calyx; CCP, Caudalcentral Protocerebrum; CMP Caudalmedial Protocerebrum; CVLP, Caudal Ventrolateral Protocerebrum; DLP, Dorsolateral Protocerebrum; DMP, Dorsomedial Protocerebrum; EB, Ellipsoid Body; FB, Fanshaped Body; FSPP, Frontal Superpeduncular Protocerebrum; IDFP, Inferior Dorsofrontal Protocerebrum; IDLP, Inner Dorsolateral Protocerebrum; LAT, Lateral Triangle; LH, Lateral Horn; LOB, Lobula; LOP, Lobula Plate; MB, Mushroom body; MED, Medulla; NOD, Noduli; OG, Optic Glomerulus; OPTU, Optic Tubercle; PAN, Proximal Antennal Protocerebrum; PCB, Protocerebral Bridge; SDFP, Superior Dorsofrontal Protocerebrum; SOG, Subesophageal Ganglion; SPP, Superpeduncular Protocerebrum; VLP Ventrolateral Protocerebrum; VMP, Ventromedial Protocerebrum.

Appendix Fig. S32

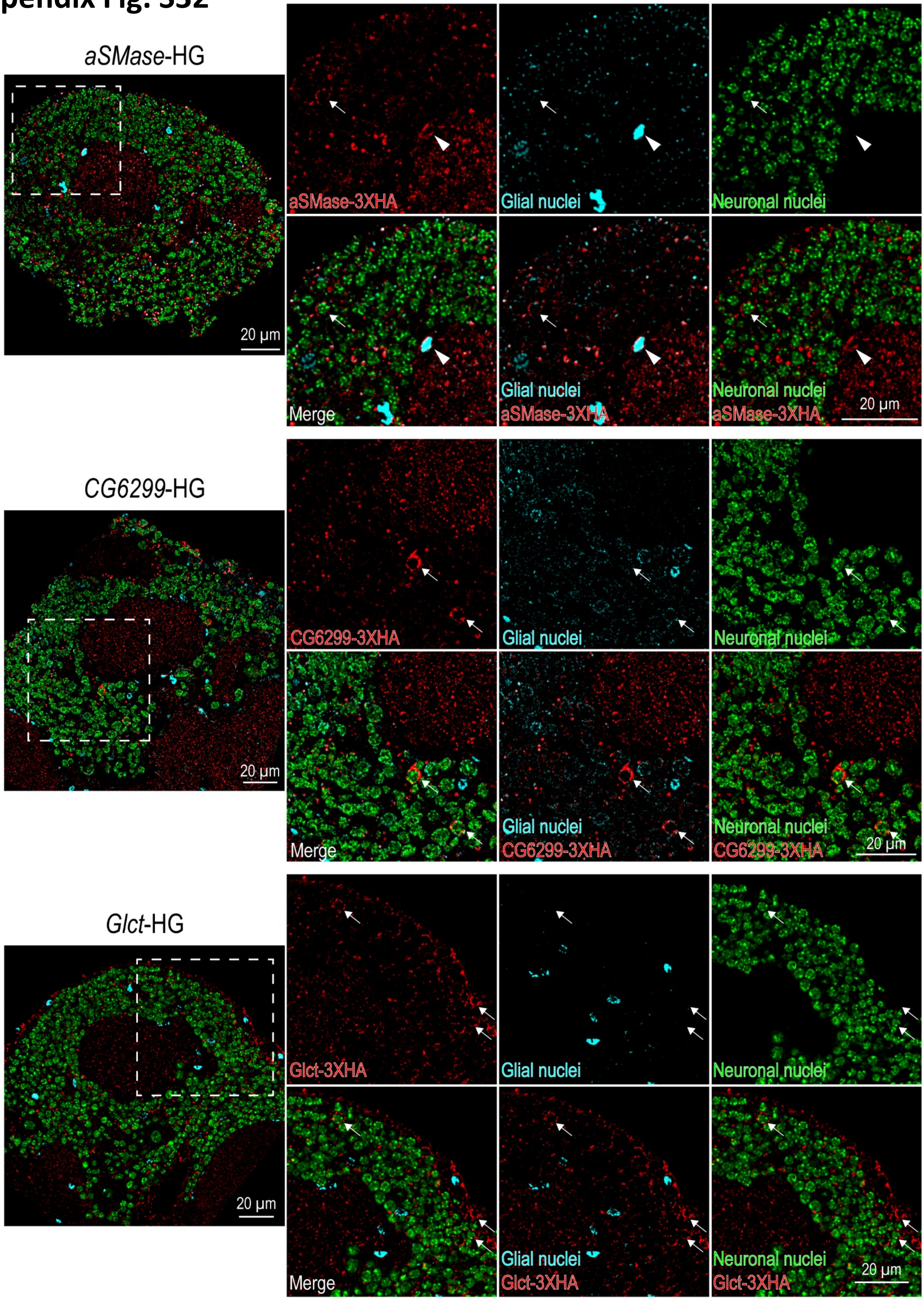

Appendix Figure S32 Anti-HA immunostainings of adult brains from HG lines

The protein distribution of 3XHA-tagged proteins is visualized by anti-HA immunostaining (red) with co-stainings of neuronal (green; anti-Elav) and glial (cyan; anti-Repo) nuclei of young adult brains (1-week-old). (Left) The representative image is taken from the posterior view of the Calyx, and the dashed-line square indicates the region of zoom-in images shown on the right.

Appendix Fig. S33

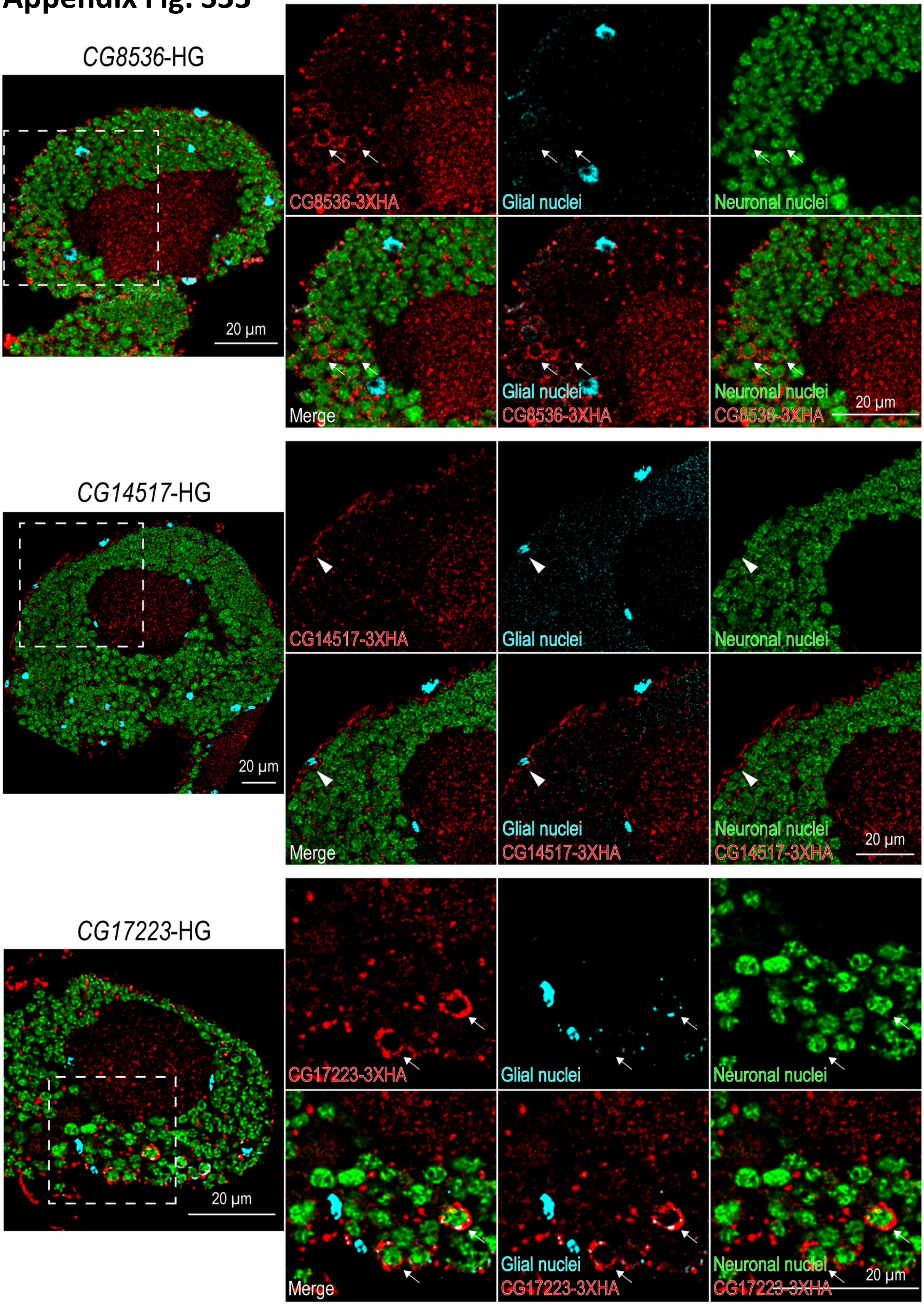

Appendix Figure S33 Anti-HA immunostainings of adult brains from HG lines

The protein distribution of 3XHA-tagged proteins is visualized by anti-HA immunostaining (red) with co-stainings of neuronal (green; anti-Elav) and glial (cyan; anti-Repo) nuclei of young adult brains (1-week-old). (Left) The representative image is taken from the posterior view of the Calyx, and the dashed-line square indicates the region of zoom-in images shown on the right.

Appendix Fig. S34

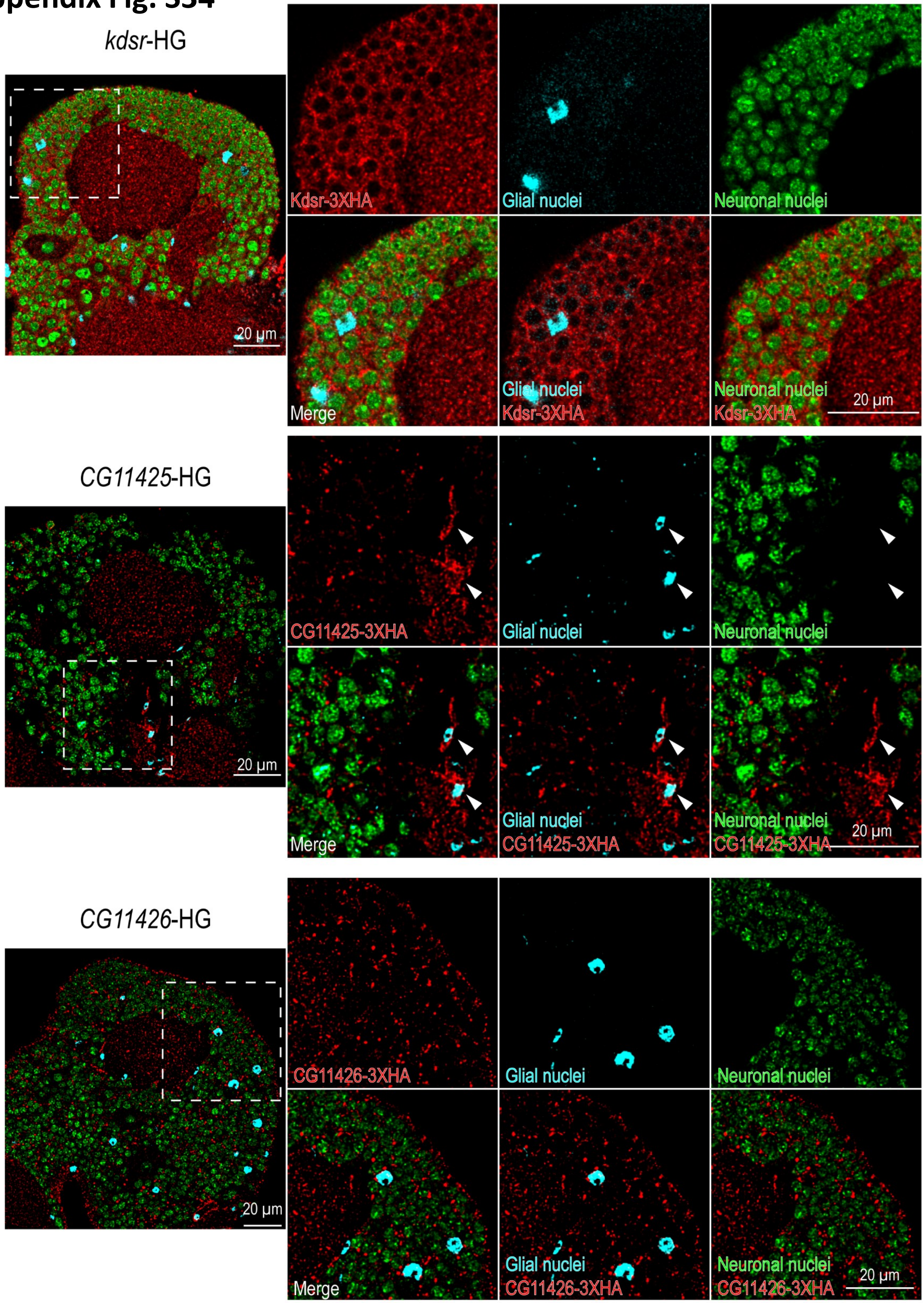

Appendix Figure S34 Anti-HA immunostainings of adult brains from HG lines

The protein distribution of 3XHA-tagged proteins is visualized by anti-HA immunostaining (red) with co-stainings of neuronal (green; anti-Elav) and glial (cyan; anti-Repo) nuclei of young adult brains (1-week-old). (Left) The representative image is taken from the posterior view of the Calyx, and the dashed-line square indicates the region of zoom-in images shown on the right.

Appendix Fig. S35

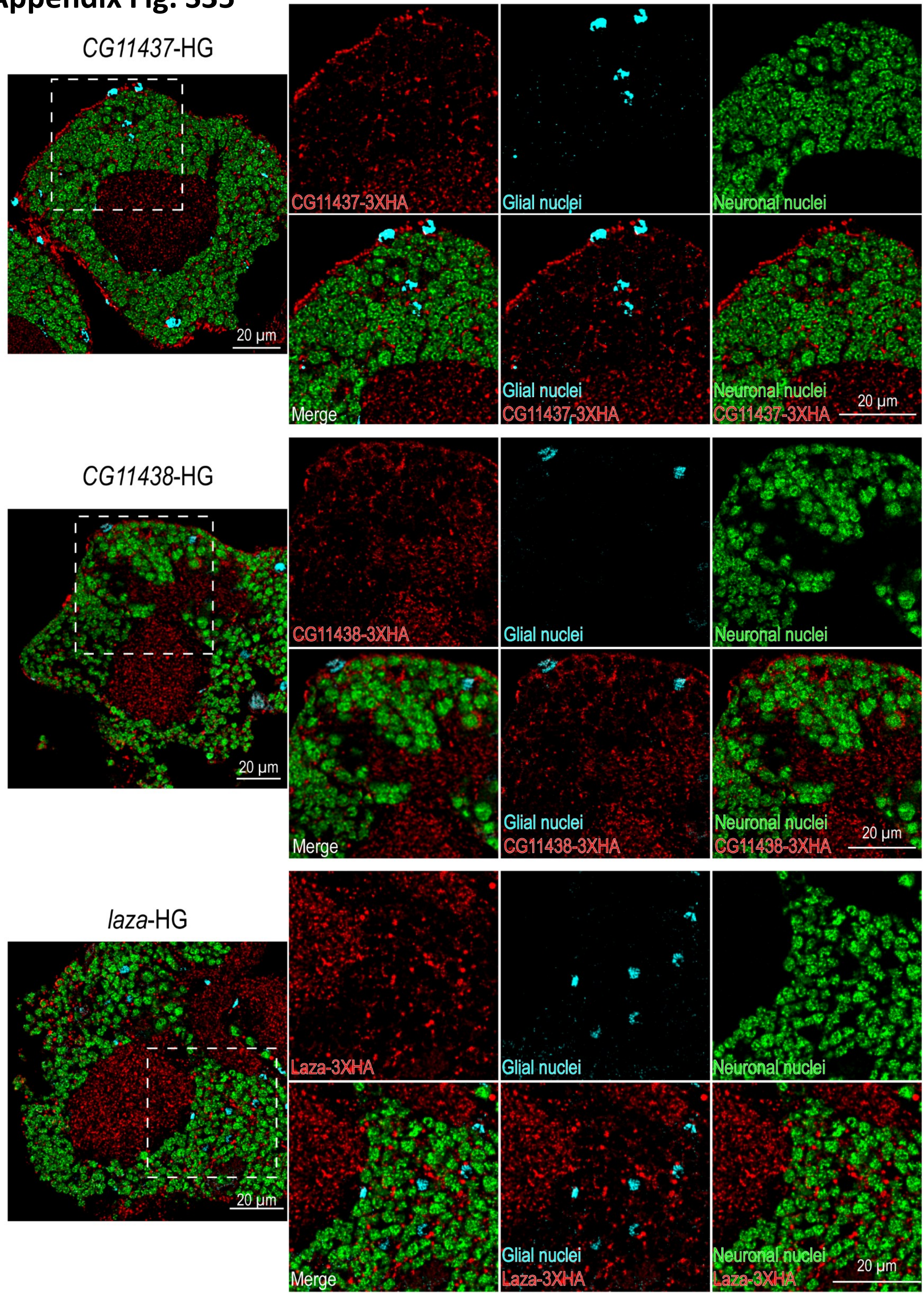

Appendix Figure S35 Anti-HA immunostainings of adult brains from HG lines

The protein distribution of 3XHA-tagged proteins is visualized by anti-HA immunostaining (red) with co-stainings of neuronal (green; anti-Elav) and glial (cyan; anti-Repo) nuclei of young adult brains (1-week-old). (Left) The representative image is taken from the posterior view of the Calyx, and the dashed-line square indicates the region of zoom-in images shown on the right.

Appendix Fig. S36

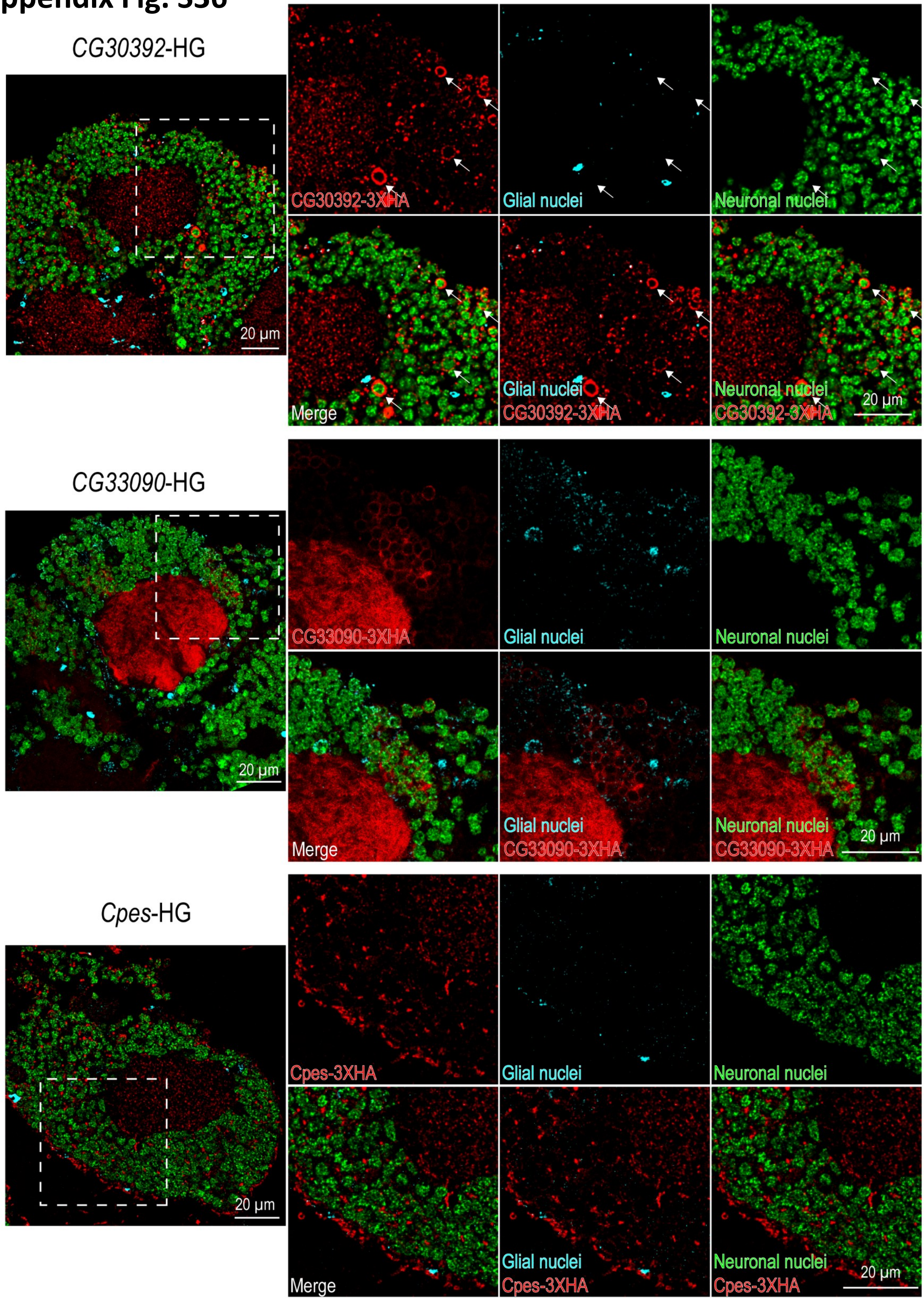

Appendix Figure S36 Anti-HA immunostainings of adult brains from HG lines

The protein distribution of 3XHA-tagged proteins is visualized by anti-HA immunostaining (red) with co-stainings of neuronal (green; anti-Elav) and glial (cyan; anti-Repo) nuclei of young adult brains (1-week-old). (Left) The representative image is taken from the posterior view of the Calyx, and the dashed-line square indicates the region of zoom-in images shown on the right.

Appendix Fig. S37

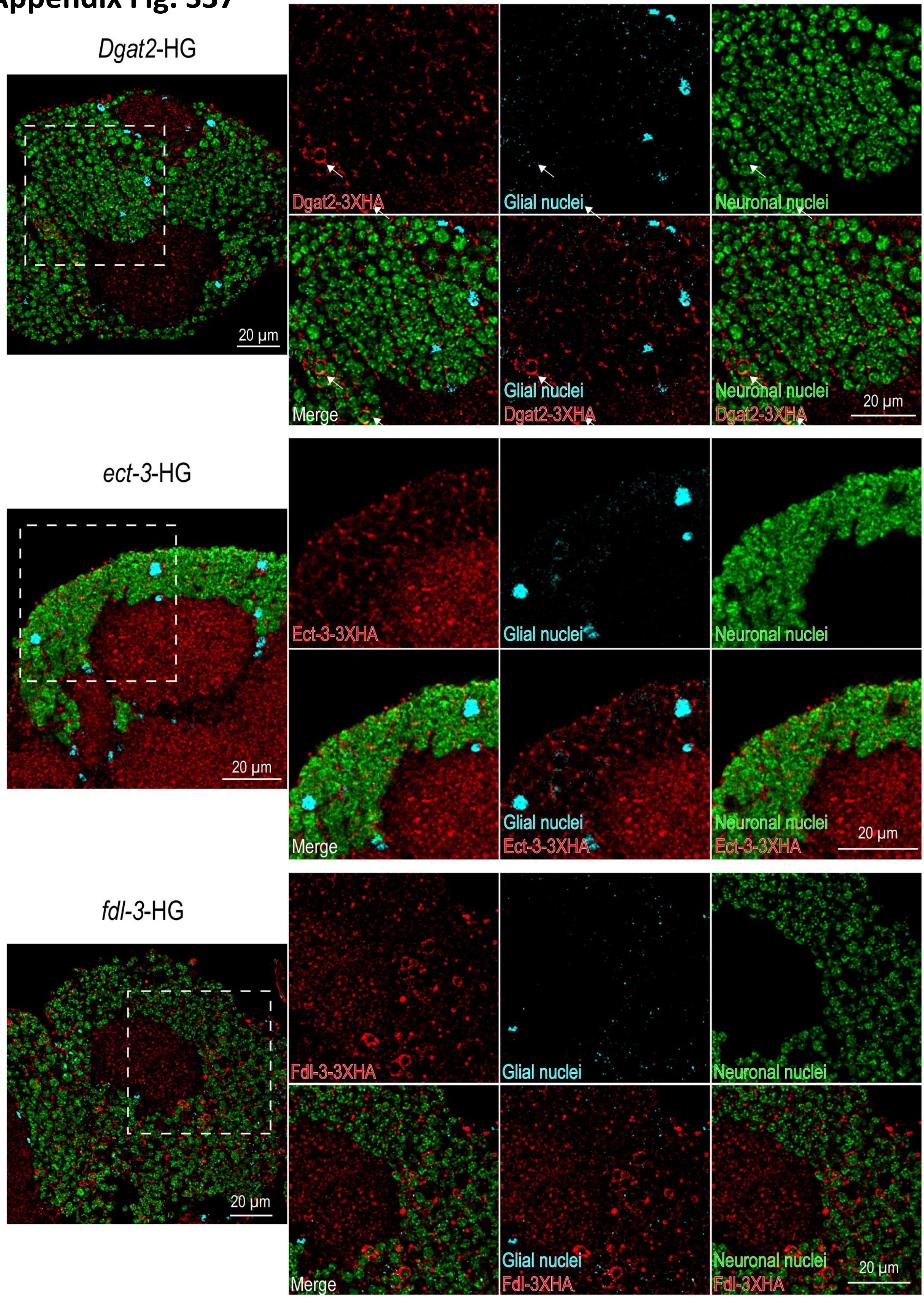

Appendix Figure S37 Anti-HA immunostainings of adult brains from HG lines

The protein distribution of 3XHA-tagged proteins is visualized by anti-HA immunostaining (red) with co-stainings of neuronal (green; anti-Elav) and glial (cyan; anti-Repo) nuclei of young adult brains (1-week-old). (Left) The representative image is taken from the posterior view of the Calyx, and the dashed-line square indicates the region of zoom-in images shown on the right.

Appendix Fig. S38

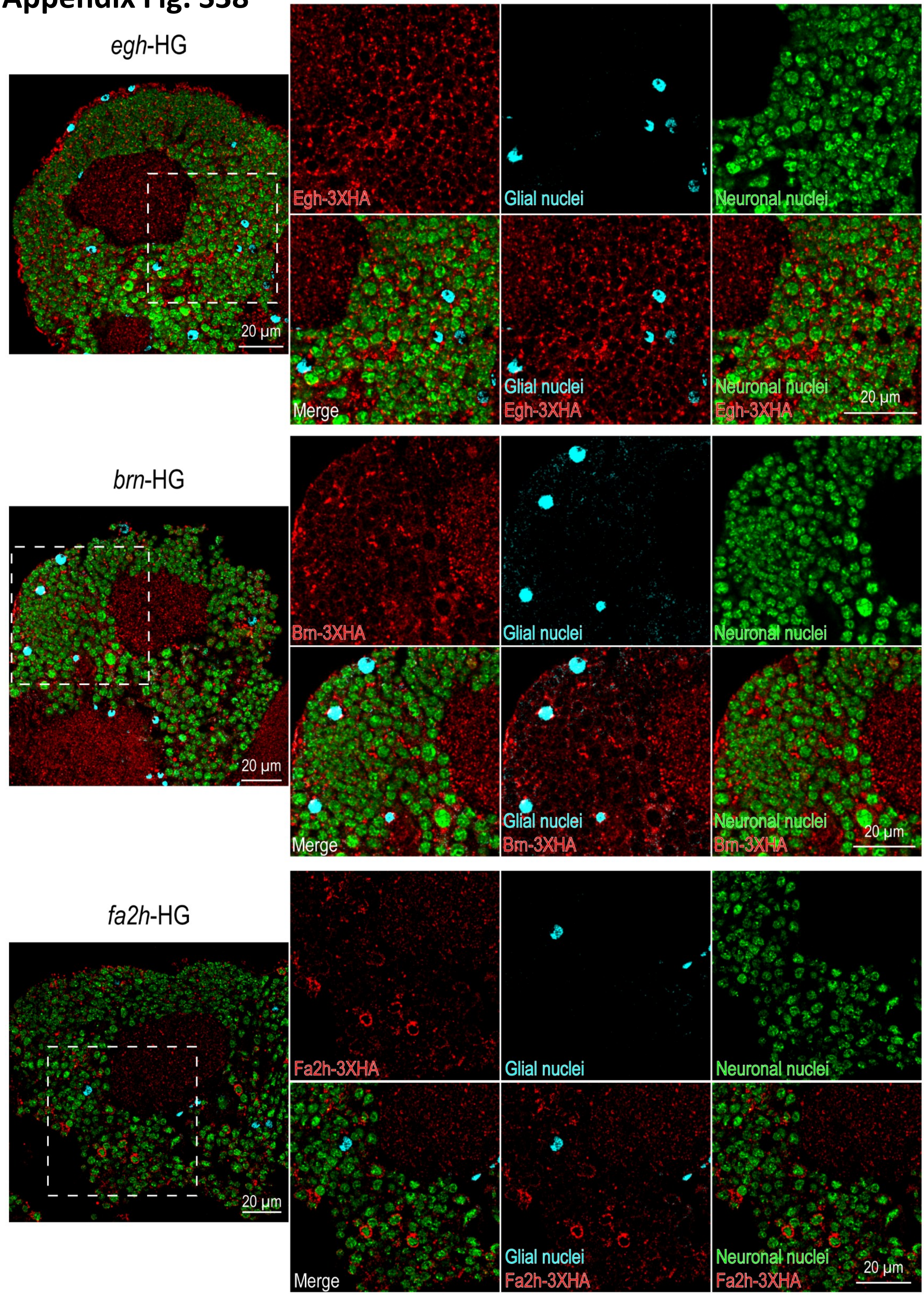

Appendix Figure S38 Anti-HA immunostainings of adult brains from HG lines

The protein distribution of 3XHA-tagged proteins is visualized by anti-HA immunostaining (red) with co-stainings of neuronal (green; anti-Elav) and glial (cyan; anti-Repo) nuclei of young adult brains (1-week-old). (Left) The representative image is taken from the posterior view of the Calyx, and the dashed-line square indicates the region of zoom-in images shown on the right.

Appendix Fig. S39

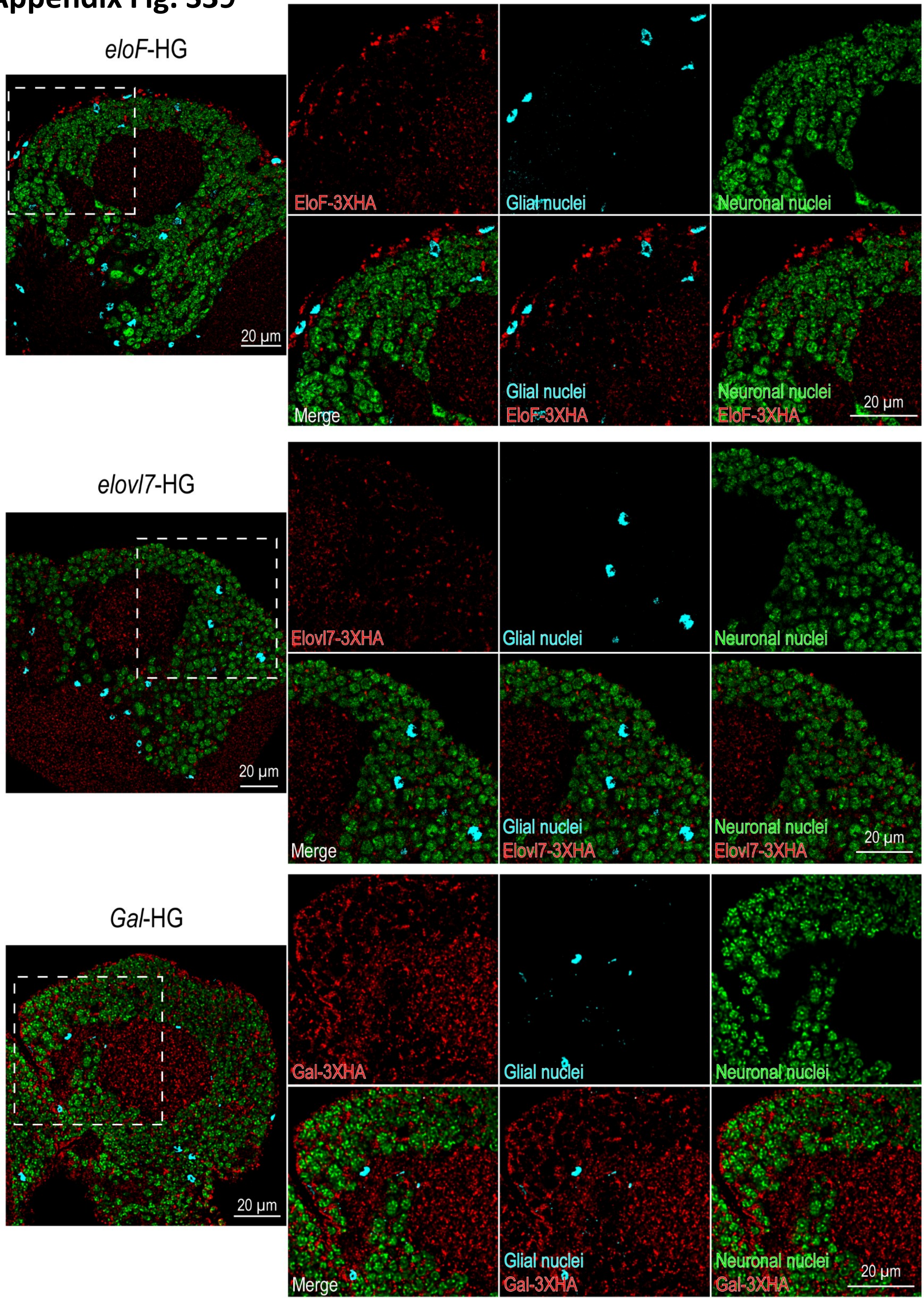

Appendix Figure S39 Anti-HA immunostainings of adult brains from HG lines

The protein distribution of 3XHA-tagged proteins is visualized by anti-HA immunostaining (red) with co-stainings of neuronal (green; anti-Elav) and glial (cyan; anti-Repo) nuclei of young adult brains (1-week-old). (Left) The representative image is taken from the posterior view of the Calyx, and the dashed-line square indicates the region of zoom-in images shown on the right.

Appendix Fig. S40

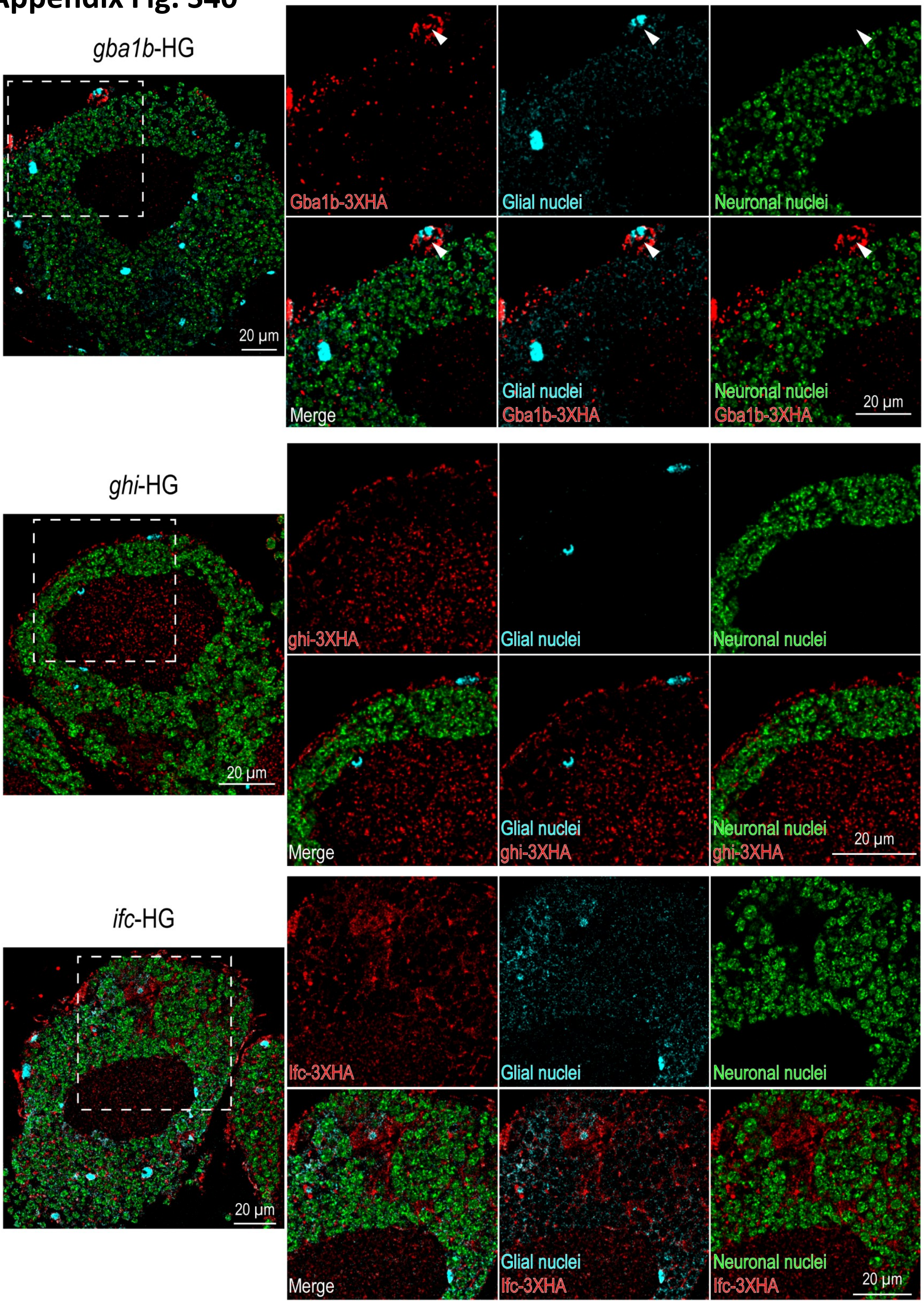

Appendix Figure S40 Anti-HA immunostainings of adult brains from HG lines

The protein distribution of 3XHA-tagged proteins is visualized by anti-HA immunostaining (red) with co-stainings of neuronal (green; anti-Elav) and glial (cyan; anti-Repo) nuclei of young adult brains (1-week-old). (Left) The representative image is taken from the posterior view of the Calyx, and the dashed-line square indicates the region of zoom-in images shown on the right.

Appendix Fig. S41

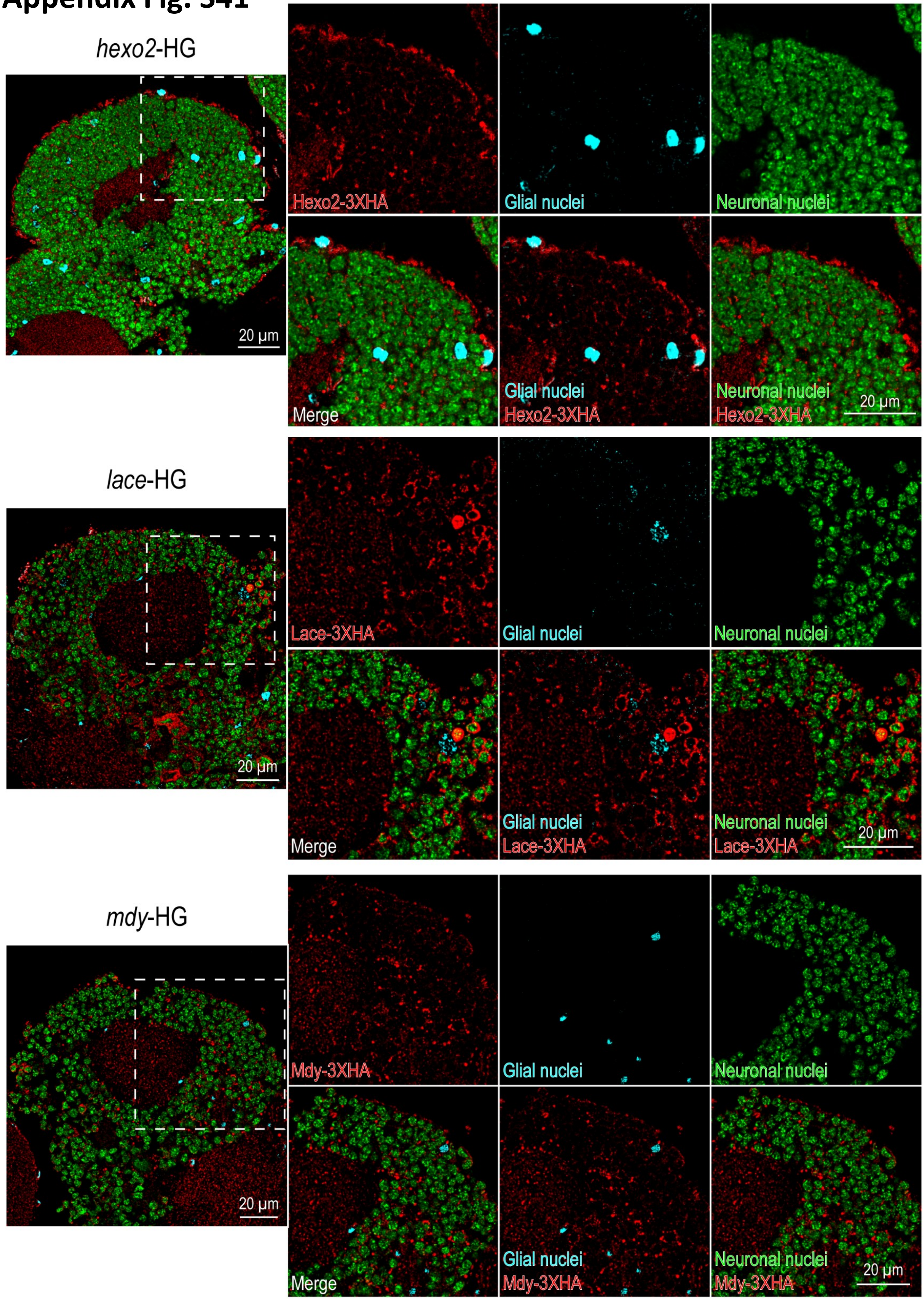

Appendix Figure S41 Anti-HA immunostainings of adult brains from HG lines

The protein distribution of 3XHA-tagged proteins is visualized by anti-HA immunostaining (red) with co-stainings of neuronal (green; anti-Elav) and glial (cyan; anti-Repo) nuclei of young adult brains (1-week-old). (Left) The representative image is taken from the posterior view of the Calyx, and the dashed-line square indicates the region of zoom-in images shown on the right.

Appendix Fig. S42

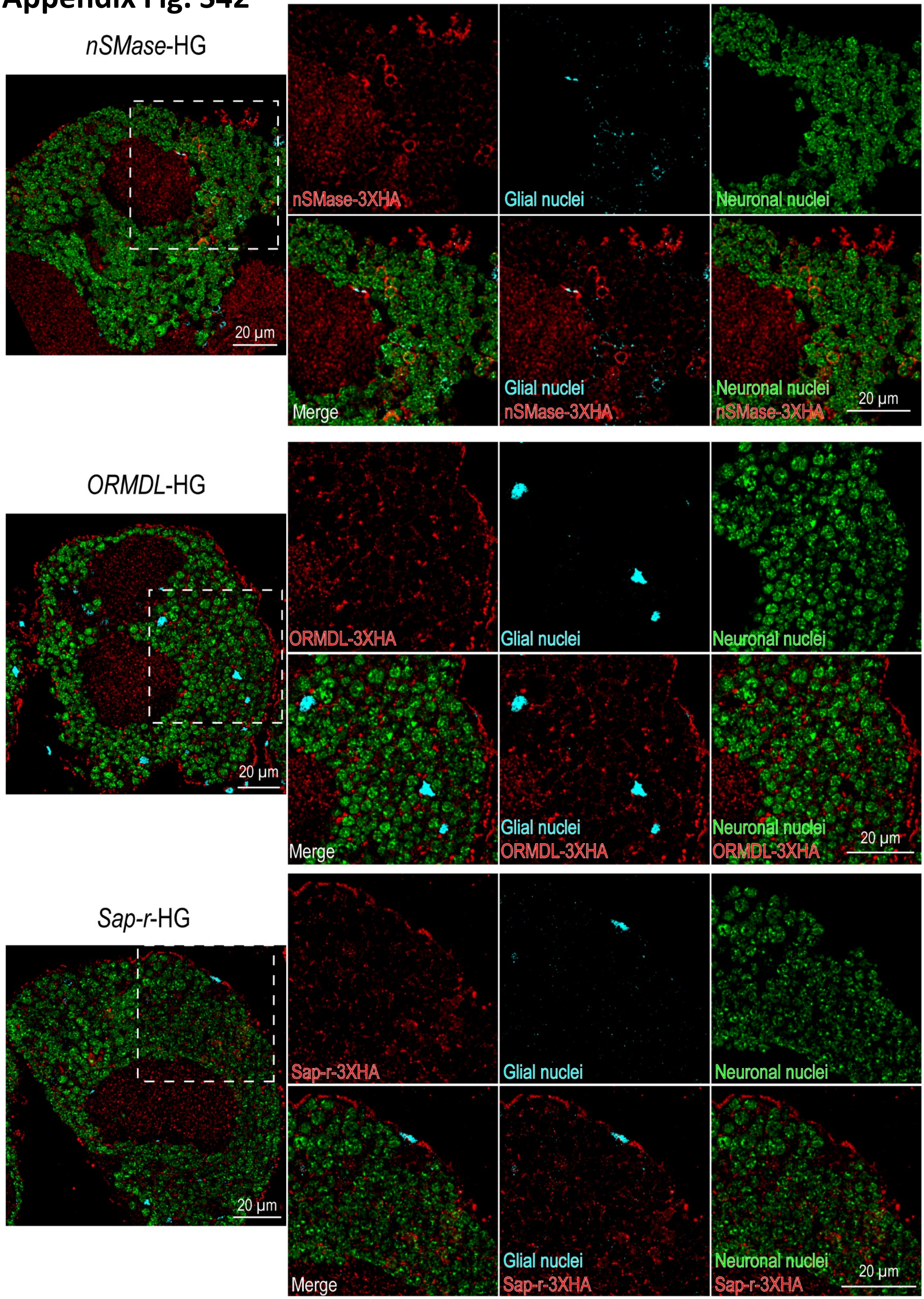

Appendix Figure S42 Anti-HA immunostainings of adult brains from HG lines

The protein distribution of 3XHA-tagged proteins is visualized by anti-HA immunostaining (red) with co-stainings of neuronal (green; anti-Elav) and glial (cyan; anti-Repo) nuclei of young adult brains (1-week-old). (Left) The representative image is taken from the posterior view of the Calyx, and the dashed-line square indicates the region of zoom-in images shown on the right.

Appendix Fig. S43

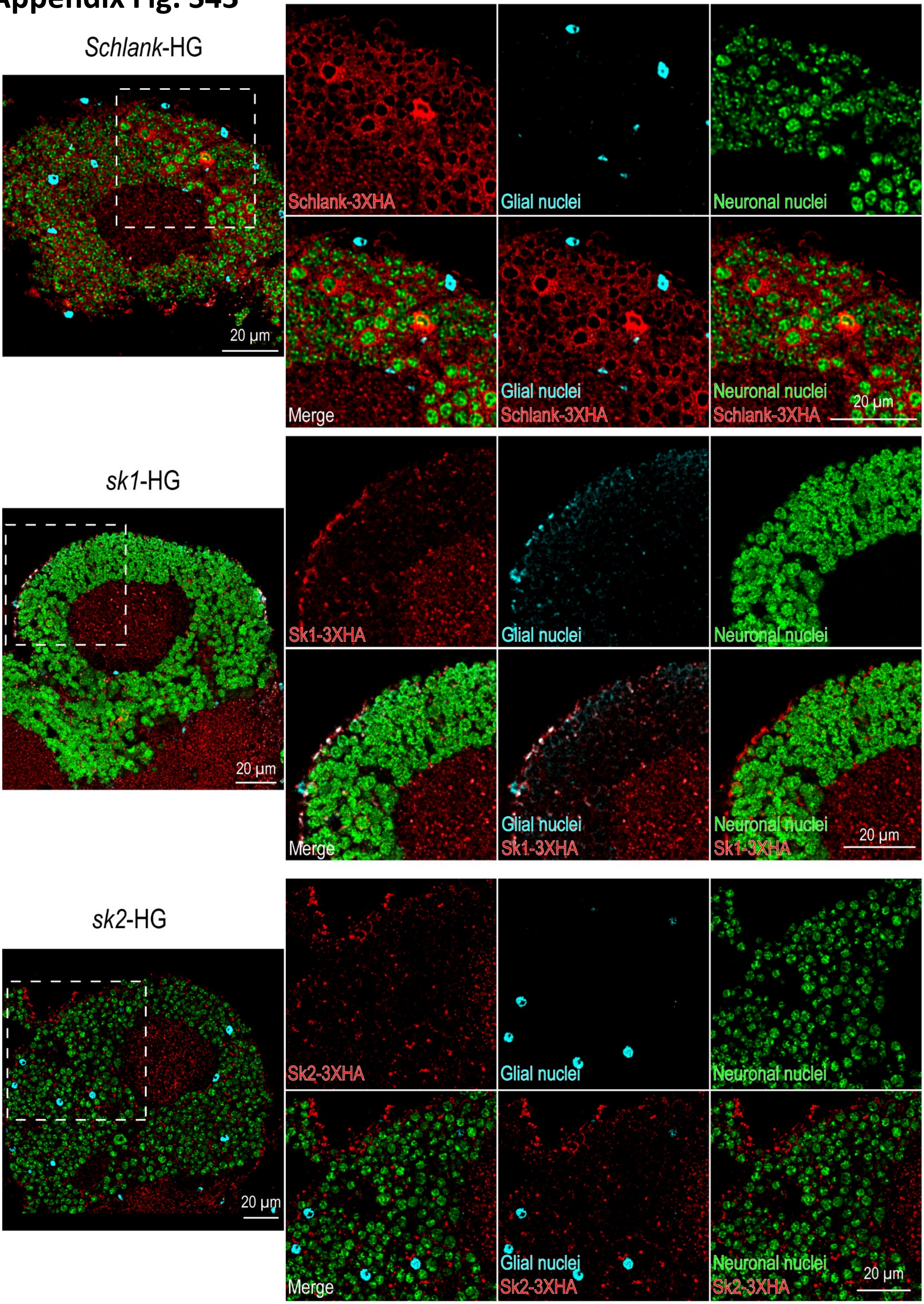

Appendix Figure S43 Anti-HA immunostainings of adult brains from HG lines

The protein distribution of 3XHA-tagged proteins is visualized by anti-HA immunostaining (red) with co-stainings of neuronal (green; anti-Elav) and glial (cyan; anti-Repo) nuclei of young adult brains (1-week-old). (Left) The representative image is taken from the posterior view of the Calyx, and the dashed-line square indicates the region of zoom-in images shown on the right.

Appendix Fig. S44

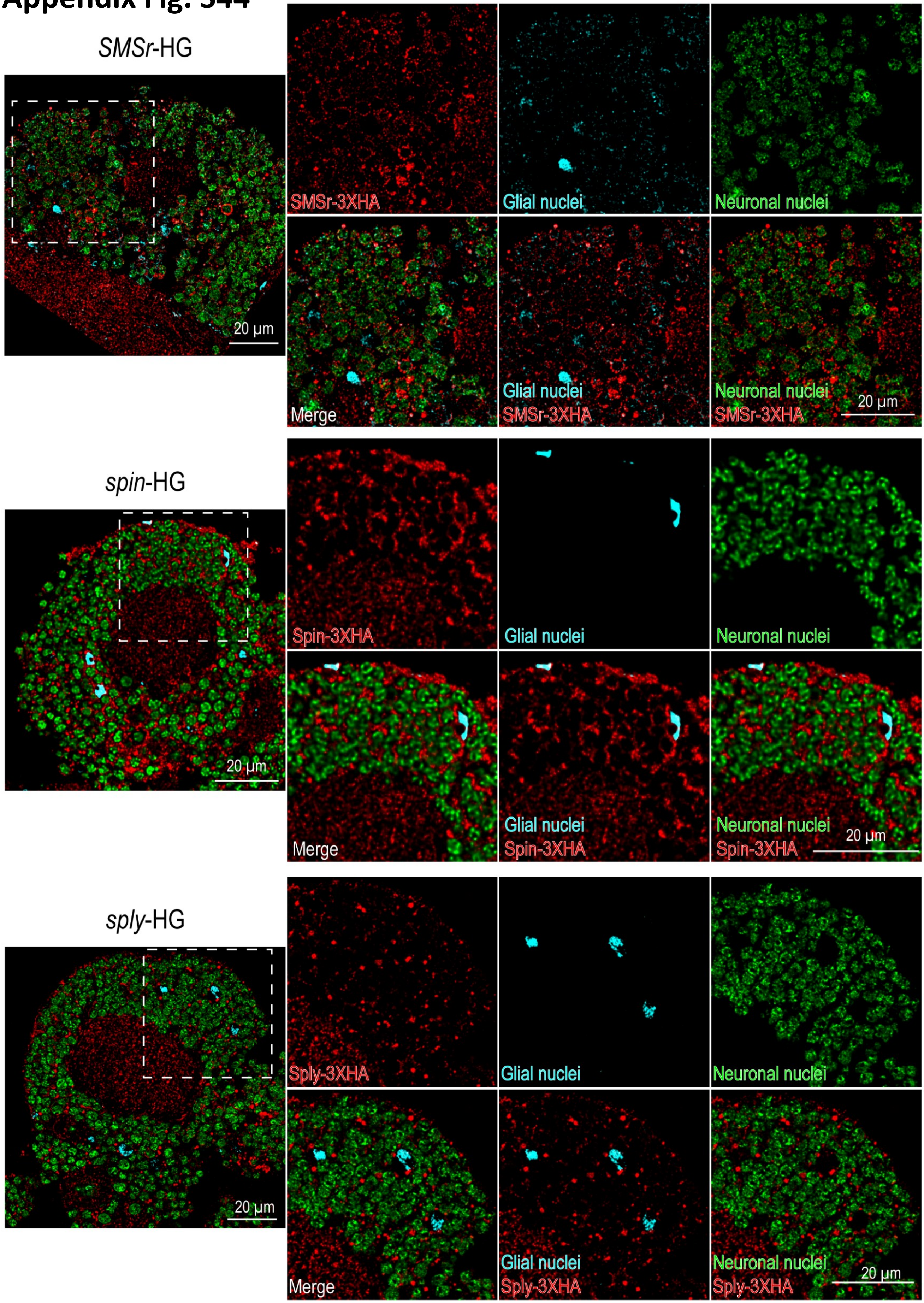

Appendix Figure S44 Anti-HA immunostainings of adult brains from HG lines

The protein distribution of 3XHA-tagged proteins is visualized by anti-HA immunostaining (red) with co-stainings of neuronal (green; anti-Elav) and glial (cyan; anti-Repo) nuclei of young adult brains (1-week-old). (Left) The representative image is taken from the posterior view of the Calyx, and the dashed-line square indicates the region of zoom-in images shown on the right.

Appendix Fig. S45

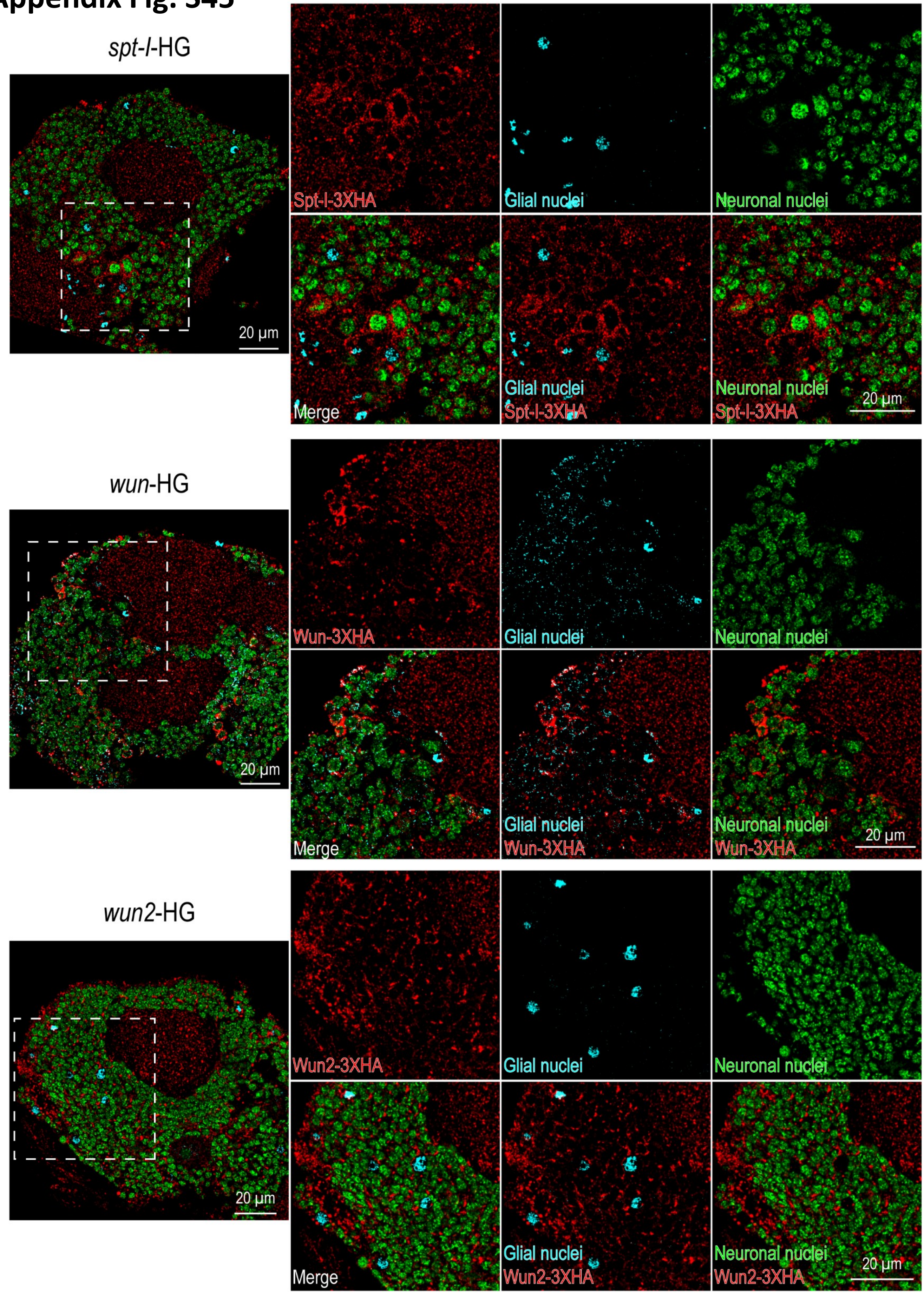

Appendix Figure S45 Anti-HA immunostainings of adult brains from HG lines

The protein distribution of 3XHA-tagged proteins is visualized by anti-HA immunostaining (red) with co-stainings of neuronal (green; anti-Elav) and glial (cyan; anti-Repo) nuclei of young adult brains (1-week-old). (Left) The representative image is taken from the posterior view of the Calyx, and the dashed-line square indicates the region of zoom-in images shown on the right.

Appendix Fig. S46

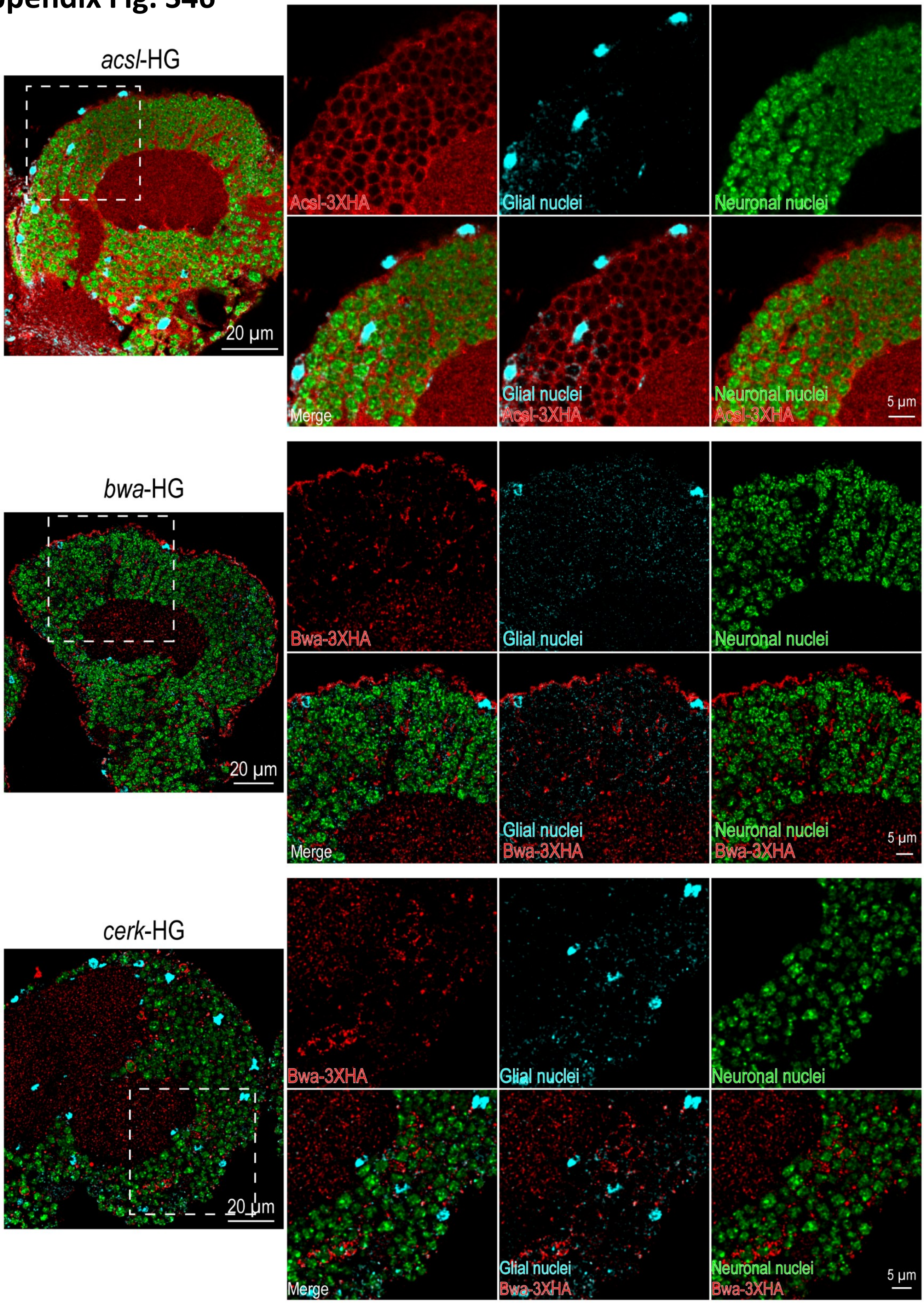

Appendix Figure S46 Anti-HA immunostainings of adult brains from HG lines

The protein distribution of 3XHA-tagged proteins is visualized by anti-HA immunostaining (red) with co-stainings of neuronal (green; anti-Elav) and glial (cyan; anti-Repo) nuclei of young adult brains (1-week-old). (Left) The representative image is taken from the posterior view of the Calyx, and the dashed-line square indicates the region of zoom-in images shown on the right.

Appendix Fig. S47

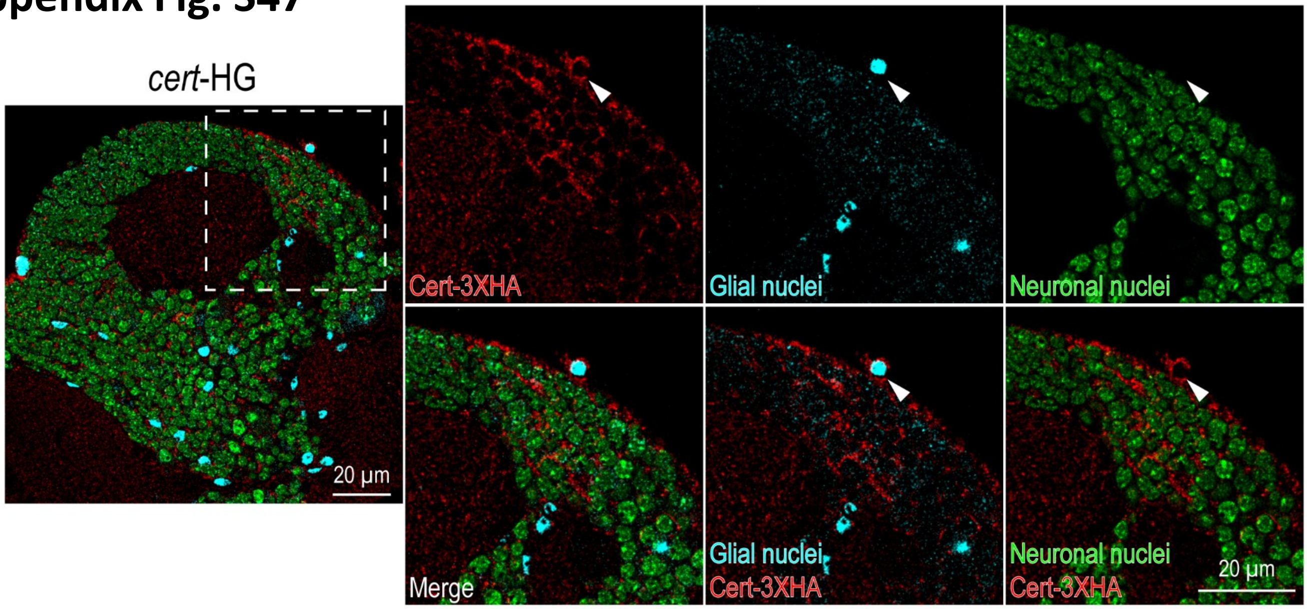

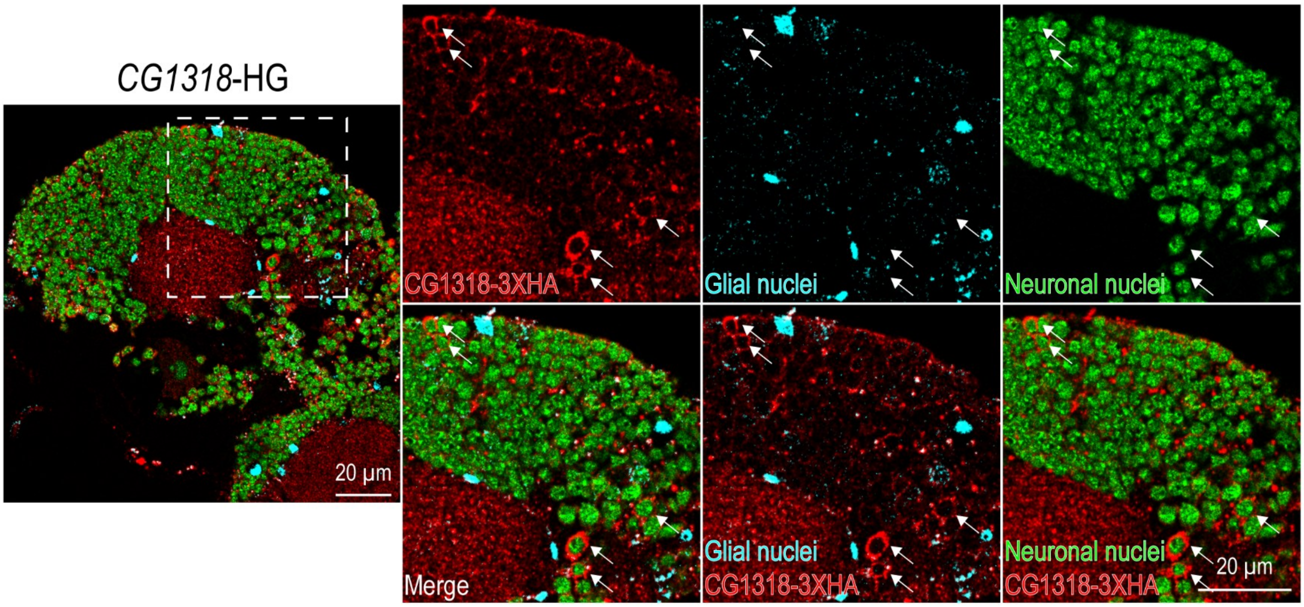

Appendix Figure S47 Anti-HA immunostainings of adult brains from HG lines

The protein distribution of 3XHA-tagged proteins is visualized by anti-HA immunostaining (red) with co-stainings of neuronal (green; anti-Elav) and glial (cyan; anti-Repo) nuclei of young adult brains (1-week-old). (Left) The representative image is taken from the posterior view of the Calyx, and the dashed-line square indicates the region of zoom-in images shown on the right.

Appendix Fig. S48

# ID	Prediction	Other	Signal Peptide (Sec/SPI)	Lipoprotein signal peptide (Sec/SPII)	TAT signal peptide (Tat/SPI)	TAT Lipoprotein signal peptide (Tat/SPII)	Pilin-like signal peptide (Sec/SPIII)	CS Position
CDase-PA	Signal Peptide	0.000251	0.999172	0.000153	0.000139	0.000138	0.000129	CS pos: 23-24. Pr: 0.9785
Gba1a-PA	Signal Peptide	0.000216	0.999204	0.000153	0.000146	0.000134	0.000126	CS pos: 23-24. Pr: 0.9781
CG15533-PA	Signal Peptide	0.000247	0.999115	0.000159	0.000164	0.000158	0.000146	CS pos: 21-22. Pr: 0.9744
CG15534-PA	Lipoprotein signal peptide	0	0	1	0	0	0	CS pos: 18-19. Pr: 0.9906

CDase-PA

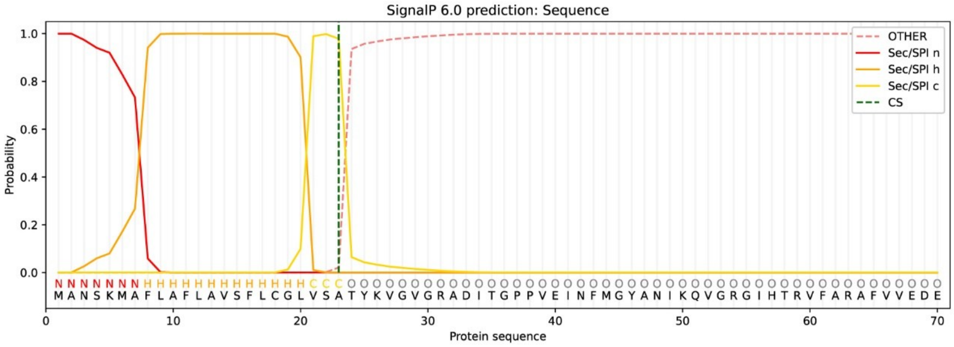

Gba1a-PA

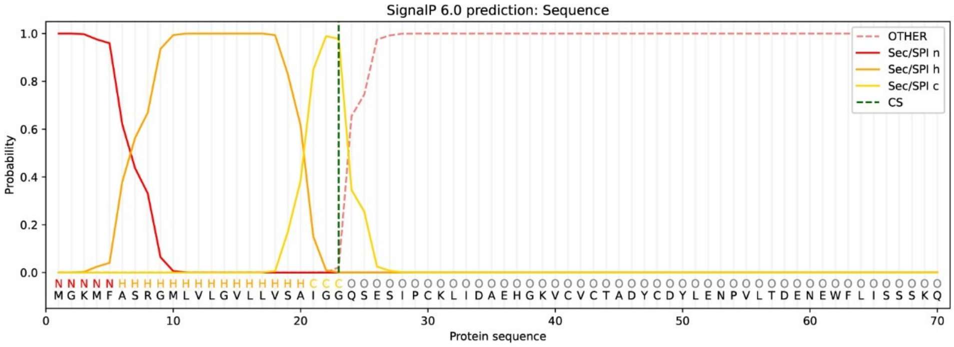

CG15533-PA

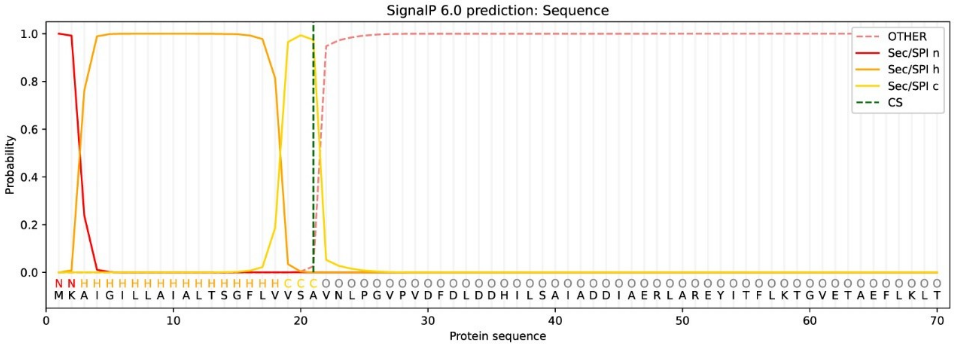

CG15534-PA

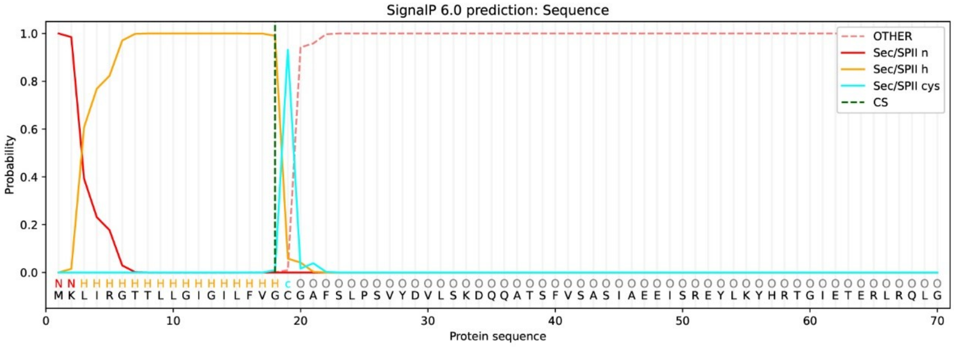

Appendix Figure S48 Prediction of signaling peptides on CDase, Gba1a, CG15533, and CG15534

(Top) Summary table of SignalP 6.0 predictions, including protein ID, predicted signal peptide presence, type, cleavage site, and probability score. (Bottom) Representative SignalP 6.0 cleavage site prediction plot.

Appendix Fig. S49

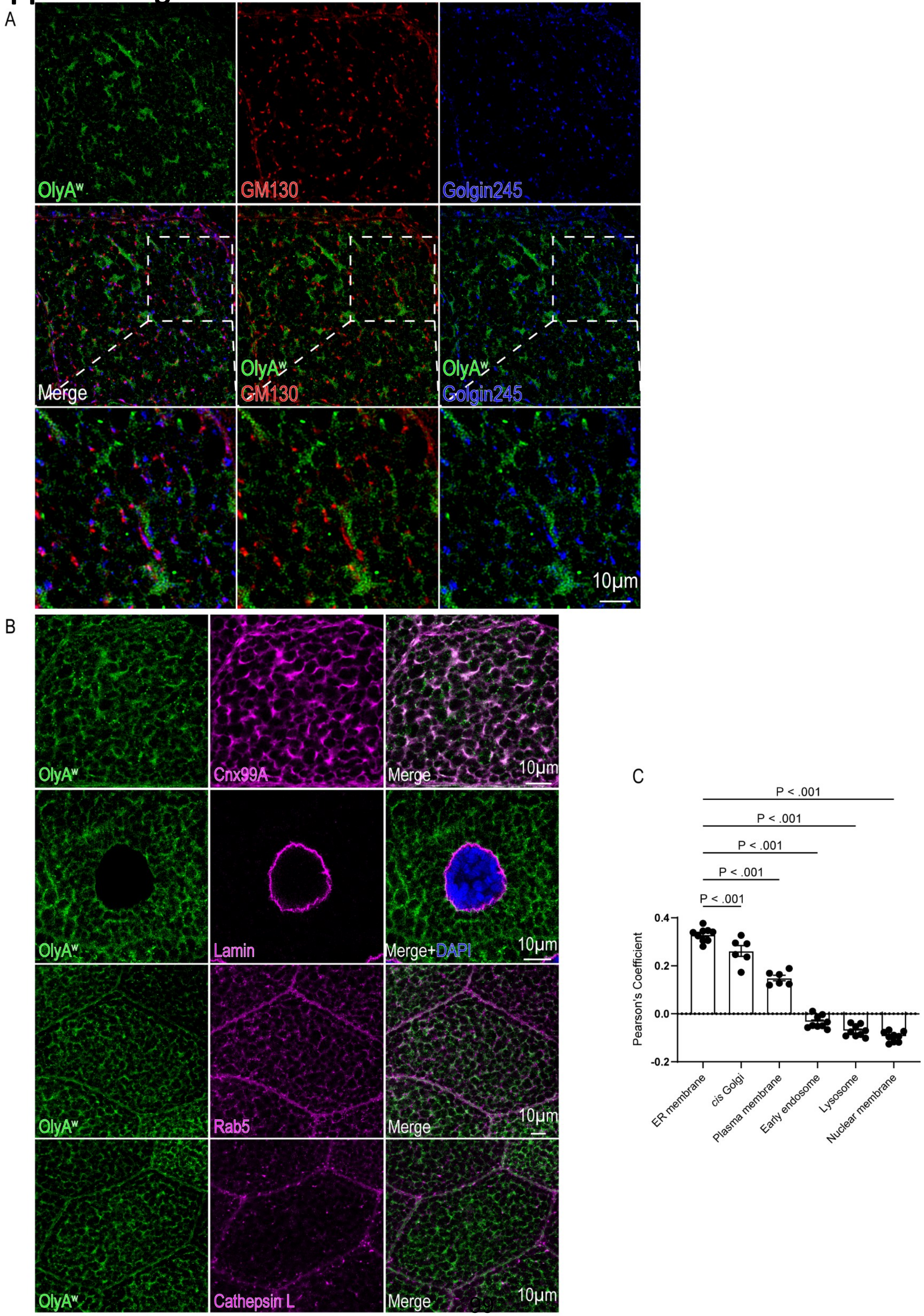

Appendix Figure S49 Subcellular localization of OlyA^{w} in L3 salivary glands

A Colocalization analysis of OlyA^{w} and Golgi markers in the L3 salivary glands, including anti-GM130 (*cis*-Golgi, red) and Golgin245 (*trans*-Golgi, blue).

B Confocal images of OlyA^{w} and stainings of organelle markers in the L3 salivary glands including anti-Cnx99A (ER), anti-Lamin (nuclear membrane), anti-Rab5 (early endosomes), and anti-Cathepsin L (lysosomes) .

C Quantifications of Pearson's coefficient of OlyA^{w} and organelle markers in the L3 salivary glands. Data are representative of at least 2 independent experiments. Data are represented as mean ± SEM (n ≥ 6). *P* values were calculated using one-way ANOVA with Dunnett's multiple comparisons.

Appendix Fig. S50

A

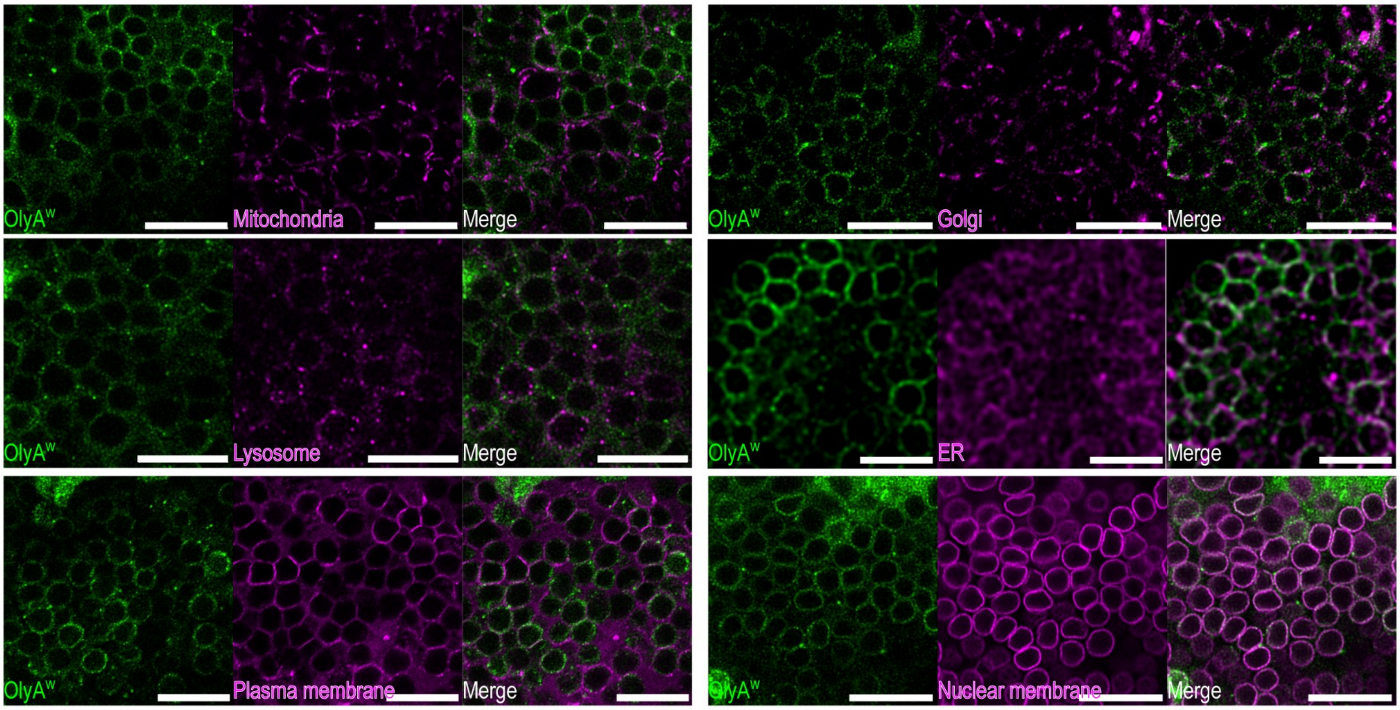

B

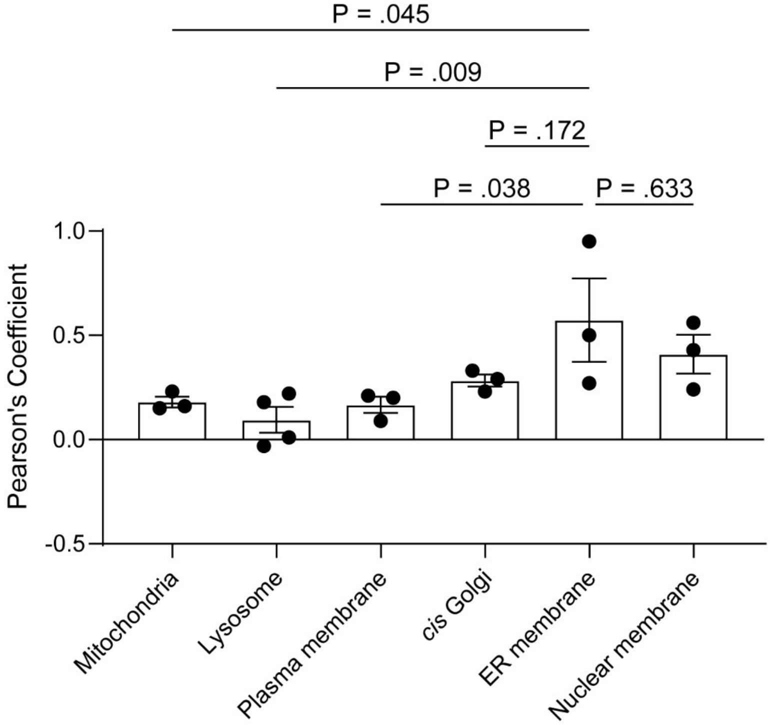

Appendix Figure S50 Subcellular localization of OlyA^{w} in adult brain

A Representative confocal images of OlyA^{w} and stainings of organelle markers including anti-ATP5A (Mitochondria), anti-GM130 (Golgi), anti-lamp1 (Lysosome), anti-Calnexin (ER), anti-Na^{+}-K^{+}-ATPase (Plasma membrane), and anti-lamin (Nuclear membrane) in the adult brains.

B Quantifications of Pearson's coefficient of OlyA^{w} and organelle markers in the adult brains. Data are representative of at least 2 independent experiments. Data are represented as mean ± SEM (n ≥ 3). *P* values were calculated using one-way ANOVA with Dunnett's multiple comparisons.

Appendix Fig. S51

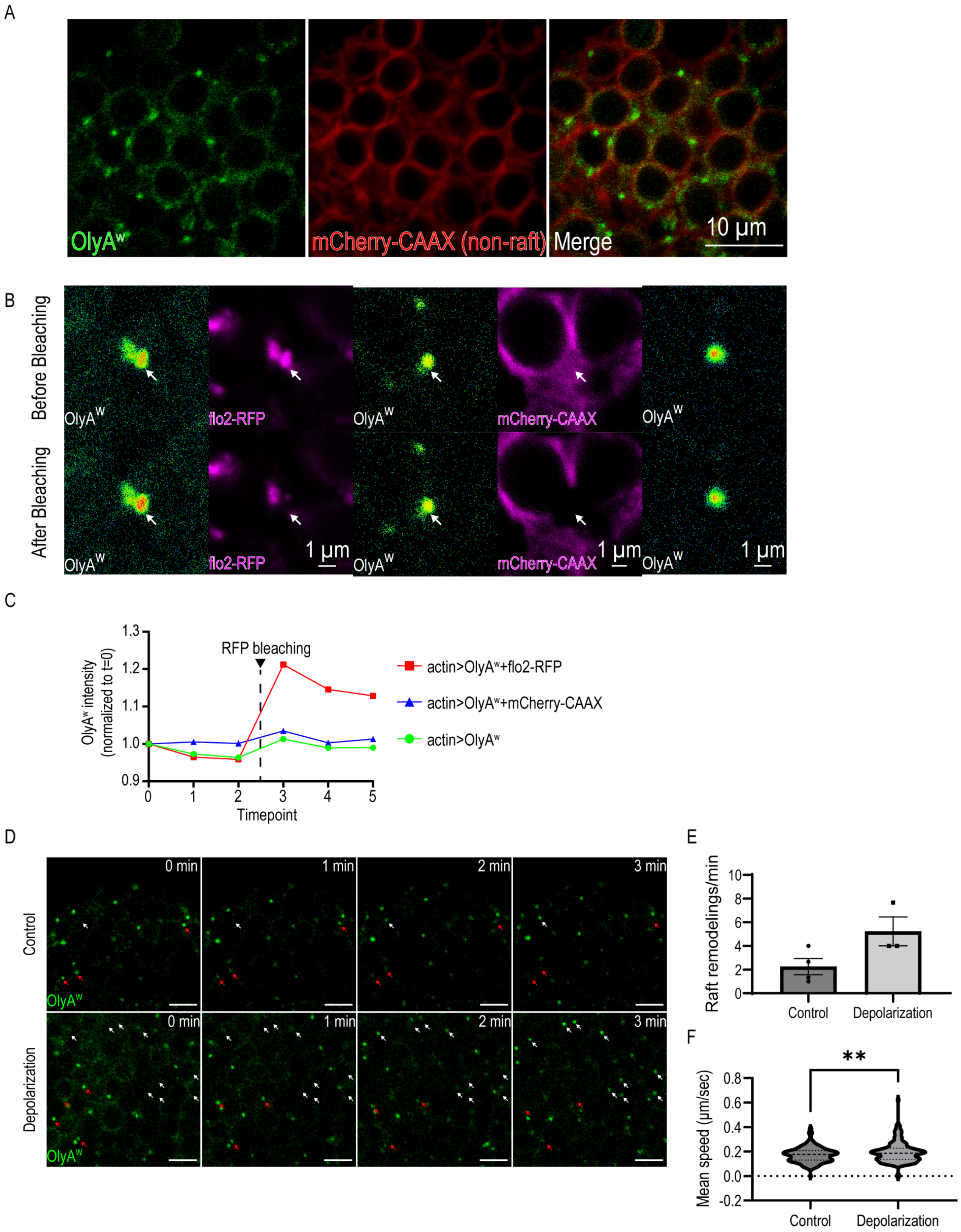

Appendix Figure S51 OlyA^w live imaging reveals lipid raft dynamic

A Confocal images of the adult brain co-overexpressing OlyA^w and a membrane marker mCherry-CAAX (*Actin*-GAL4/*UAS-mCherry-CAAX; UAS-OlyA^w /+*).

B Acceptor Photo-bleaching Förster Resonance Energy Transfer (AP-FRET) experiments of OlyA^w (color scale) with flo2-RFP (raft marker), mCherry-CAAX (non-raft marker), and the donor-only control.

C The OlyA^w intensity of each time point. The OlyA^w intensity of each group is normalized to its intensity at time t_0.

D *Ex vivo* live imaging of OlyA^w in adult hemolymph-like saline (AHL), comparing normal AHL and that with 50 mM KCl.

E Quantification of emerging and disappearing OlyAw puncta per minutes. Data are represented as mean $\pm$ SEM ($n \geq 4$).

F Quantification of the mean speed of OlyA^w tracks. Data are represented as mean $\pm$ SEM. P values (***P* < 0.01) were calculated using two-tailed unpaired Student's t-test.

Appendix Fig. S52

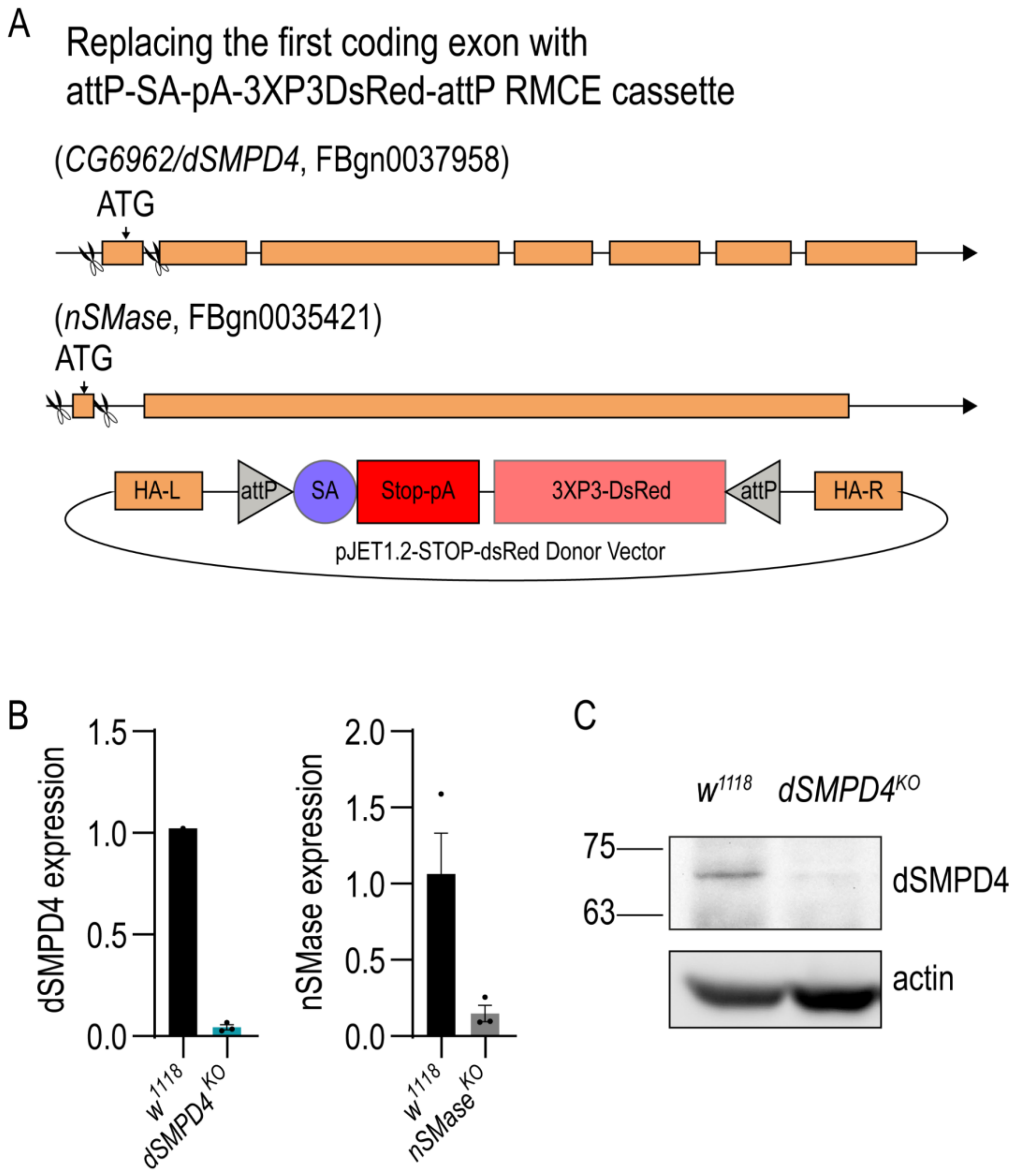

Appendix Figure S52 CRISPR-Knockout of *dSMPD4* and *nSMase*

A Schematic figure of CRISPR-mediated exon1-ATG knockout of the *dSMPD4* and *nSMase*

B RT-qPCR analysis of *dSMPD4*- and *nSMase*-knockout flies

C Western blot analysis of *dSMPD4*-knockout flies.

Appendix Fig. S53

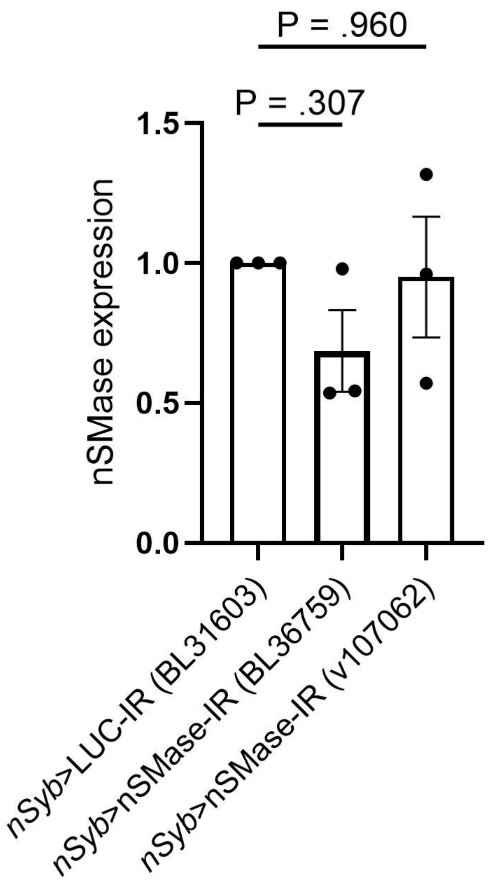

Appendix Figure S53 RT-qPCR analysis of nSMase RNAi knockdown efficiency in neurons of the adult brain

Quantification of *nSMase* expression levels in the adult head normalized to the control [nSyb-GAL4>UAS-LUC-IR (BL31603)] genotype. Data are represented as mean ± SEM. *P* values were calculated using one-way ANOVA with Dunnett's multiple comparisons.

Appendix Fig. S54

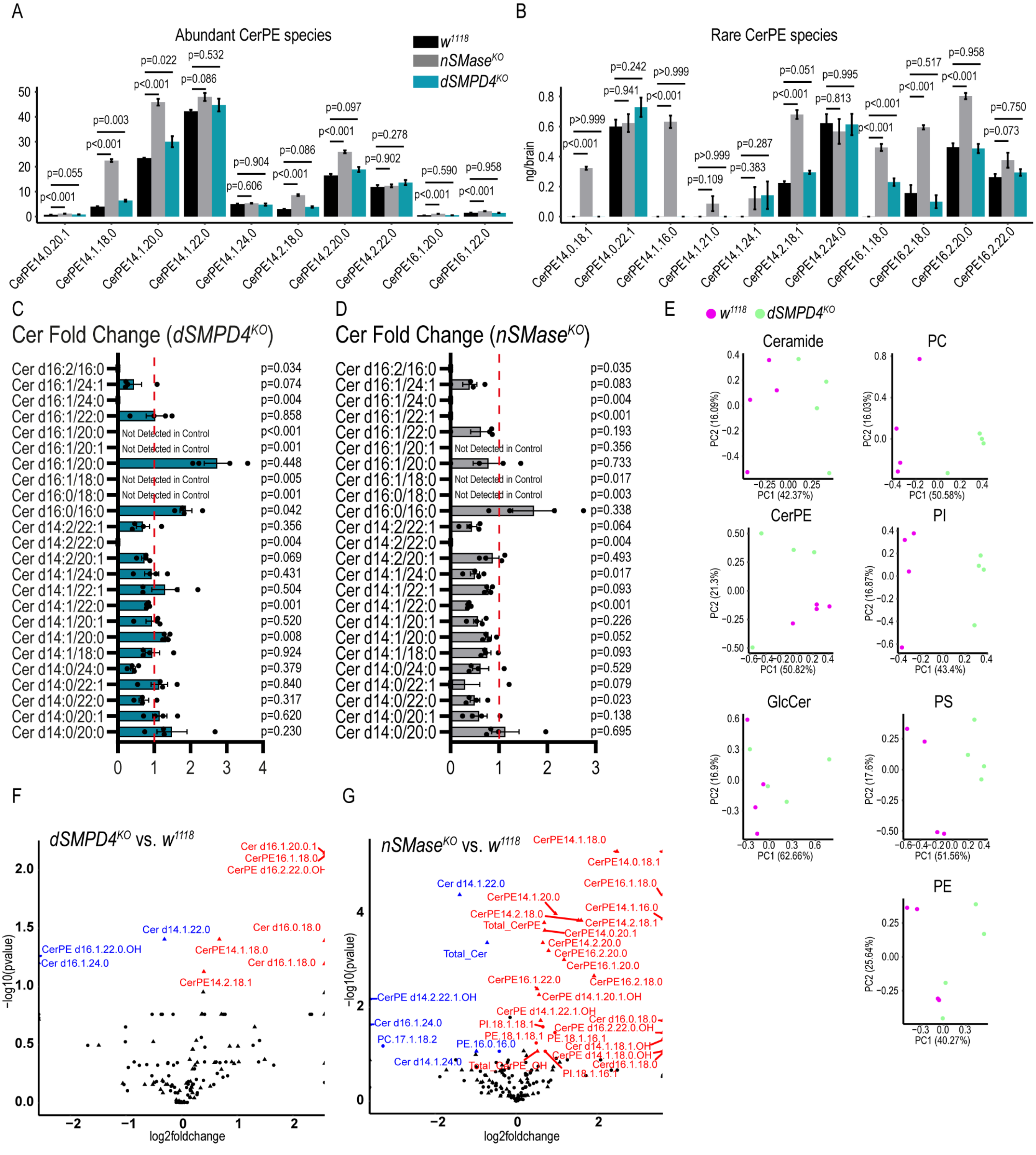

Appendix Figure S54 Lipidomic analysis of *dSMPD4*^{KO} and *nSMase*^{KO} brains

A Levels of abundant CerPE species (>1 ng/brain) in brains of *w*^{1118} control, *nSMase*^{KO}, and *dSMPD4*^{KO} flies. *P* values were calculated using one-way ANOVA with Dunnett's multiple comparisons.

B Levels of rare CerPE species (<1 ng/brain) in brains of of *w*^{1118} control, *nSMase*^{KO}, and *dSMPD4*^{KO} flies. *P* values were calculated using one-way ANOVA with Dunnett's multiple comparisons.

C Quantification of the fold change in ceramide levels of *dSMPD4*^{KO} brains compared to *w*^{1118} control. Data are represented as mean ± SEM. *P* values were calculated using two-tailed unpaired Student's t-test.

D Quantification of the fold change in ceramide levels of *nSMase*^{KO} brains compared to *w*^{1118} control. Data are represented as mean ± SEM. *P* values were calculated using two-tailed unpaired Student's t-test.

E PCA plots of phospholipids and SPLs in brains of *dSMPD4*^{KO} and *w*^{1118} control flies.

F Volcano plot showing increased and decreased lipid species in the brain of *dSMPD4*^{KO} flies. SPLs are shown as triangles, while phospholipids are circles.

G Volcano plot showing increased and decreased lipid species in the brain of *nSMase*^{KO} flies. SPLs are shown as triangles, while phospholipids are circles.

Appendix Fig. S55

A

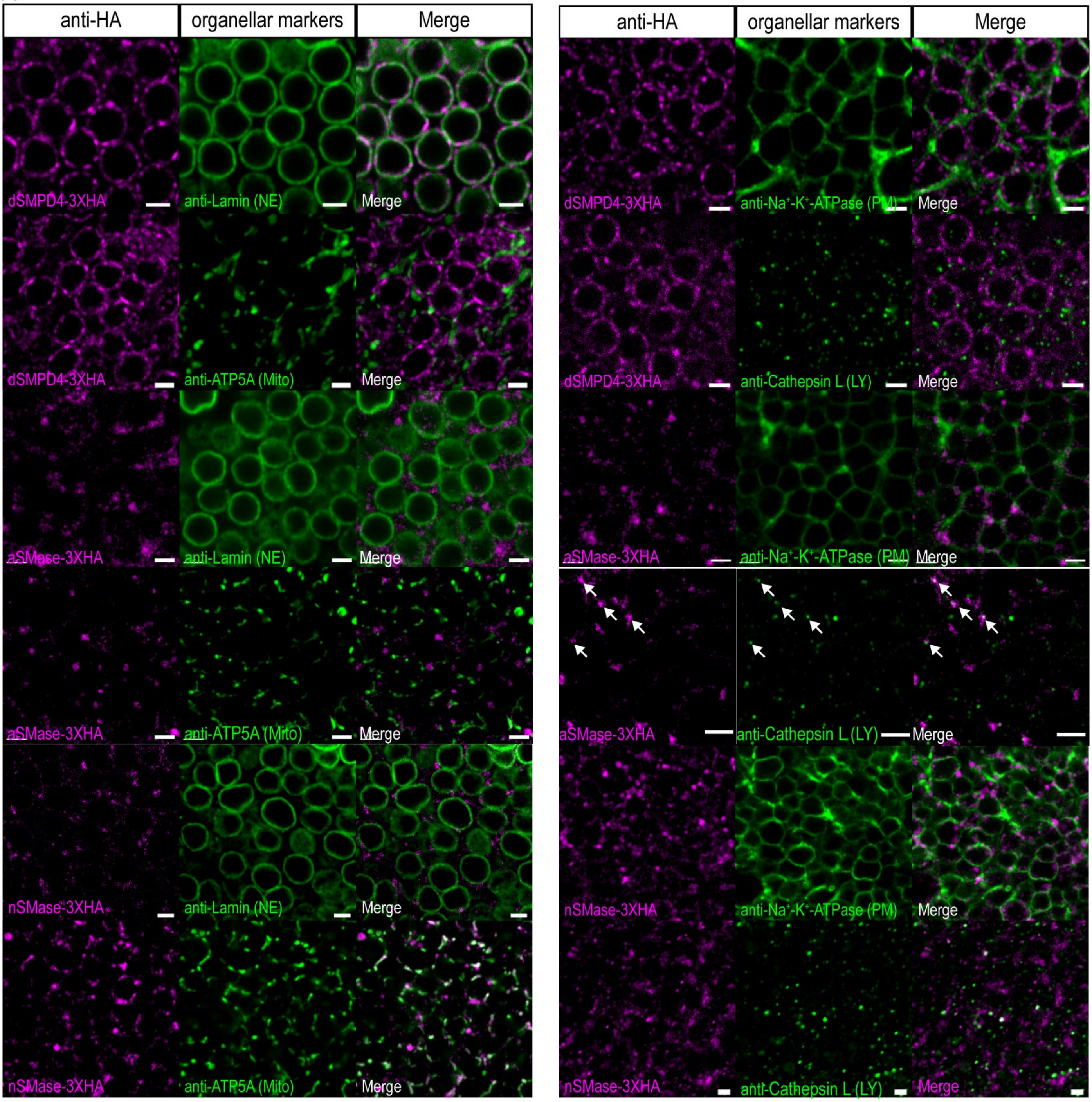

B

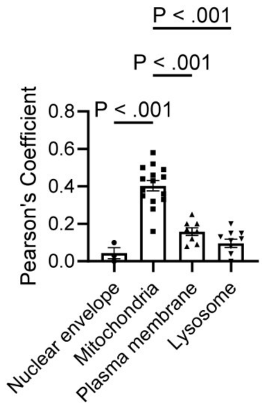

Appendix Figure S55 Subcellular localization of dSMPD4-3XHA, nSMase-3XHA, and aSMase-3XHA in the adult brain

A Representative confocal images of anti-HA stainings of organelle markers including anti-Lamin (nuclear envelope, NE), anti-Na^+-K^+-ATPase (Plasma membrane, PM), anti-ATP5A (Mitochondria, Mito), anti-Cathepsin L (Lysosome, LY) in the adult brains.

B Quantifications of Pearson's coefficient of nSMase-3XHA and organelle markers in the adult brains. Data are representative of at least 2 independent experiments. Data are represented as mean ± SEM (n ≥ 3). *P* values were calculated using one-way ANOVA with Dunnett's multiple comparisons.

Appendix Fig. S56

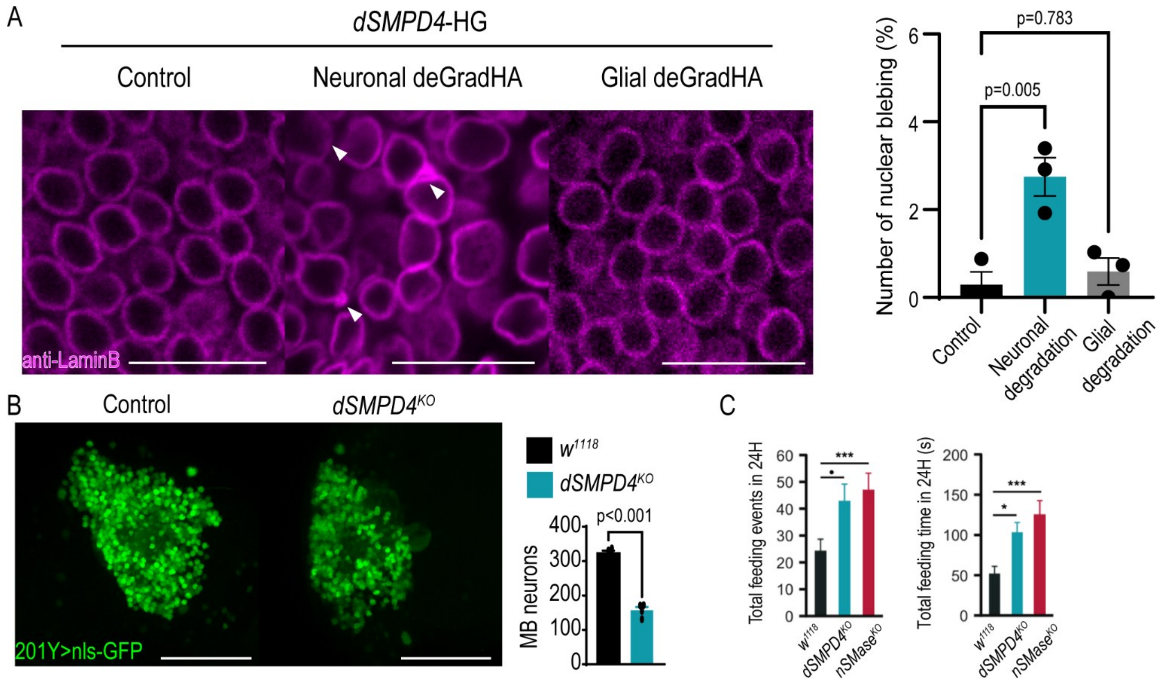

Appendix Figure S56 *dSMPD4* loss-of-function phenotypes

A Morphology of the nuclear membrane (anti-Lamin, magenta) in the adult brains of homozygous *dSMPD4*-HG flies with *LexAop-deGradHA* in neurons driven by *nsyb*-LexA and in glia driven by *repo*-LexA. (Right). Quantifications of the percentage of nuclei with blebs. Scale Bar = 20 µm. Control: +/+; *dSMPD4*-HG/*dSMPD4*-HG; Neuronal degradation: *nSyb*-LexA/*LexAop-deGradHA*; *dSMPD4*-HG/*dSMPD4*-HG; Glial degradation: *repo*-LexA/+; *LexAop-deGradHA*/+; *dSMPD4*-HG/*dSMPD4*-HG.

B Confocal images of mushroom body neuron with cell bodies labeled by *201Y*-GAL4-driven nls-GFP in the adult brains of *dSMPD4*^{KO} and control flies. (Right) quantification of mushroom body neuron numbers. Control: *201Y*-GAL4/*UAS-nls-GFP;+/+; dSMPD4*^{KO}: *201Y*-GAL4/*UAS-nls-GFP;dSMPD4*^{KO}/*dSMPD4*^{KO}.

C Quantification of total feeding events and total feeding time of *w*^{1118} control, *dSMPD4*^{KO}, *nSMase*^{KO} female flies (n = 16 for each group). *P* values (**P* <0.05, ****P* < 0.001) were calculated using one-way ANOVA with Dunnett's multiple comparisons.
