# Supplementary figures and images for: Functional profiling and visualization of the sphingolipid metabolic network in vivo (part 1 of 2)
Source: EMBO Rep. 2025 Nov 10;26(24):6380–417. doi: 10.1038/s44319-025-00632-0 (PMC12714868; doi:10.1038/s44319-025-00632-0)

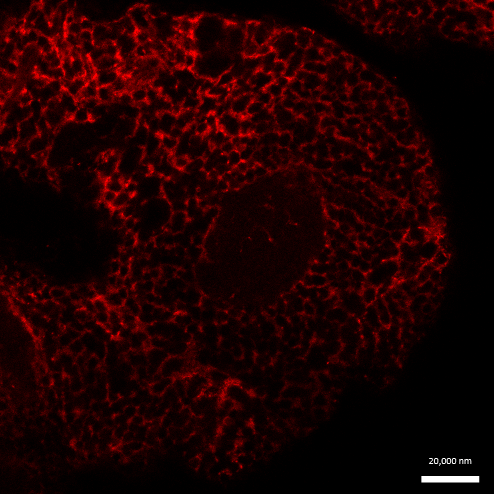

Supplement: Supplementary file 4 — Source data Fig. 1 [file 44319_2025_632_MOESM4_ESM.zip › Figure 1/1C/CDase mCD8GFP Luc-IR posterior 2/230223 CDase mCD8GFP Luc-IR posterior 2_c1.tif]

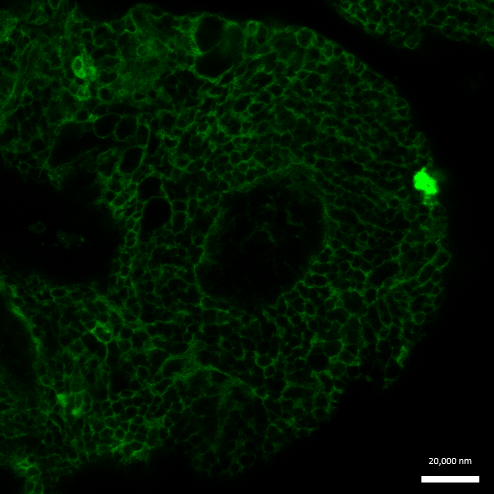

Supplement: Supplementary file 4 — Source data Fig. 1 [file 44319_2025_632_MOESM4_ESM.zip › Figure 1/1C/CDase mCD8GFP Luc-IR posterior 2/230223 CDase mCD8GFP Luc-IR posterior 2_c2.tif]

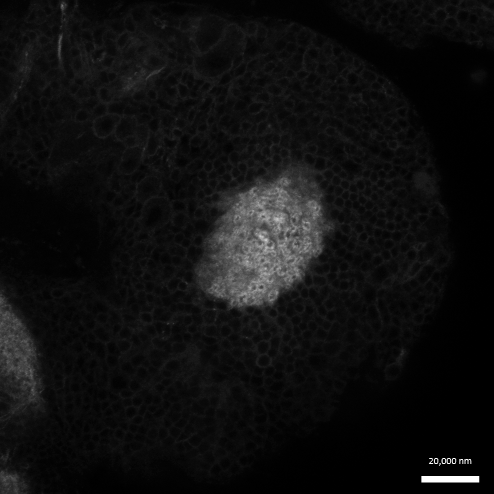

Supplement: Supplementary file 4 — Source data Fig. 1 [file 44319_2025_632_MOESM4_ESM.zip › Figure 1/1C/CDase mCD8GFP Luc-IR posterior 2/230223 CDase mCD8GFP Luc-IR posterior 2_c3.tif]

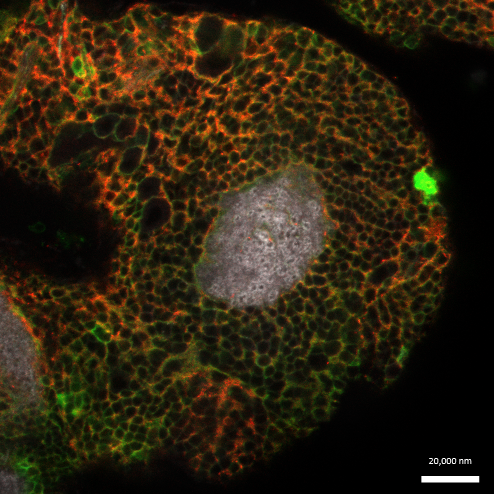

Supplement: Supplementary file 4 — Source data Fig. 1 [file 44319_2025_632_MOESM4_ESM.zip › Figure 1/1C/CDase mCD8GFP Luc-IR posterior 2/230223 CDase mCD8GFP Luc-IR posterior 2_c1+2+3.tif]

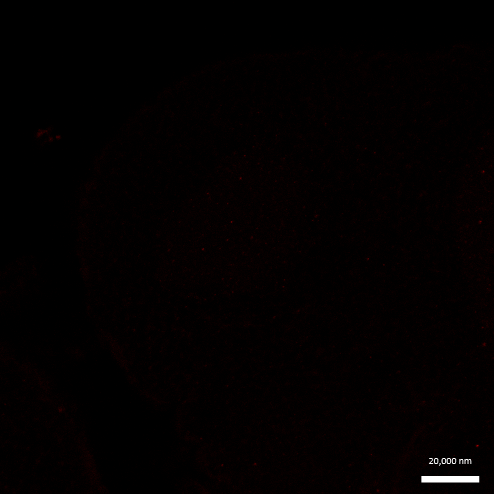

Supplement: Supplementary file 4 — Source data Fig. 1 [file 44319_2025_632_MOESM4_ESM.zip › Figure 1/1C/CDase mCD8GFP CDase-IR posterior 1/230223 CDase mCD8GFP CDase-IR posterior 1_c1.tif]

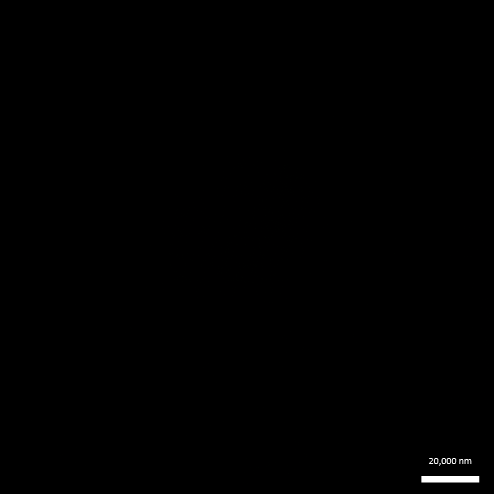

Supplement: Supplementary file 4 — Source data Fig. 1 [file 44319_2025_632_MOESM4_ESM.zip › Figure 1/1C/CDase mCD8GFP CDase-IR posterior 1/230223 CDase mCD8GFP CDase-IR posterior 1_c2.tif]

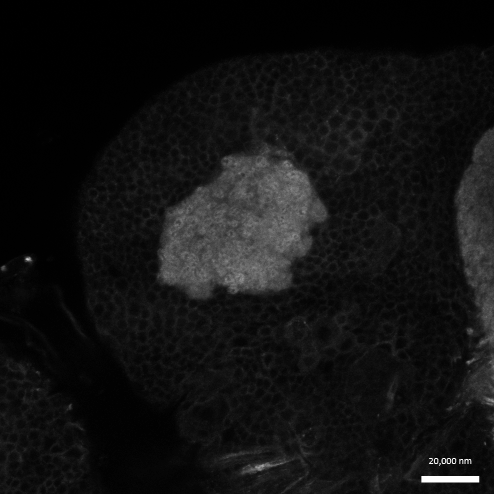

Supplement: Supplementary file 4 — Source data Fig. 1 [file 44319_2025_632_MOESM4_ESM.zip › Figure 1/1C/CDase mCD8GFP CDase-IR posterior 1/230223 CDase mCD8GFP CDase-IR posterior 1_c3.tif]

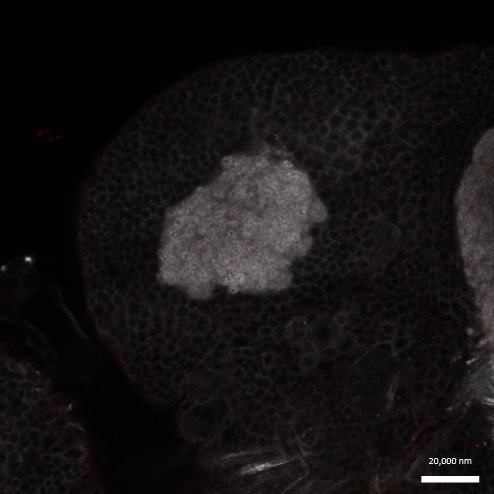

Supplement: Supplementary file 4 — Source data Fig. 1 [file 44319_2025_632_MOESM4_ESM.zip › Figure 1/1C/CDase mCD8GFP CDase-IR posterior 1/230223 CDase mCD8GFP CDase-IR posterior 1_c1+2+3.tif]

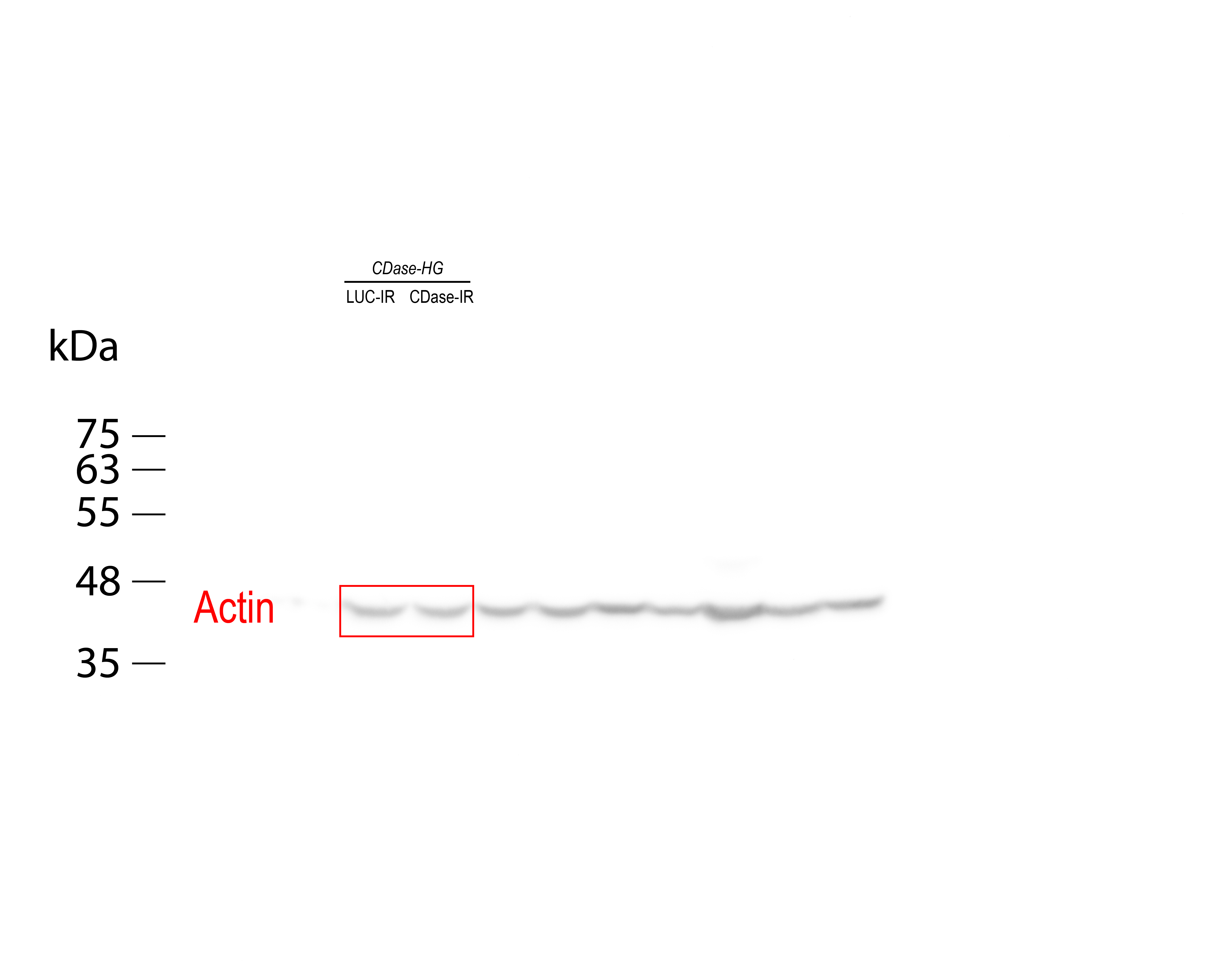

Supplement: Supplementary file 4 — Source data Fig. 1 [file 44319_2025_632_MOESM4_ESM.zip › Figure 1/1D/Actin/Fig 1D Actin.jpg]

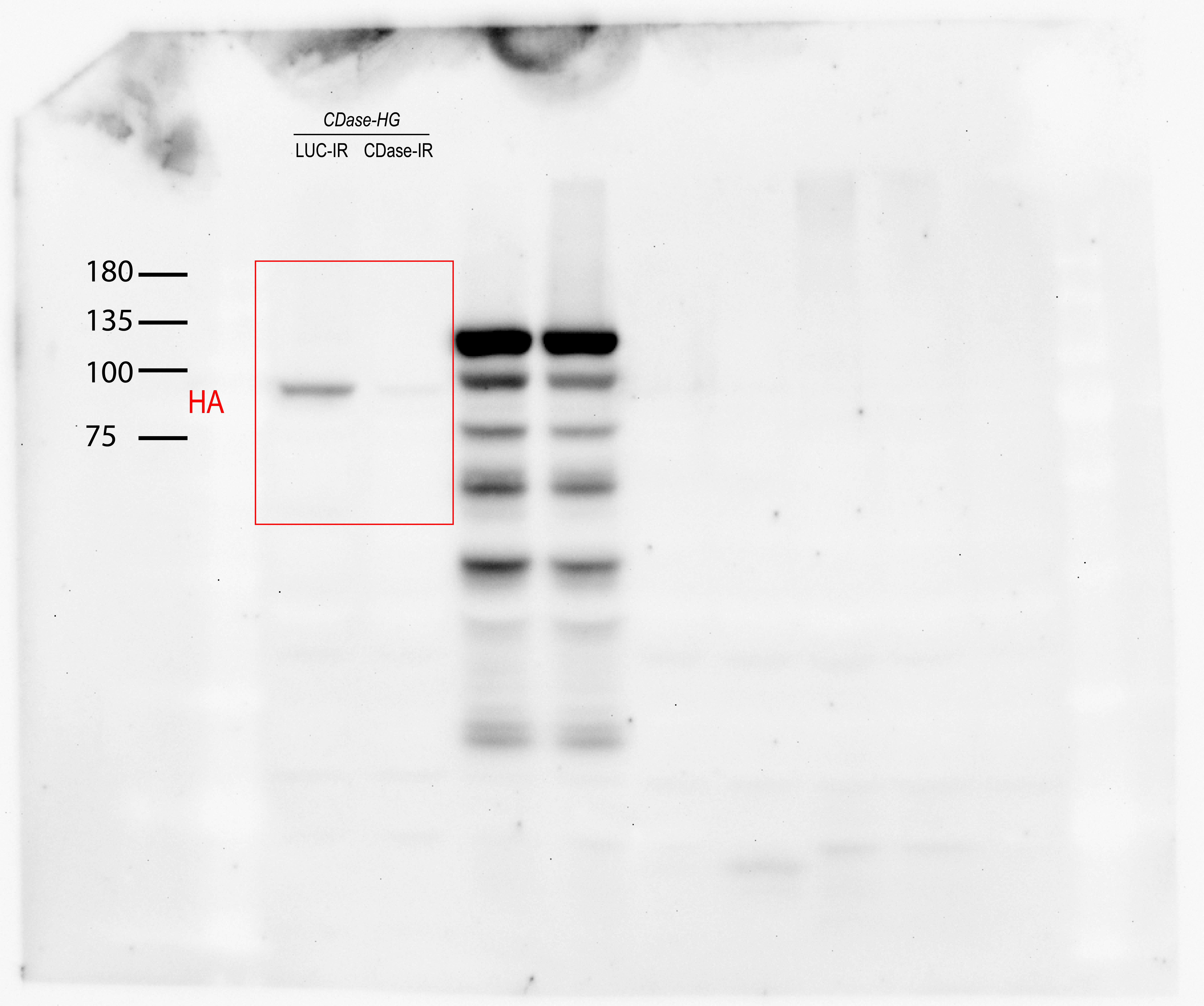

Supplement: Supplementary file 4 — Source data Fig. 1 [file 44319_2025_632_MOESM4_ESM.zip › Figure 1/1D/HA/Fig 1D HA.jpg]

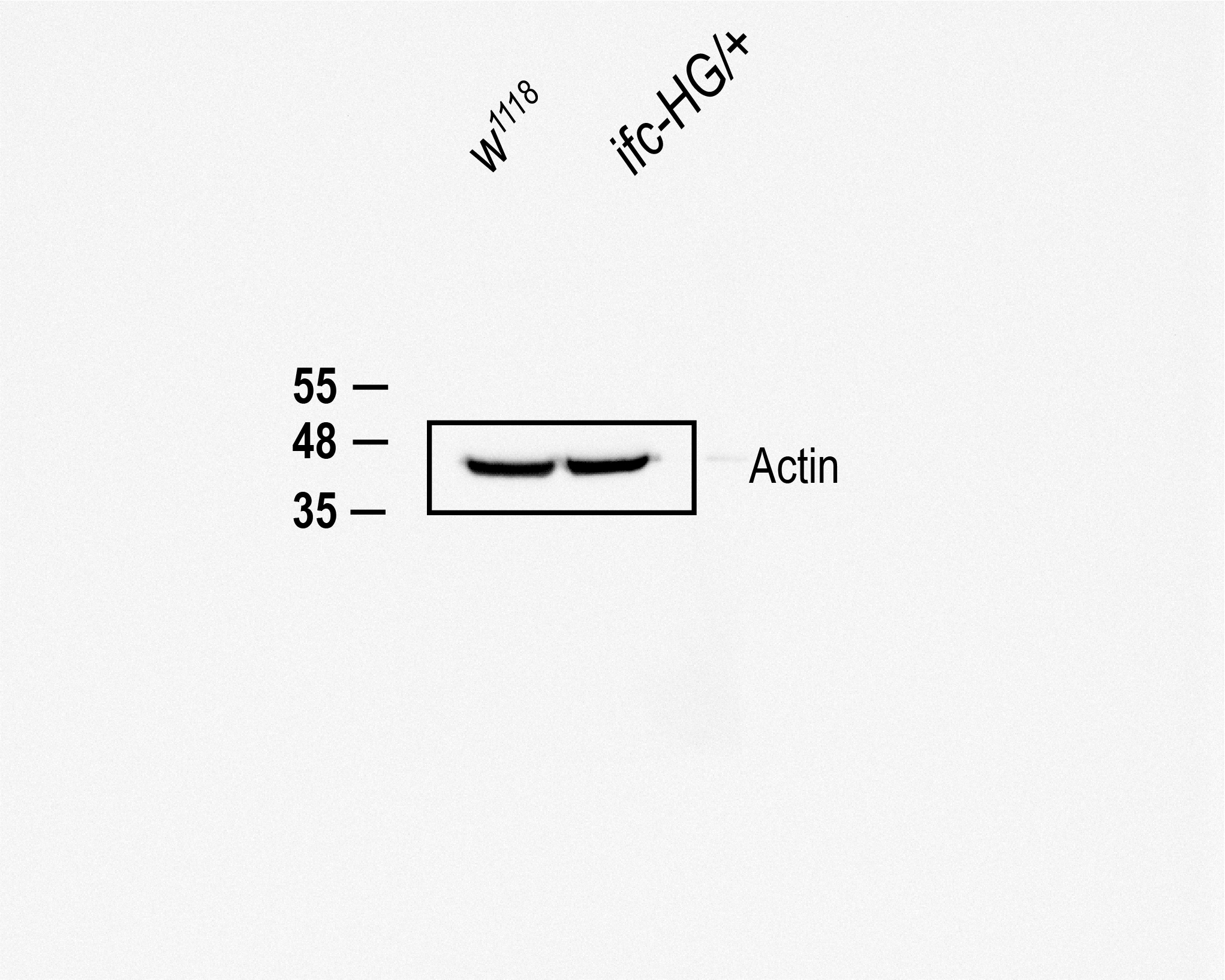

Supplement: Supplementary file 5 — Source data Fig. 2 [file 44319_2025_632_MOESM5_ESM.zip › Figure 2/2A/Actin/Fig 2A Actin.jpg]

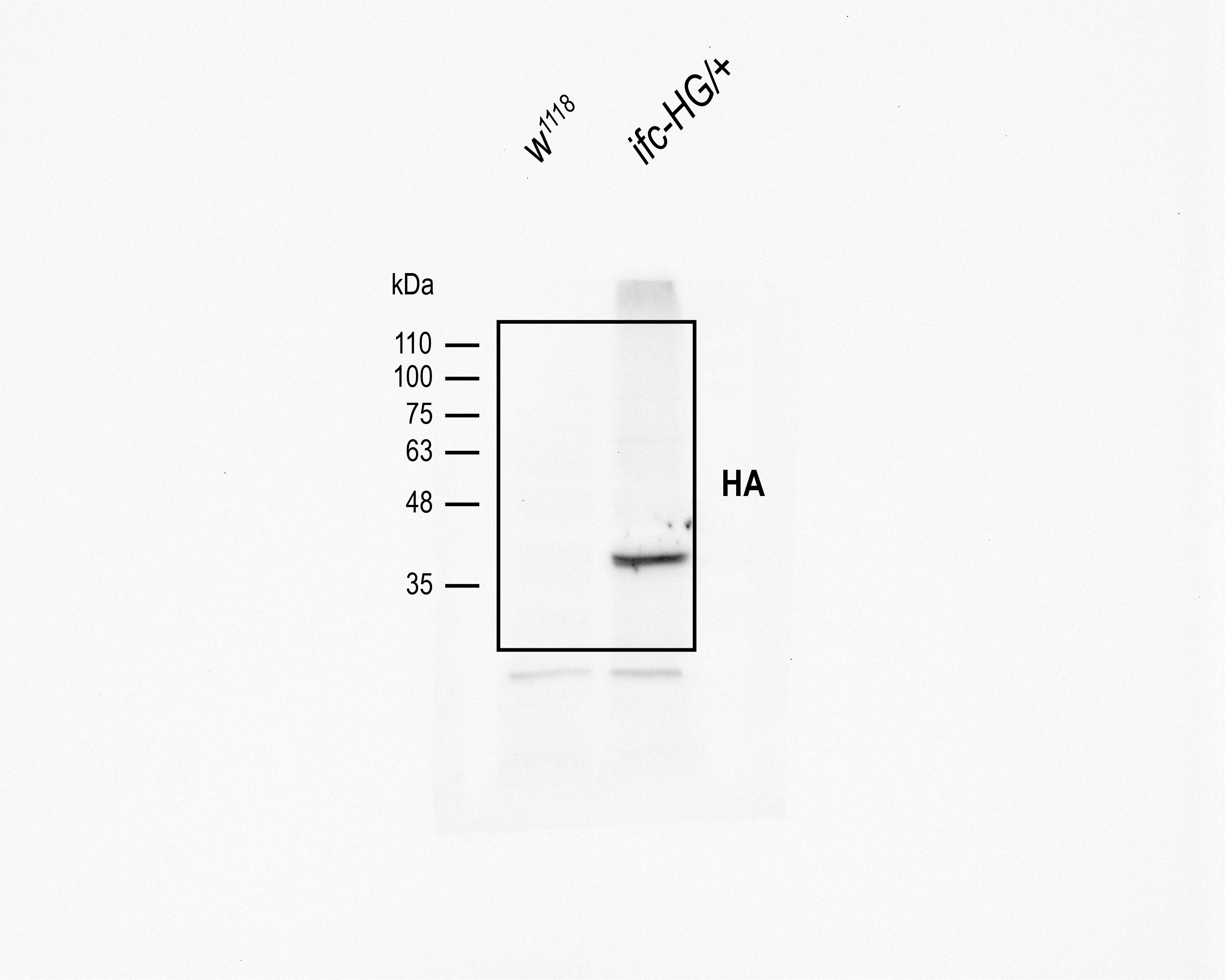

Supplement: Supplementary file 5 — Source data Fig. 2 [file 44319_2025_632_MOESM5_ESM.zip › Figure 2/2A/HA/Fig 2A HA.jpg]

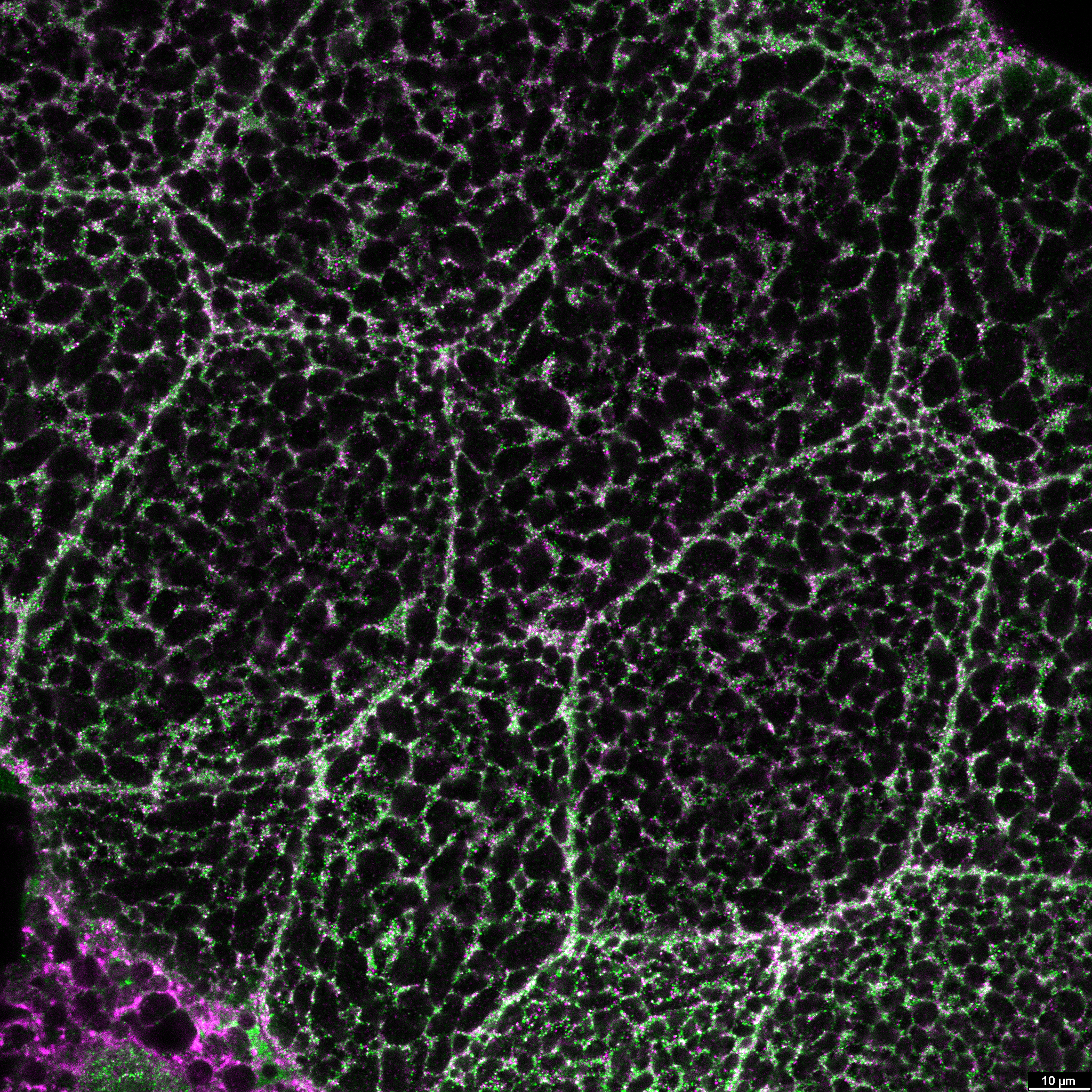

Supplement: Supplementary file 5 — Source data Fig. 2 [file 44319_2025_632_MOESM5_ESM.zip › Figure 2/2B/2B Merge.tif]

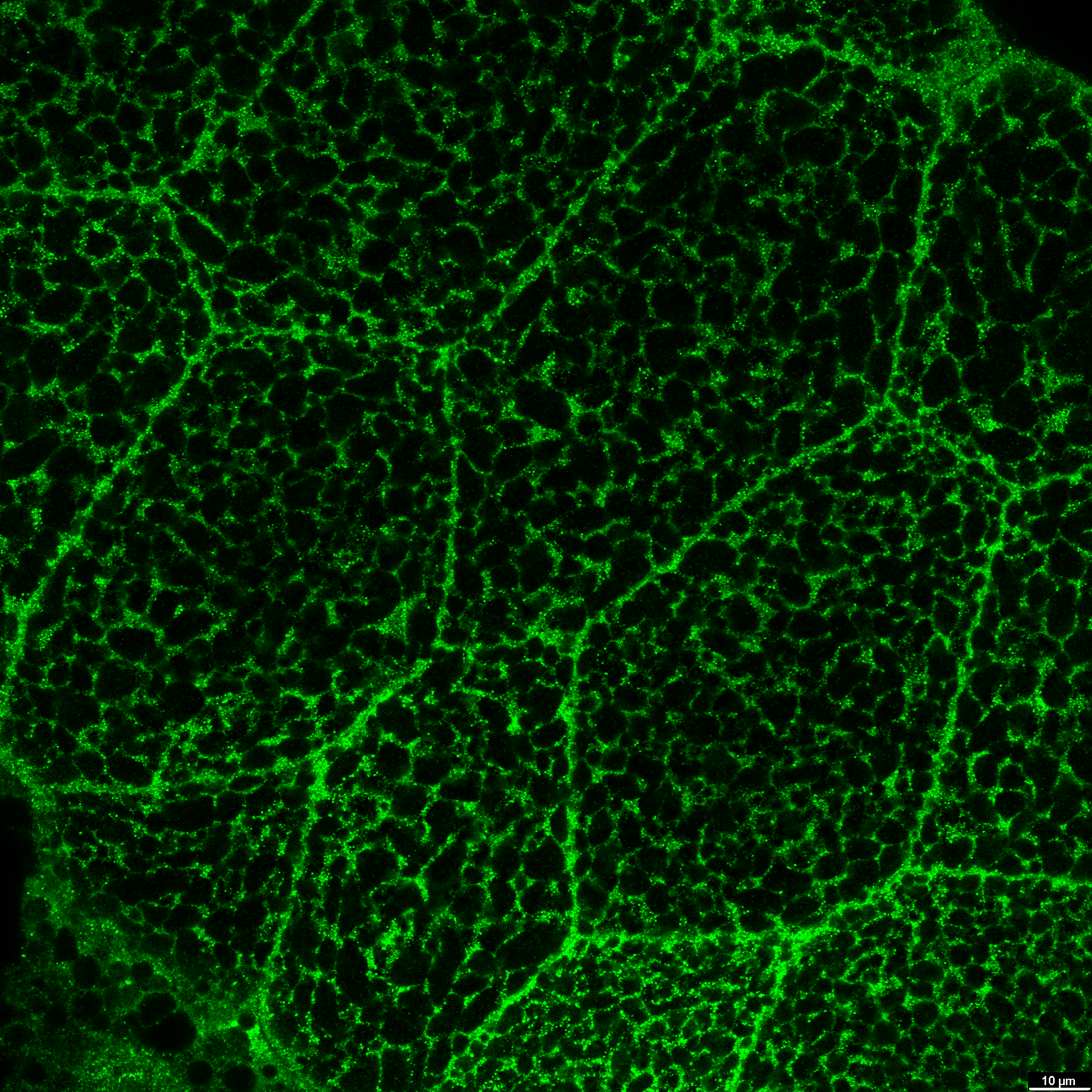

Supplement: Supplementary file 5 — Source data Fig. 2 [file 44319_2025_632_MOESM5_ESM.zip › Figure 2/2B/2B_ch00.tif]

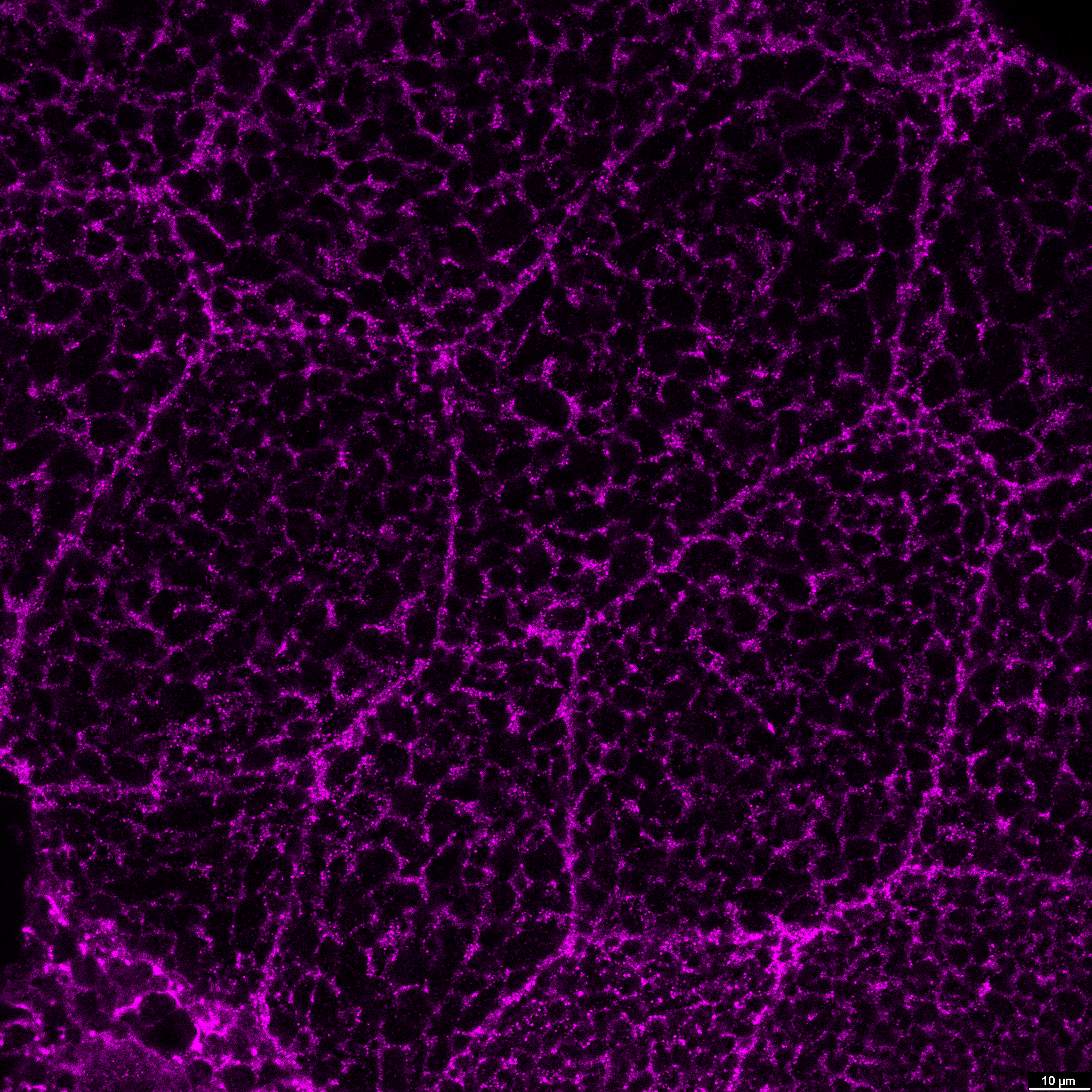

Supplement: Supplementary file 5 — Source data Fig. 2 [file 44319_2025_632_MOESM5_ESM.zip › Figure 2/2B/2B_ch01.tif]

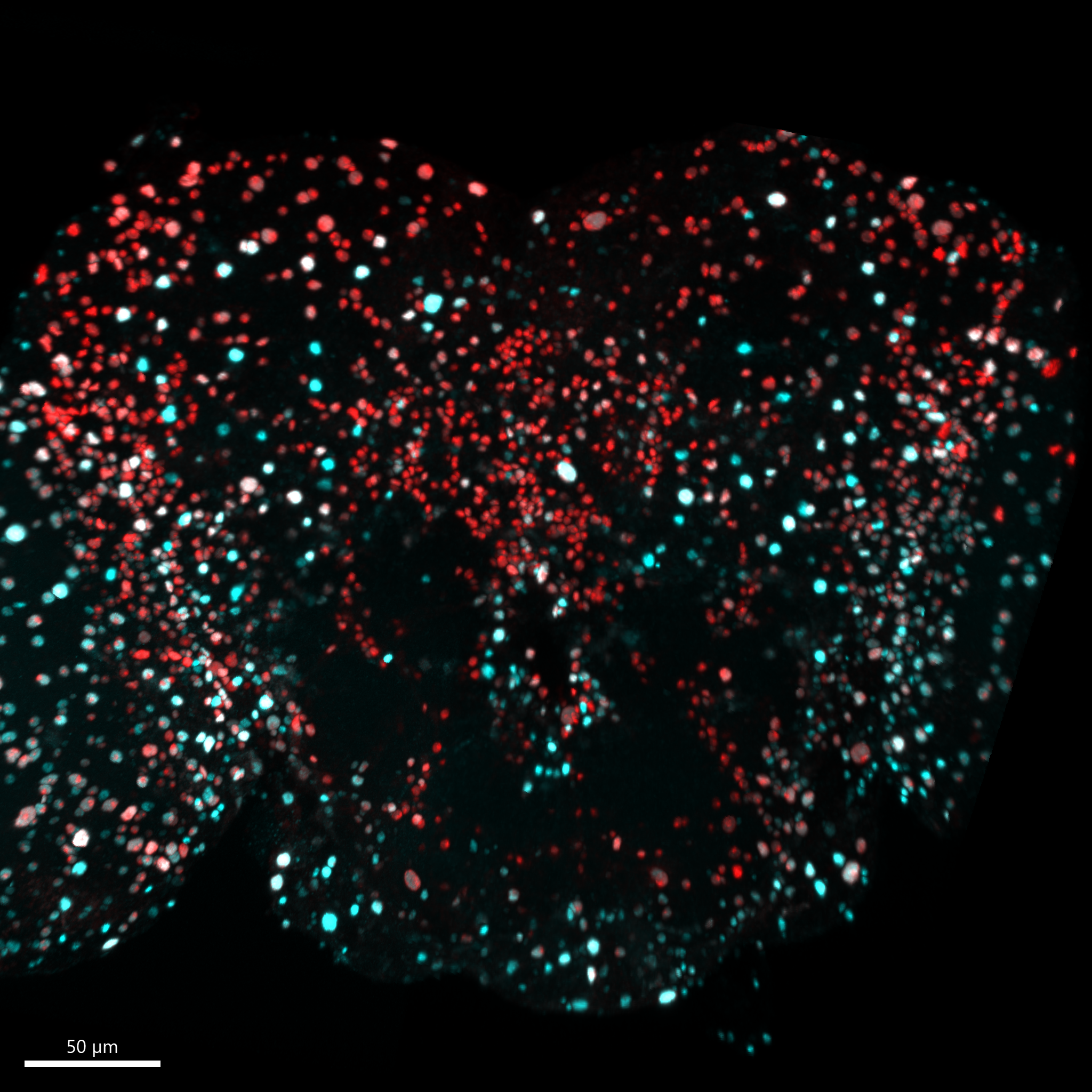

Supplement: Supplementary file 5 — Source data Fig. 2 [file 44319_2025_632_MOESM5_ESM.zip › Figure 2/2C/sk1-HG nls-mCherry Merge.tif]

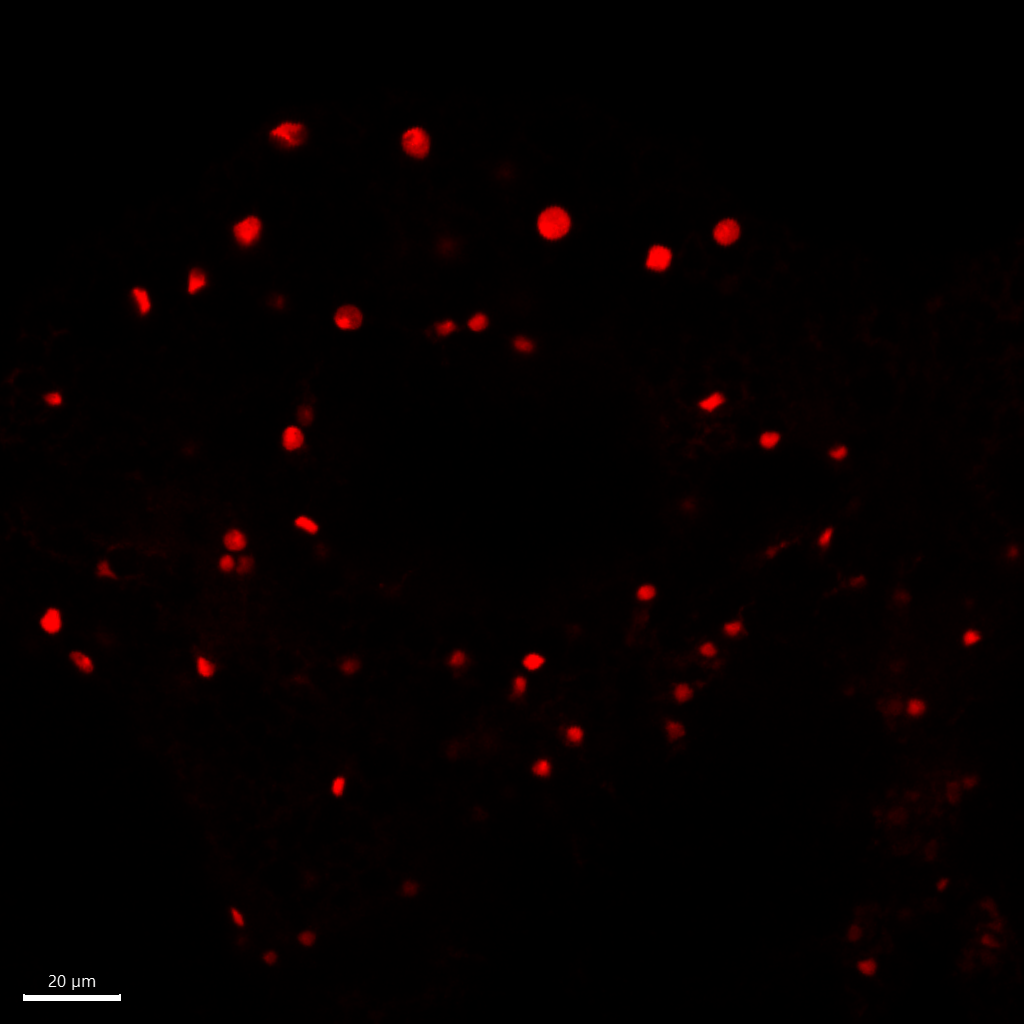

Supplement: Supplementary file 5 — Source data Fig. 2 [file 44319_2025_632_MOESM5_ESM.zip › Figure 2/2C/sk1-HG nls-mCherry inset mCherry.tif]

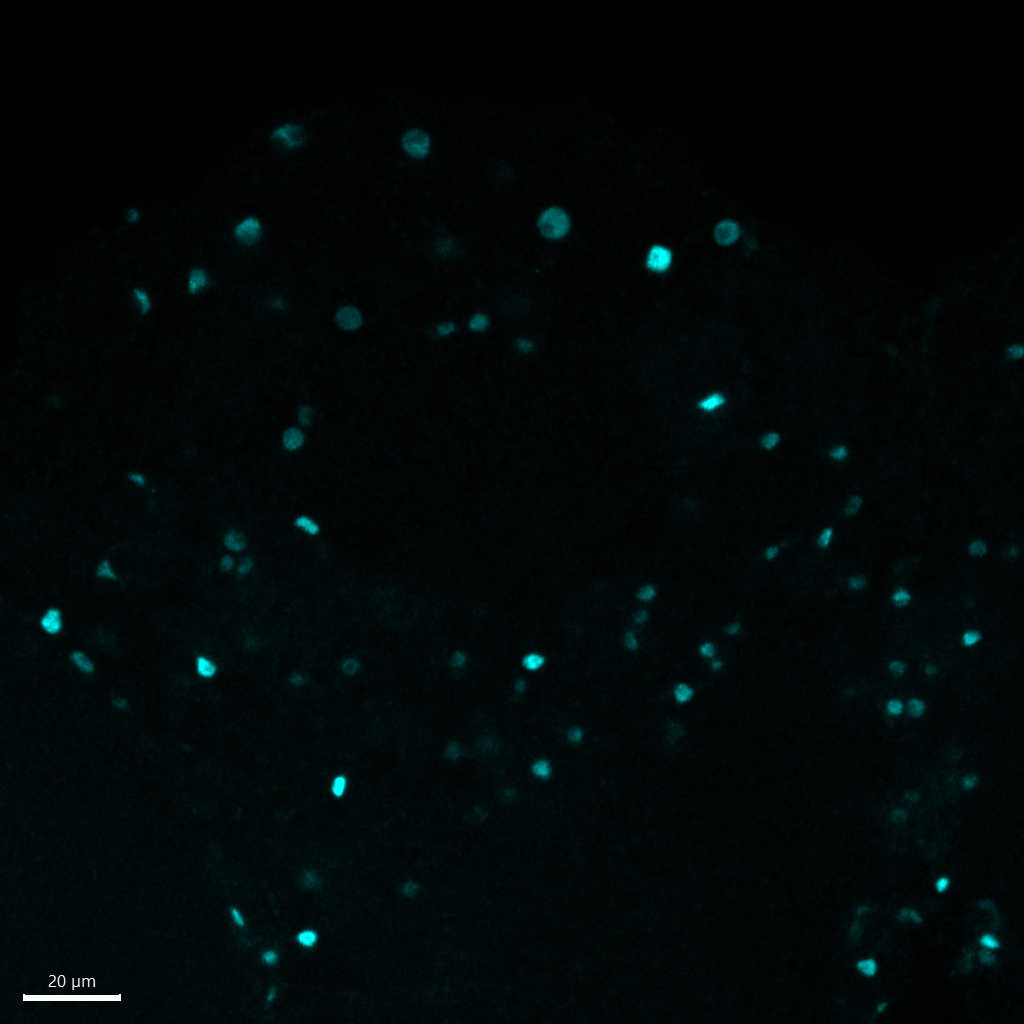

Supplement: Supplementary file 5 — Source data Fig. 2 [file 44319_2025_632_MOESM5_ESM.zip › Figure 2/2C/sk1-HG nls-mCherry inset repo.tif]

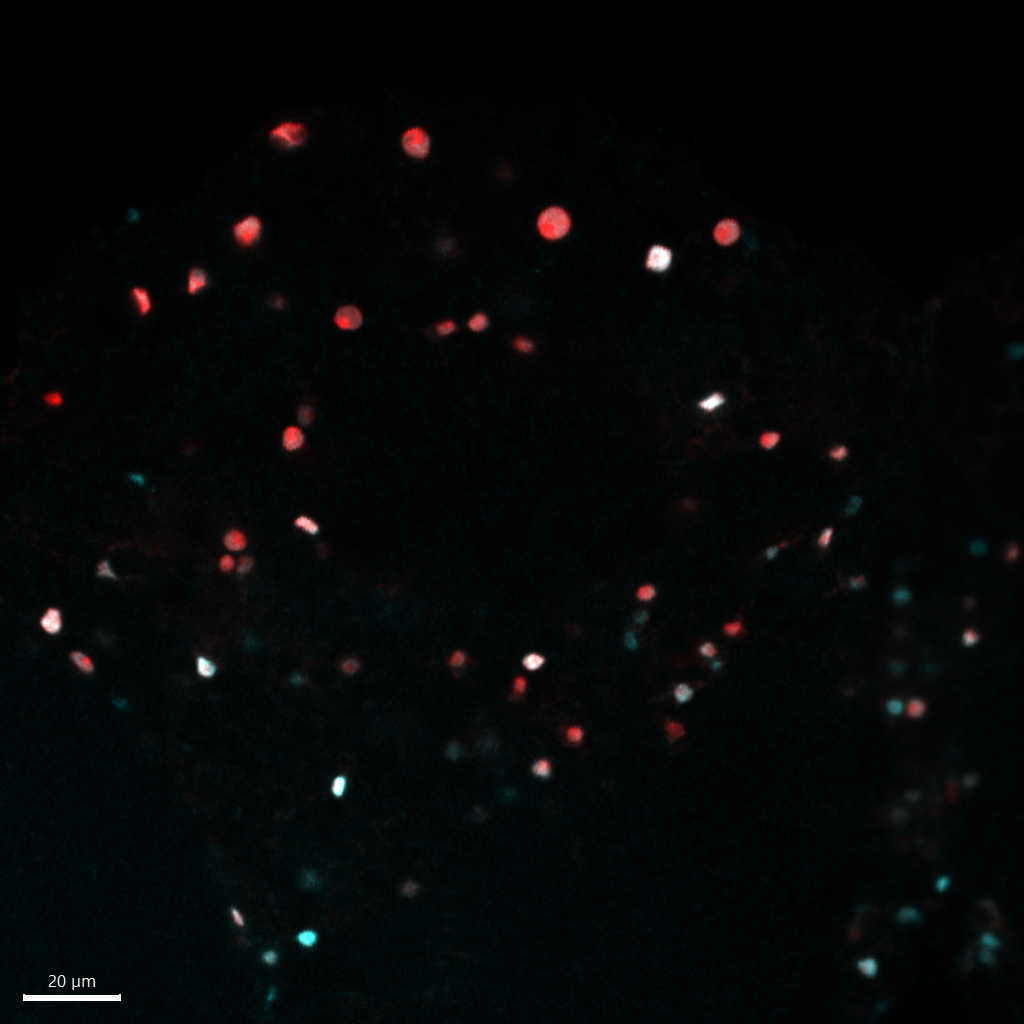

Supplement: Supplementary file 5 — Source data Fig. 2 [file 44319_2025_632_MOESM5_ESM.zip › Figure 2/2C/sk1-HG nls-mCherry inset Merge.tif]

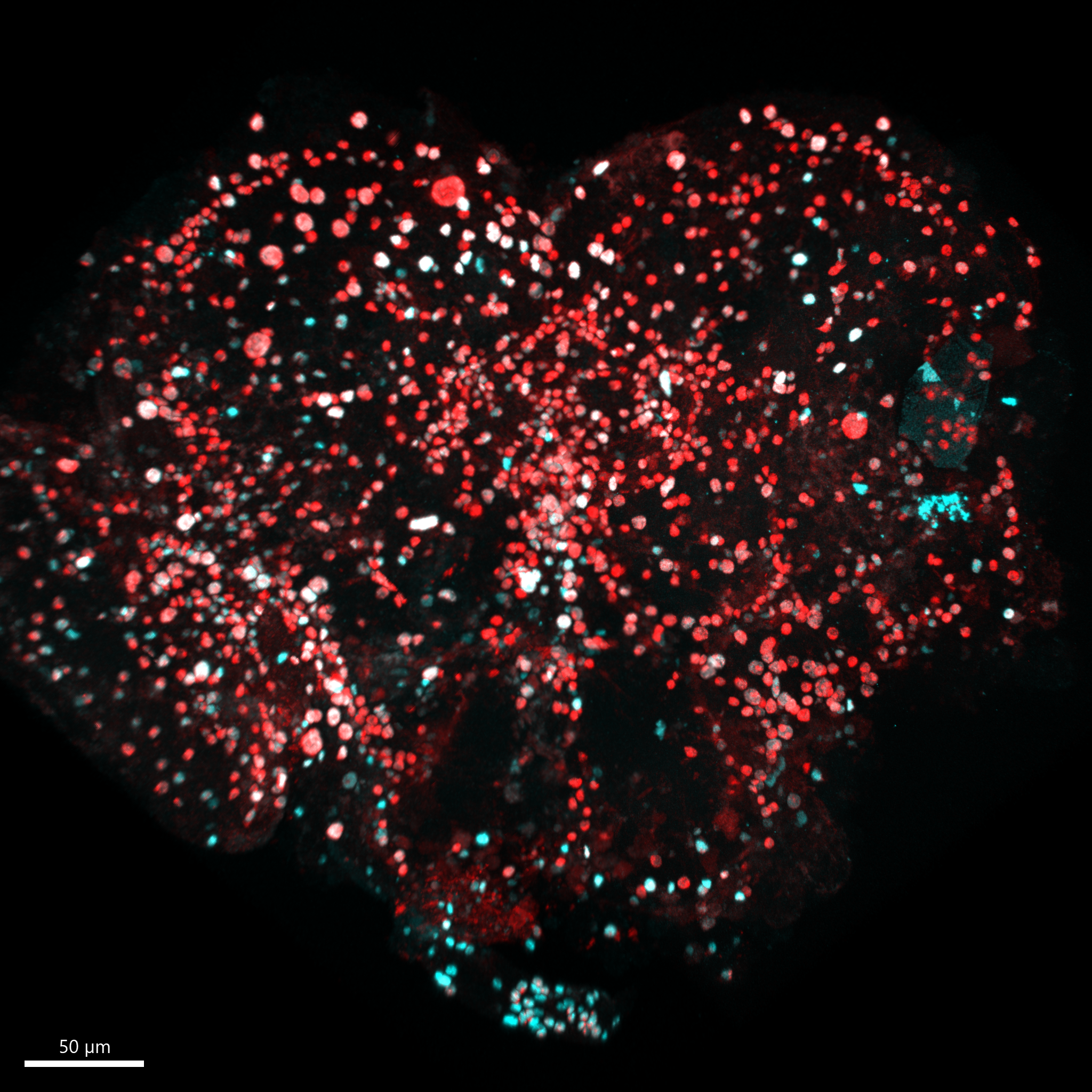

Supplement: Supplementary file 5 — Source data Fig. 2 [file 44319_2025_632_MOESM5_ESM.zip › Figure 2/2C/sk1-CRIMIC nls-mCherry Merge.tif]

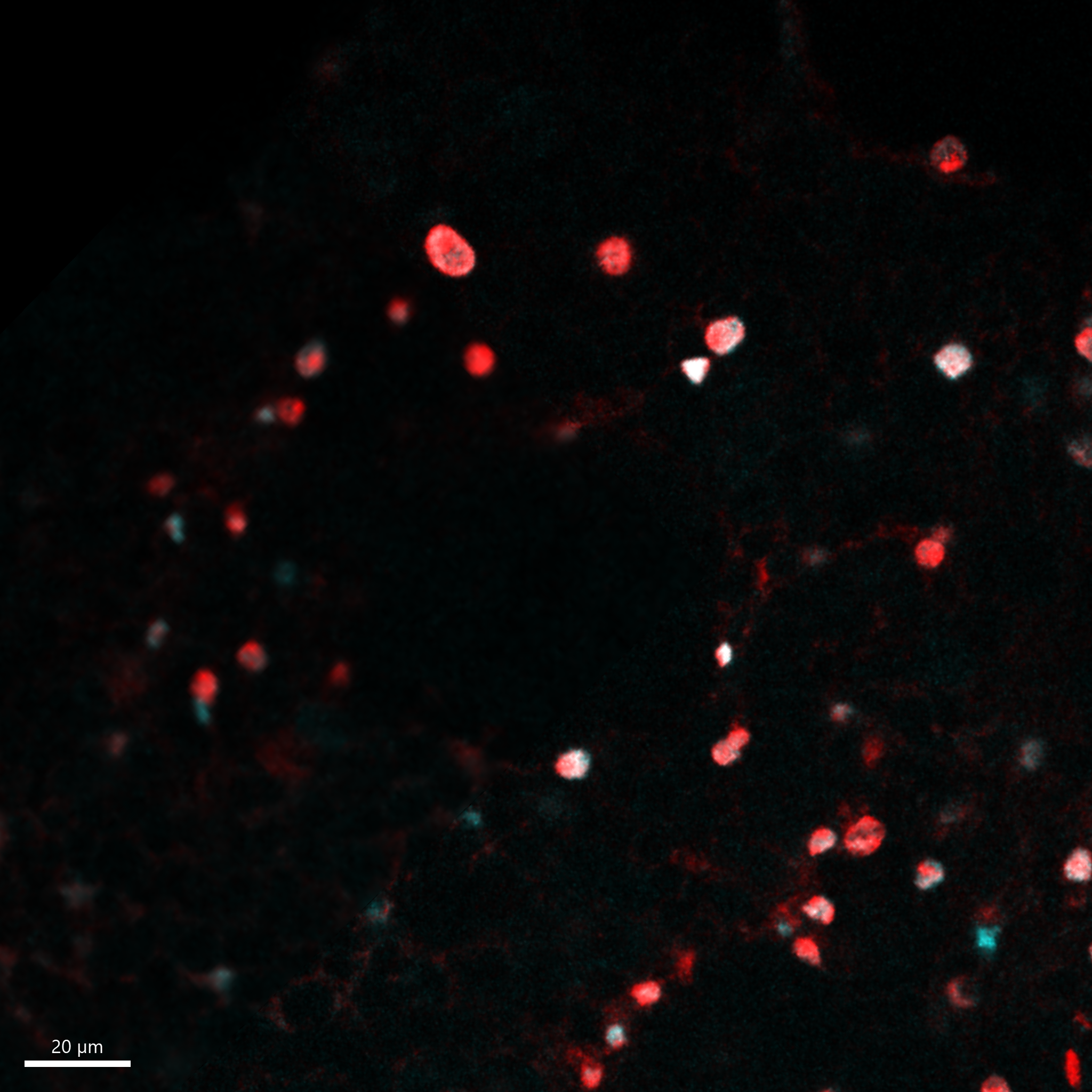

Supplement: Supplementary file 5 — Source data Fig. 2 [file 44319_2025_632_MOESM5_ESM.zip › Figure 2/2C/sk1-CRIMIC nls-mCherry inset Merge.tif]

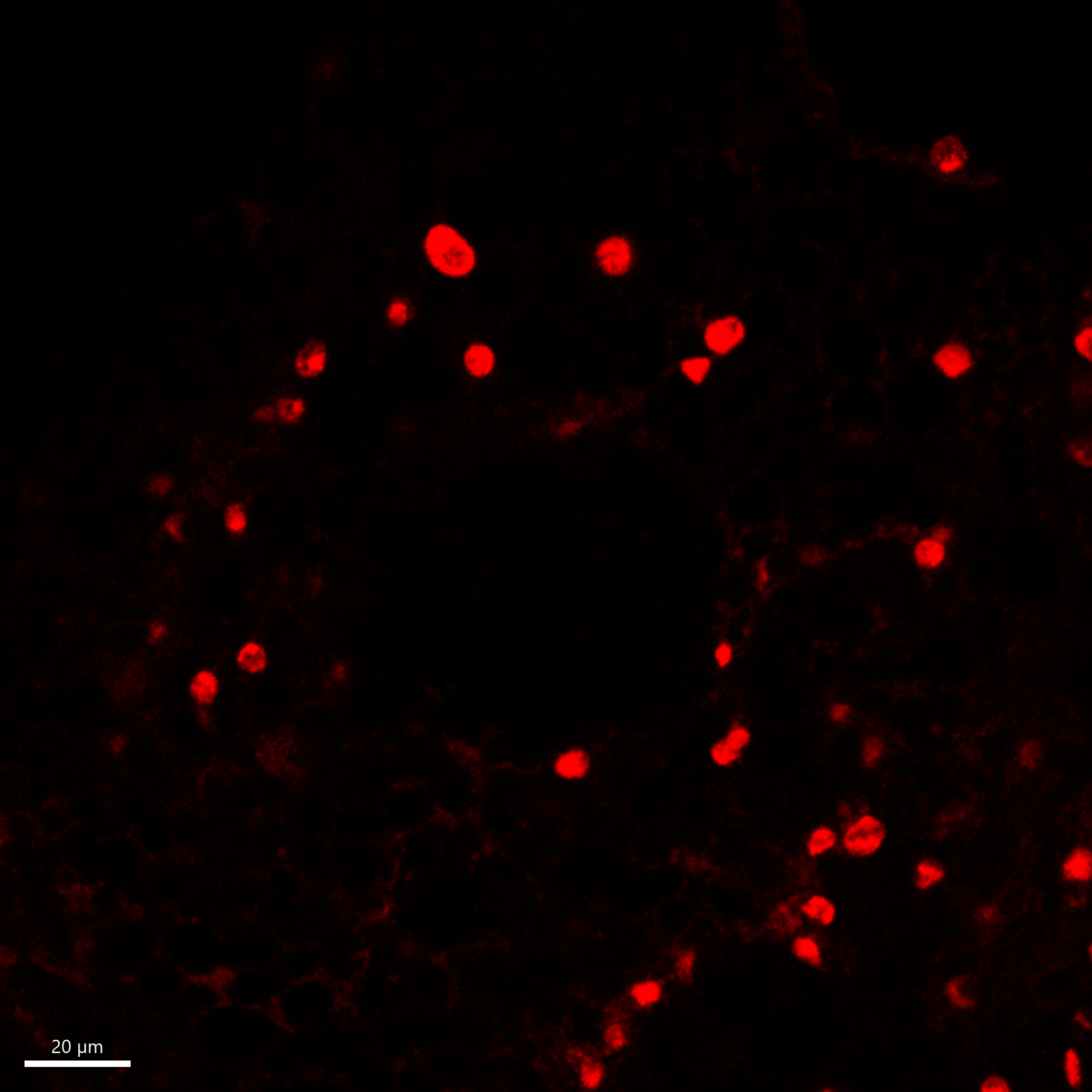

Supplement: Supplementary file 5 — Source data Fig. 2 [file 44319_2025_632_MOESM5_ESM.zip › Figure 2/2C/sk1-CRIMIC nls-mCherry inset mCherry.tif]

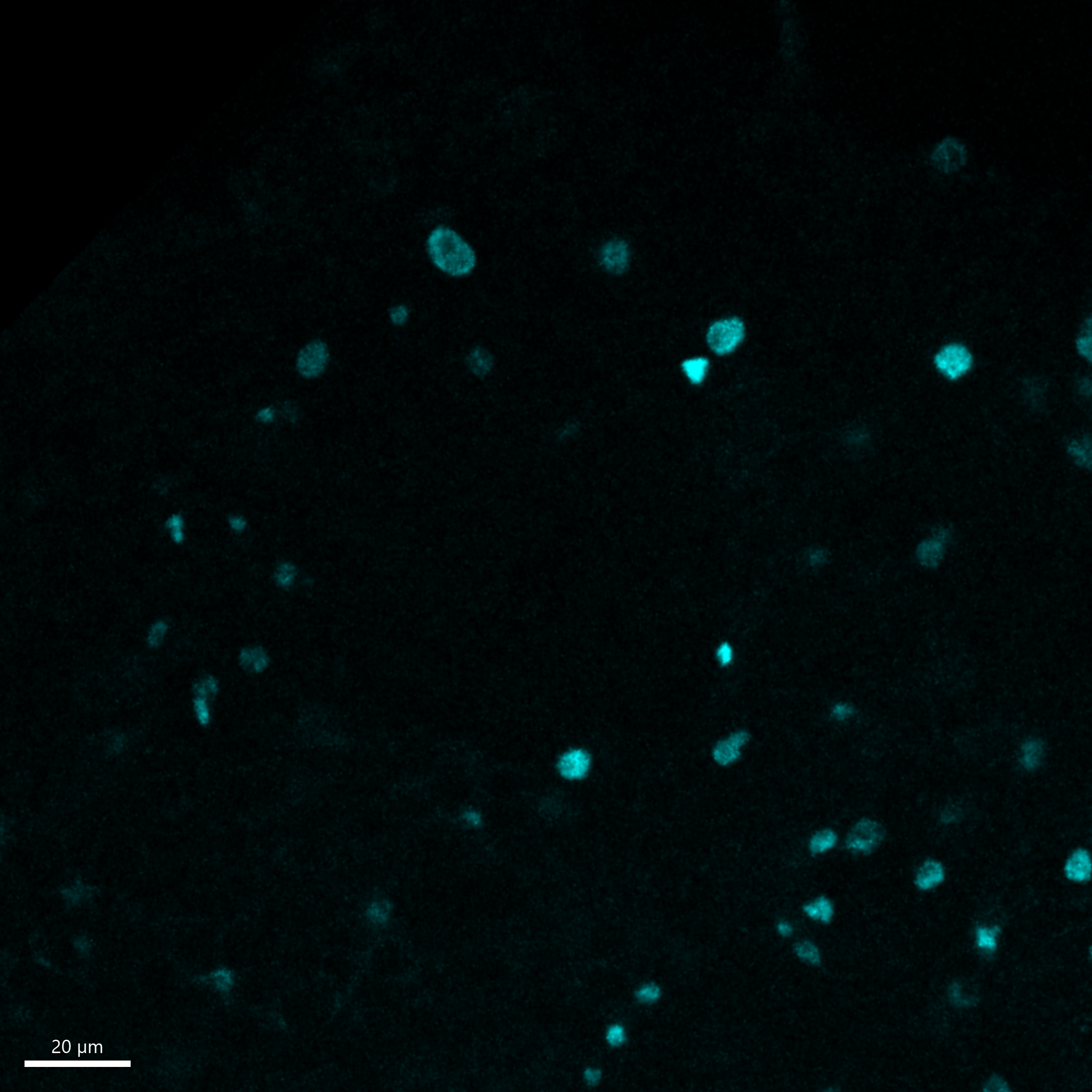

Supplement: Supplementary file 5 — Source data Fig. 2 [file 44319_2025_632_MOESM5_ESM.zip › Figure 2/2C/sk1-CRIMIC nls-mCherry inset repo.tif]

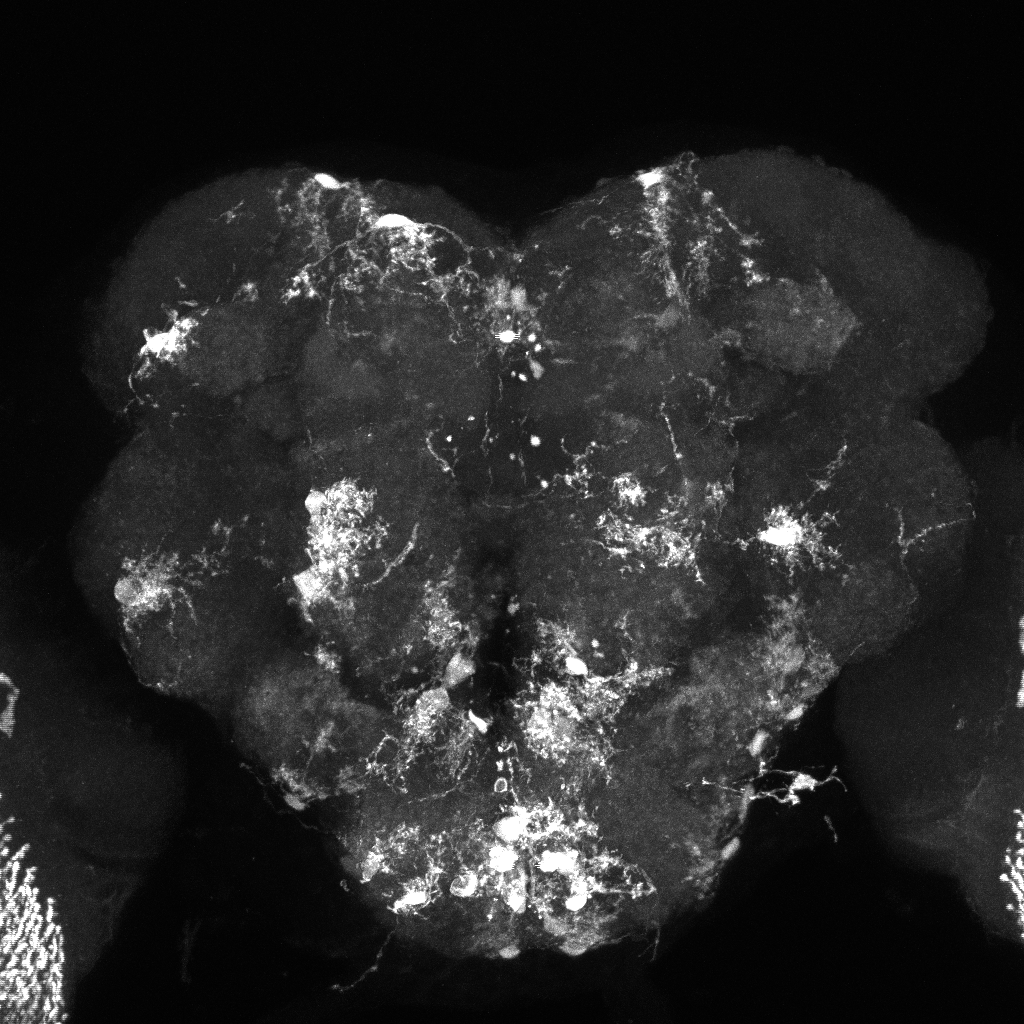

Supplement: Supplementary file 5 — Source data Fig. 2 [file 44319_2025_632_MOESM5_ESM.zip › Figure 2/2G/(top)R25H07 wun2 rCD2RFP_ORG.tif]

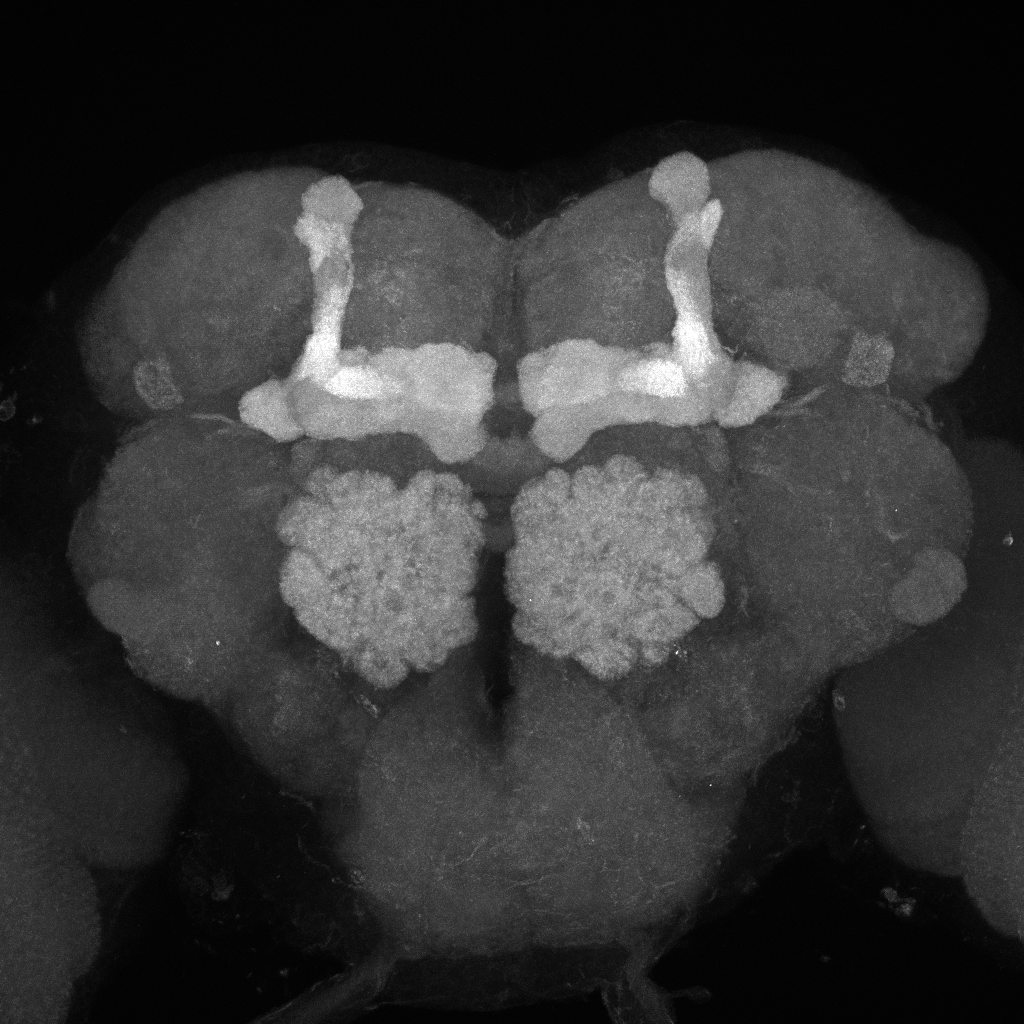

Supplement: Supplementary file 5 — Source data Fig. 2 [file 44319_2025_632_MOESM5_ESM.zip › Figure 2/2G/(top)R25H07 wun2 DLG_ORG.tif]

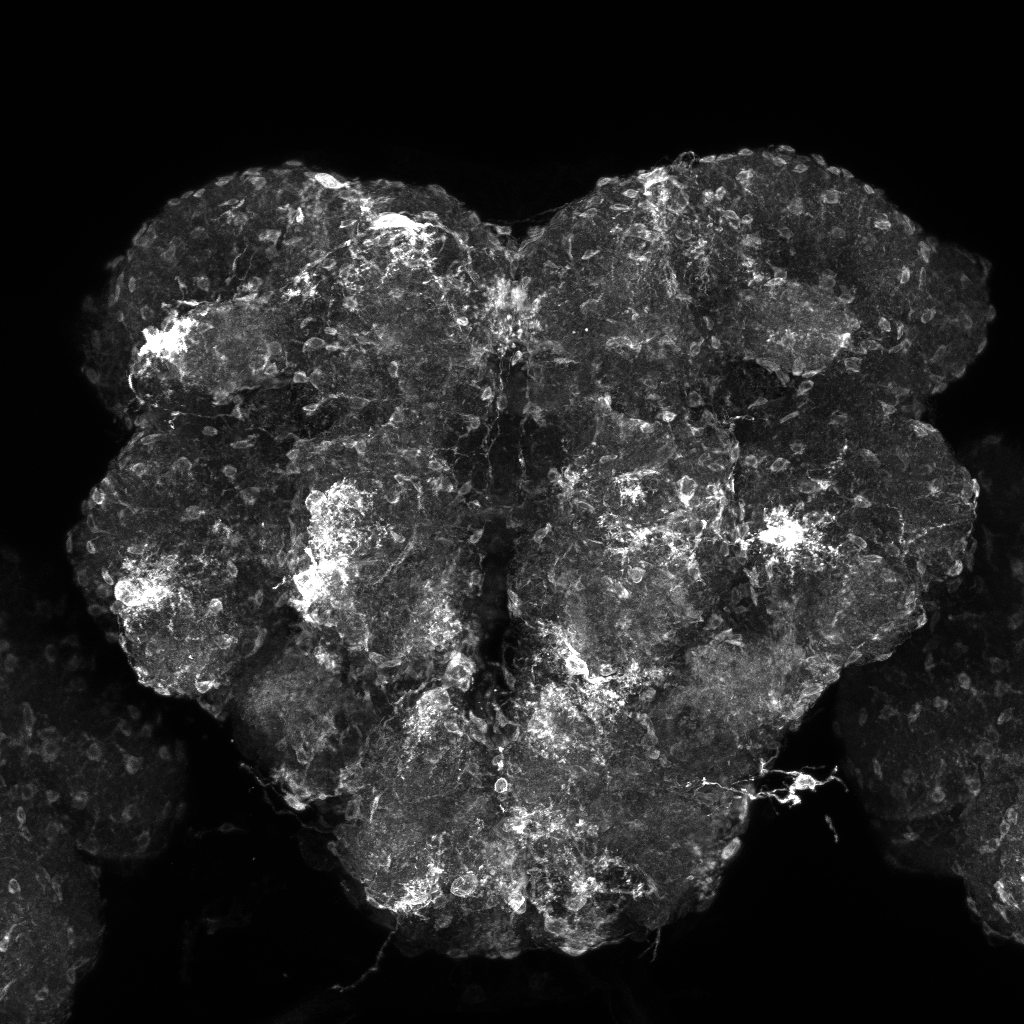

Supplement: Supplementary file 5 — Source data Fig. 2 [file 44319_2025_632_MOESM5_ESM.zip › Figure 2/2G/(top)R25H07 wun2 mCD8GFP_ORG.tif]

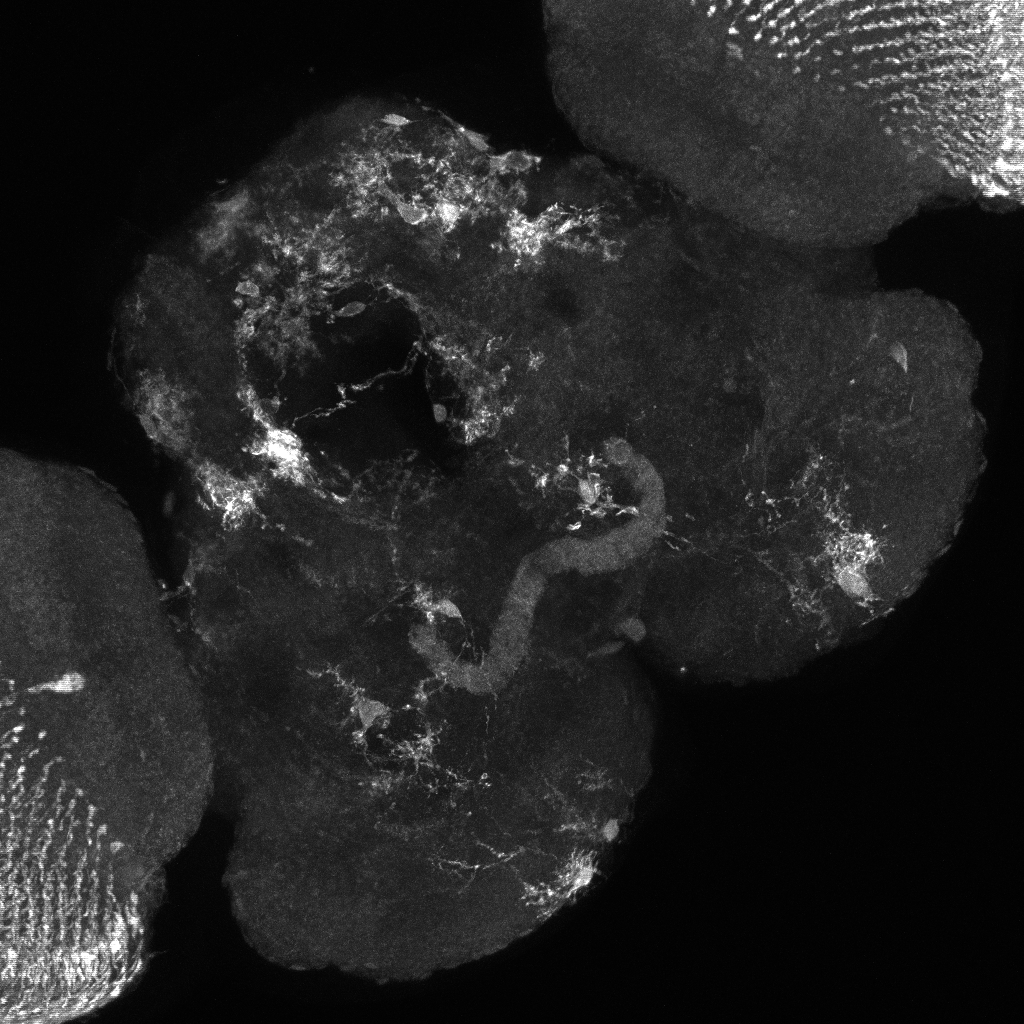

Supplement: Supplementary file 5 — Source data Fig. 2 [file 44319_2025_632_MOESM5_ESM.zip › Figure 2/2G/(bot)R25H07 wun2 rCD2RFP_ORG.tif]

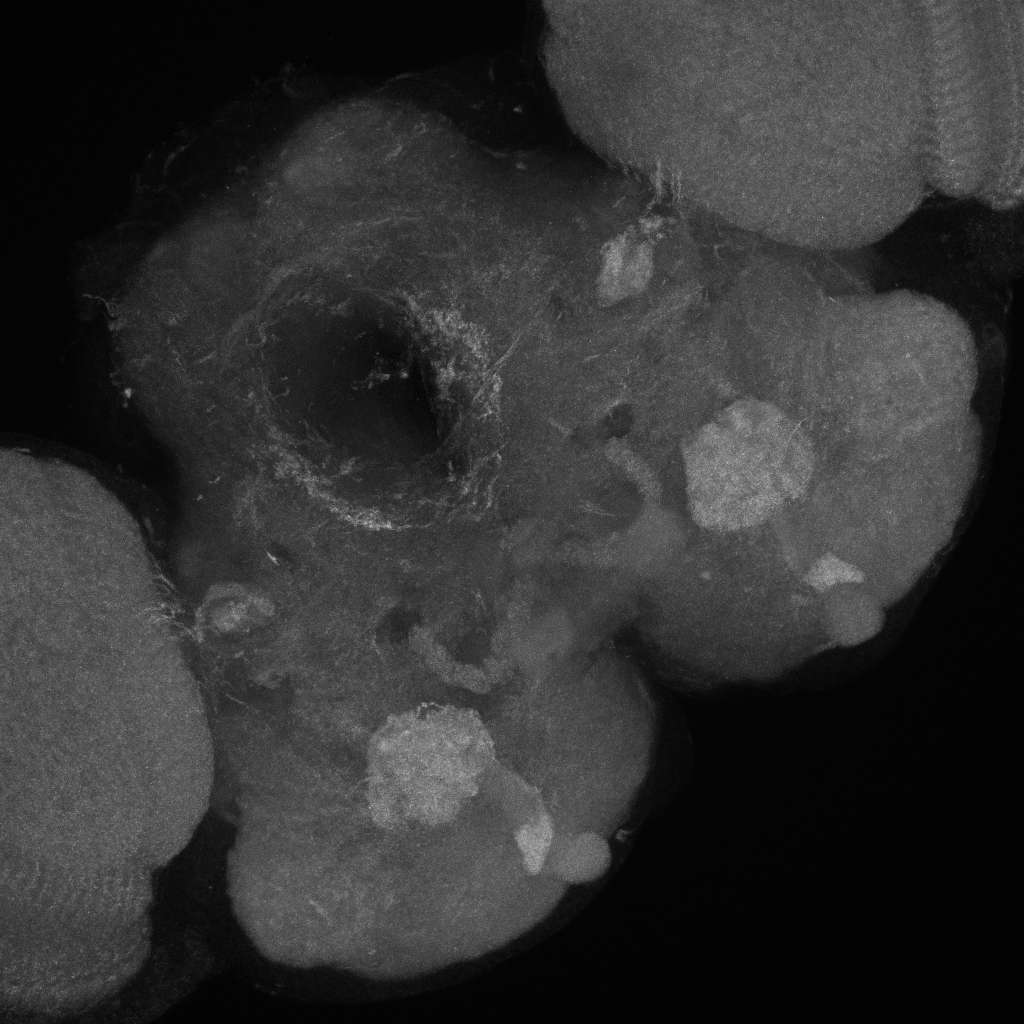

Supplement: Supplementary file 5 — Source data Fig. 2 [file 44319_2025_632_MOESM5_ESM.zip › Figure 2/2G/(bot)R25H07 wun2 DLG_ORG.tif]

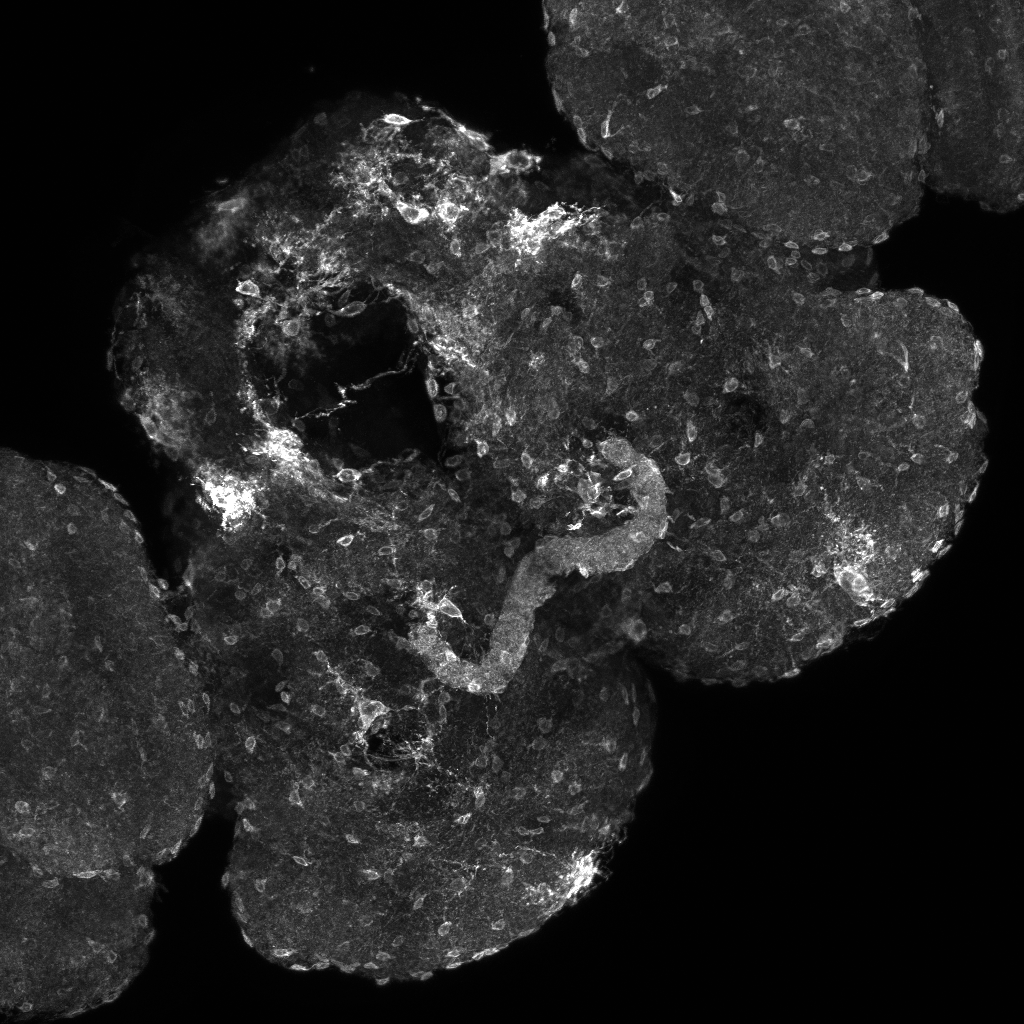

Supplement: Supplementary file 5 — Source data Fig. 2 [file 44319_2025_632_MOESM5_ESM.zip › Figure 2/2G/(bot)R25H07 wun2 mCD8GFP_ORG.tif]

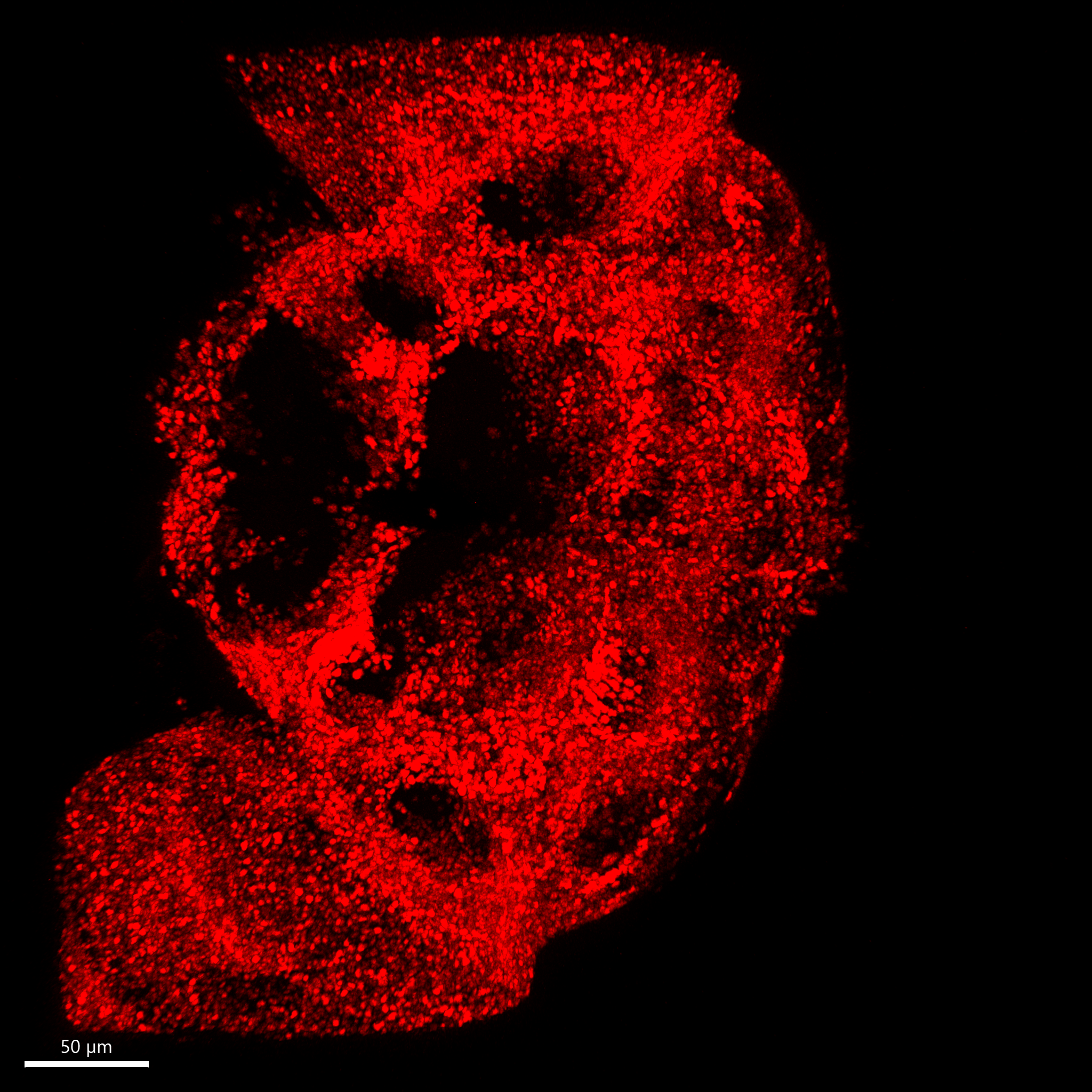

Supplement: Supplementary file 6 — Source data Fig. 3 [file 44319_2025_632_MOESM6_ESM.zip › Figure 3/3A/3A mCherry.tif]

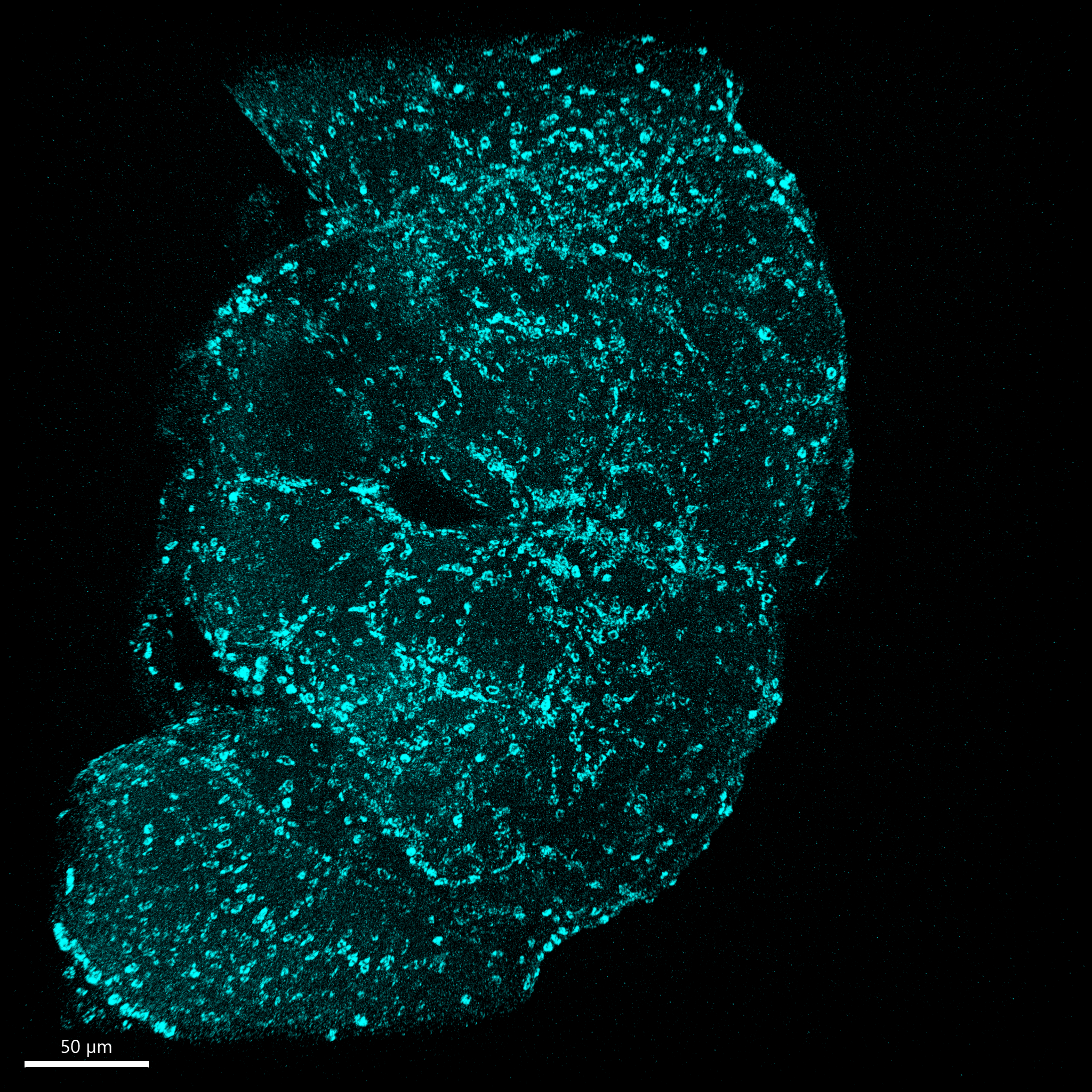

Supplement: Supplementary file 6 — Source data Fig. 3 [file 44319_2025_632_MOESM6_ESM.zip › Figure 3/3A/3A repo.tif]

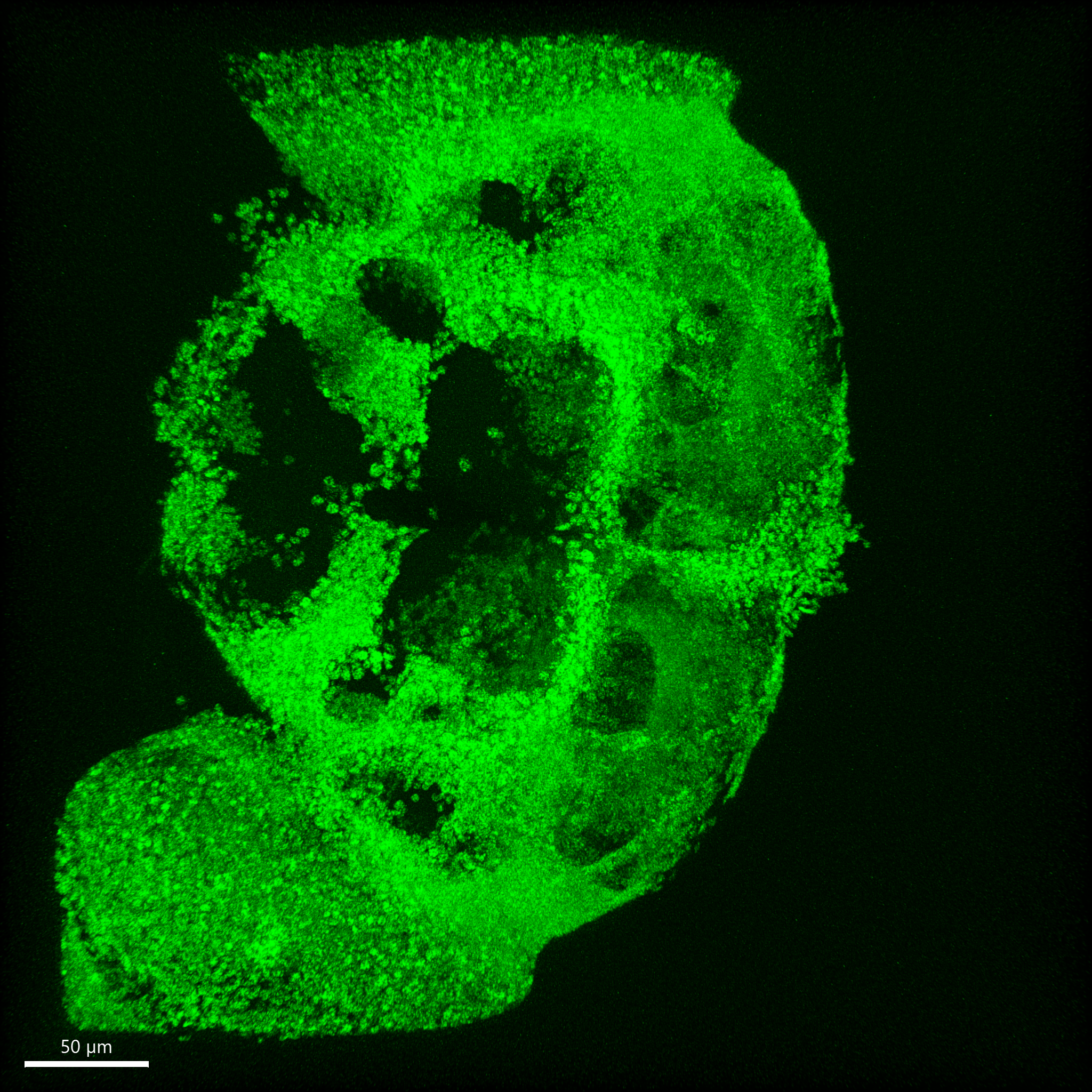

Supplement: Supplementary file 6 — Source data Fig. 3 [file 44319_2025_632_MOESM6_ESM.zip › Figure 3/3A/3A elav.tif]

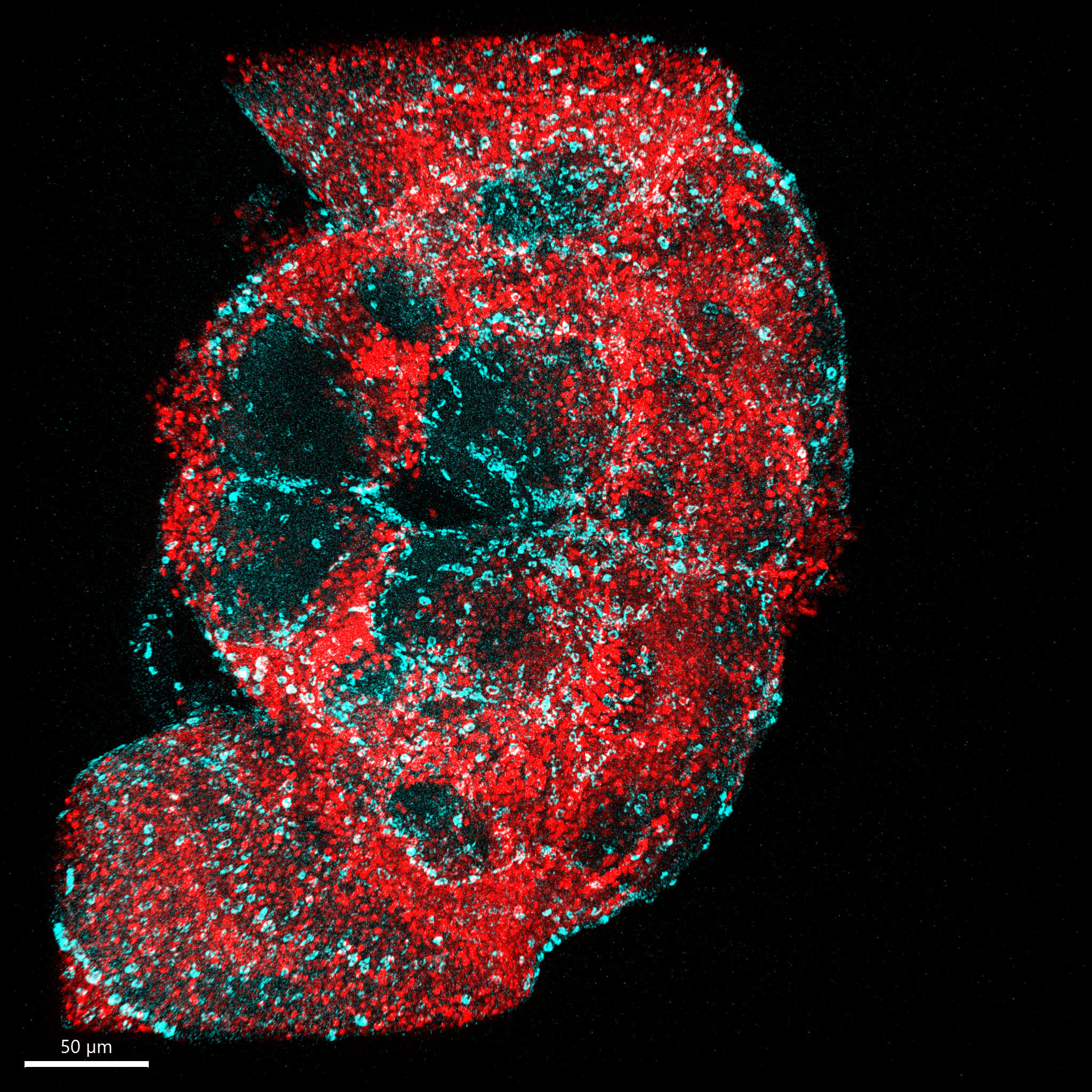

Supplement: Supplementary file 6 — Source data Fig. 3 [file 44319_2025_632_MOESM6_ESM.zip › Figure 3/3A/3A repo+mCherry.tif]

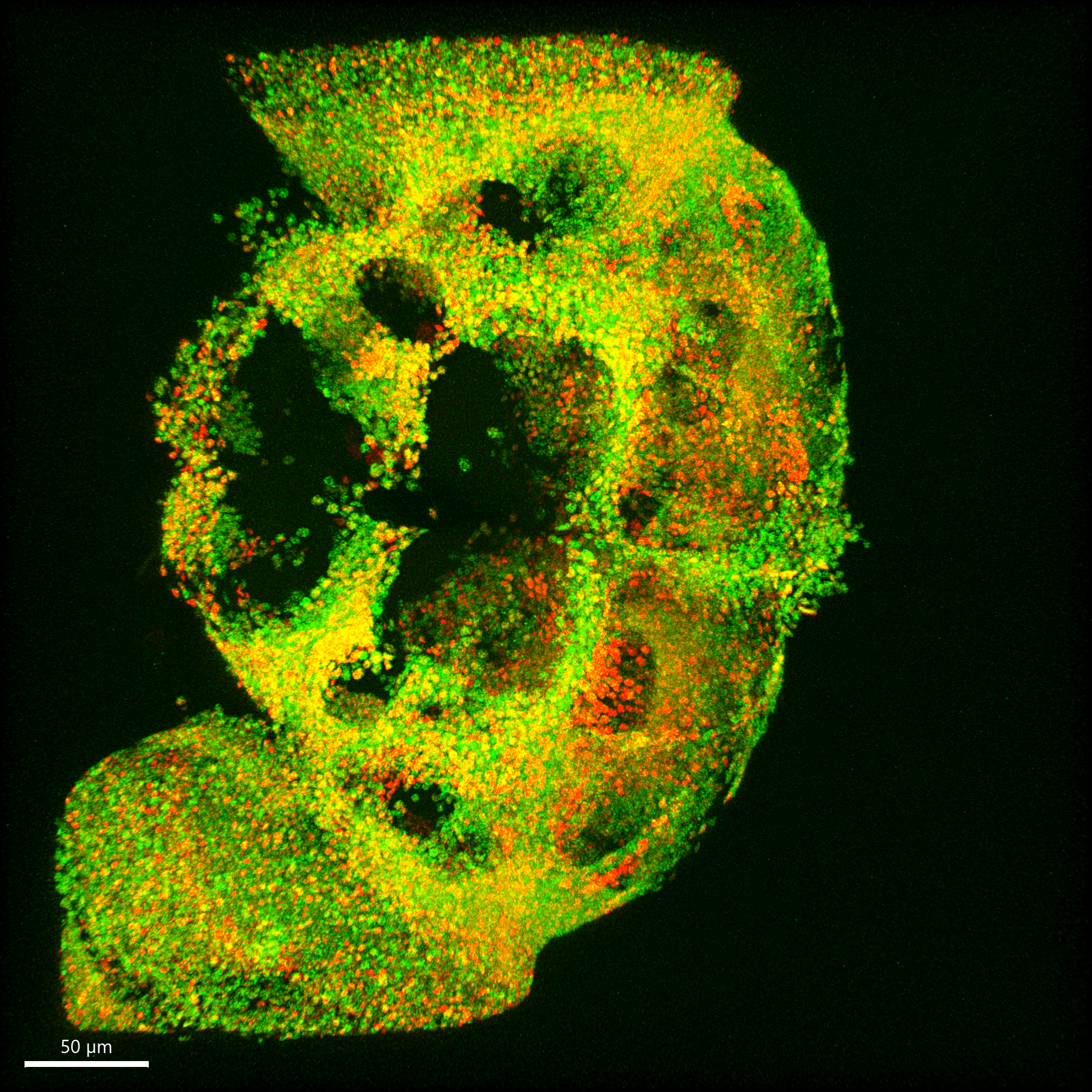

Supplement: Supplementary file 6 — Source data Fig. 3 [file 44319_2025_632_MOESM6_ESM.zip › Figure 3/3A/3A elav+mCherry.tif]

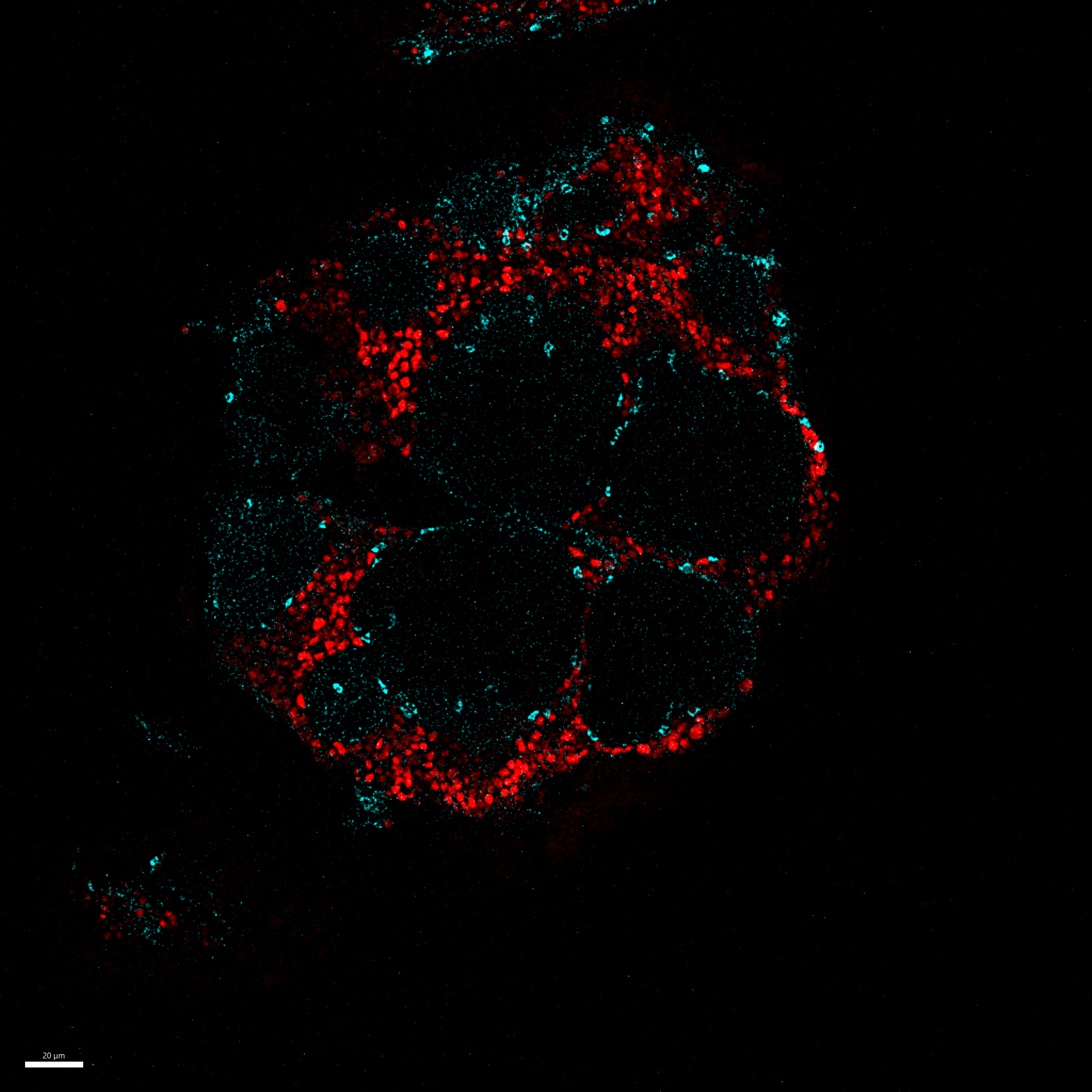

Supplement: Supplementary file 6 — Source data Fig. 3 [file 44319_2025_632_MOESM6_ESM.zip › Figure 3/3A/3A single z repo+mCherry.tif]

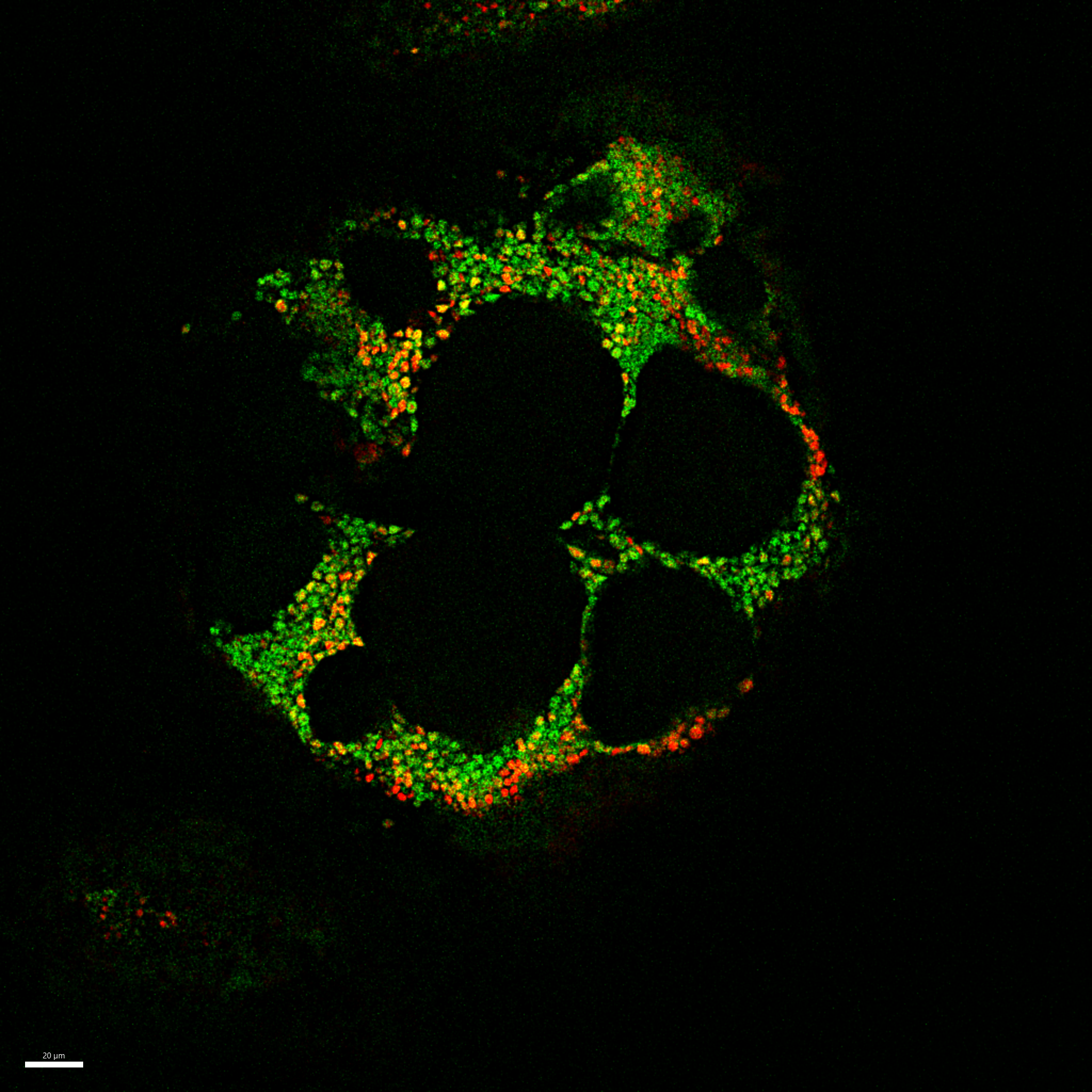

Supplement: Supplementary file 6 — Source data Fig. 3 [file 44319_2025_632_MOESM6_ESM.zip › Figure 3/3A/3A single z elav+mCherry.tif]

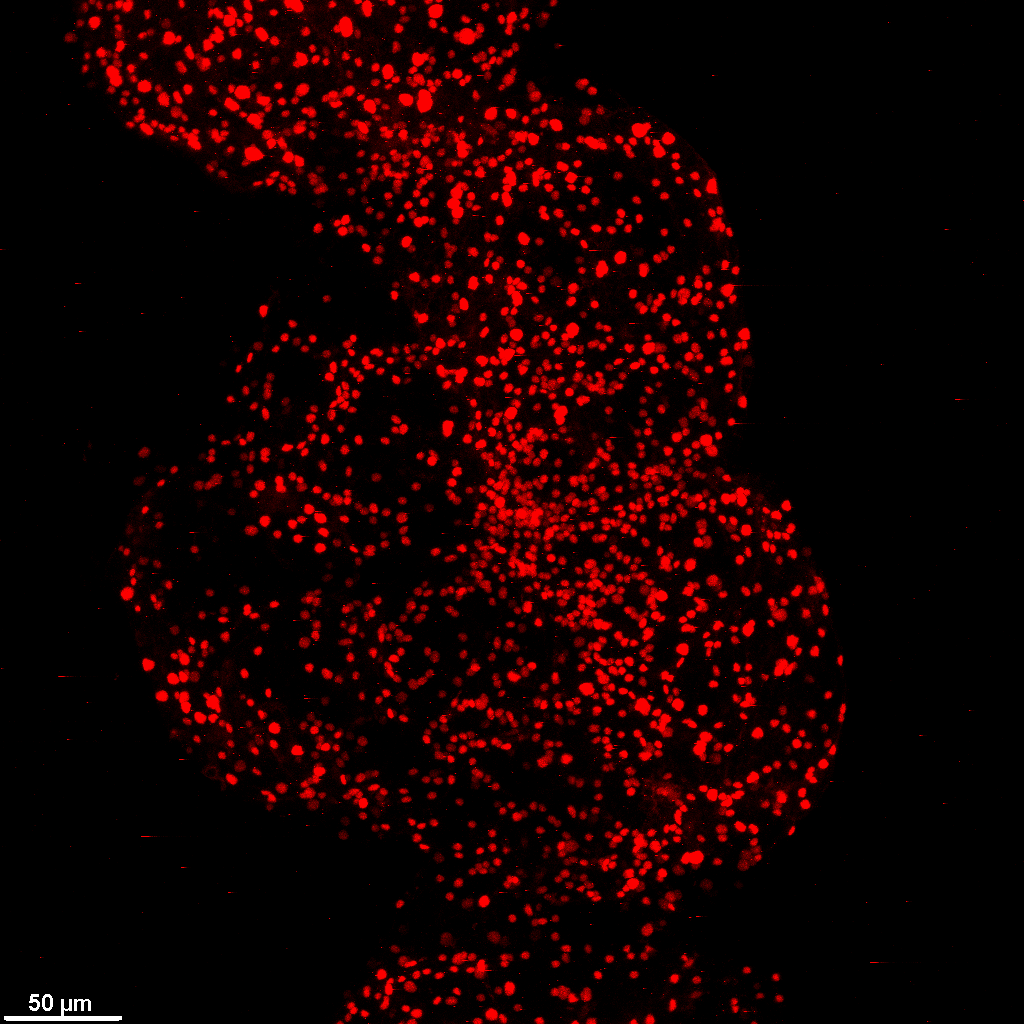

Supplement: Supplementary file 6 — Source data Fig. 3 [file 44319_2025_632_MOESM6_ESM.zip › Figure 3/3B/3B mCherry.tif]

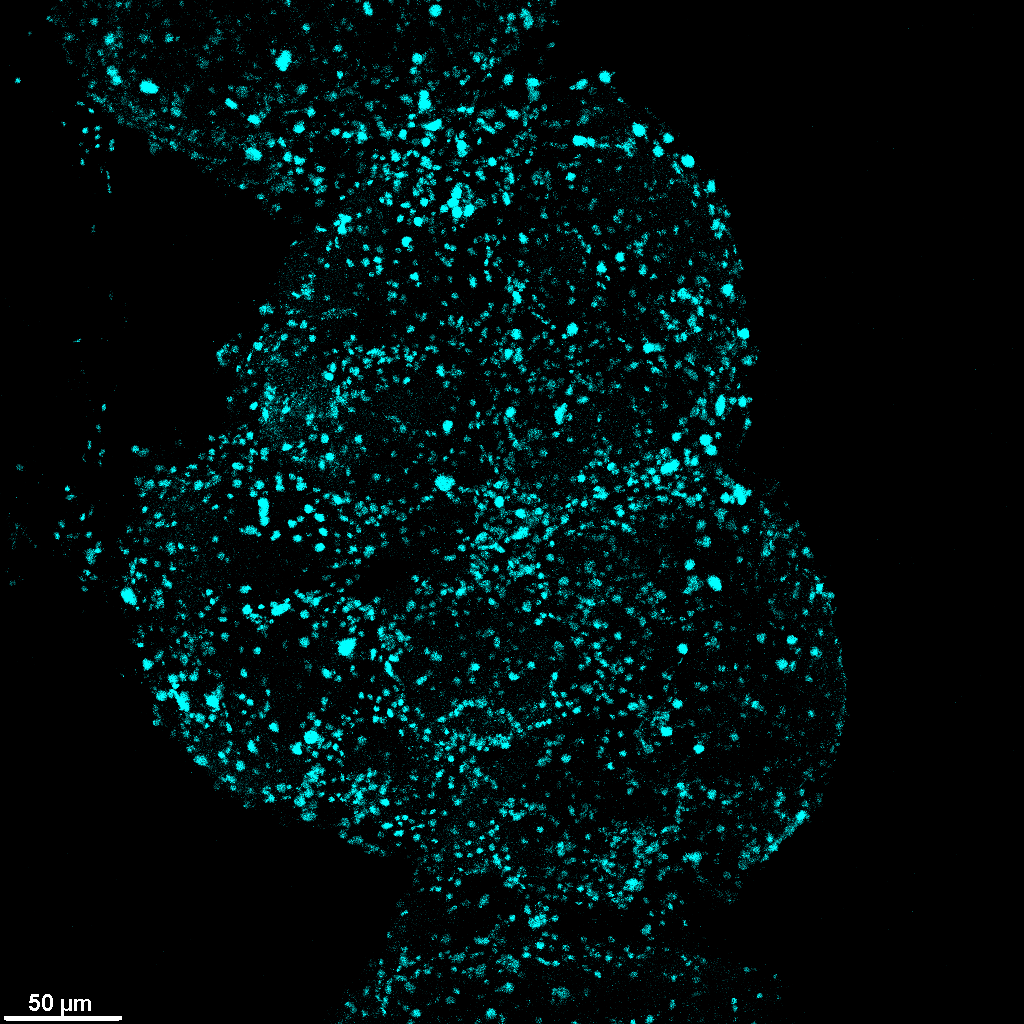

Supplement: Supplementary file 6 — Source data Fig. 3 [file 44319_2025_632_MOESM6_ESM.zip › Figure 3/3B/3B repo.tif]

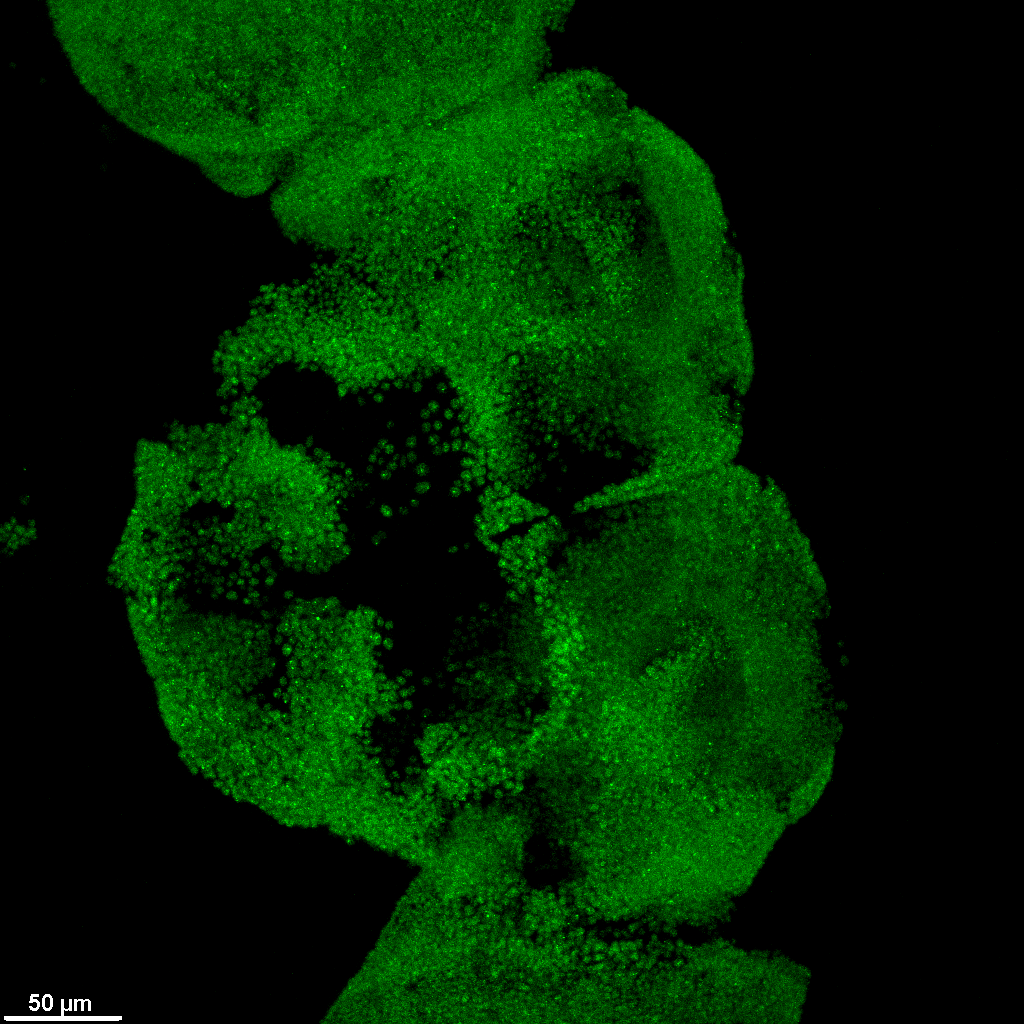

Supplement: Supplementary file 6 — Source data Fig. 3 [file 44319_2025_632_MOESM6_ESM.zip › Figure 3/3B/3B elav.tif]

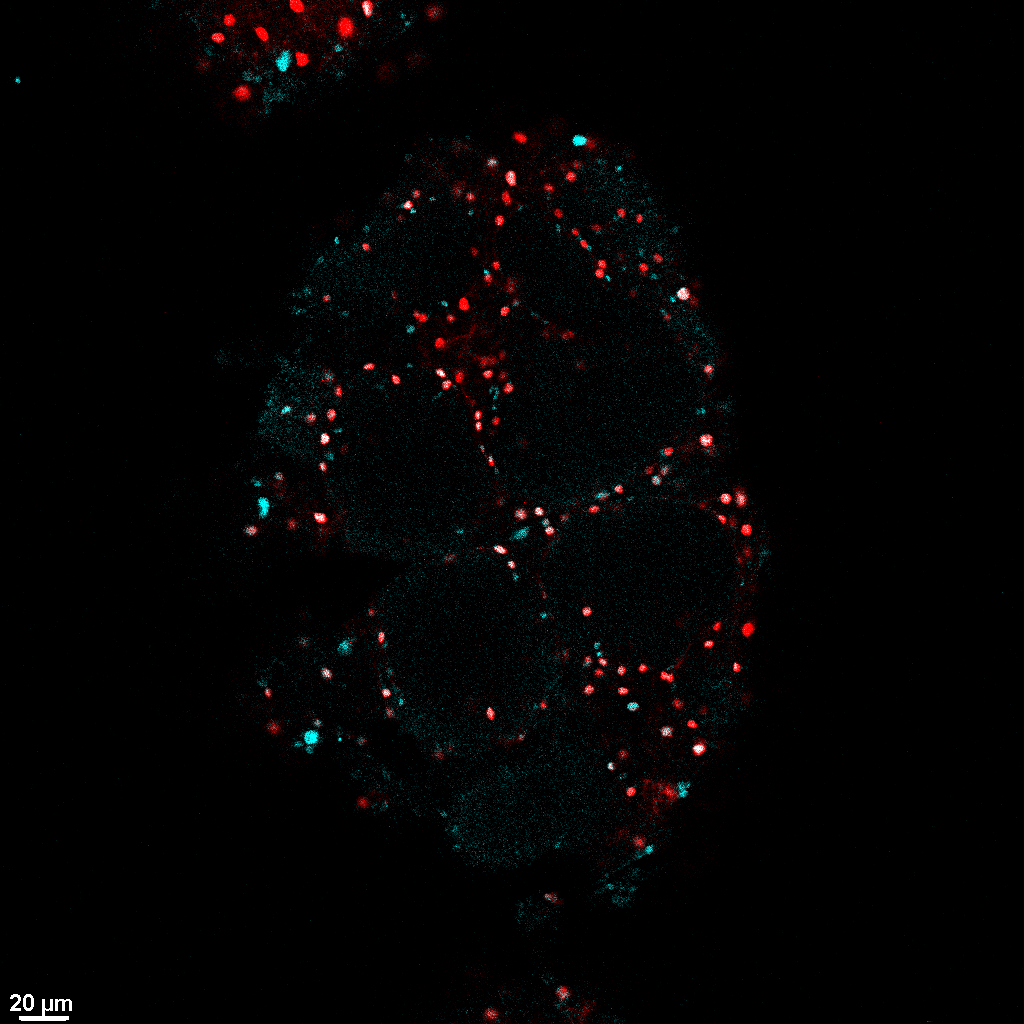

Supplement: Supplementary file 6 — Source data Fig. 3 [file 44319_2025_632_MOESM6_ESM.zip › Figure 3/3B/3B repo+mCherry.tif]

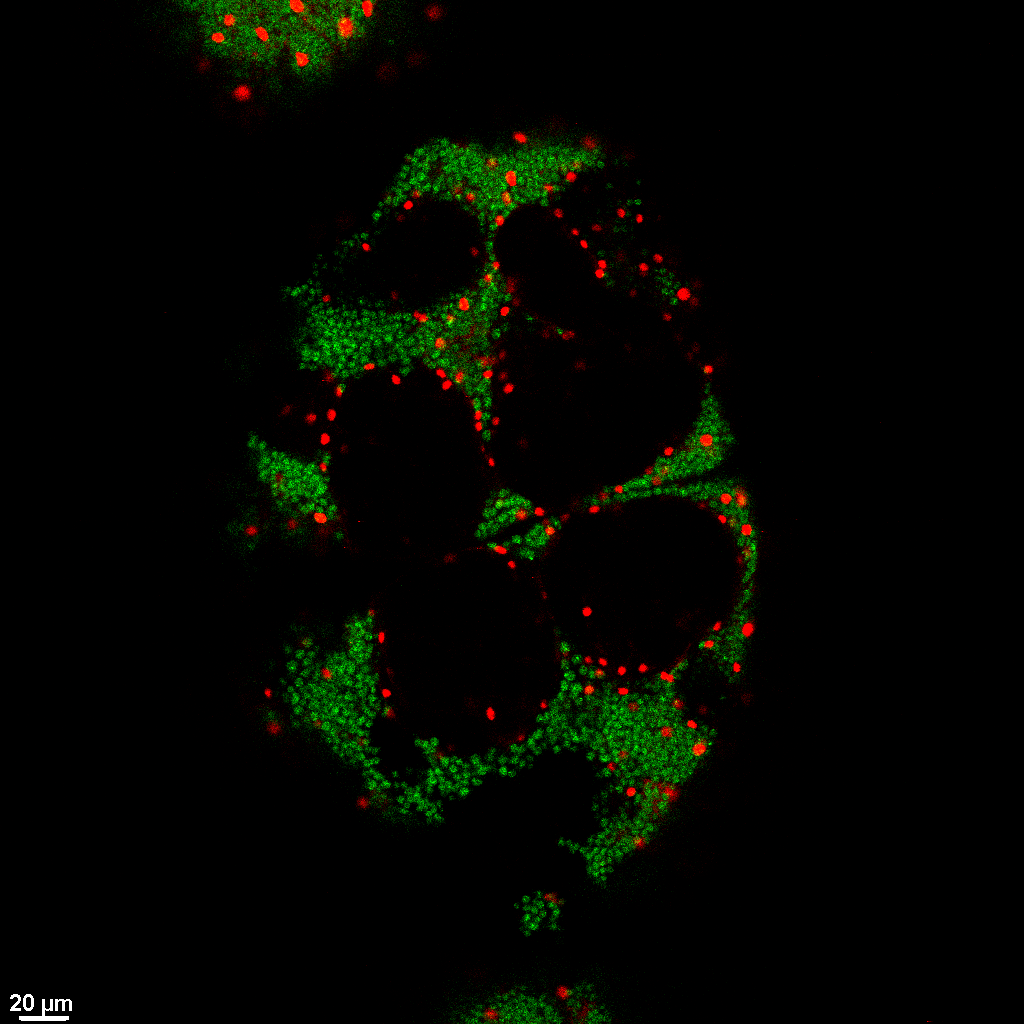

Supplement: Supplementary file 6 — Source data Fig. 3 [file 44319_2025_632_MOESM6_ESM.zip › Figure 3/3B/3B elav+mCherry.tif]

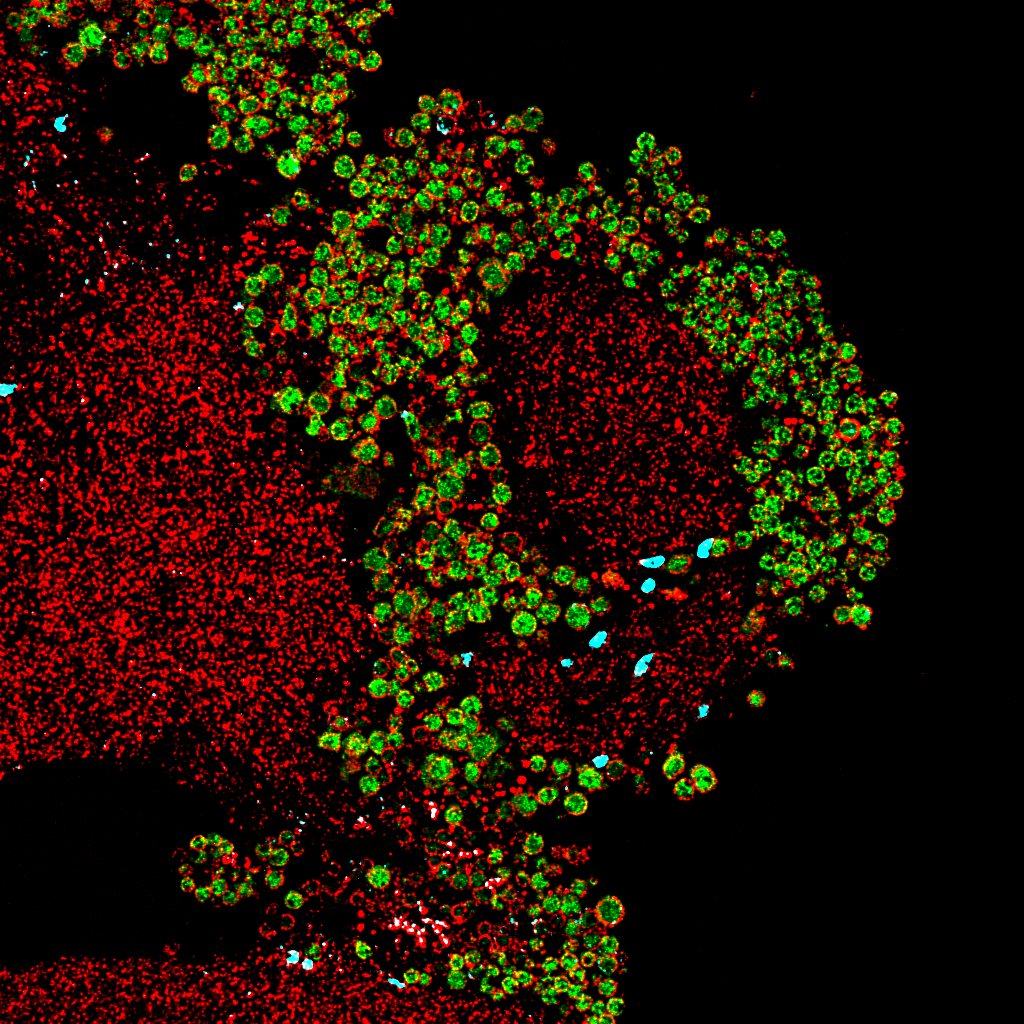

Supplement: Supplementary file 7 — Source data Fig. 4 [file 44319_2025_632_MOESM7_ESM.zip › Figure 4/4A/4A single z Merge.tif]

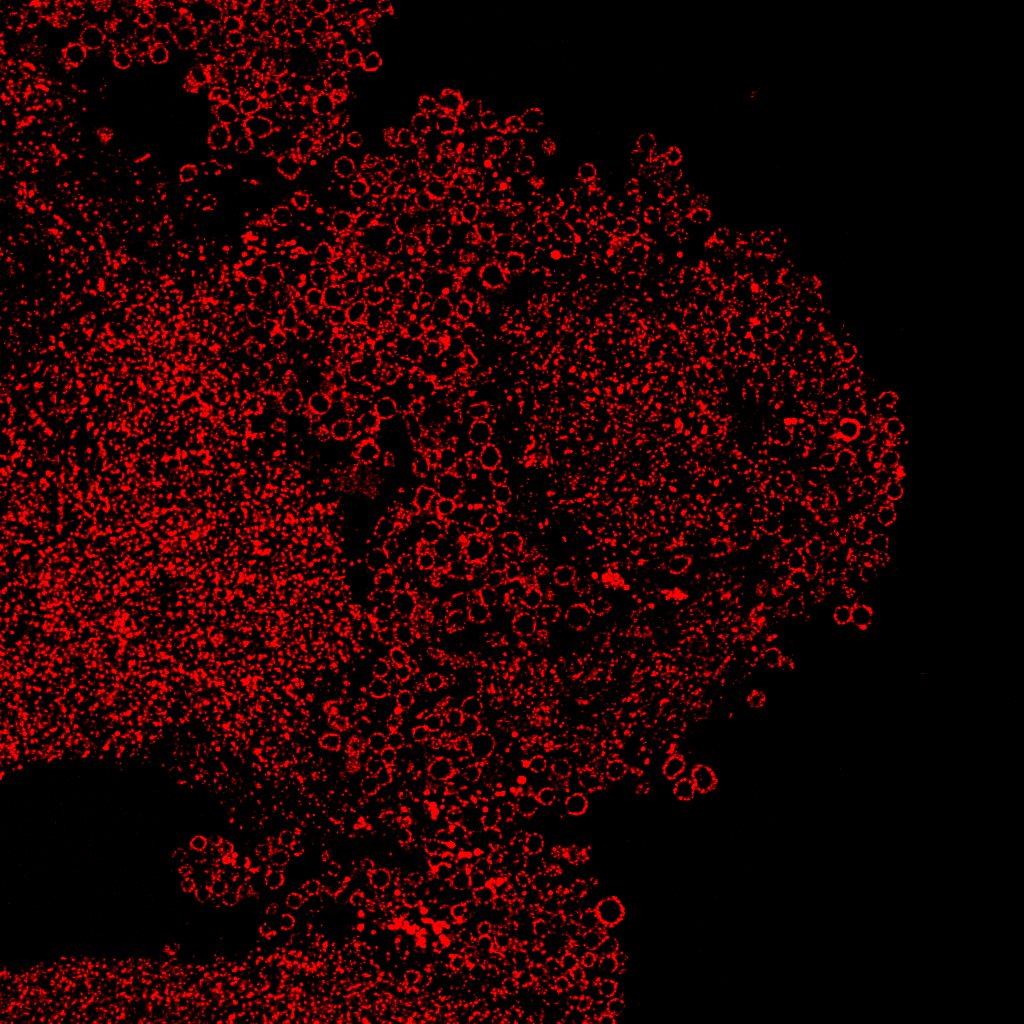

Supplement: Supplementary file 7 — Source data Fig. 4 [file 44319_2025_632_MOESM7_ESM.zip › Figure 4/4A/4A zoom-in HA.tif]

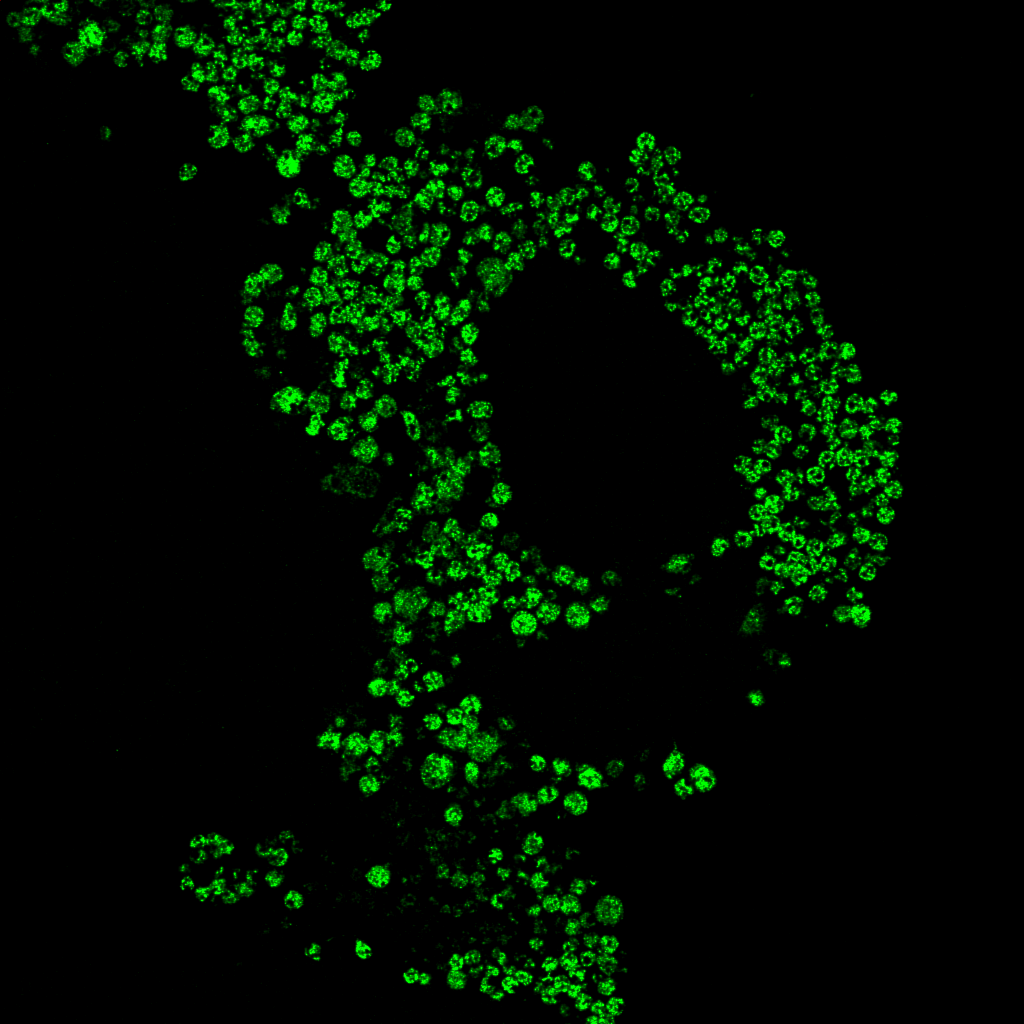

Supplement: Supplementary file 7 — Source data Fig. 4 [file 44319_2025_632_MOESM7_ESM.zip › Figure 4/4A/4A zoom-in elav.tif]

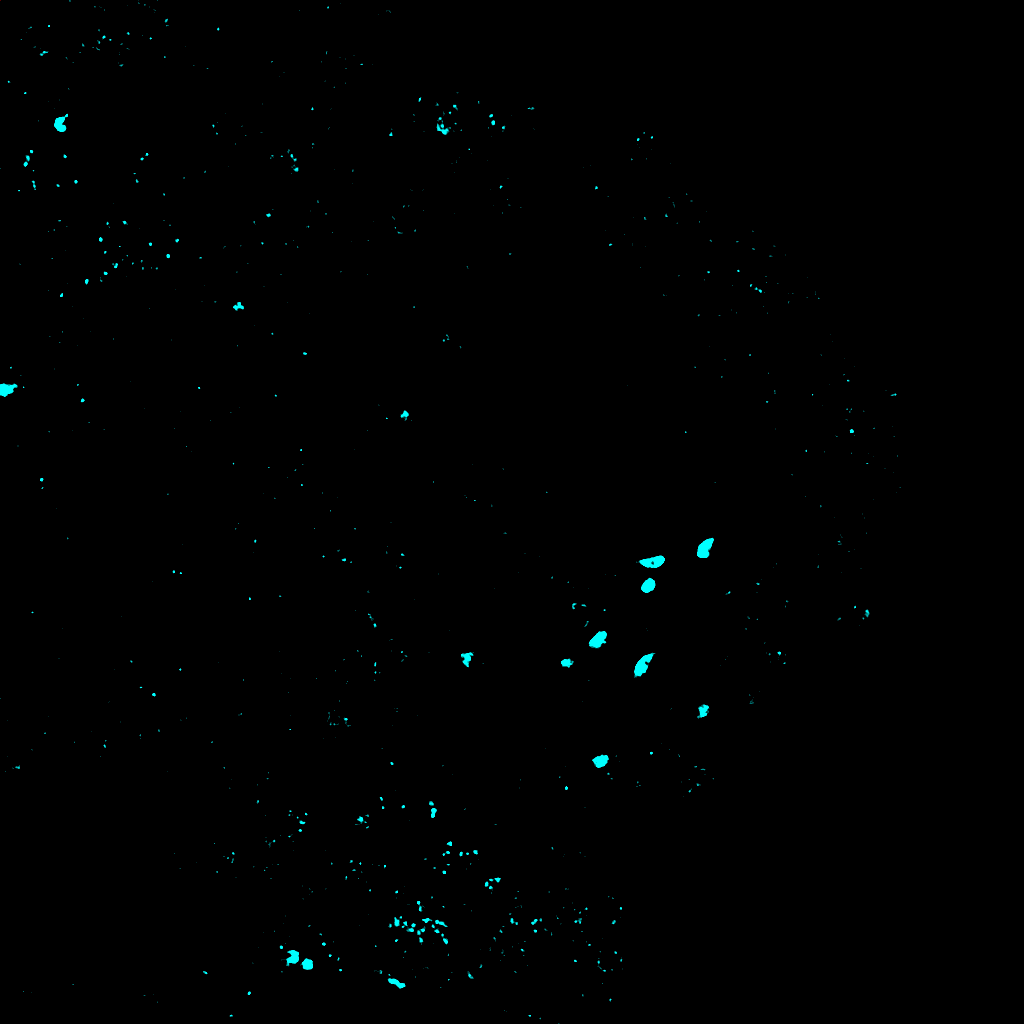

Supplement: Supplementary file 7 — Source data Fig. 4 [file 44319_2025_632_MOESM7_ESM.zip › Figure 4/4A/4A zoom-in repo.tif]

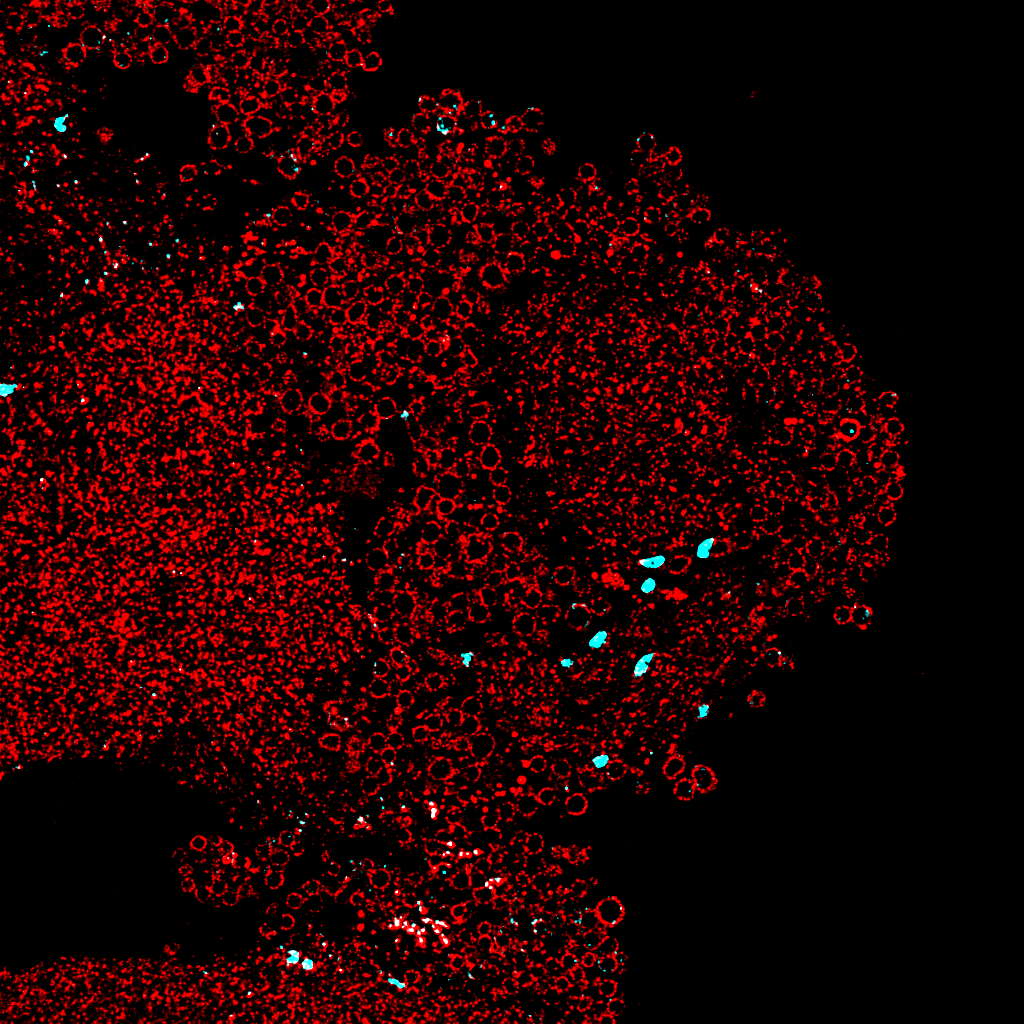

Supplement: Supplementary file 7 — Source data Fig. 4 [file 44319_2025_632_MOESM7_ESM.zip › Figure 4/4A/4A zoom-in HA+repo.tif]

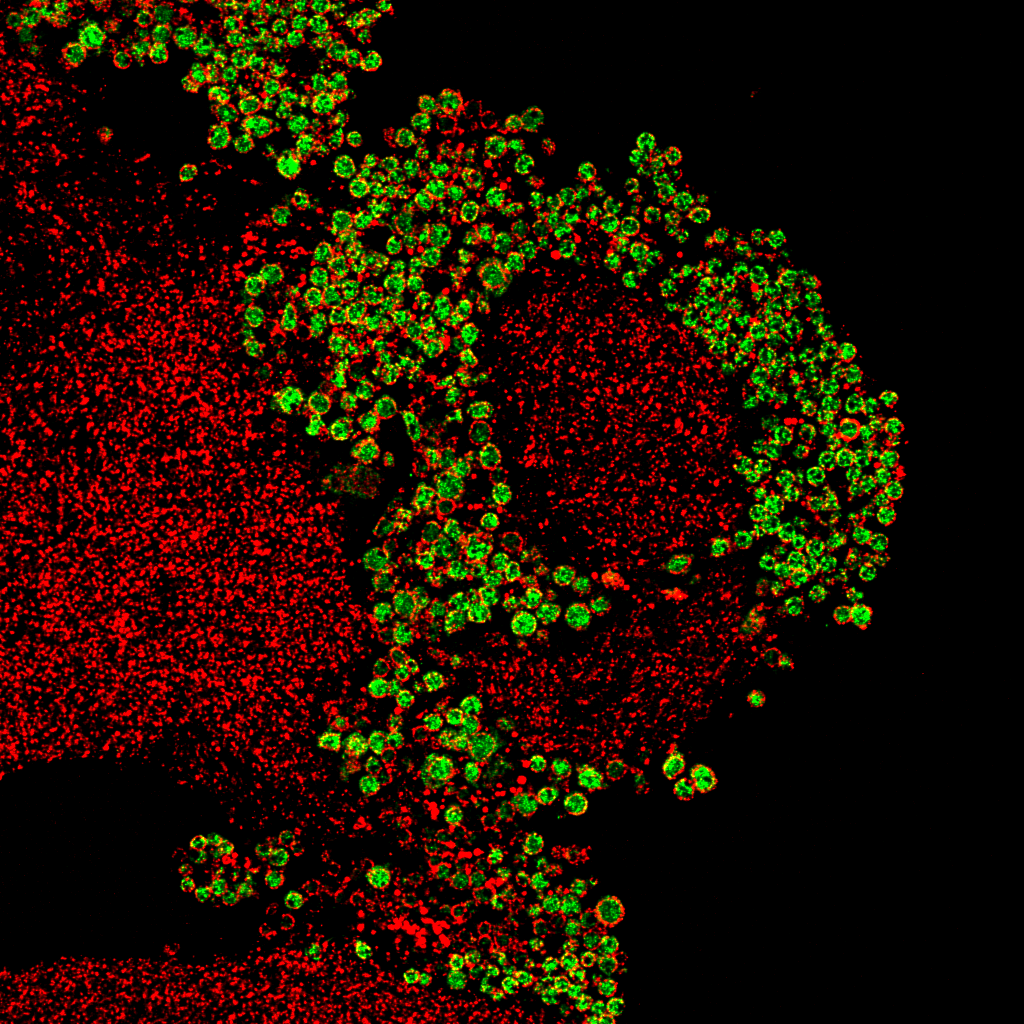

Supplement: Supplementary file 7 — Source data Fig. 4 [file 44319_2025_632_MOESM7_ESM.zip › Figure 4/4A/4A zoom-in HA+elav.tif]

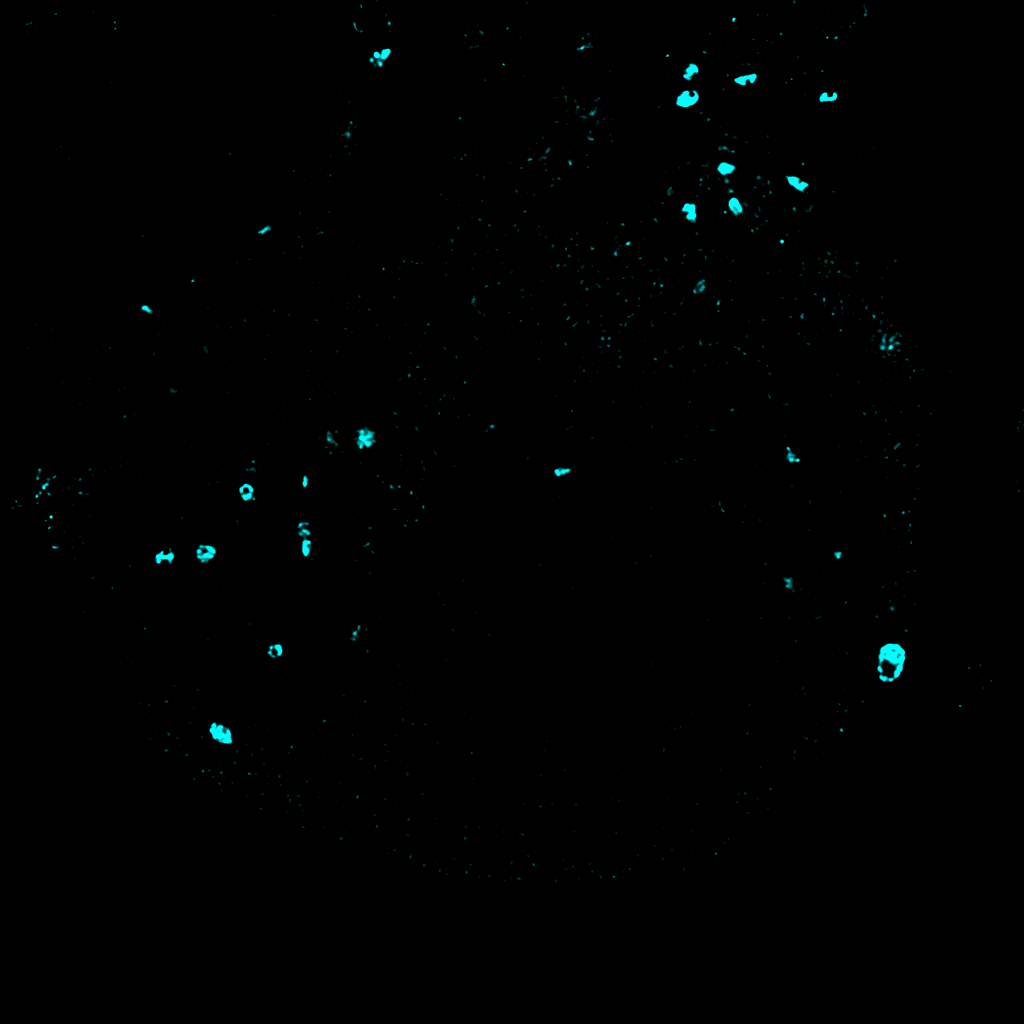

Supplement: Supplementary file 7 — Source data Fig. 4 [file 44319_2025_632_MOESM7_ESM.zip › Figure 4/4B/4B zoom-in repo.tif]

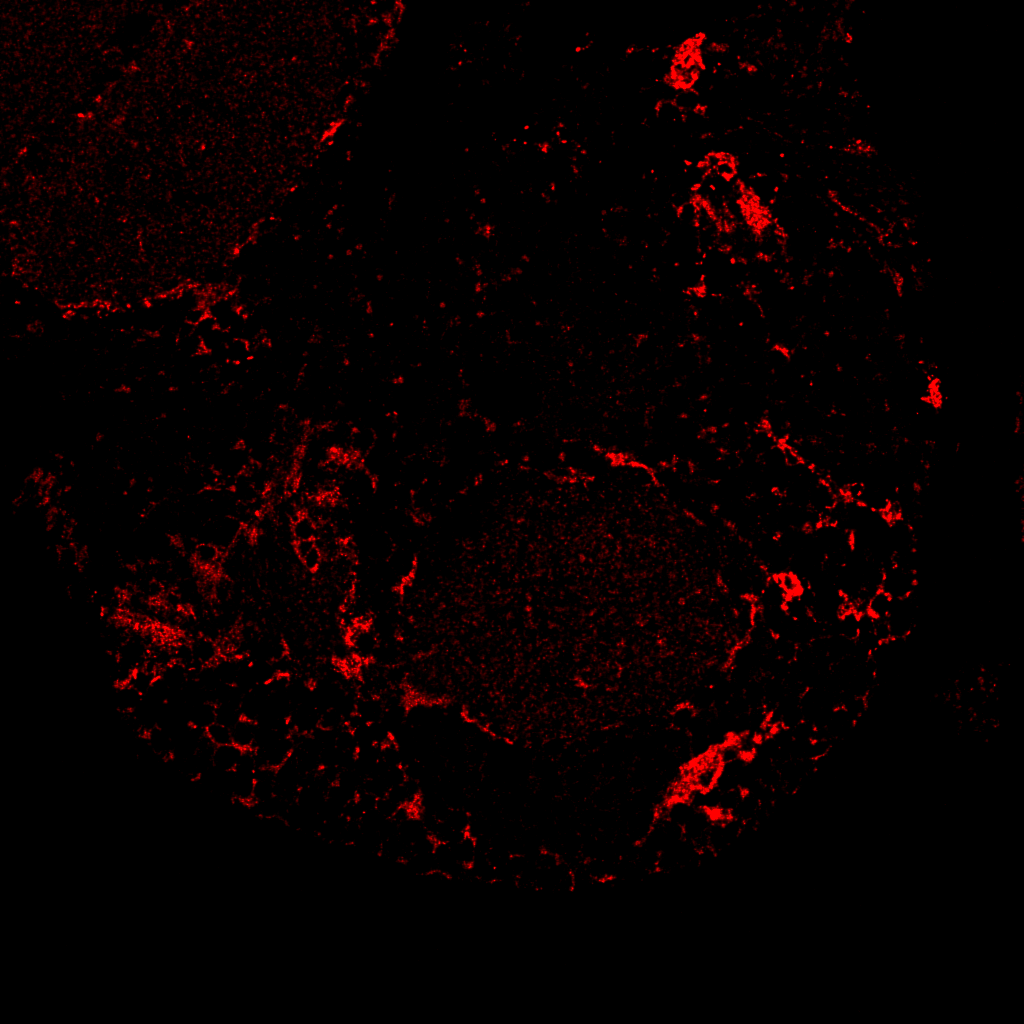

Supplement: Supplementary file 7 — Source data Fig. 4 [file 44319_2025_632_MOESM7_ESM.zip › Figure 4/4B/4B zoom-in HA.tif]

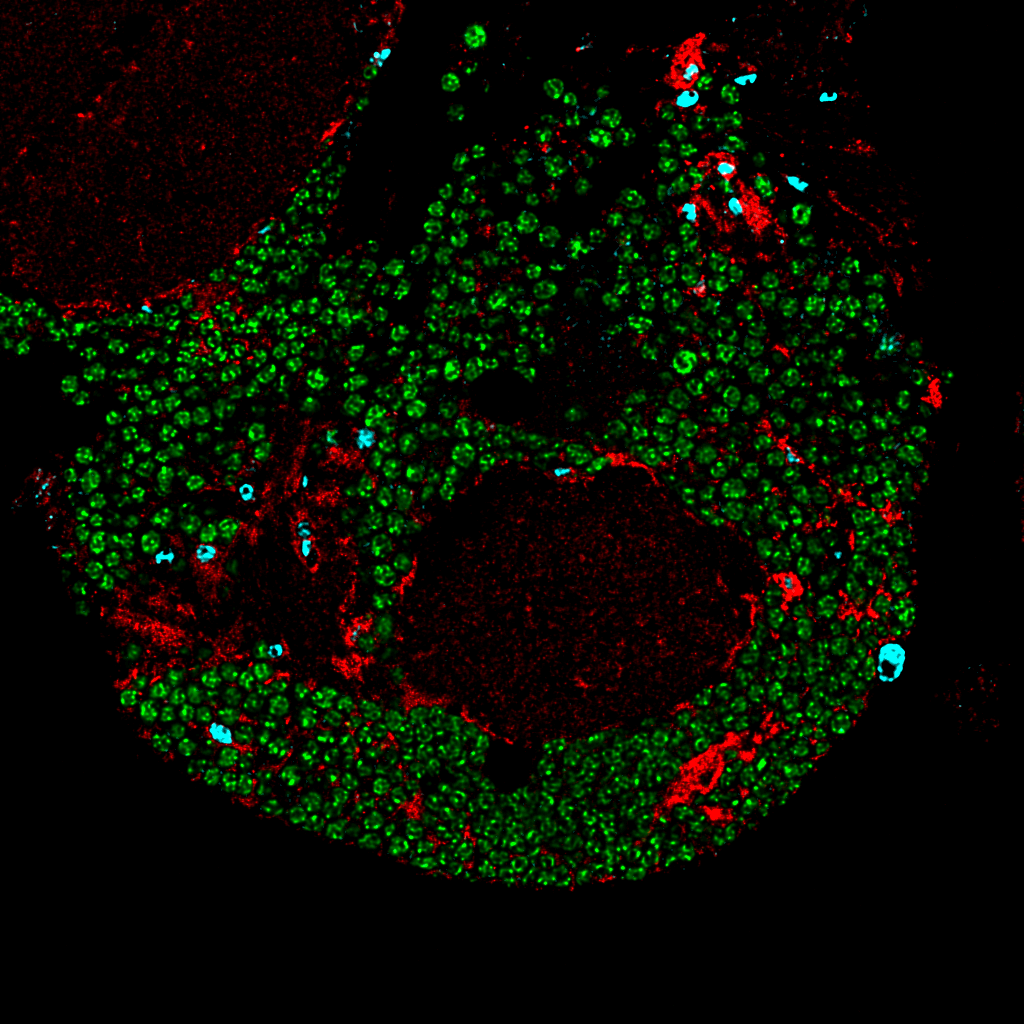

Supplement: Supplementary file 7 — Source data Fig. 4 [file 44319_2025_632_MOESM7_ESM.zip › Figure 4/4B/4B single z Merge .tif]

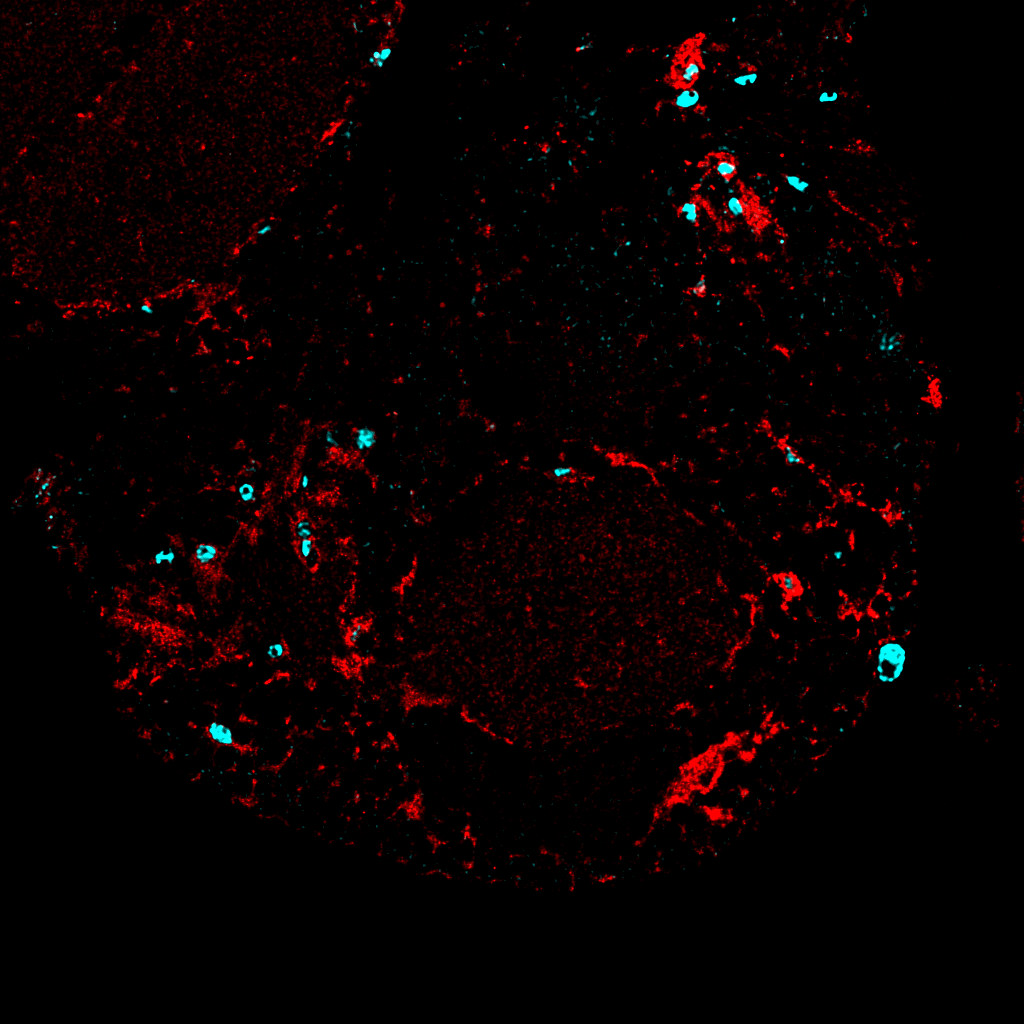

Supplement: Supplementary file 7 — Source data Fig. 4 [file 44319_2025_632_MOESM7_ESM.zip › Figure 4/4B/4B zoom-in HA+repo.tif]

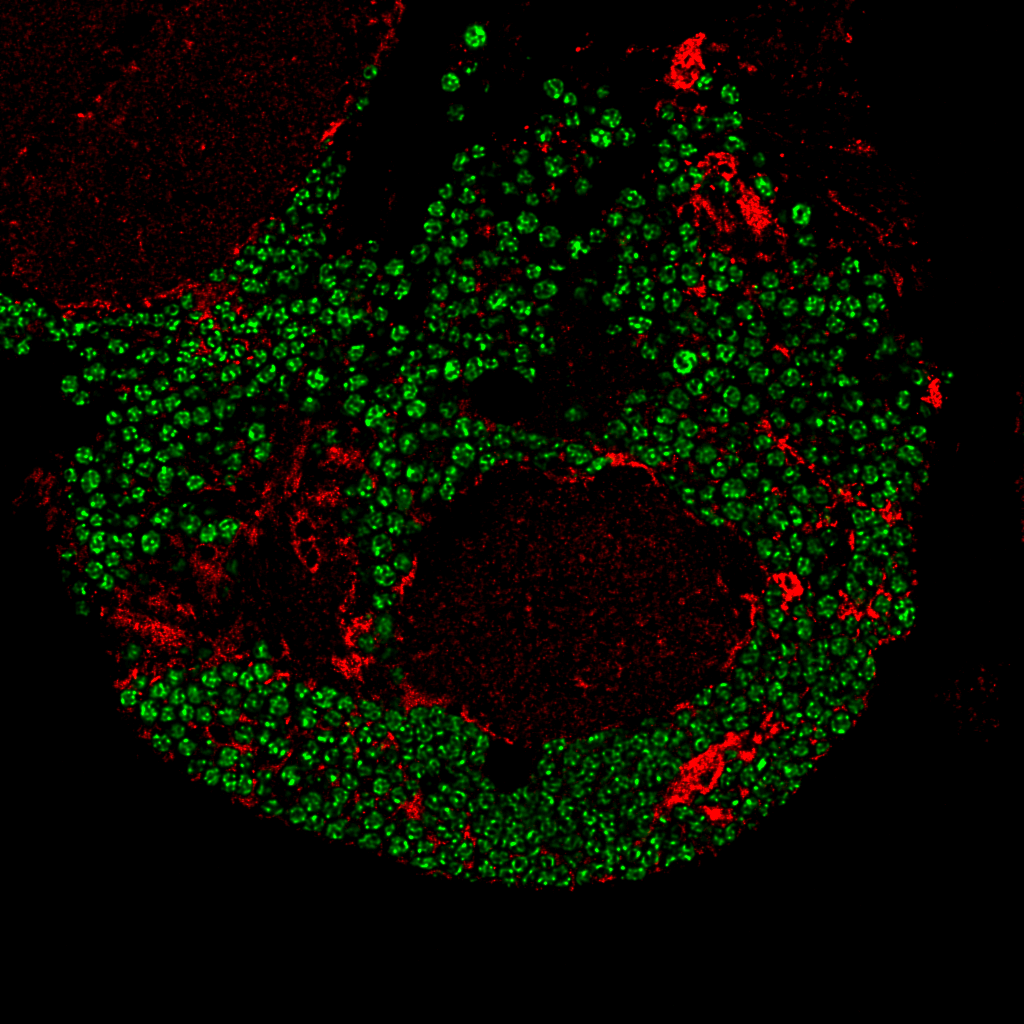

Supplement: Supplementary file 7 — Source data Fig. 4 [file 44319_2025_632_MOESM7_ESM.zip › Figure 4/4B/4B zoom-in HA+elav.tif]

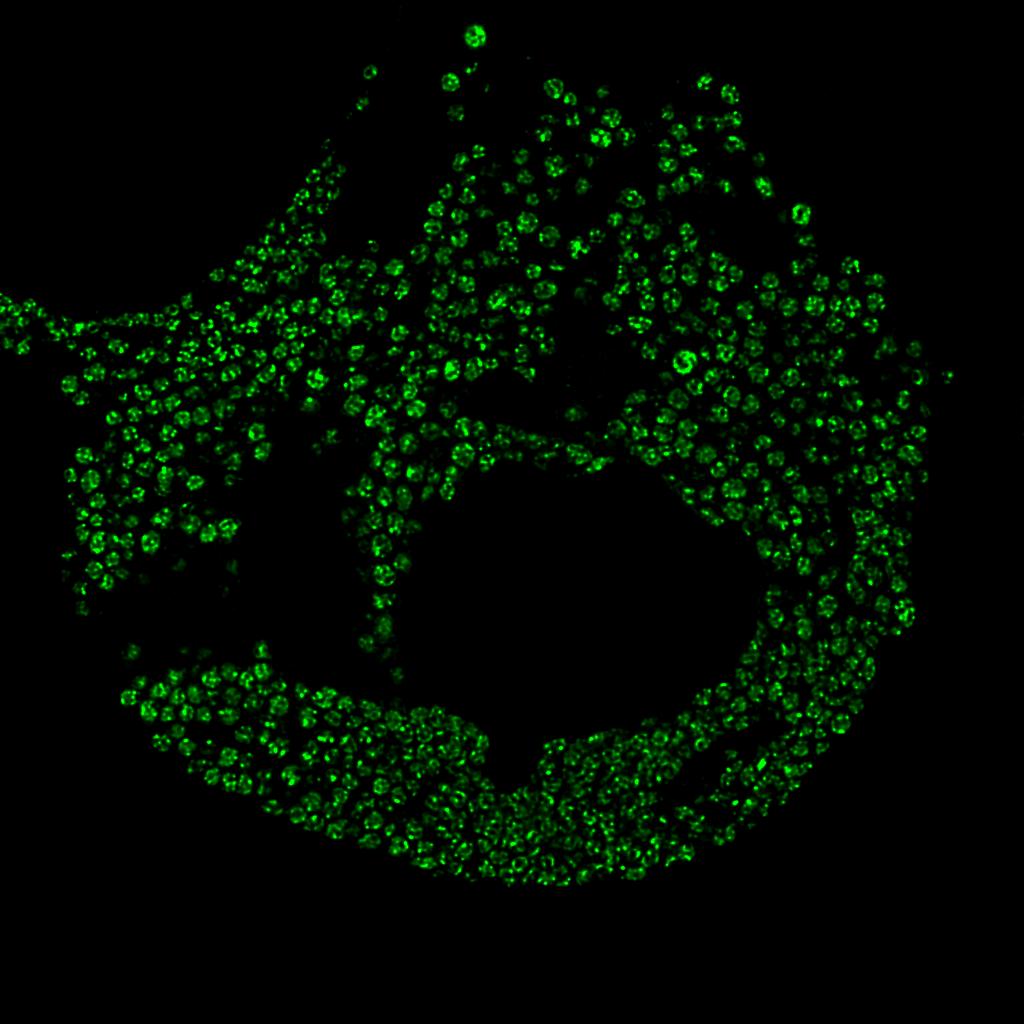

Supplement: Supplementary file 7 — Source data Fig. 4 [file 44319_2025_632_MOESM7_ESM.zip › Figure 4/4B/4B zoom-in elav.tif]

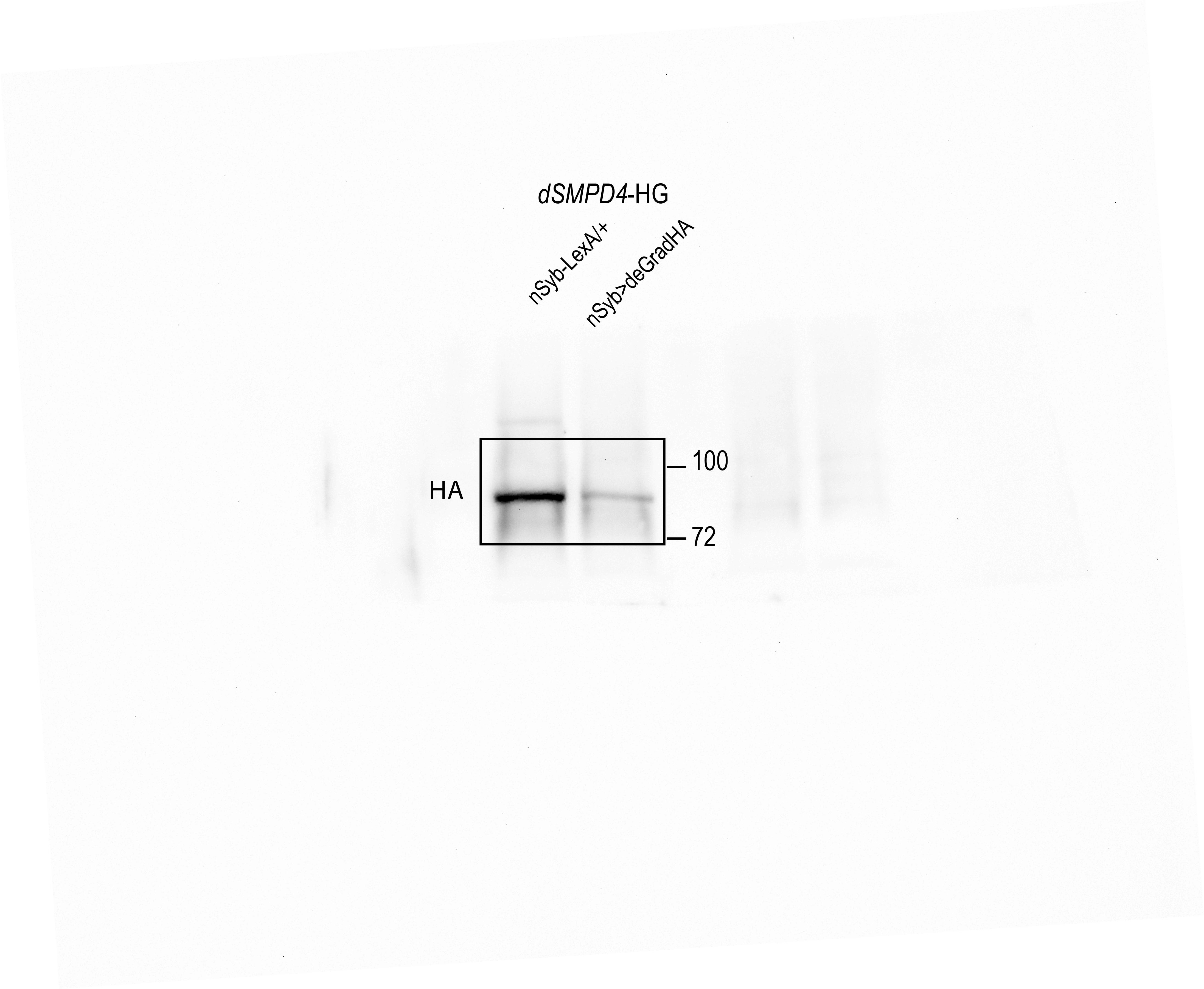

Supplement: Supplementary file 8 — Source data Fig. 5 [file 44319_2025_632_MOESM8_ESM.zip › Figure 5/5C/5C HA.jpg]

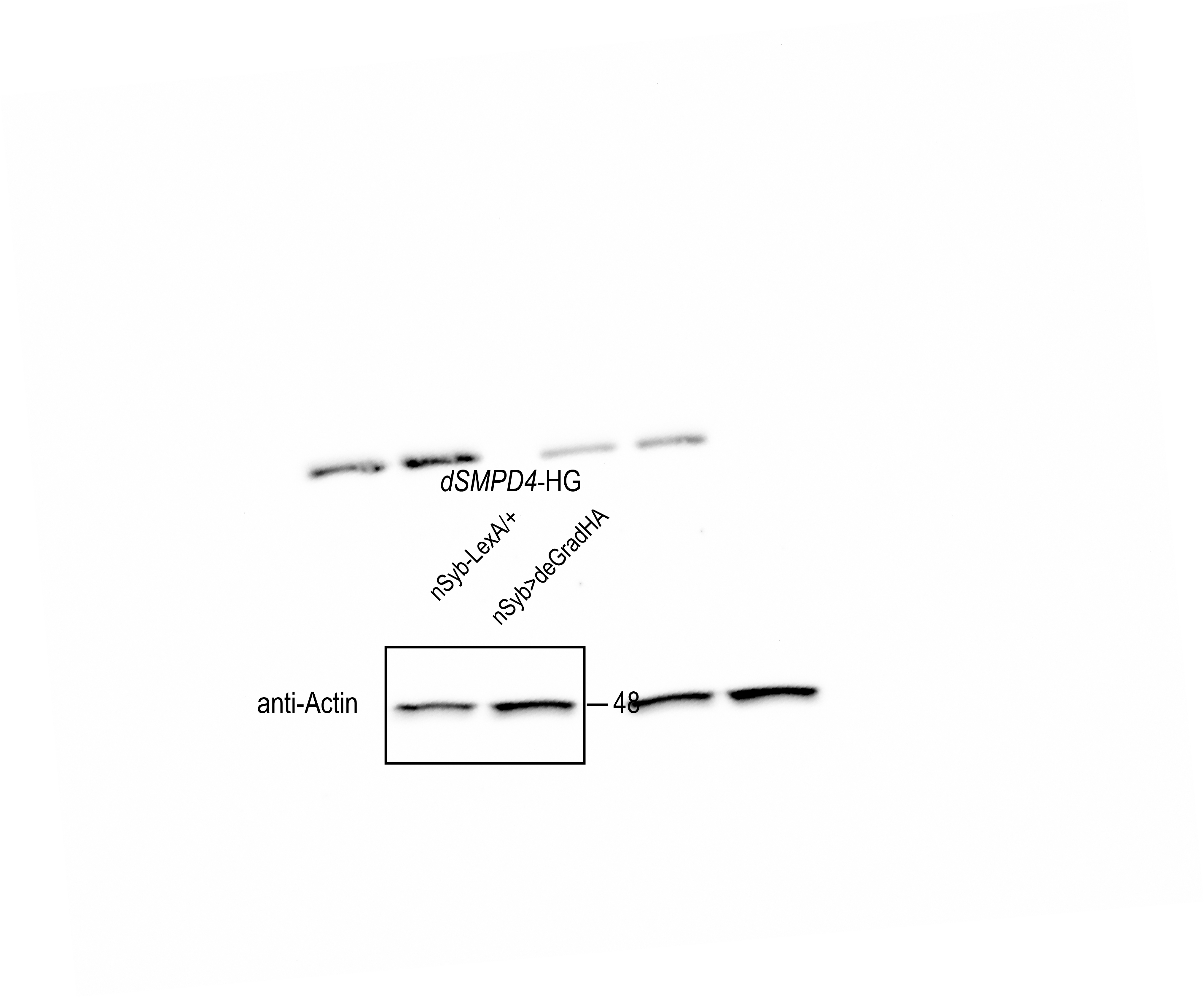

Supplement: Supplementary file 8 — Source data Fig. 5 [file 44319_2025_632_MOESM8_ESM.zip › Figure 5/5C/5C Actin.jpg]

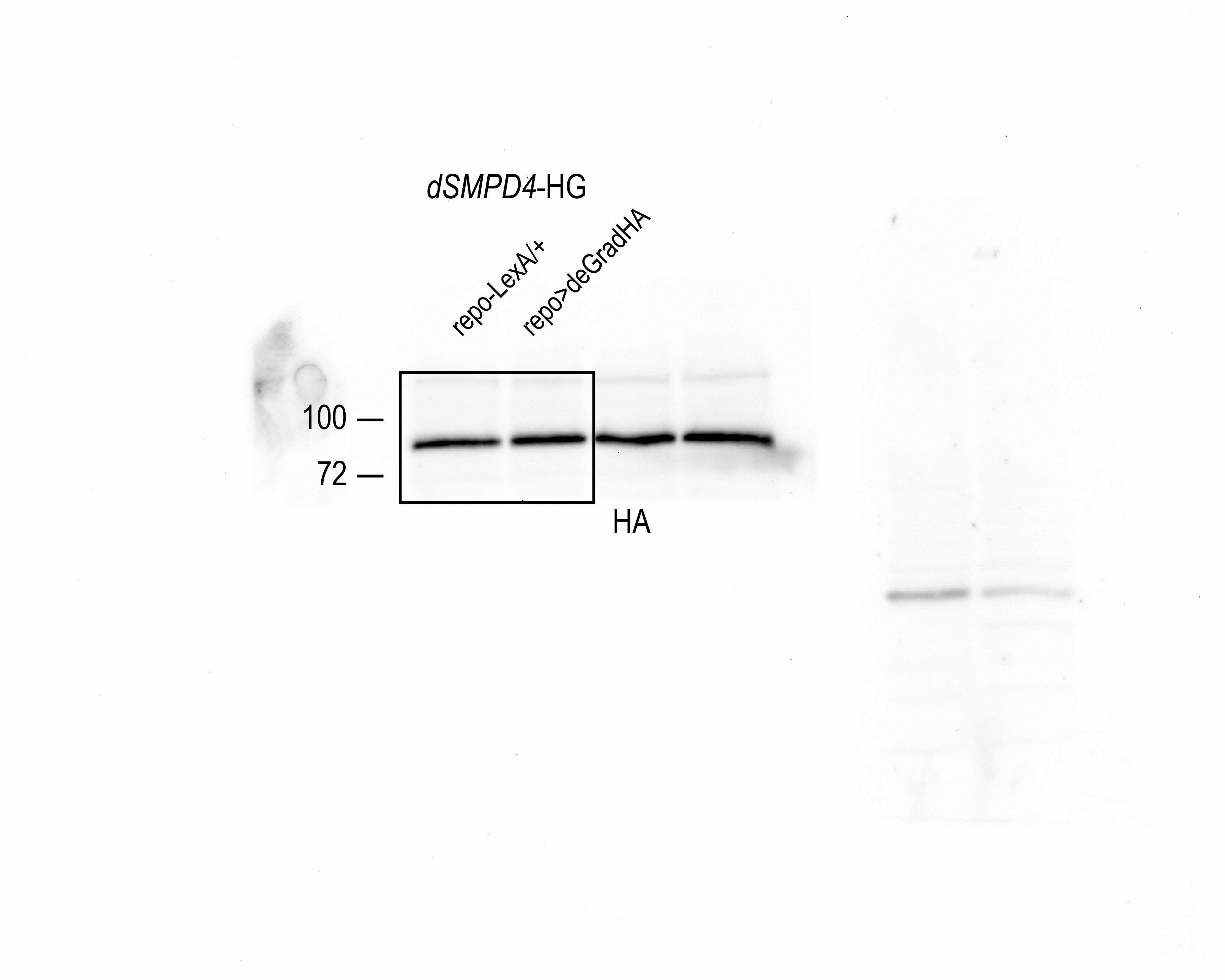

Supplement: Supplementary file 8 — Source data Fig. 5 [file 44319_2025_632_MOESM8_ESM.zip › Figure 5/5D/5D HA.jpg]

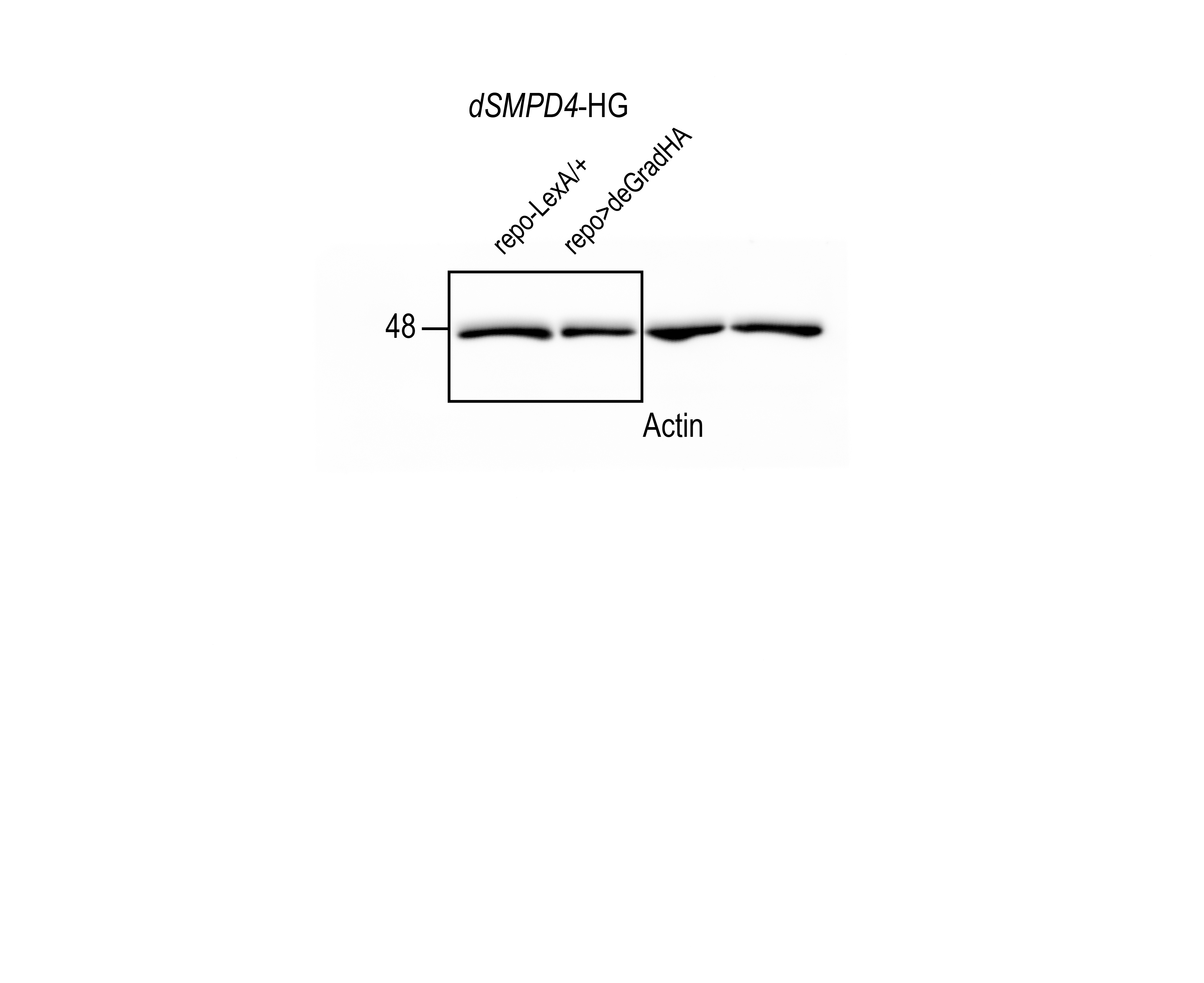

Supplement: Supplementary file 8 — Source data Fig. 5 [file 44319_2025_632_MOESM8_ESM.zip › Figure 5/5D/5D Actin.jpg]

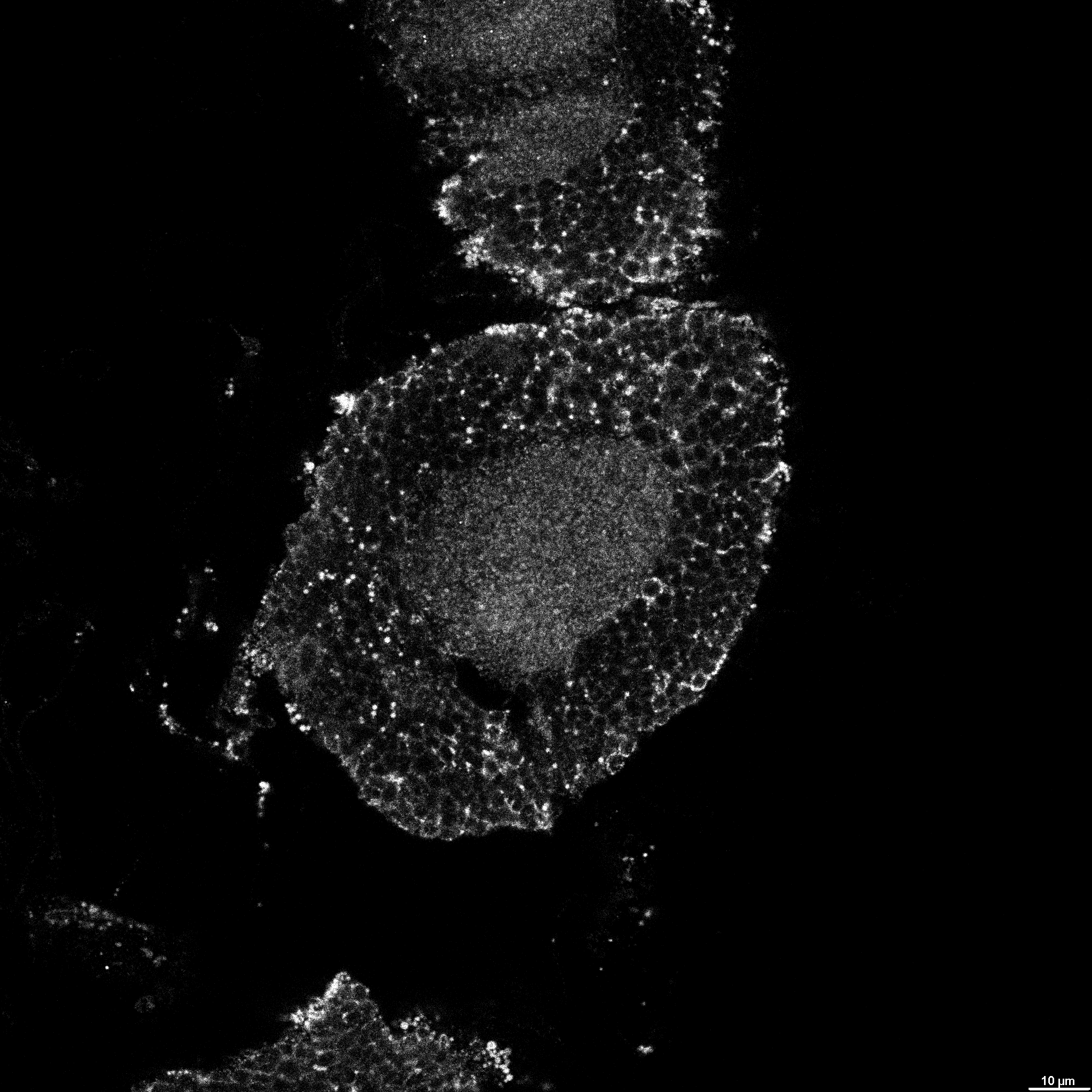

Supplement: Supplementary file 8 — Source data Fig. 5 [file 44319_2025_632_MOESM8_ESM.zip › Figure 5/5E/(bot)nSyb-LexA LexAop-deGradHA plus HA.tif]

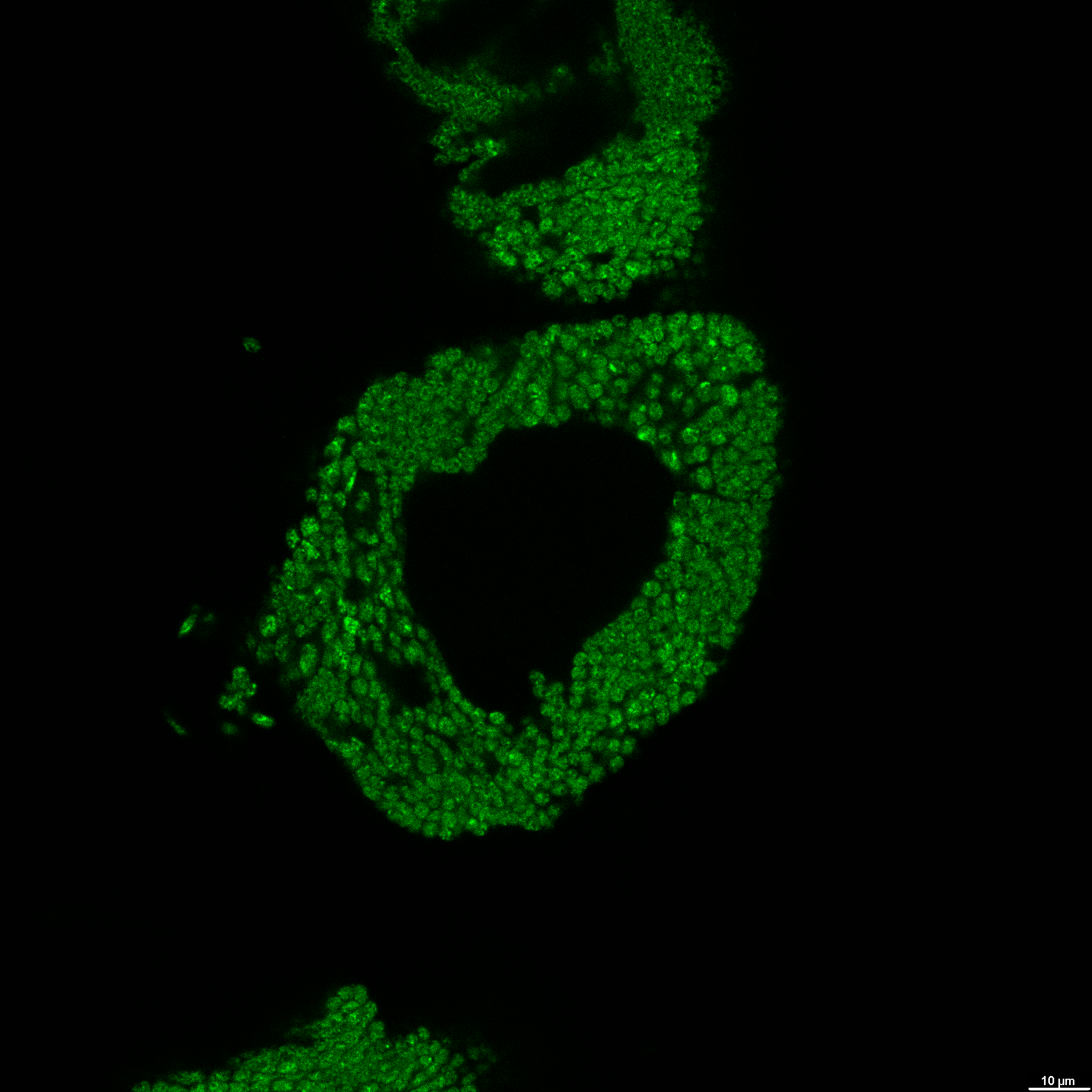

Supplement: Supplementary file 8 — Source data Fig. 5 [file 44319_2025_632_MOESM8_ESM.zip › Figure 5/5E/(bot)nSyb-LexA LexAop-deGradHA elav.tif]

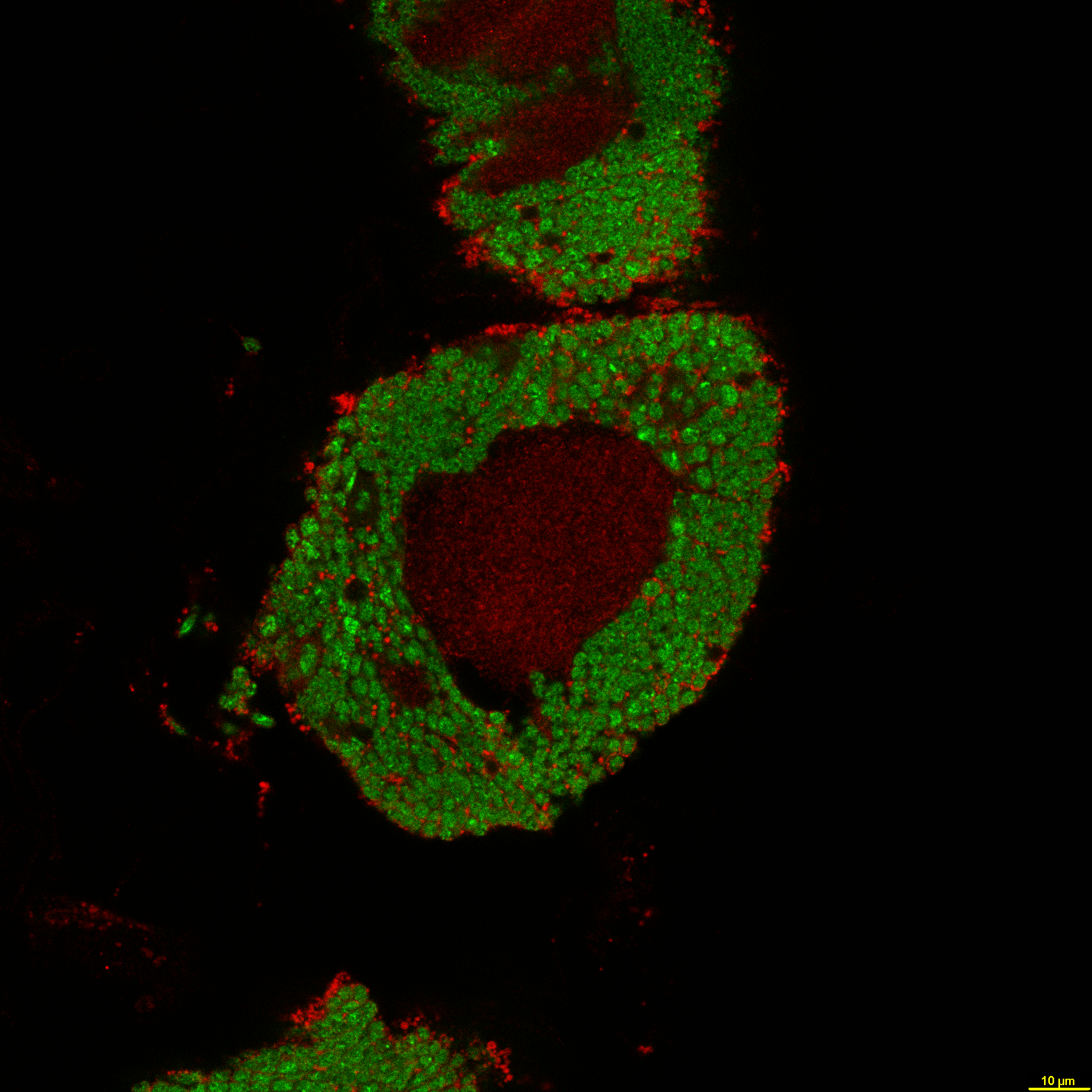

Supplement: Supplementary file 8 — Source data Fig. 5 [file 44319_2025_632_MOESM8_ESM.zip › Figure 5/5E/(bot)nSyb-LexA LexAop-deGradHA elav+HA.tif]

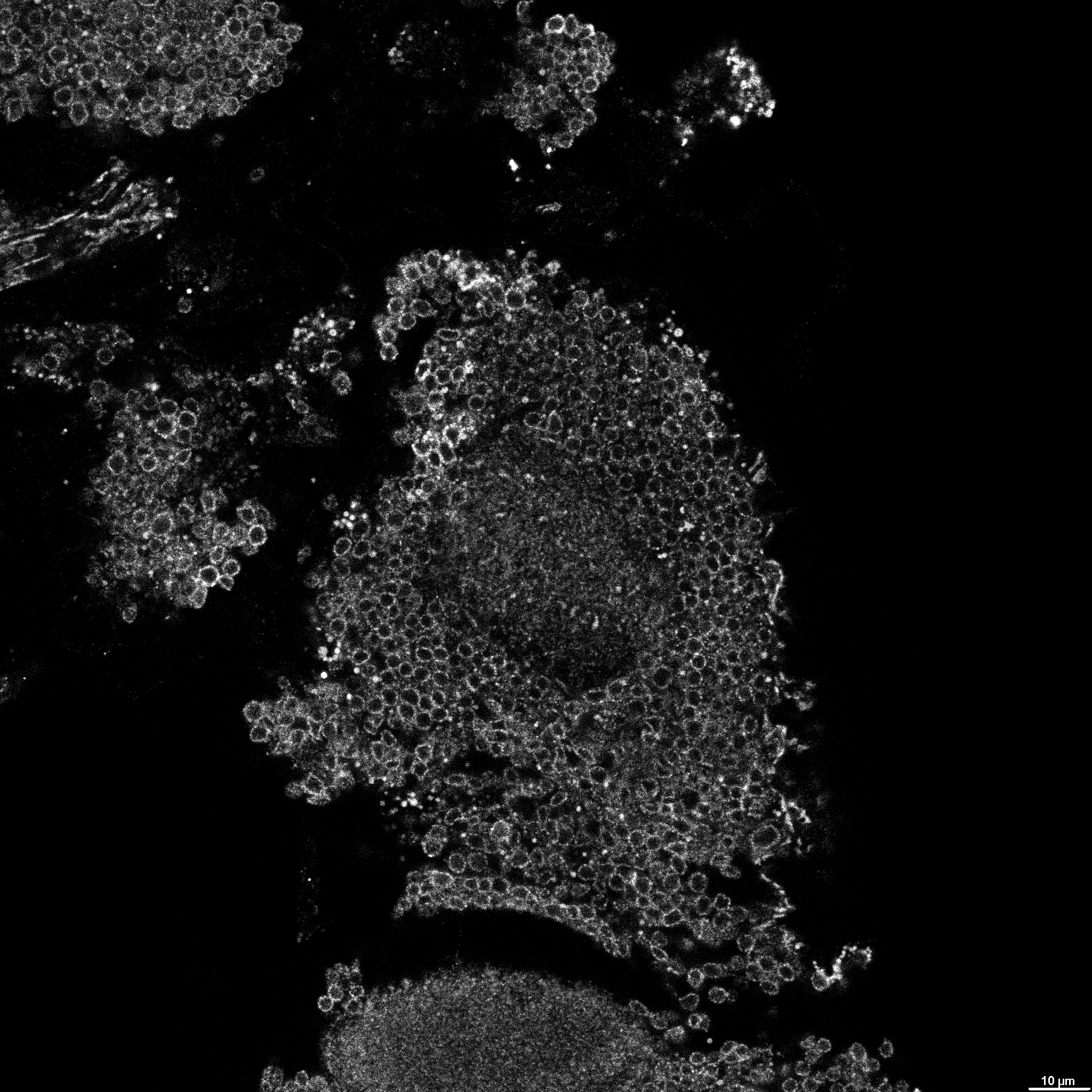

Supplement: Supplementary file 8 — Source data Fig. 5 [file 44319_2025_632_MOESM8_ESM.zip › Figure 5/5E/(top)nSyb-LexA over plus HA.tif]

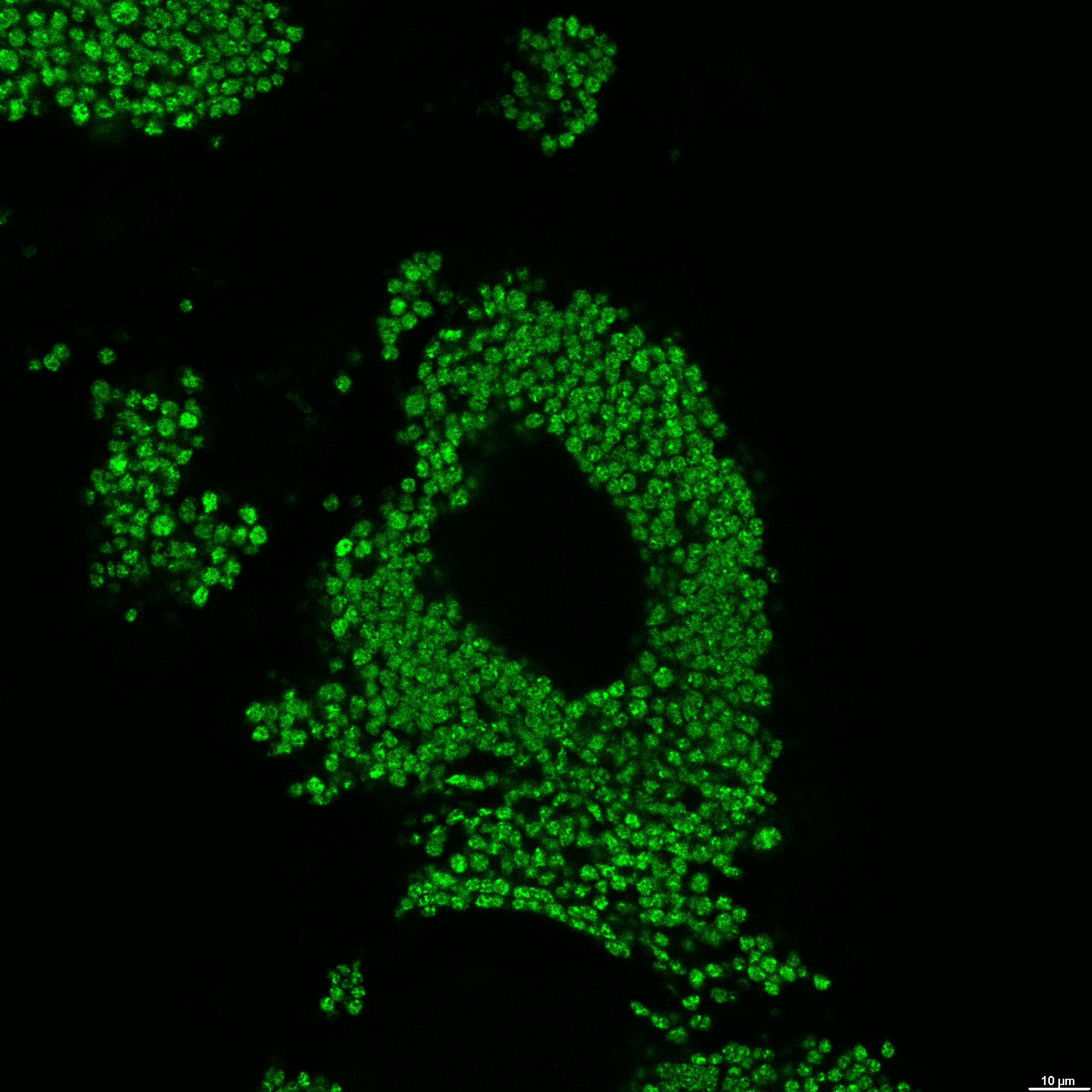

Supplement: Supplementary file 8 — Source data Fig. 5 [file 44319_2025_632_MOESM8_ESM.zip › Figure 5/5E/(top)nSyb-LexA over plus elav.tif]

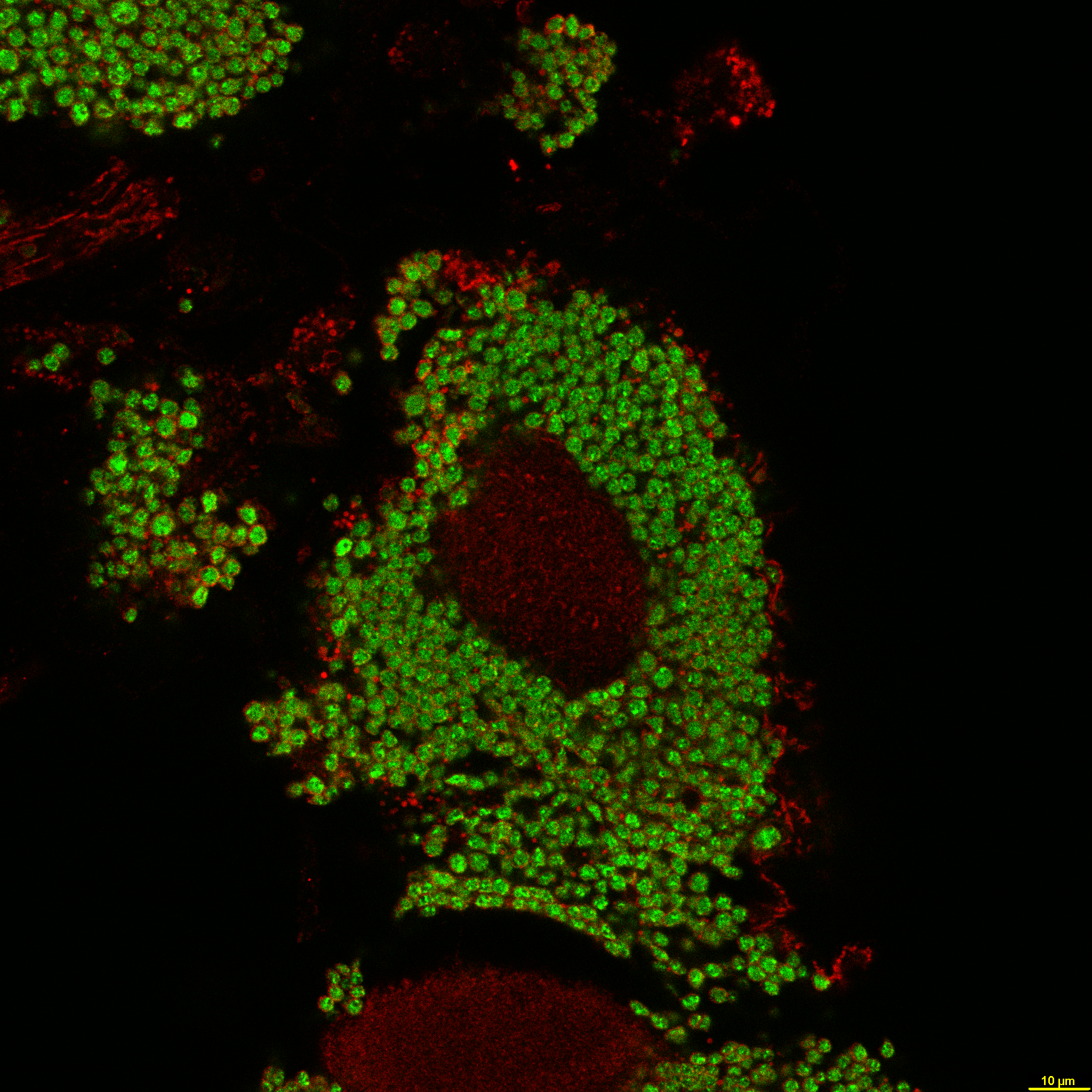

Supplement: Supplementary file 8 — Source data Fig. 5 [file 44319_2025_632_MOESM8_ESM.zip › Figure 5/5E/(top)nSyb-LexA over plus elav+HA.tif]

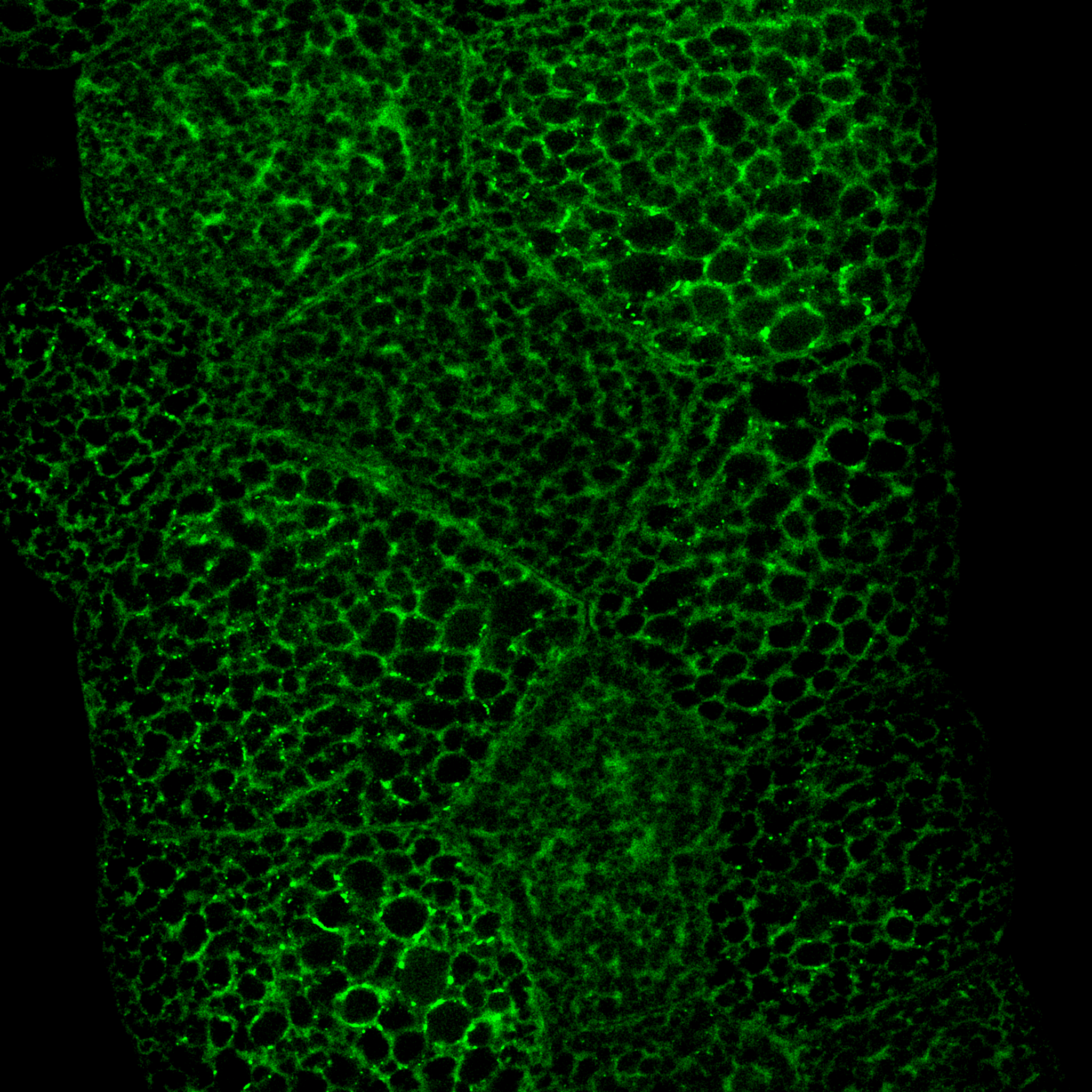

Supplement: Supplementary file 9 — Source data Fig. 6 [file 44319_2025_632_MOESM9_ESM.zip › Figure 6/6B/(top) control OlyAw.tif]

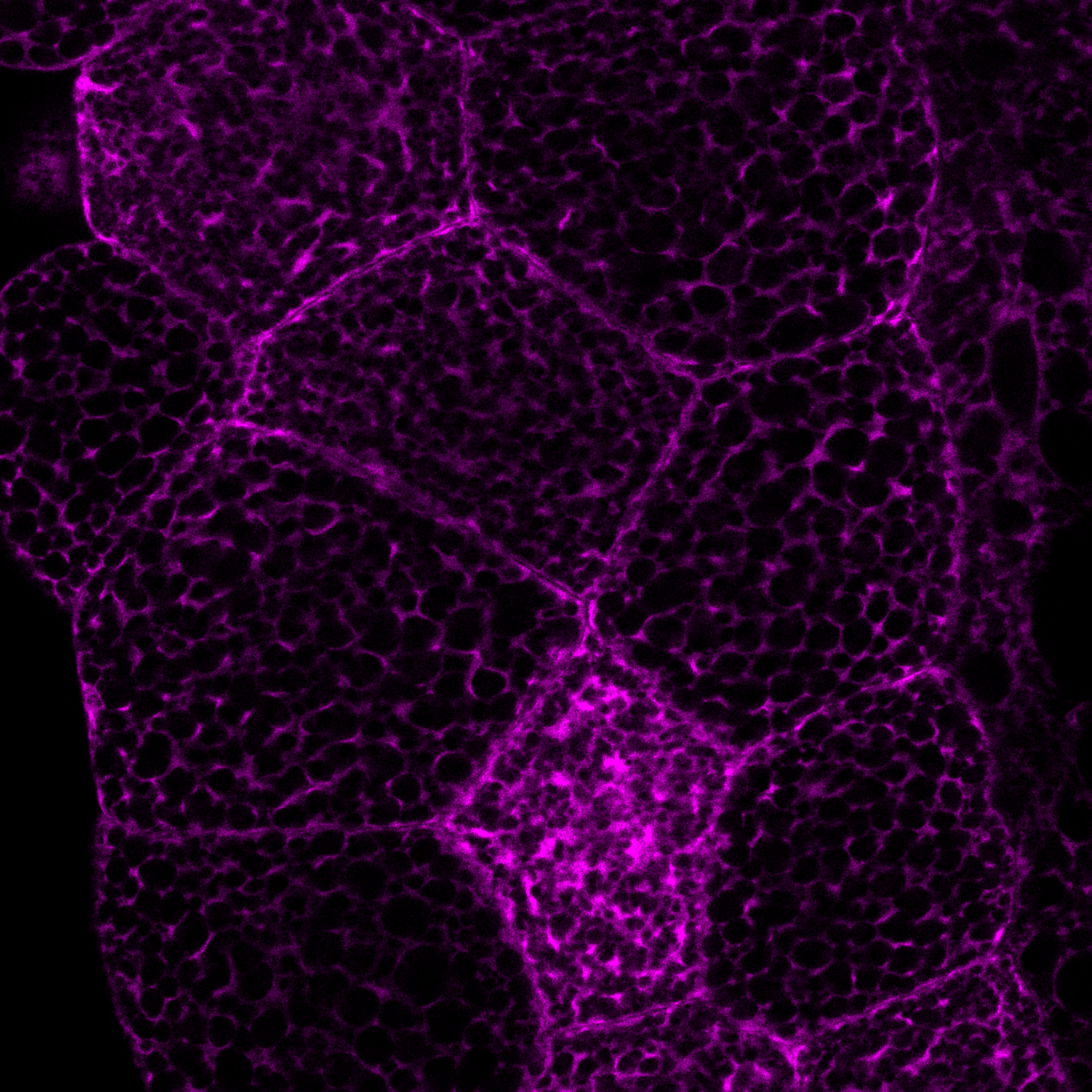

Supplement: Supplementary file 9 — Source data Fig. 6 [file 44319_2025_632_MOESM9_ESM.zip › Figure 6/6B/(top) control cnx99a.tif]

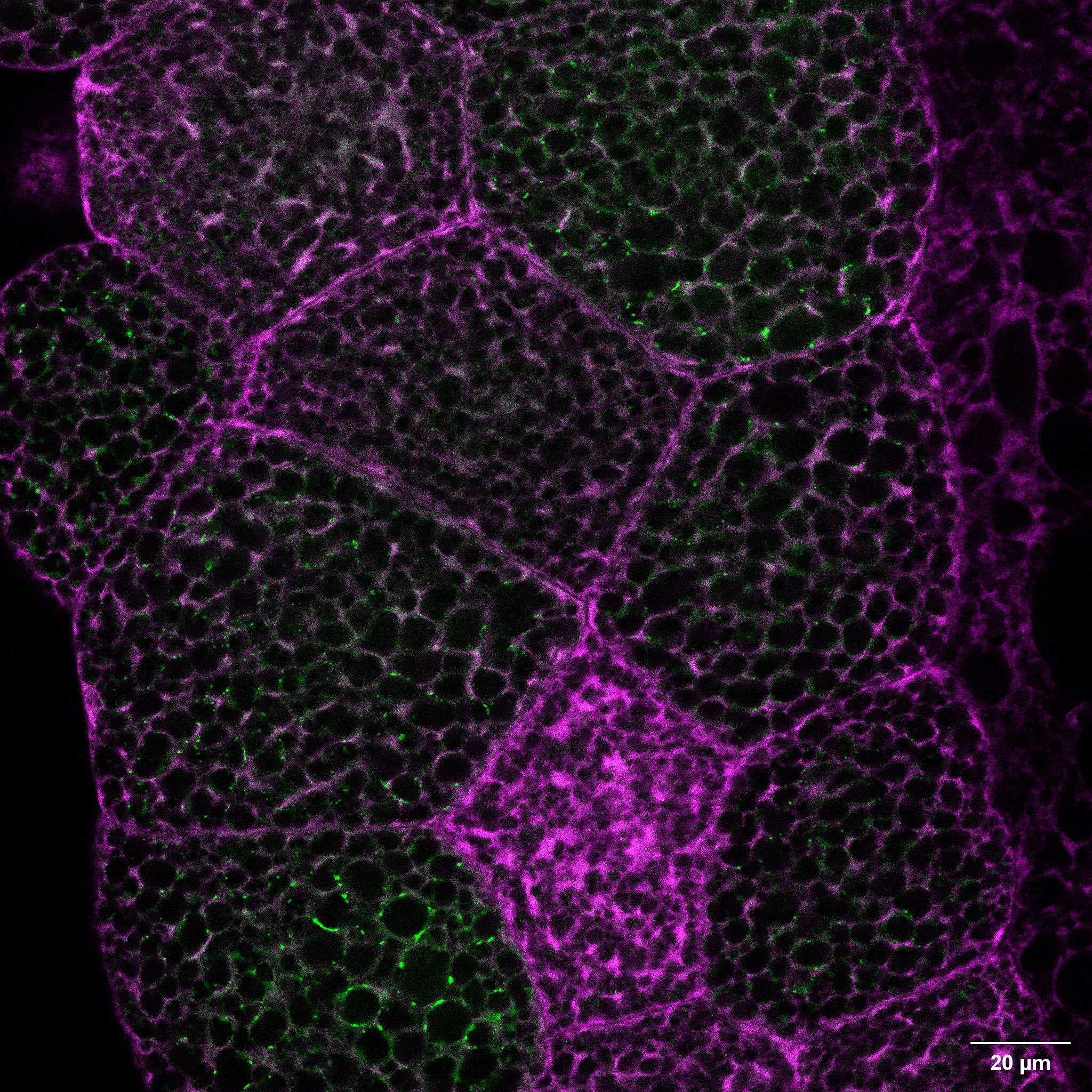

Supplement: Supplementary file 9 — Source data Fig. 6 [file 44319_2025_632_MOESM9_ESM.zip › Figure 6/6B/(top) control merge.tif]

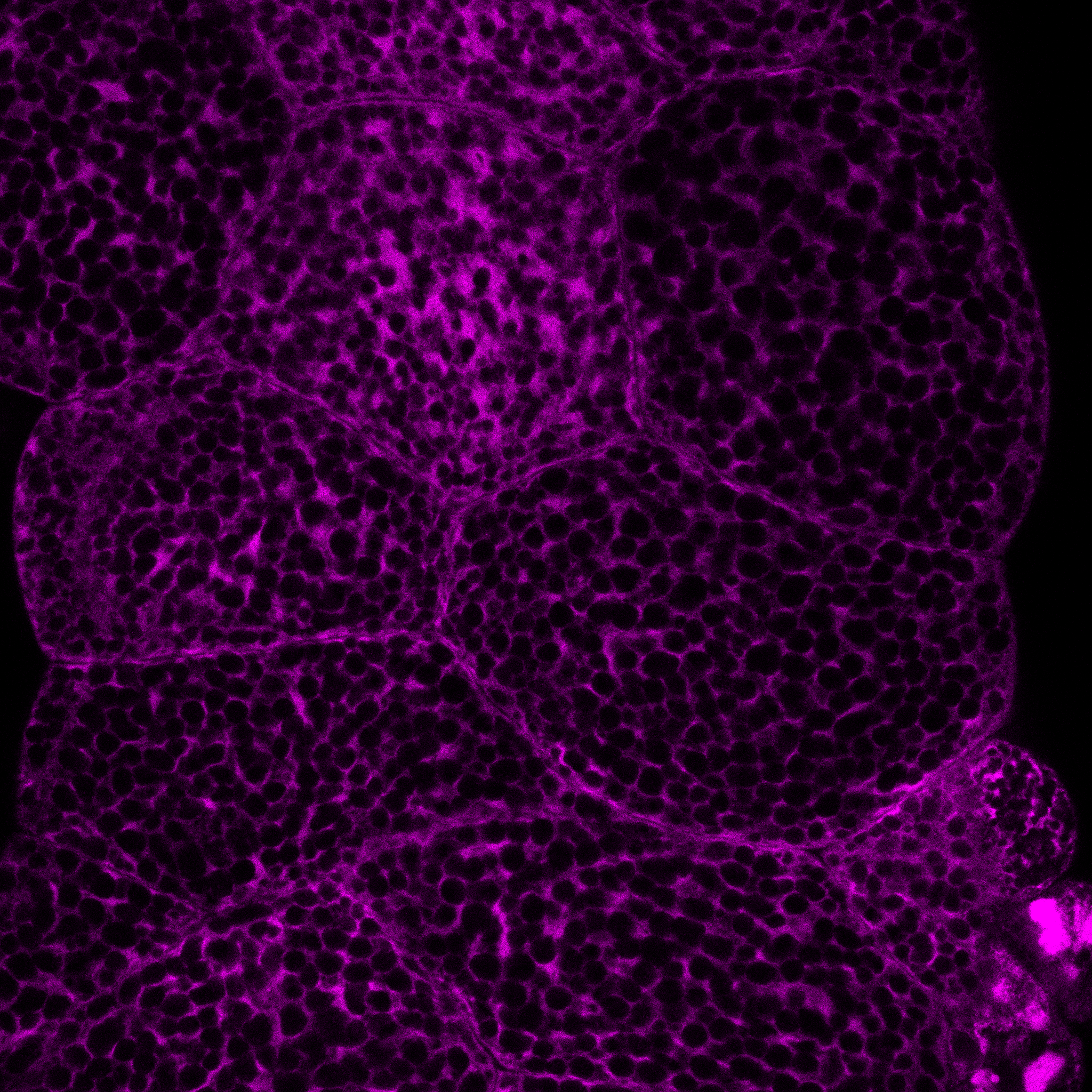

Supplement: Supplementary file 9 — Source data Fig. 6 [file 44319_2025_632_MOESM9_ESM.zip › Figure 6/6B/KO cnx99a.tif]

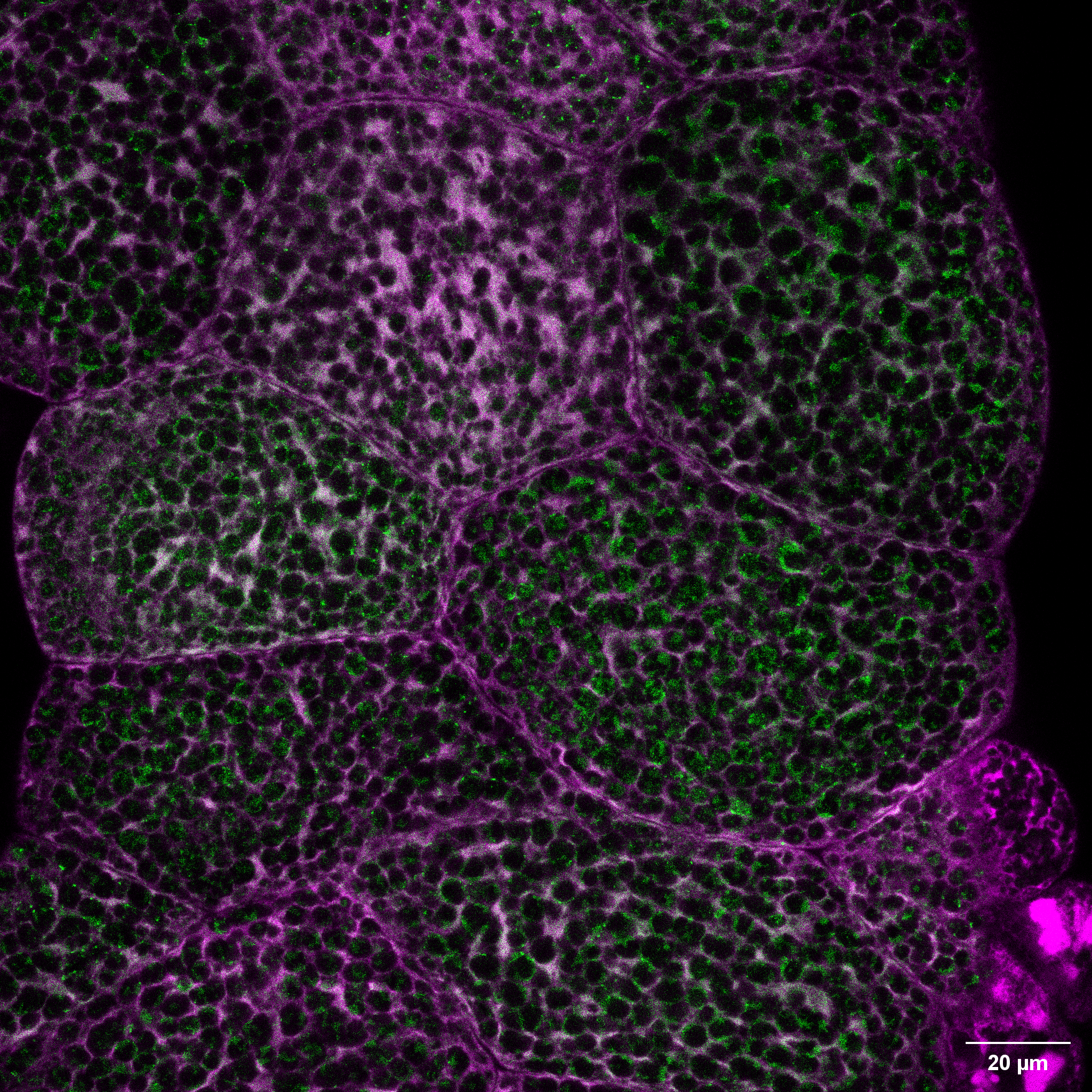

Supplement: Supplementary file 9 — Source data Fig. 6 [file 44319_2025_632_MOESM9_ESM.zip › Figure 6/6B/KO merge-1.tif]

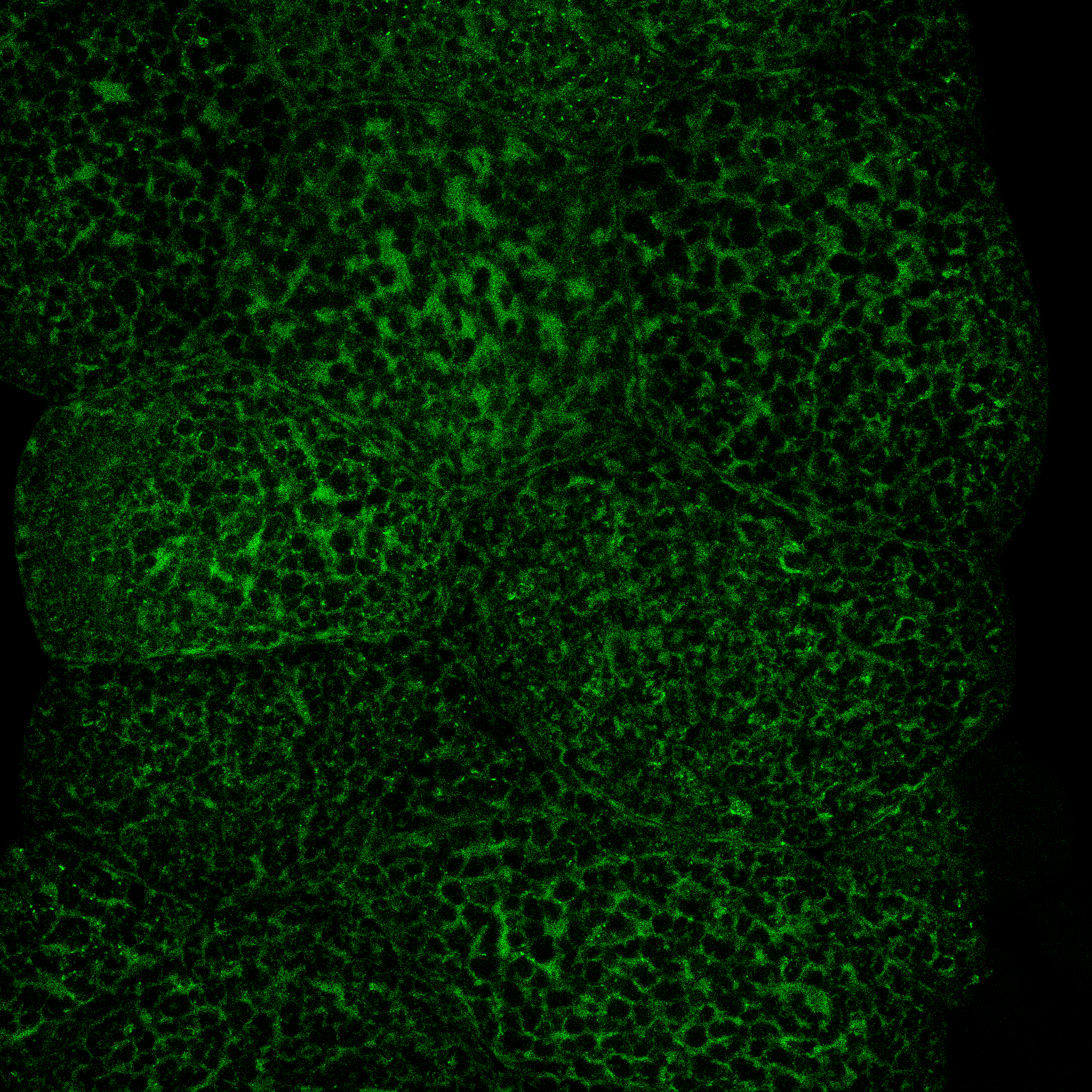

Supplement: Supplementary file 9 — Source data Fig. 6 [file 44319_2025_632_MOESM9_ESM.zip › Figure 6/6B/KO OlyAw.tif]

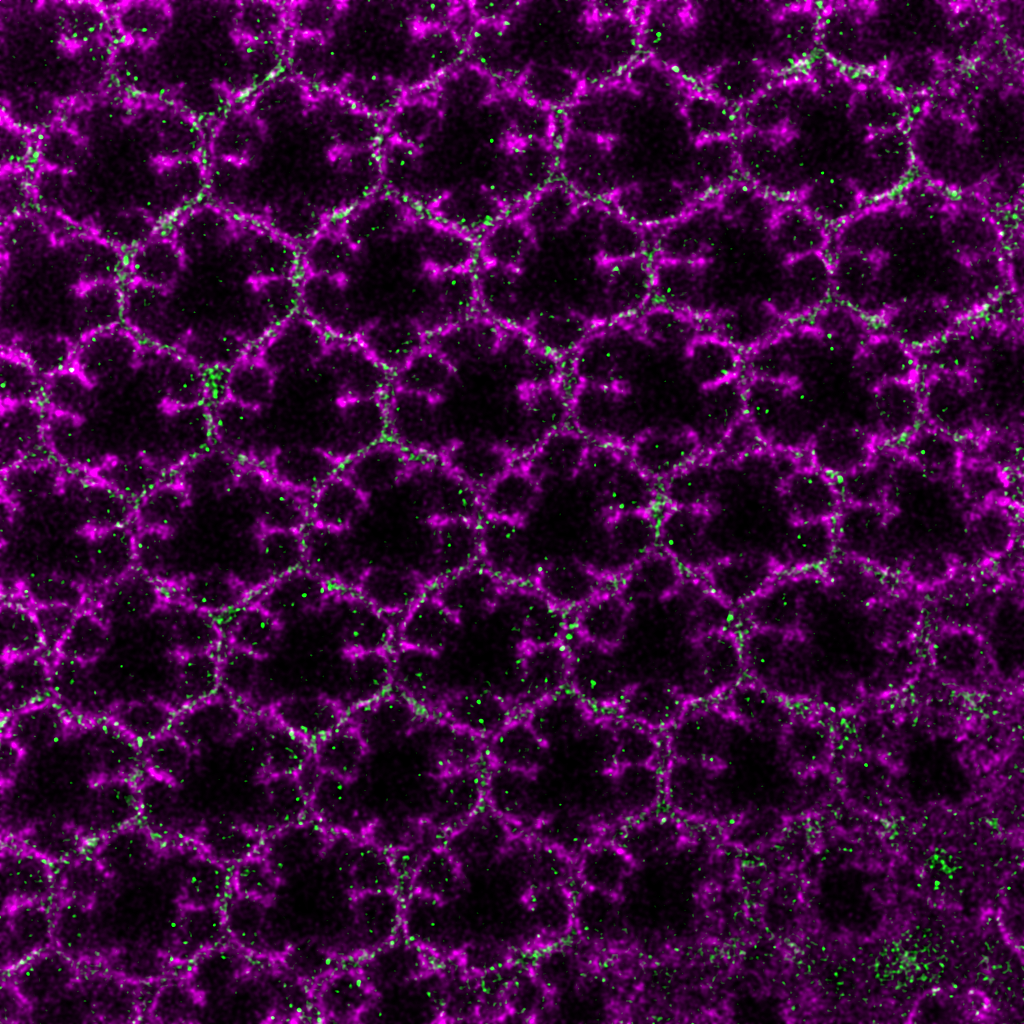

Supplement: Supplementary file 9 — Source data Fig. 6 [file 44319_2025_632_MOESM9_ESM.zip › Figure 6/6F/54C_decon_OlyA+membrane.tif]

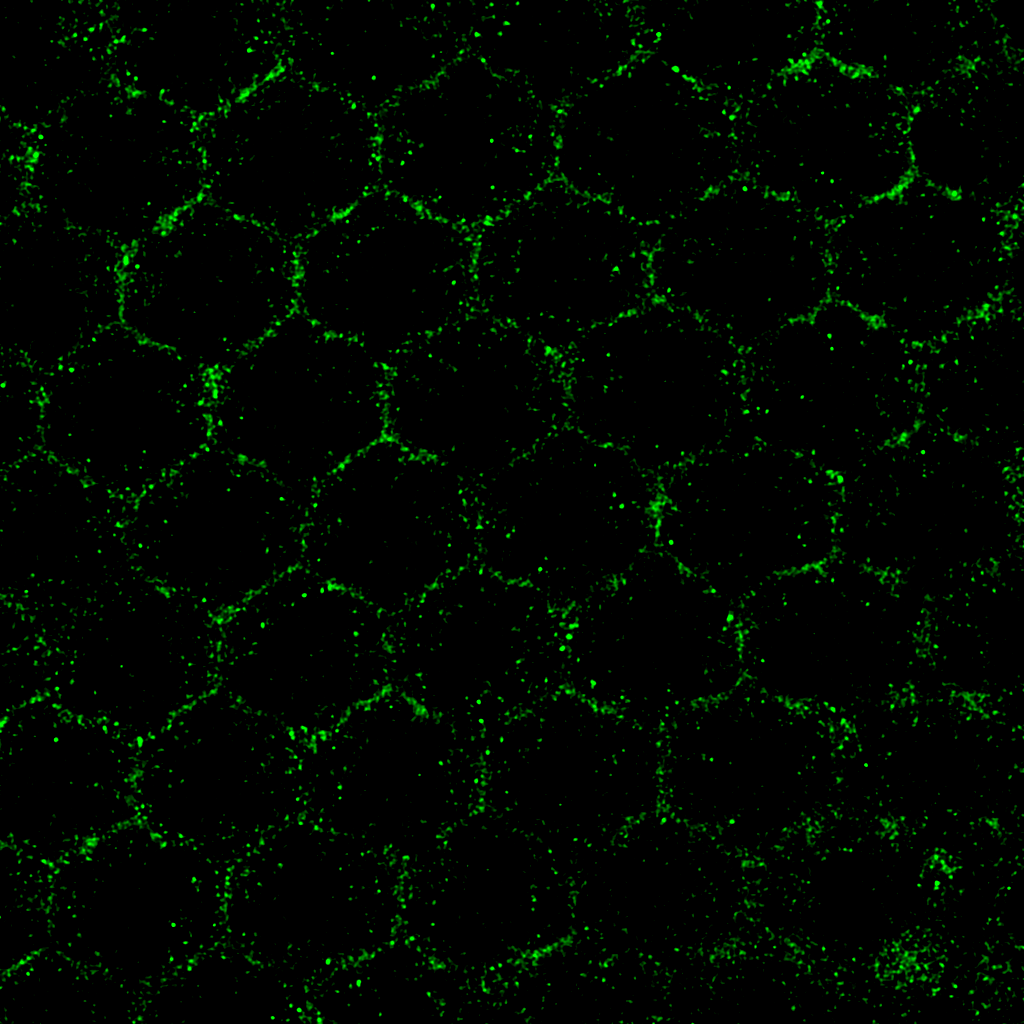

Supplement: Supplementary file 9 — Source data Fig. 6 [file 44319_2025_632_MOESM9_ESM.zip › Figure 6/6F/54C_decon_OlyAw.tif]

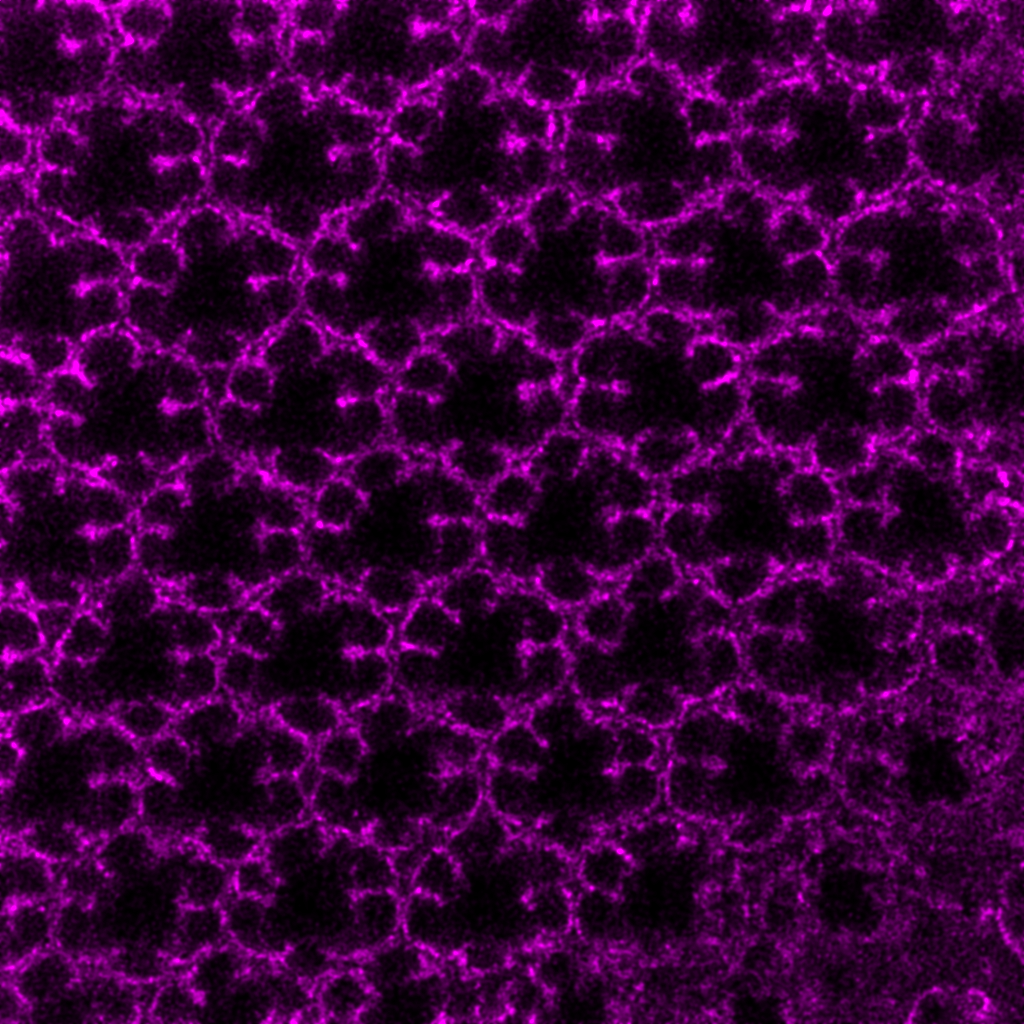

Supplement: Supplementary file 9 — Source data Fig. 6 [file 44319_2025_632_MOESM9_ESM.zip › Figure 6/6F/54C_decon_membrane.tif]

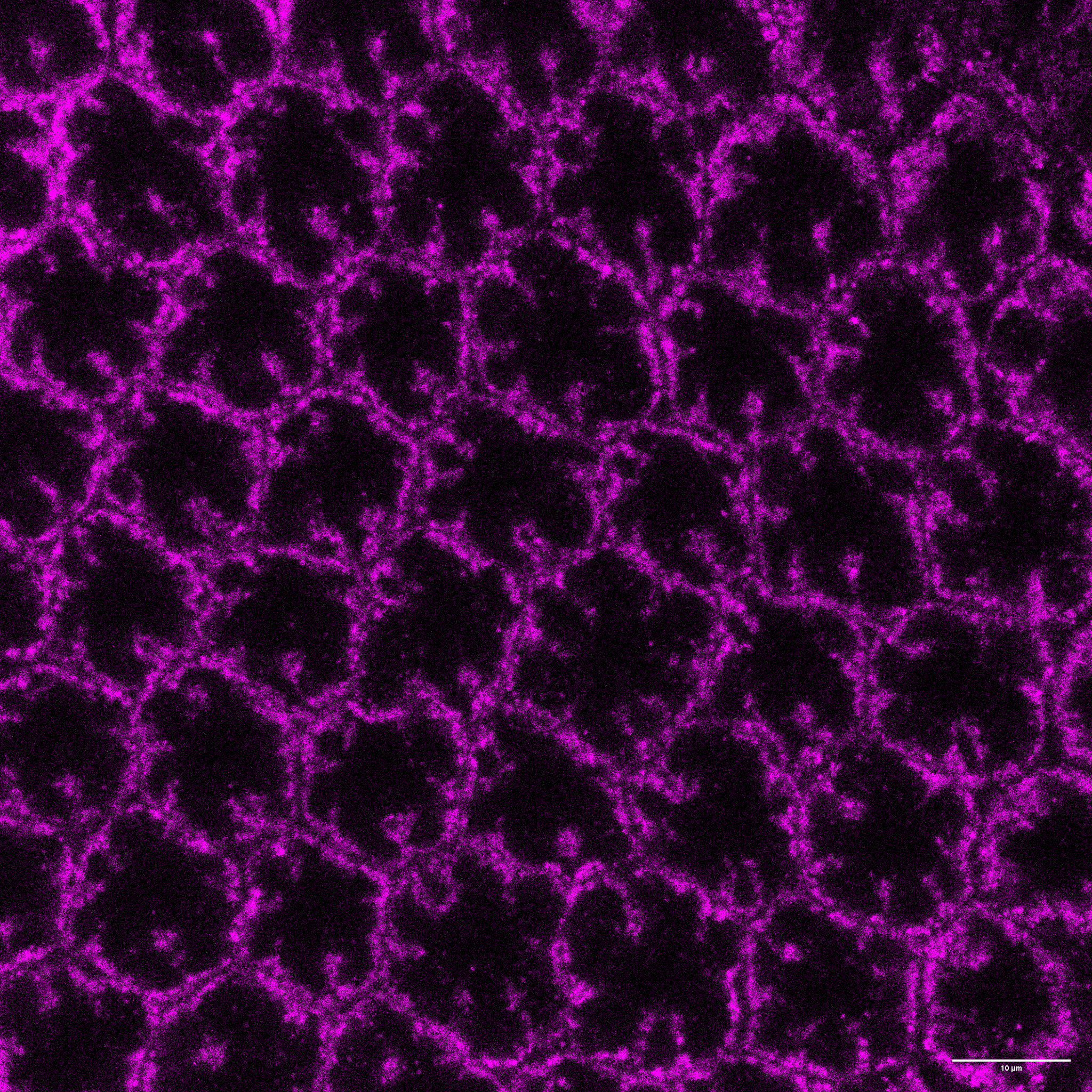

Supplement: Supplementary file 9 — Source data Fig. 6 [file 44319_2025_632_MOESM9_ESM.zip › Figure 6/6G/rh1 membrane.tif (RGB).tif]

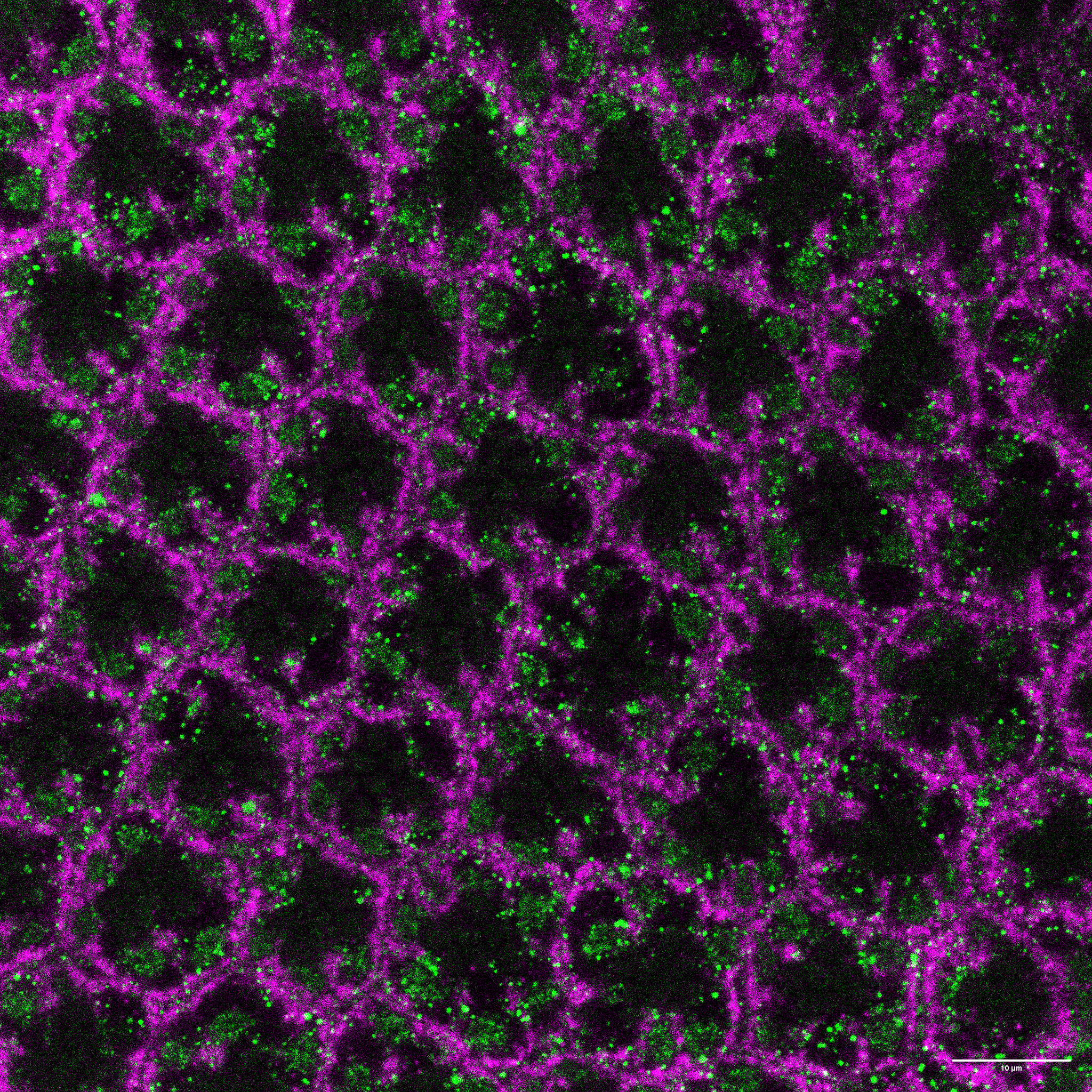

Supplement: Supplementary file 9 — Source data Fig. 6 [file 44319_2025_632_MOESM9_ESM.zip › Figure 6/6G/rh1 merge.tif (RGB).tif]

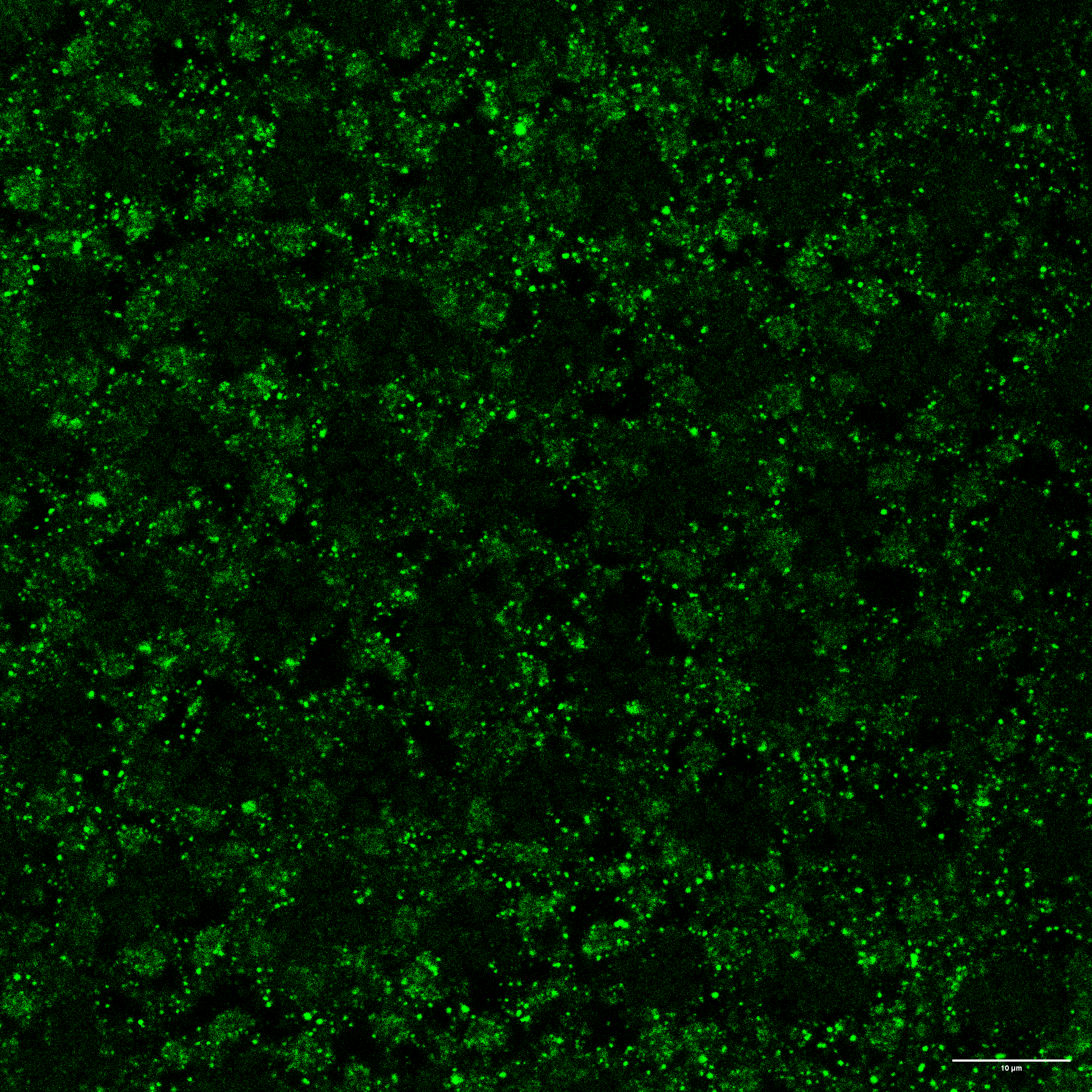

Supplement: Supplementary file 9 — Source data Fig. 6 [file 44319_2025_632_MOESM9_ESM.zip › Figure 6/6G/rh1 OlyAw.tif (RGB).tif]

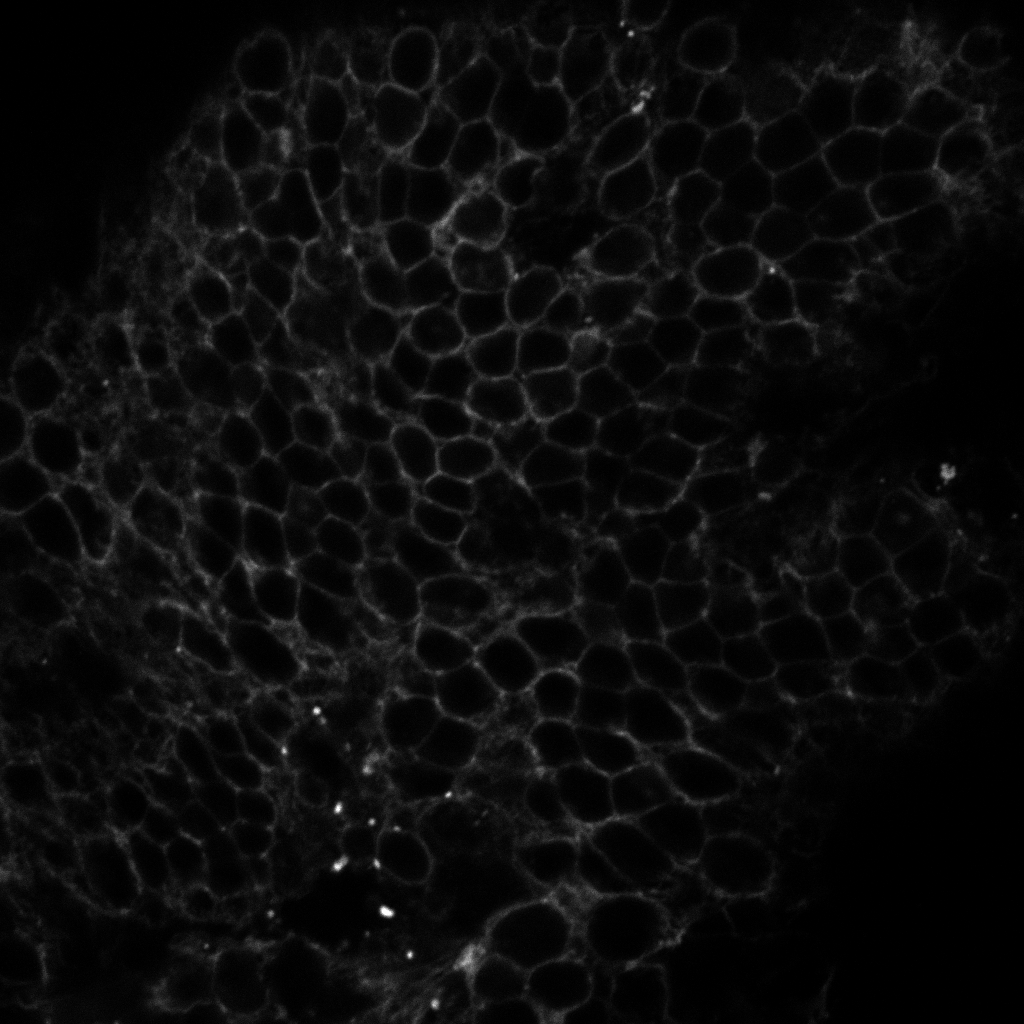

Supplement: Supplementary file 9 — Source data Fig. 6 [file 44319_2025_632_MOESM9_ESM.zip › Figure 6/6H/flo2RFP.tif]

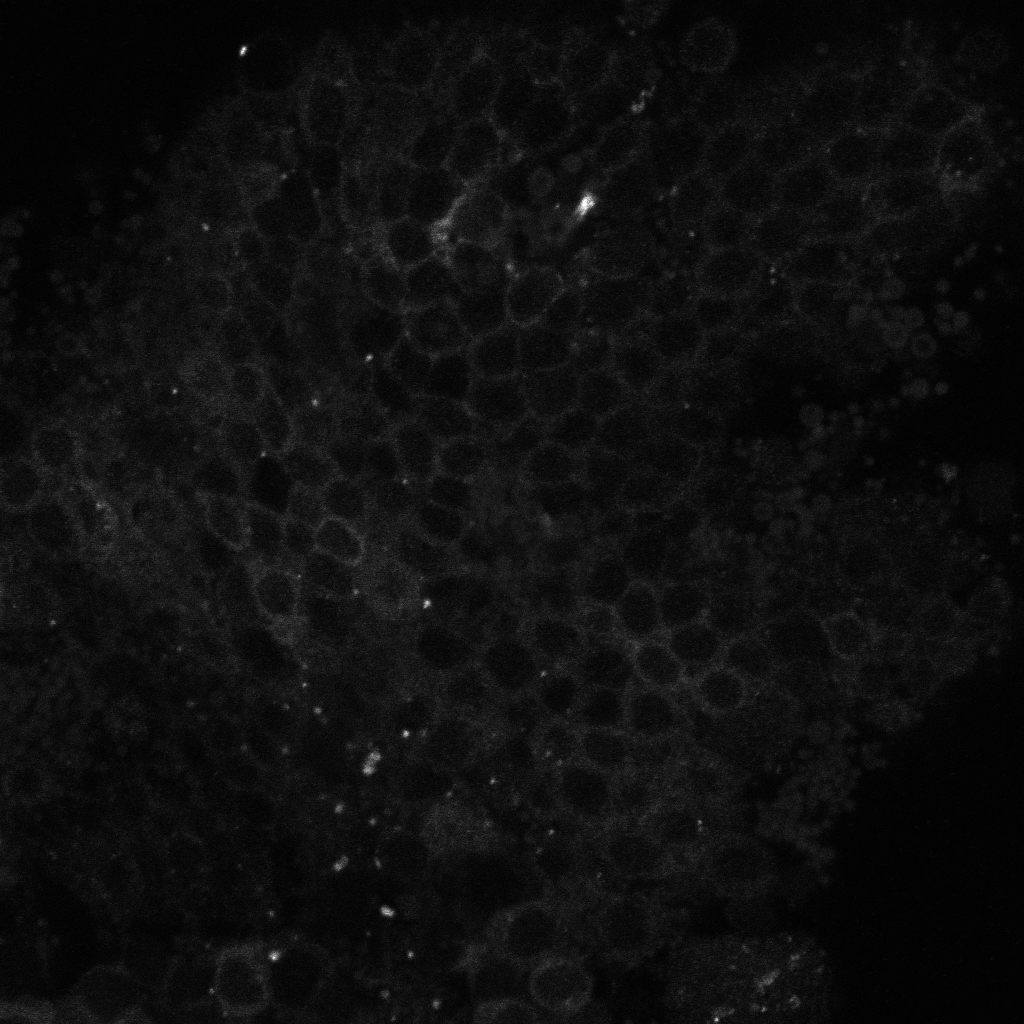

Supplement: Supplementary file 9 — Source data Fig. 6 [file 44319_2025_632_MOESM9_ESM.zip › Figure 6/6H/OlyAw.tif]

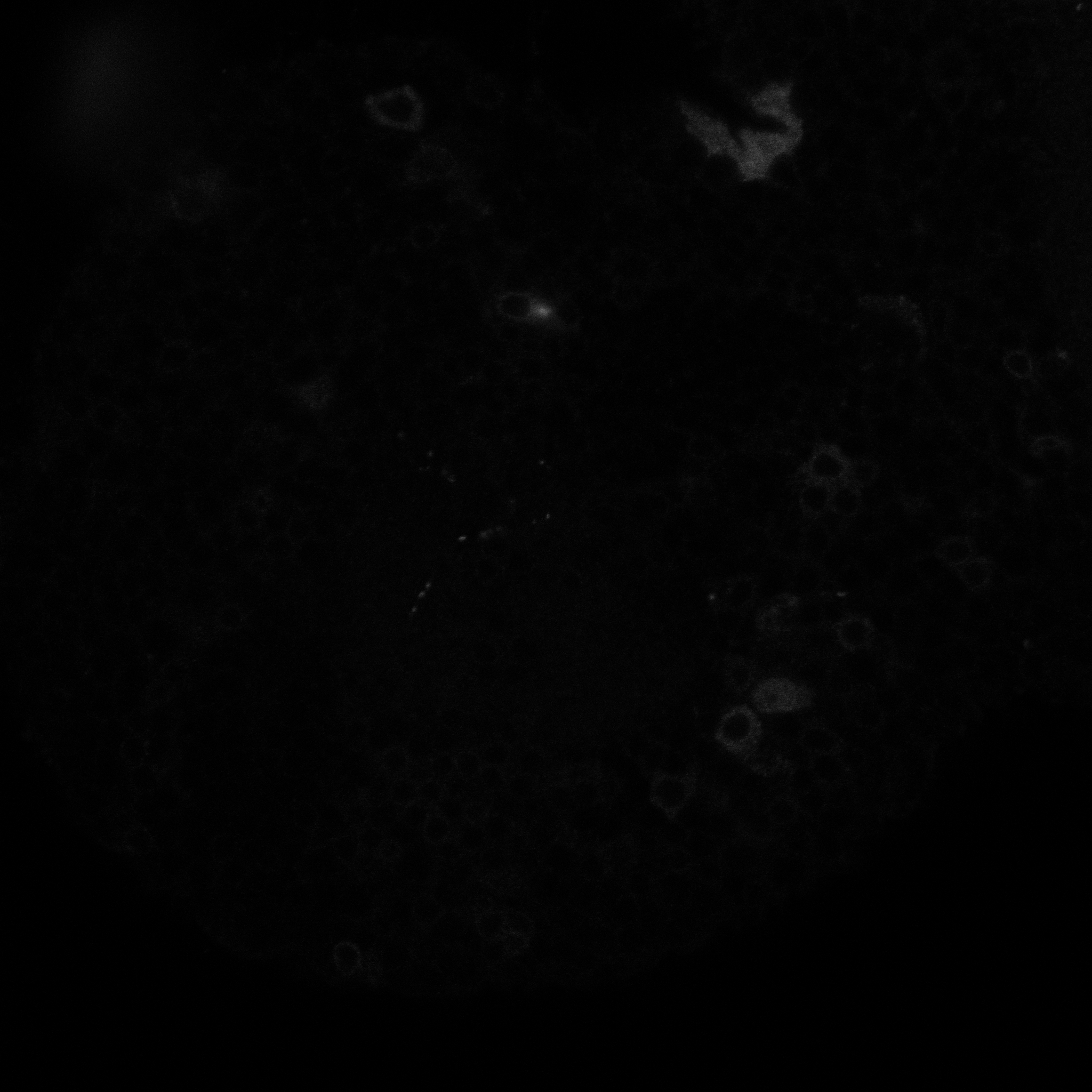

Supplement: Supplementary file 9 — Source data Fig. 6 [file 44319_2025_632_MOESM9_ESM.zip › Figure 6/6I/OlyAw.tif]

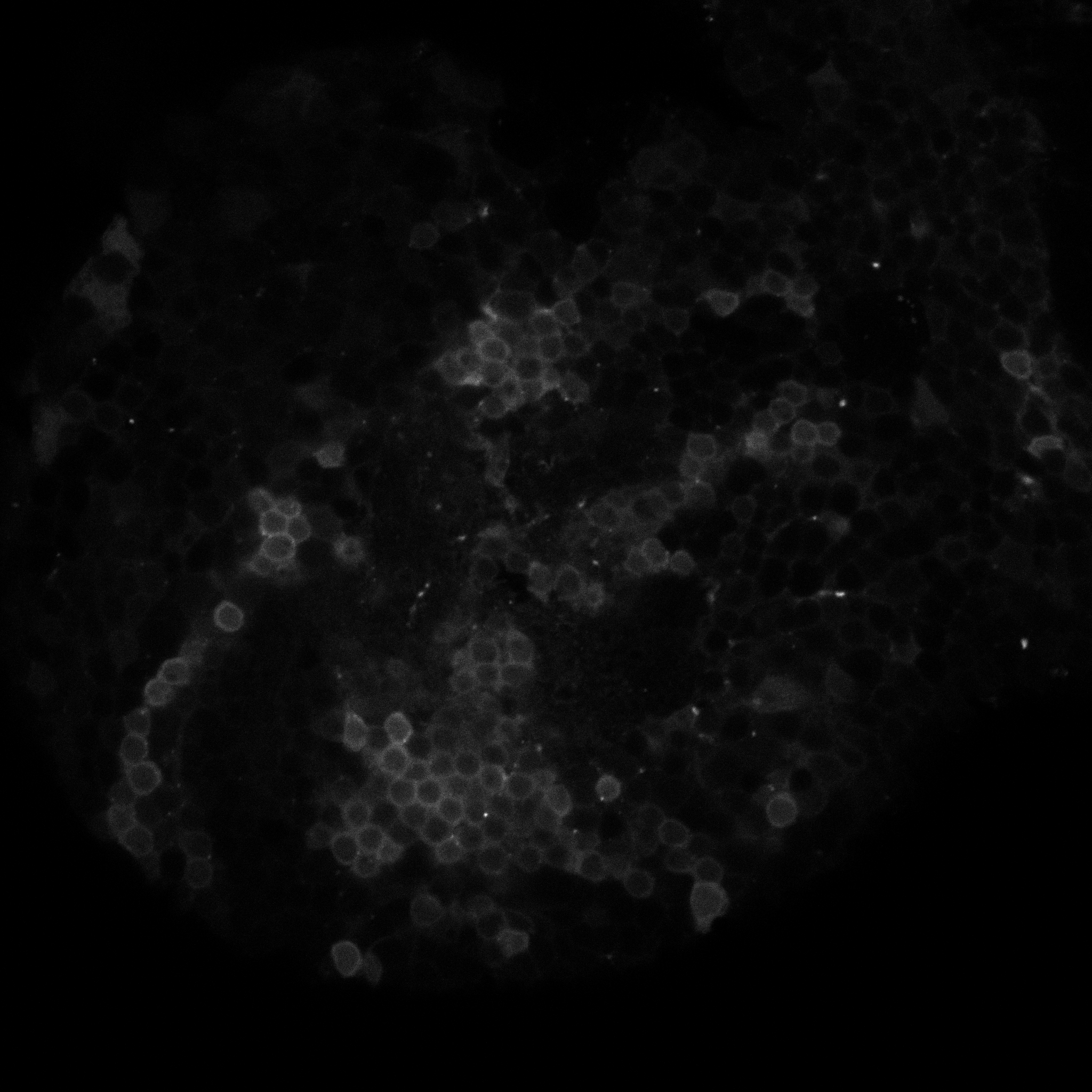

Supplement: Supplementary file 9 — Source data Fig. 6 [file 44319_2025_632_MOESM9_ESM.zip › Figure 6/6I/GPI-mcherry.tif]

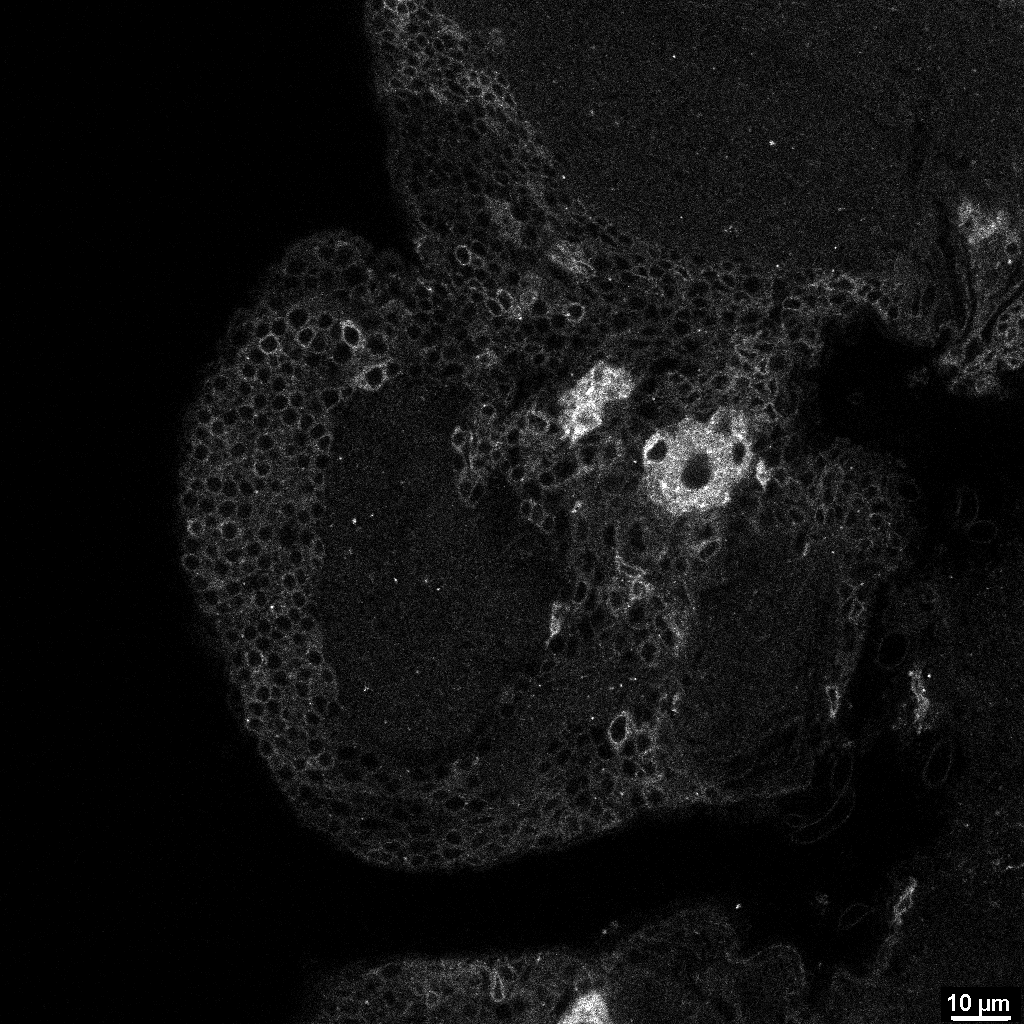

Supplement: Supplementary file 10 — Source data Fig. 7 [file 44319_2025_632_MOESM10_ESM.zip › Figure 7/7A/nSyb-GAL4 LUC-IR_inset.tif]

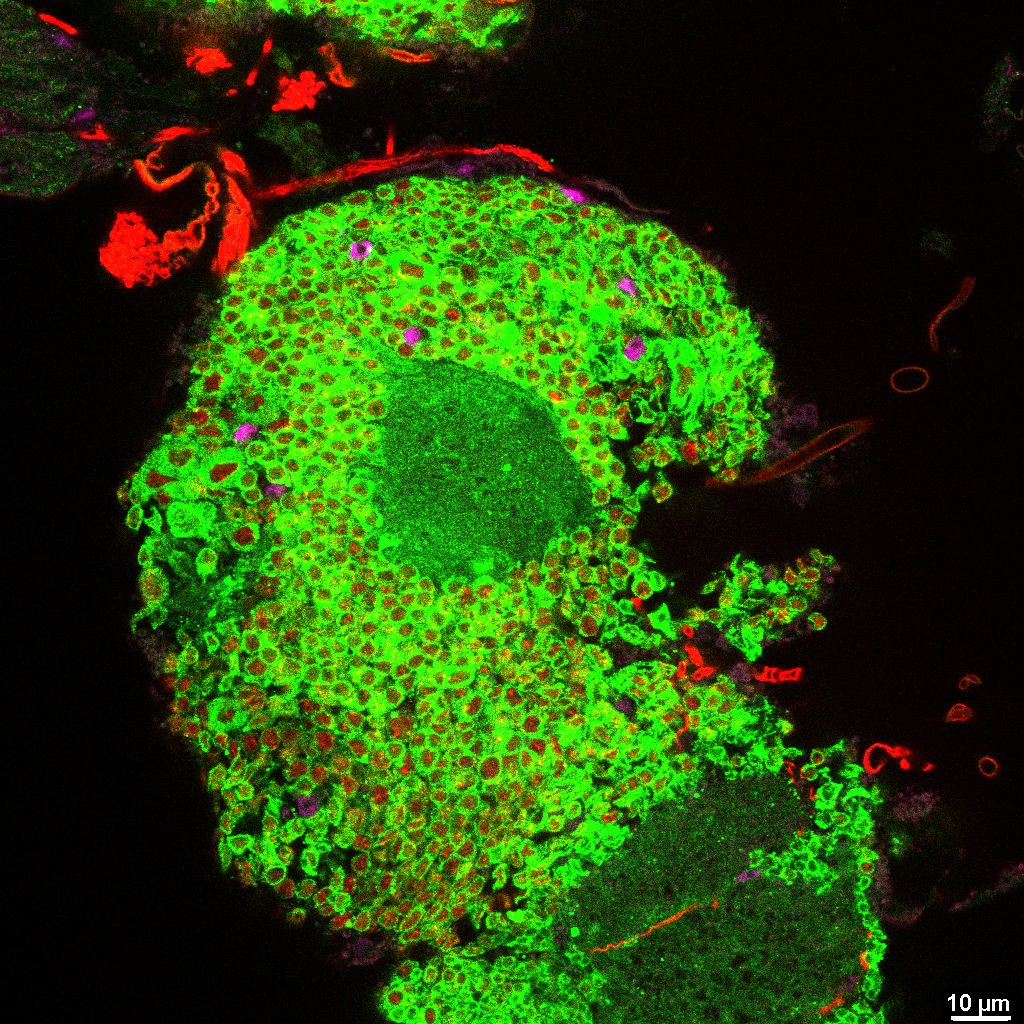

Supplement: Supplementary file 10 — Source data Fig. 7 [file 44319_2025_632_MOESM10_ESM.zip › Figure 7/7A/nSyb-GAL4 dSMPD4-IR BL51682_Merge.tif]

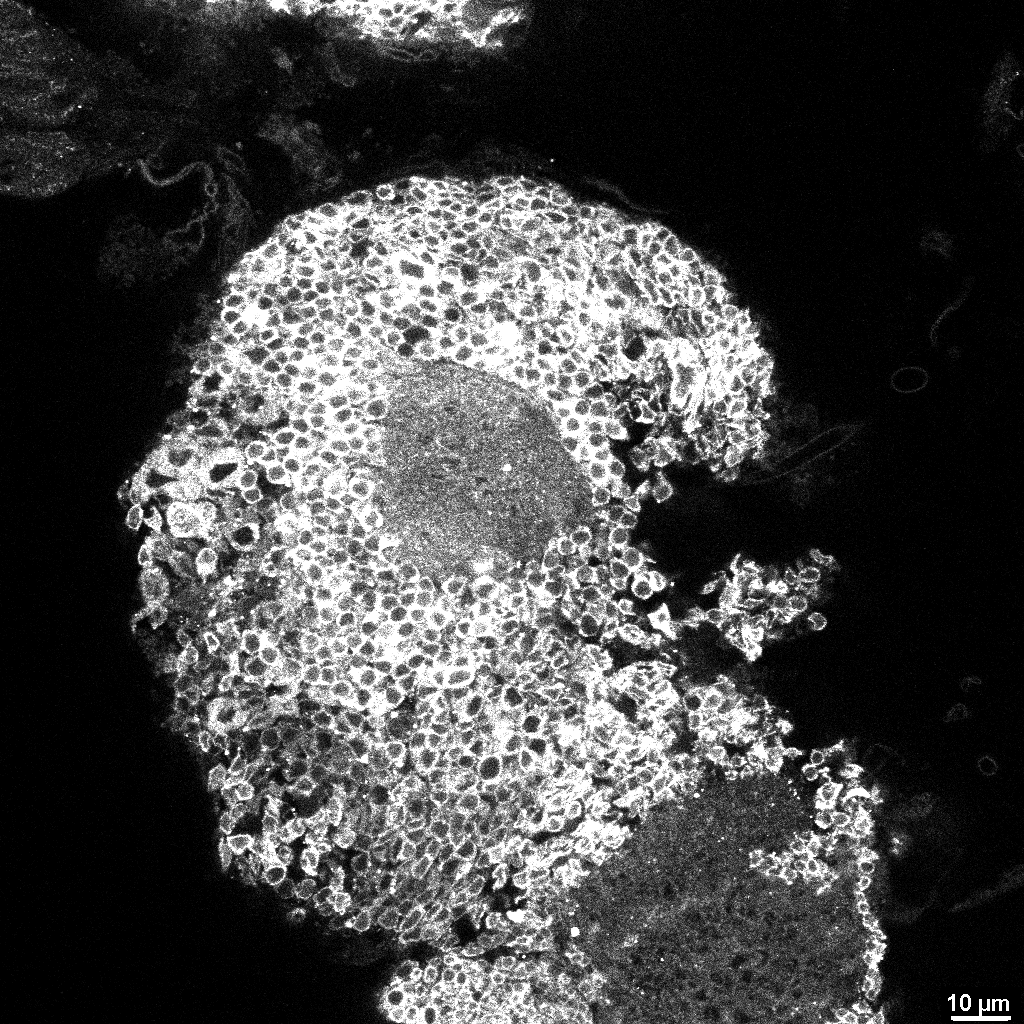

Supplement: Supplementary file 10 — Source data Fig. 7 [file 44319_2025_632_MOESM10_ESM.zip › Figure 7/7A/nSyb-GAL4 dSMPD4-IR BL51682_inset.tif]

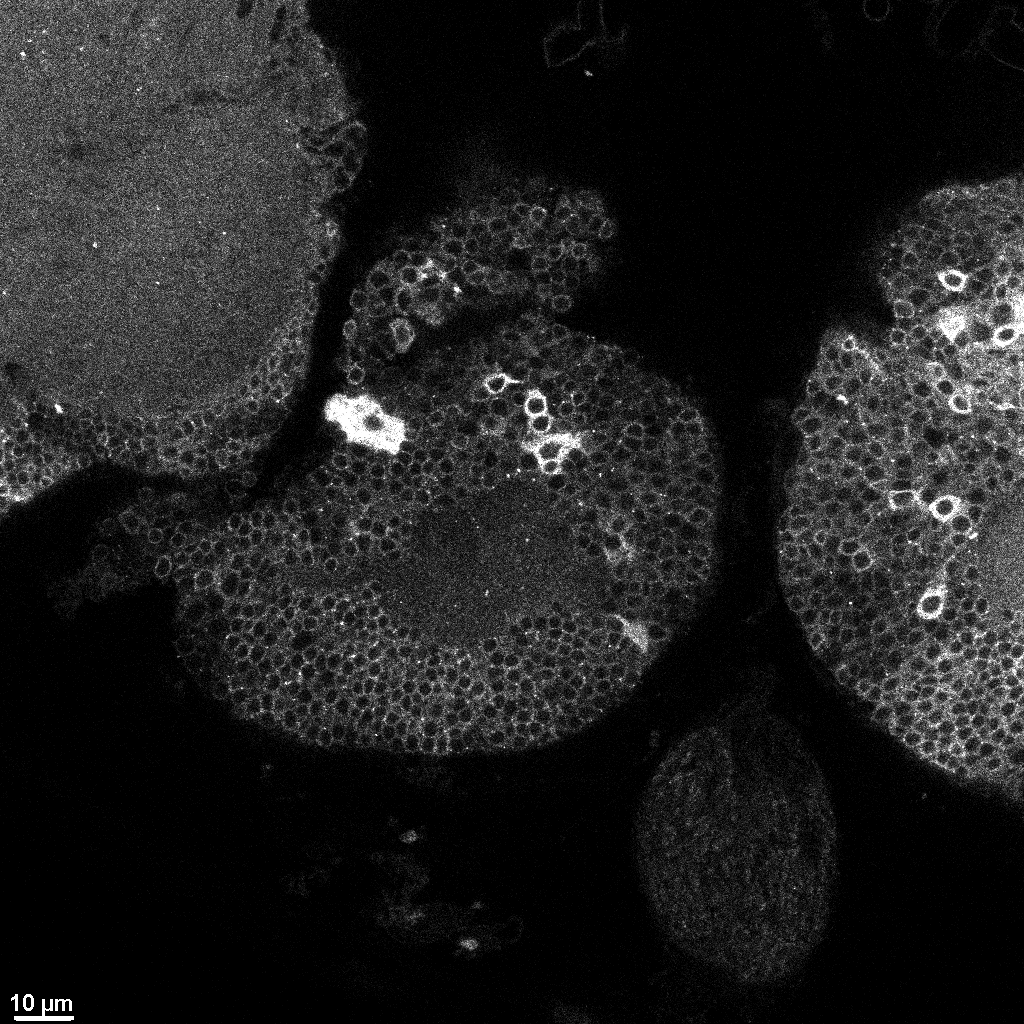

Supplement: Supplementary file 10 — Source data Fig. 7 [file 44319_2025_632_MOESM10_ESM.zip › Figure 7/7A/nSyb-GAL4 dSMPD4-IR v110163_inset.tif]

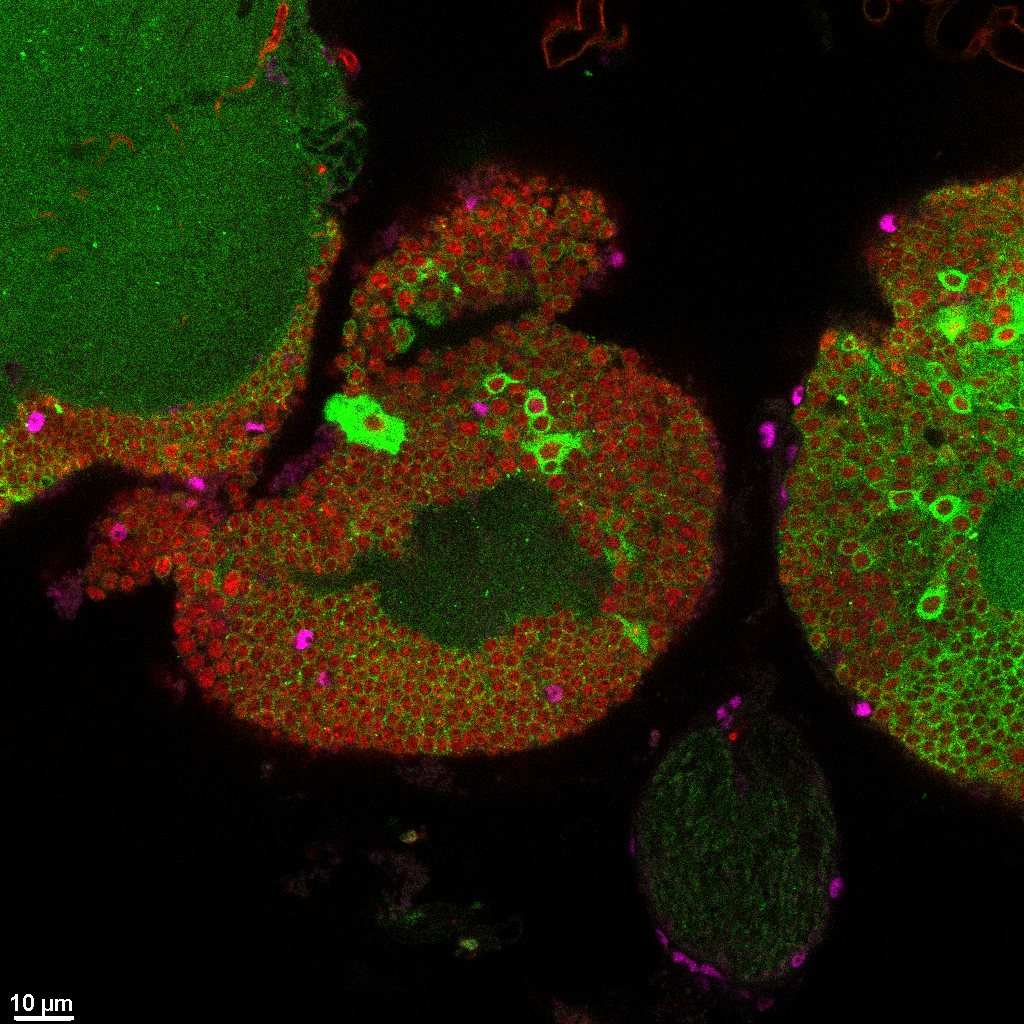

Supplement: Supplementary file 10 — Source data Fig. 7 [file 44319_2025_632_MOESM10_ESM.zip › Figure 7/7A/nSyb-GAL4 dSMPD4-IR v110163_Merge.tif]

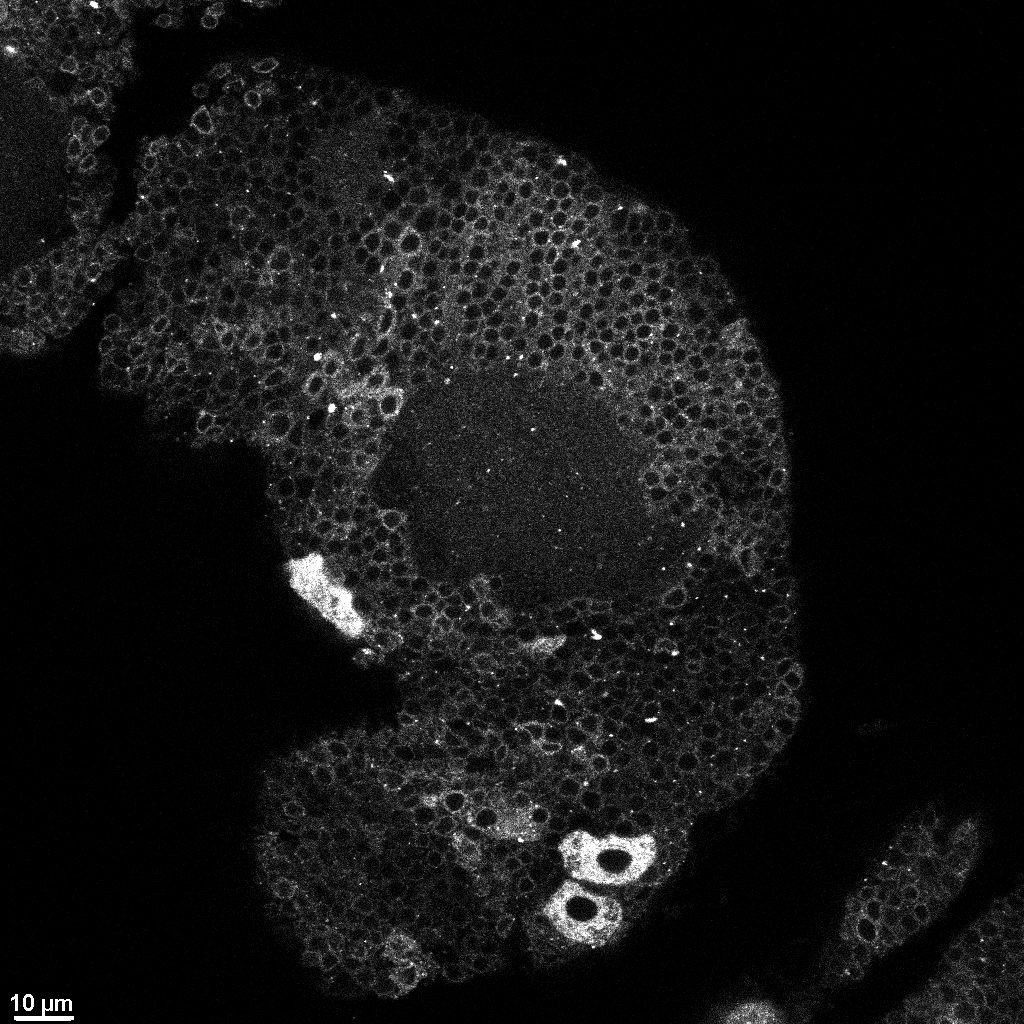

Supplement: Supplementary file 10 — Source data Fig. 7 [file 44319_2025_632_MOESM10_ESM.zip › Figure 7/7A/nSyb-GAL4 nSMase-IR BL36759_inset.tif]

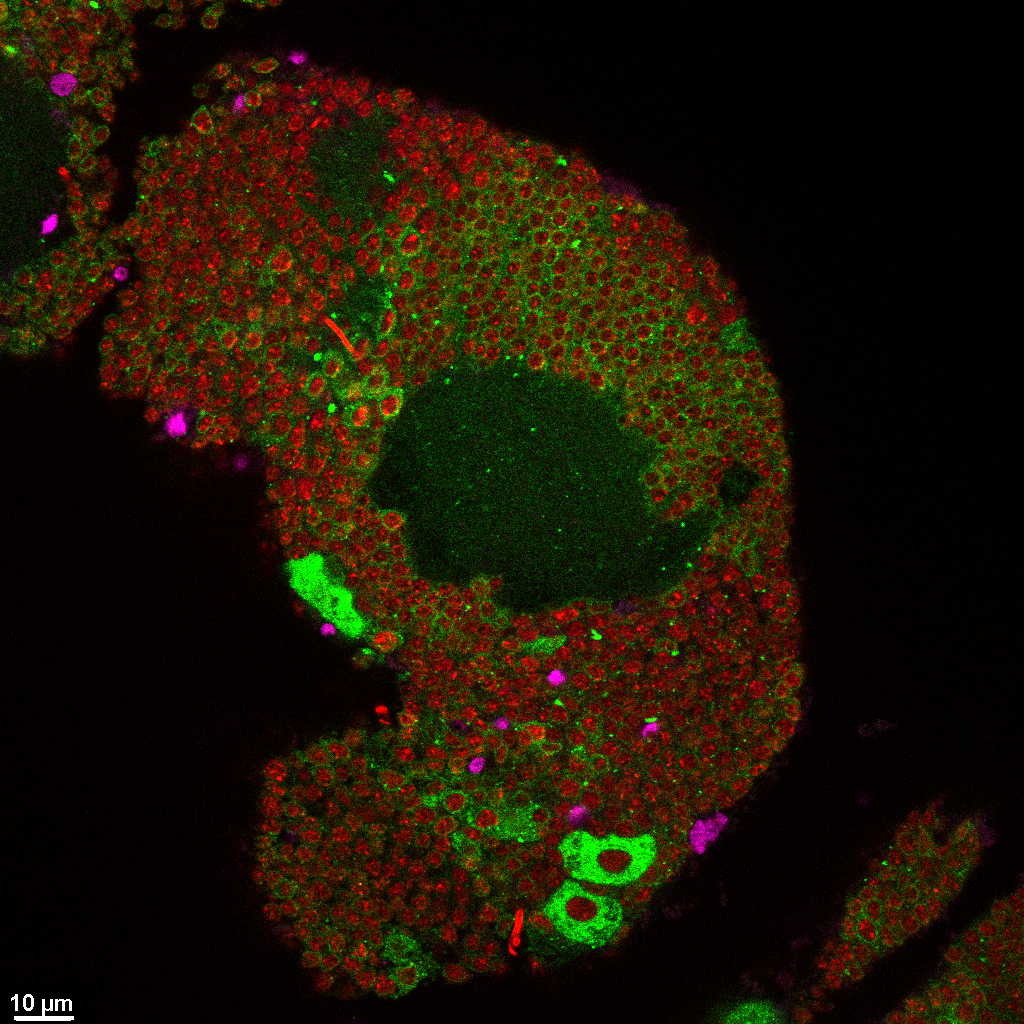

Supplement: Supplementary file 10 — Source data Fig. 7 [file 44319_2025_632_MOESM10_ESM.zip › Figure 7/7A/nSyb-GAL4 nSMase-IR BL36759_Merge.tif]

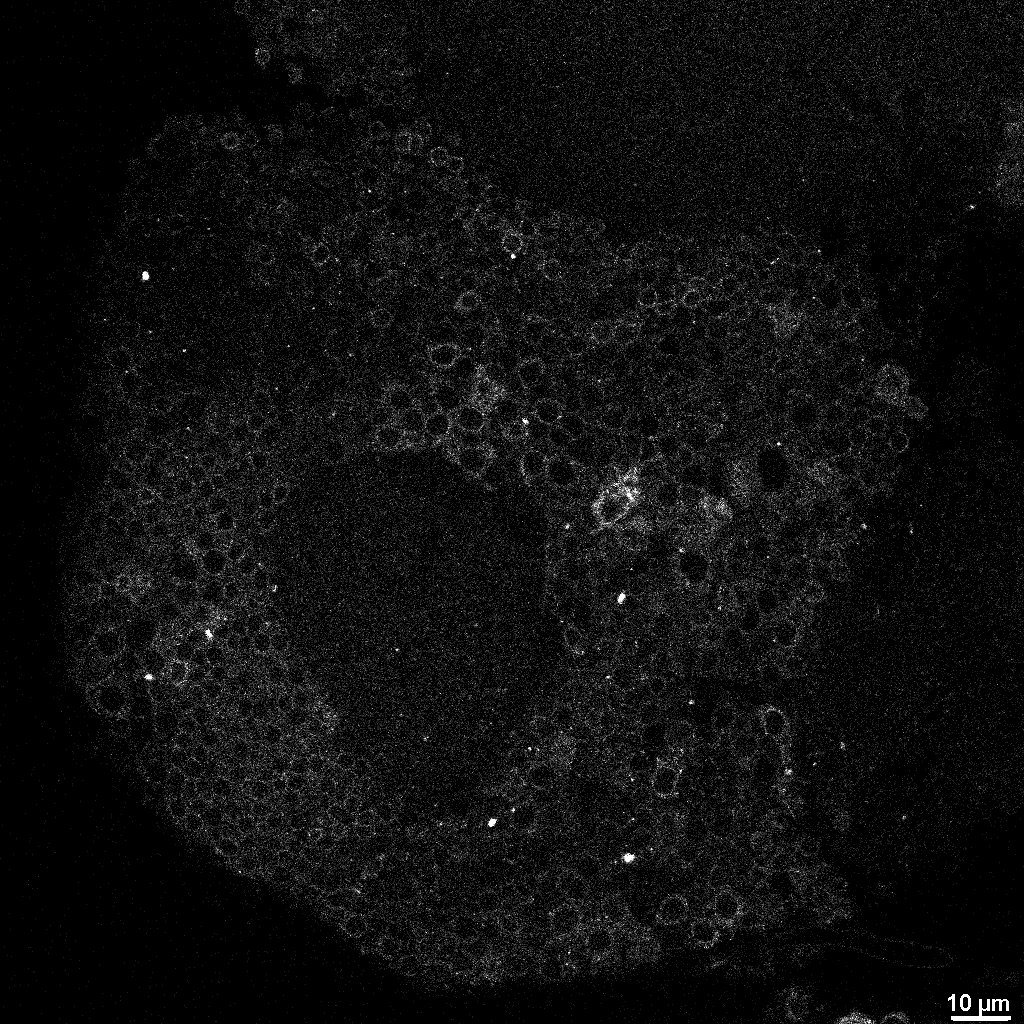

Supplement: Supplementary file 10 — Source data Fig. 7 [file 44319_2025_632_MOESM10_ESM.zip › Figure 7/7A/nSyb-GAL4 nSMase-IR v107062_inset.tif]

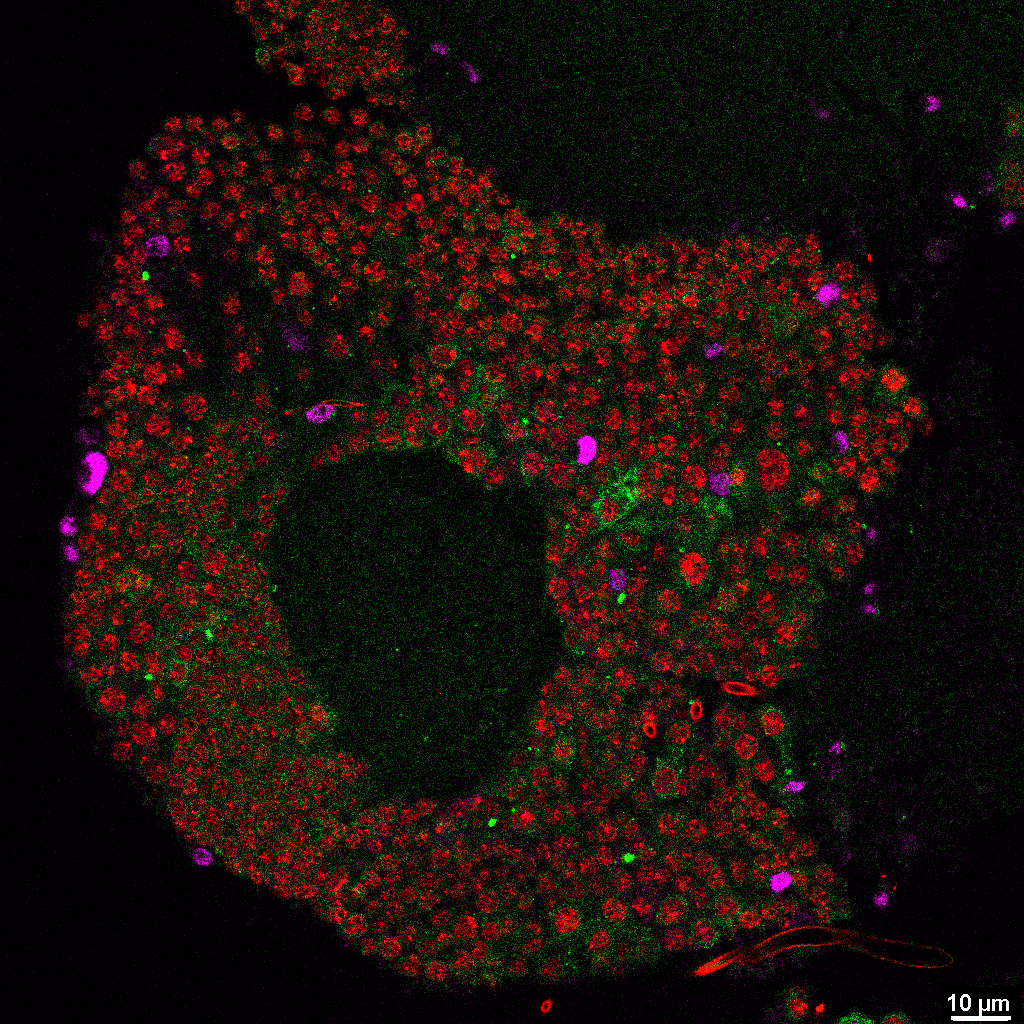

Supplement: Supplementary file 10 — Source data Fig. 7 [file 44319_2025_632_MOESM10_ESM.zip › Figure 7/7A/nSyb-GAL4 nSMase-IR v107062_Merge.tif]

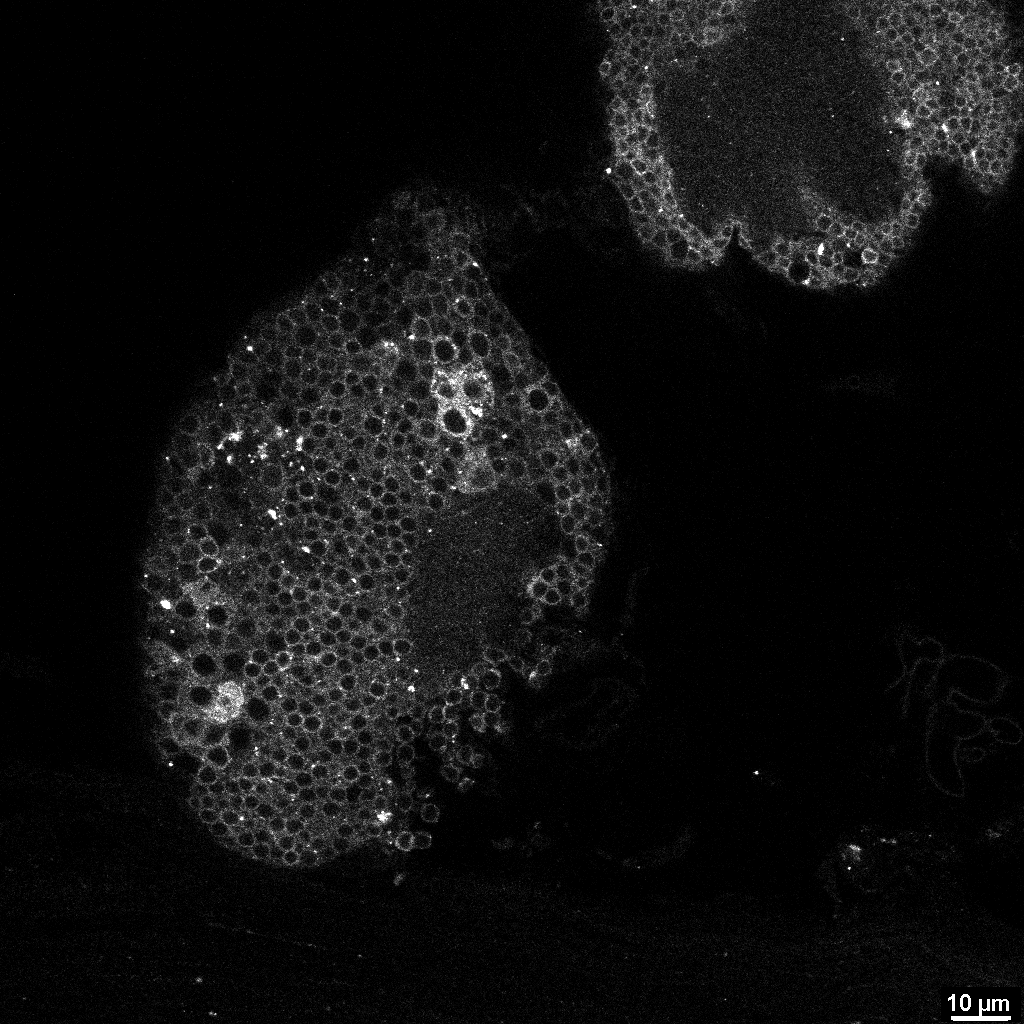

Supplement: Supplementary file 10 — Source data Fig. 7 [file 44319_2025_632_MOESM10_ESM.zip › Figure 7/7A/nSyb-GAL4 aSMase-IR v12227_inset.tif]

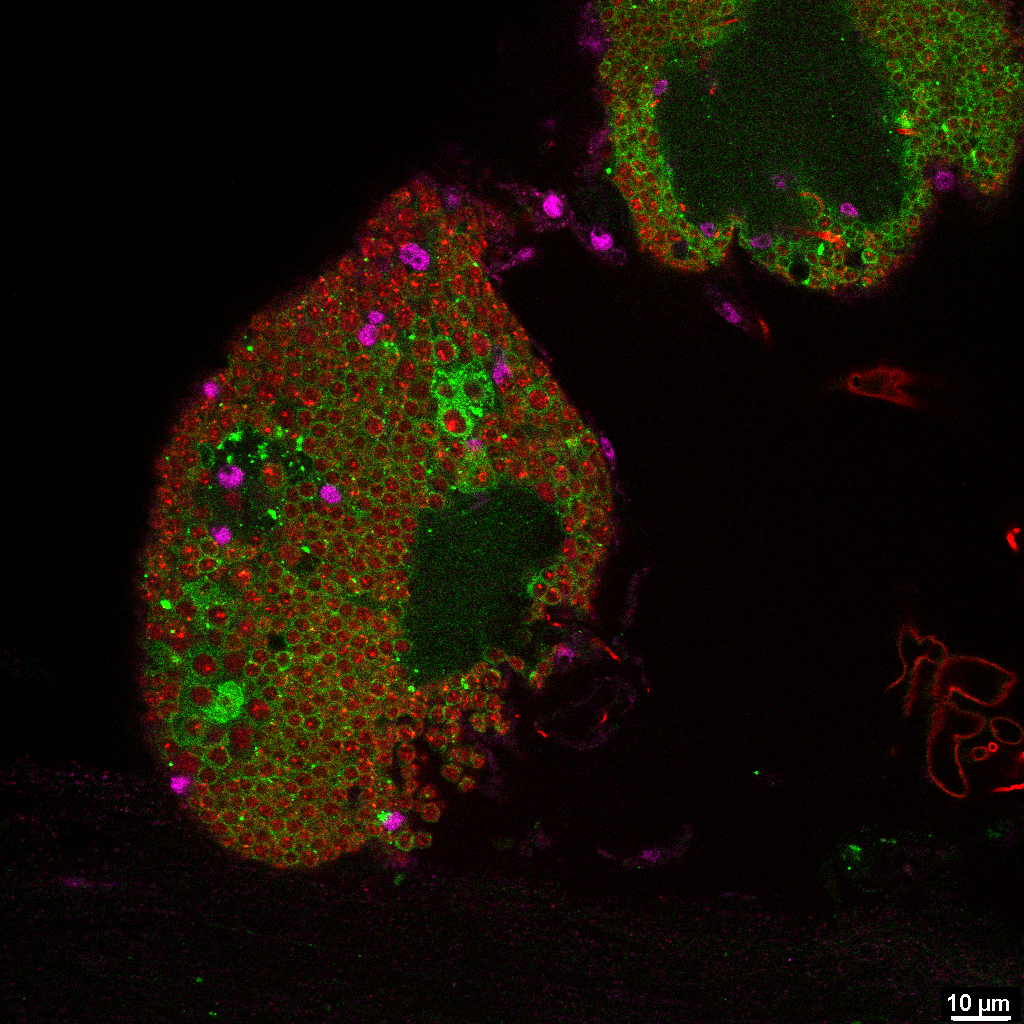

Supplement: Supplementary file 10 — Source data Fig. 7 [file 44319_2025_632_MOESM10_ESM.zip › Figure 7/7A/nSyb-GAL4 aSMase-IR v12227_Merge.tif]

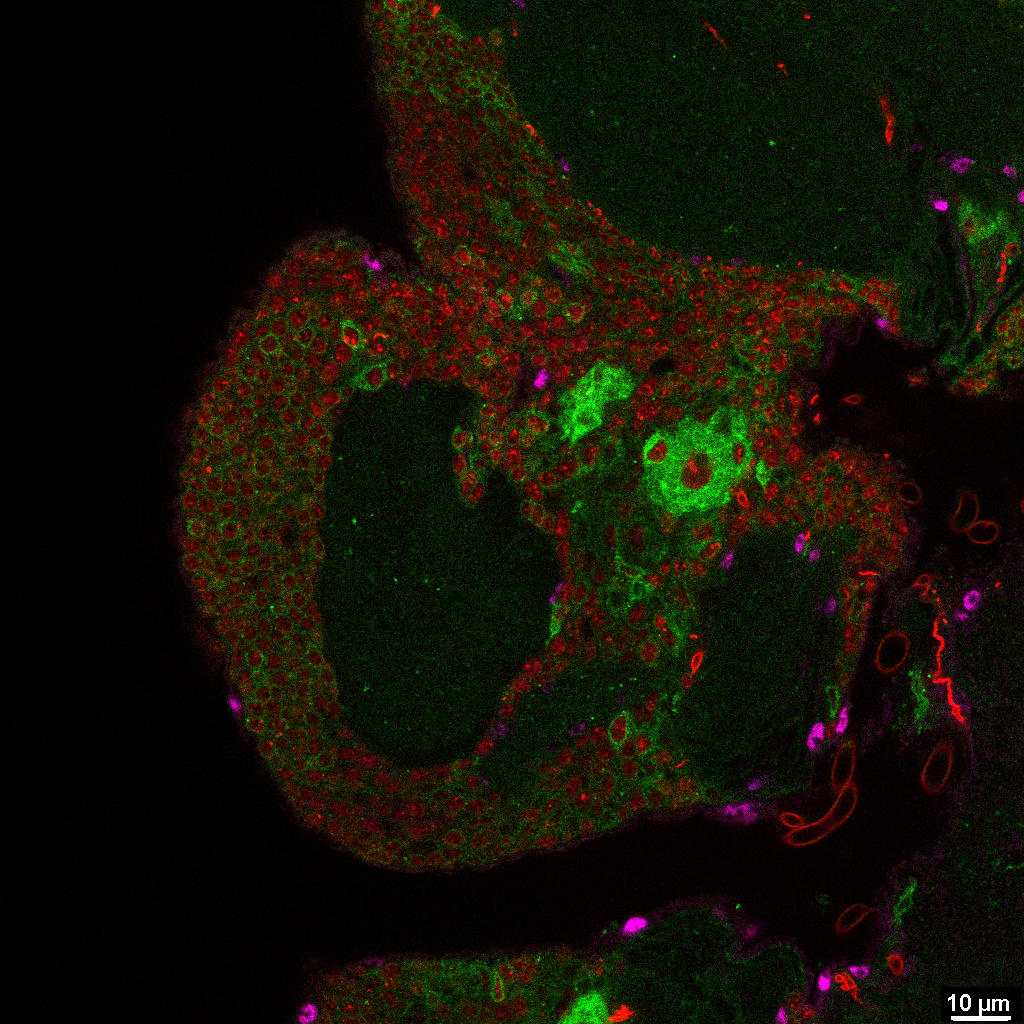

Supplement: Supplementary file 10 — Source data Fig. 7 [file 44319_2025_632_MOESM10_ESM.zip › Figure 7/7A/nSyb-GAL4 LUC-IR_Merge.tif]

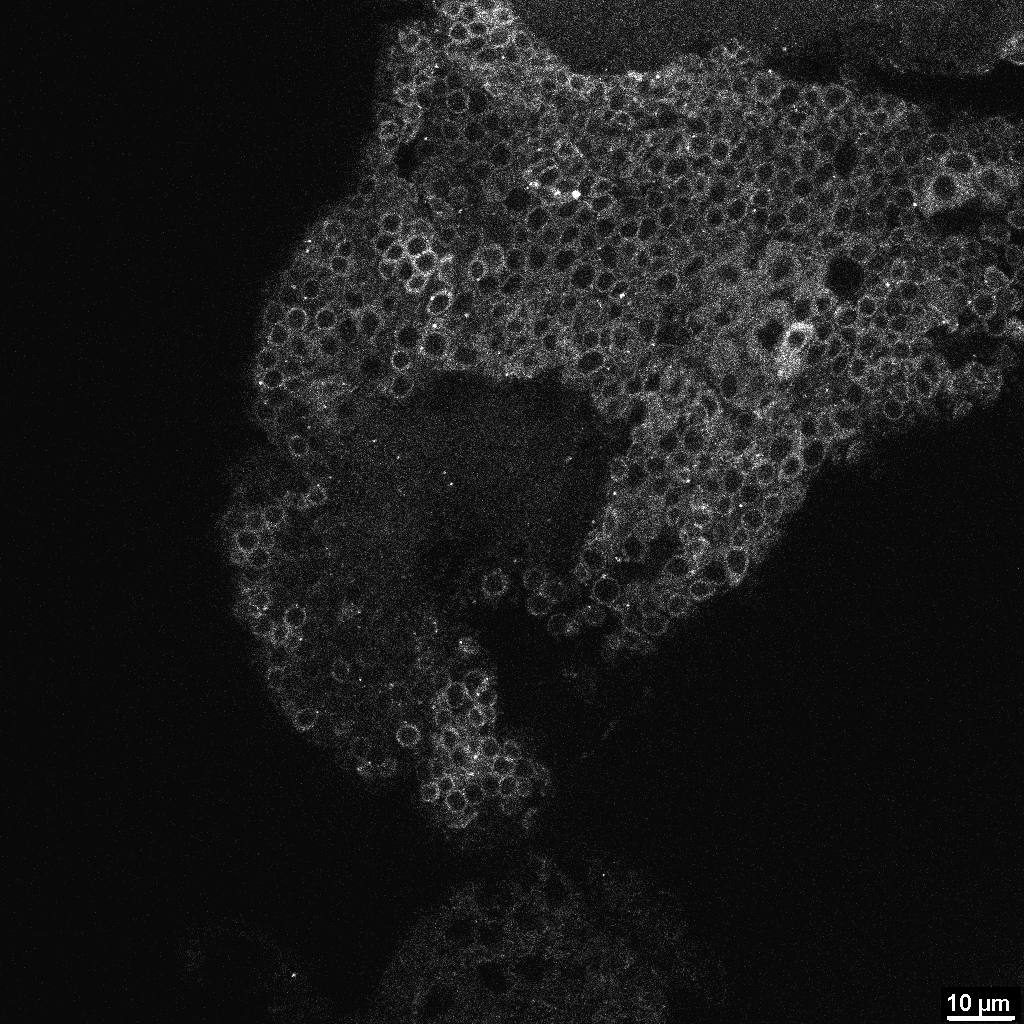

Supplement: Supplementary file 10 — Source data Fig. 7 [file 44319_2025_632_MOESM10_ESM.zip › Figure 7/7B/repo-GAL4 dSMPD4-IR BL51682_inset.tif]

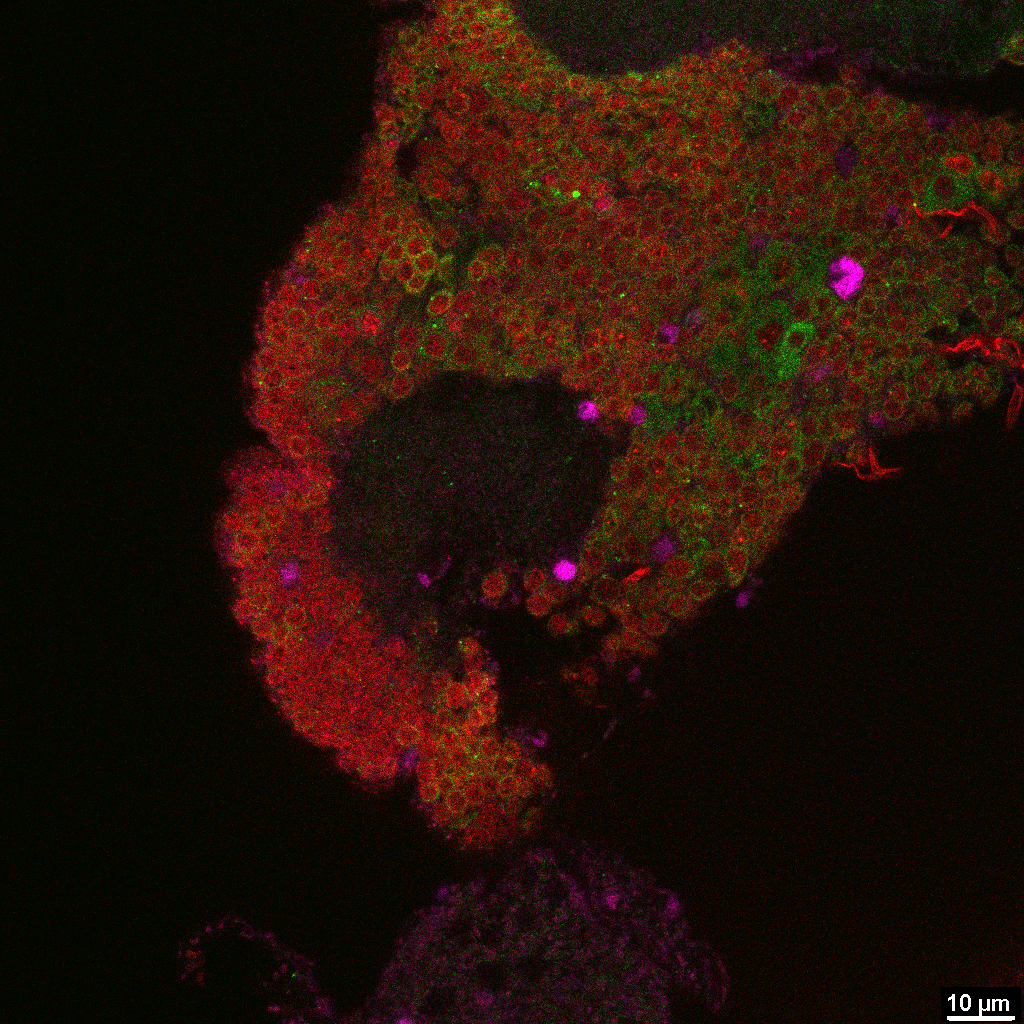

Supplement: Supplementary file 10 — Source data Fig. 7 [file 44319_2025_632_MOESM10_ESM.zip › Figure 7/7B/repo-GAL4 dSMPD4-IR BL51682_Merge.tif]

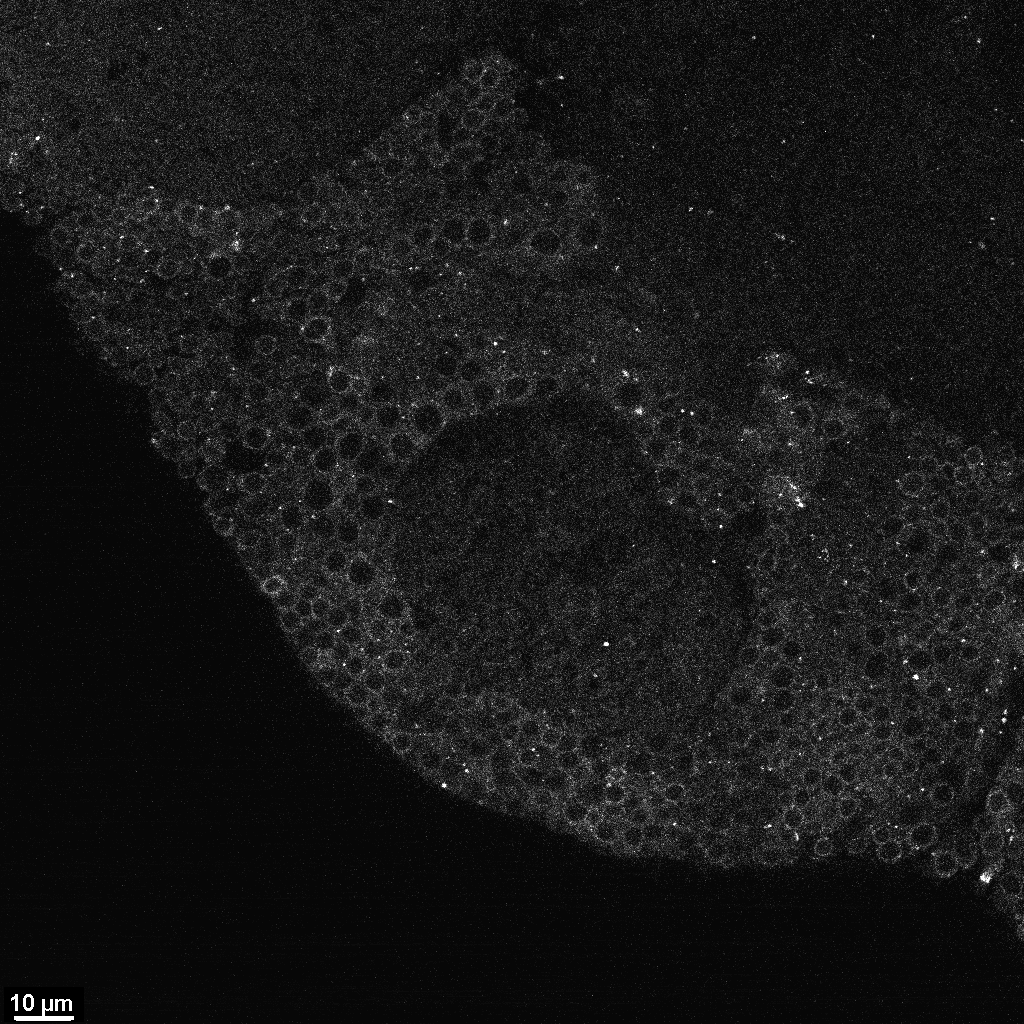

Supplement: Supplementary file 10 — Source data Fig. 7 [file 44319_2025_632_MOESM10_ESM.zip › Figure 7/7B/repo-GAL4 dSMPD4-IR v110163_inset.tif]

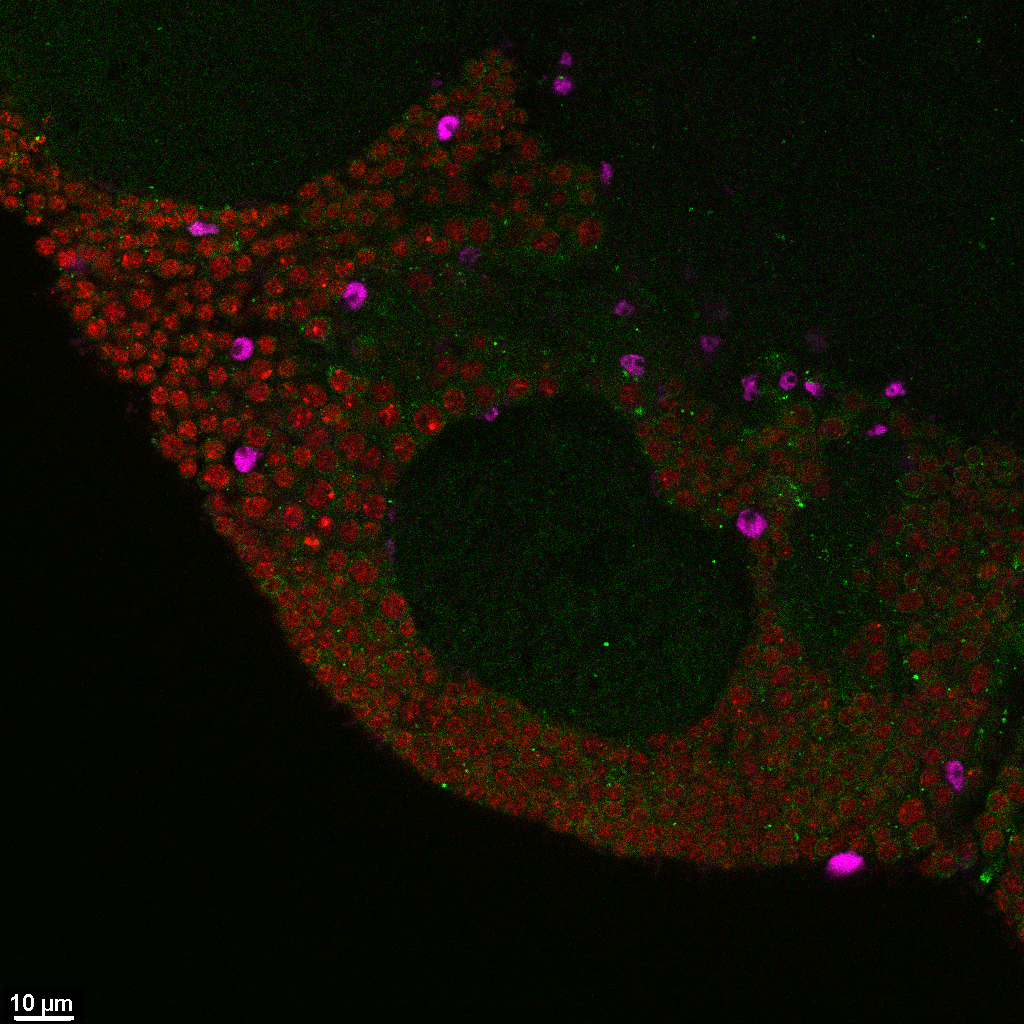

Supplement: Supplementary file 10 — Source data Fig. 7 [file 44319_2025_632_MOESM10_ESM.zip › Figure 7/7B/repo-GAL4 dSMPD4-IR v110163_Merge.tif]

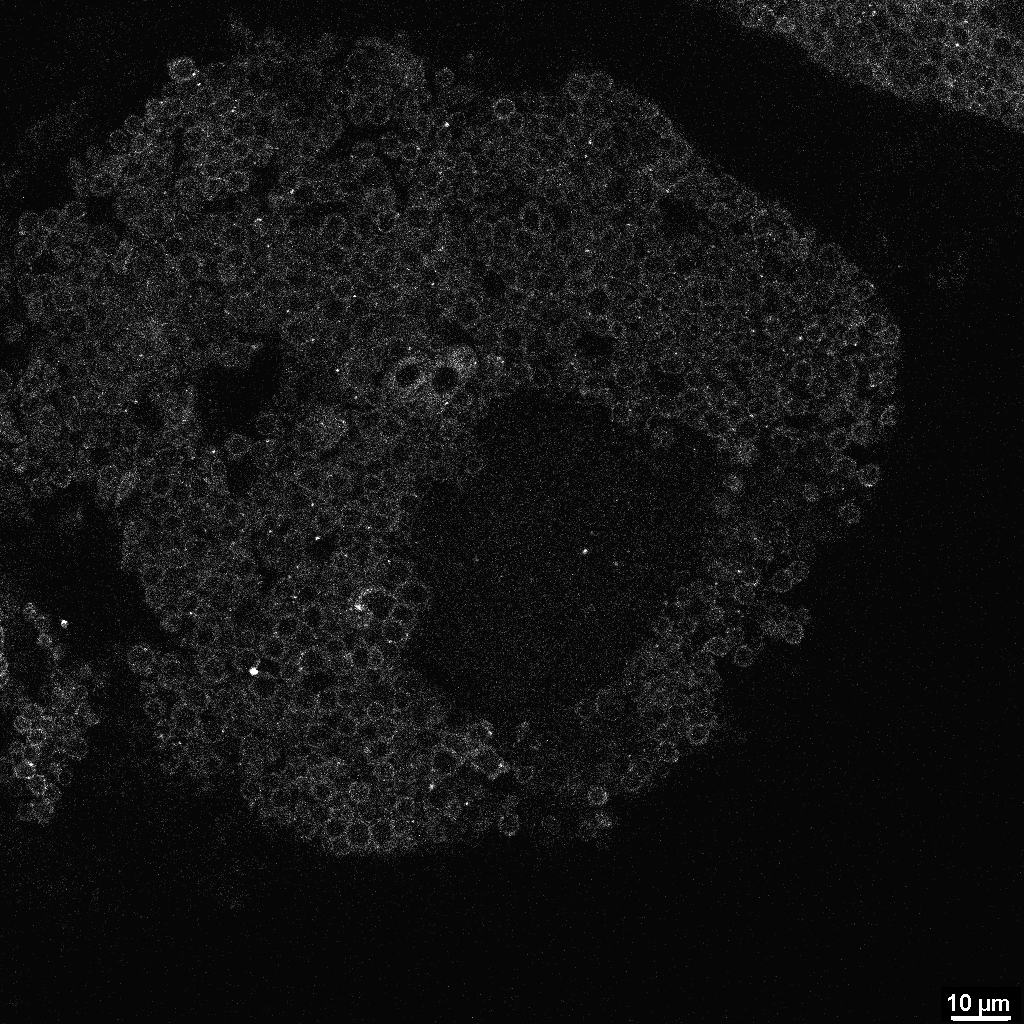

Supplement: Supplementary file 10 — Source data Fig. 7 [file 44319_2025_632_MOESM10_ESM.zip › Figure 7/7B/repo-GAL4 nSMase-IR v107062_inset.tif]

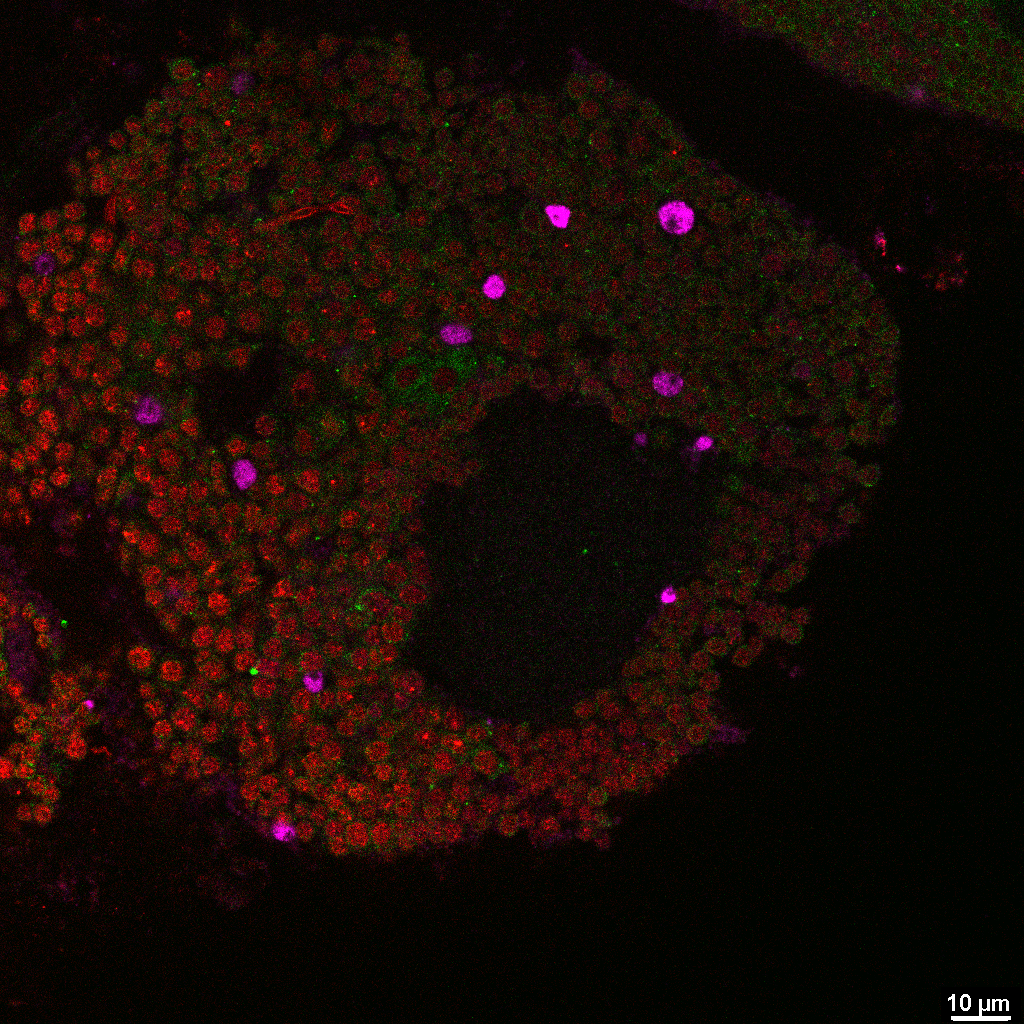

Supplement: Supplementary file 10 — Source data Fig. 7 [file 44319_2025_632_MOESM10_ESM.zip › Figure 7/7B/repo-GAL4 nSMase-IR v107062_Merge.tif]

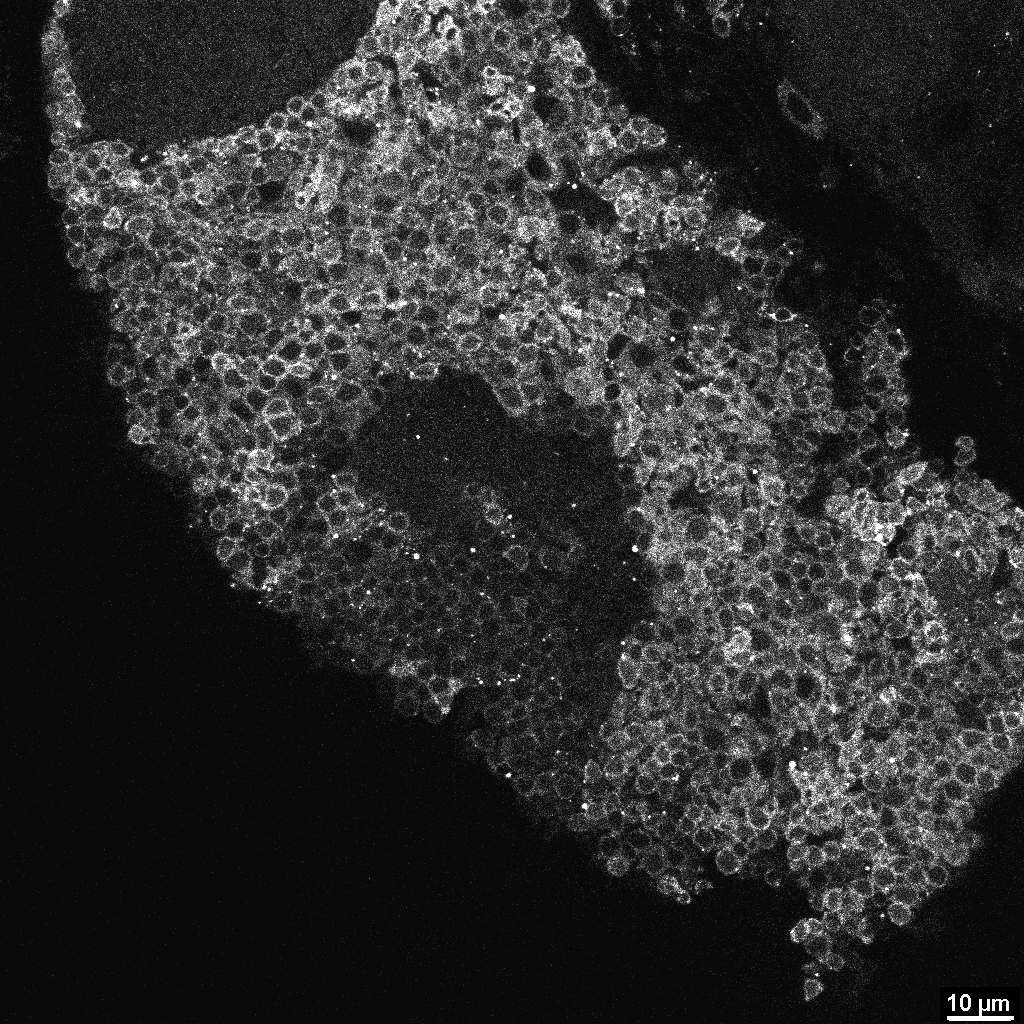

Supplement: Supplementary file 10 — Source data Fig. 7 [file 44319_2025_632_MOESM10_ESM.zip › Figure 7/7B/repo-GAL4 aSMase-IR v12227_inset.tif]

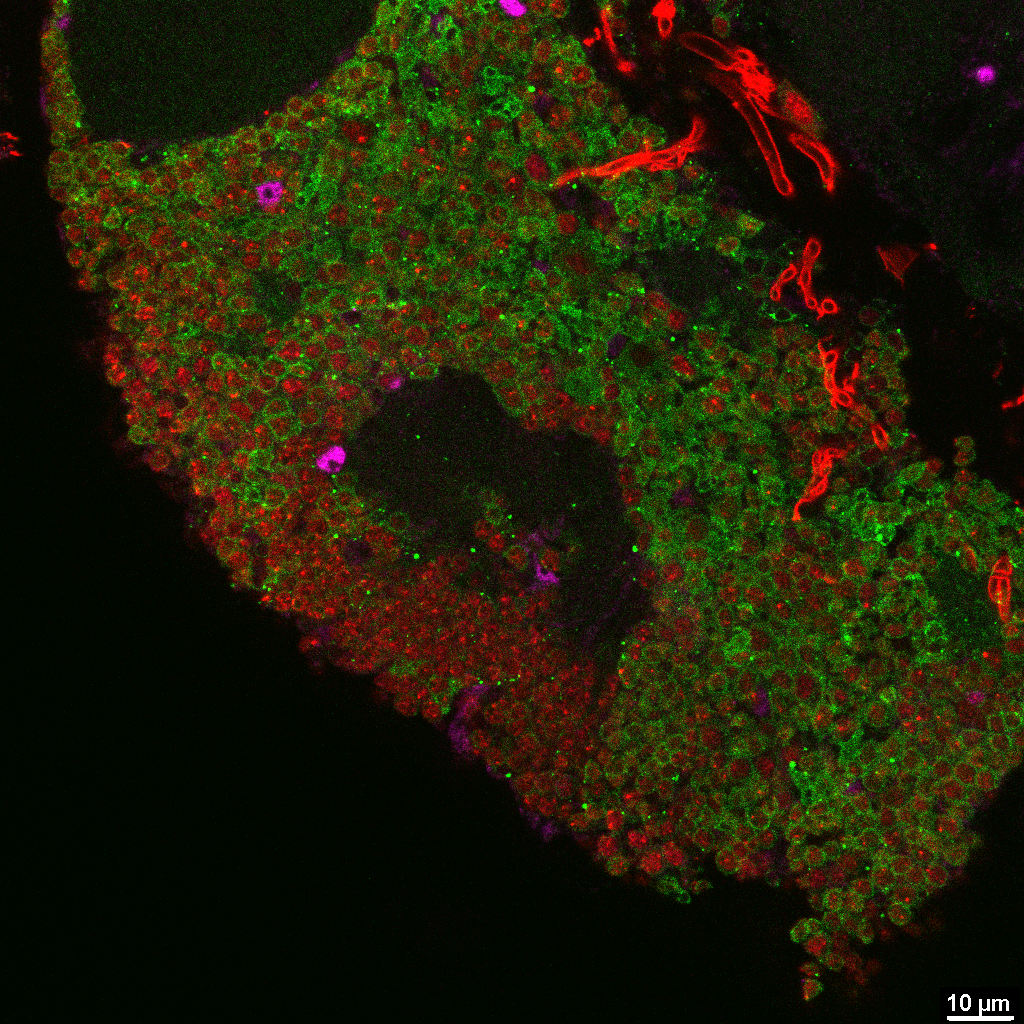

Supplement: Supplementary file 10 — Source data Fig. 7 [file 44319_2025_632_MOESM10_ESM.zip › Figure 7/7B/repo-GAL4 aSMase-IR v12227_Merge.tif]

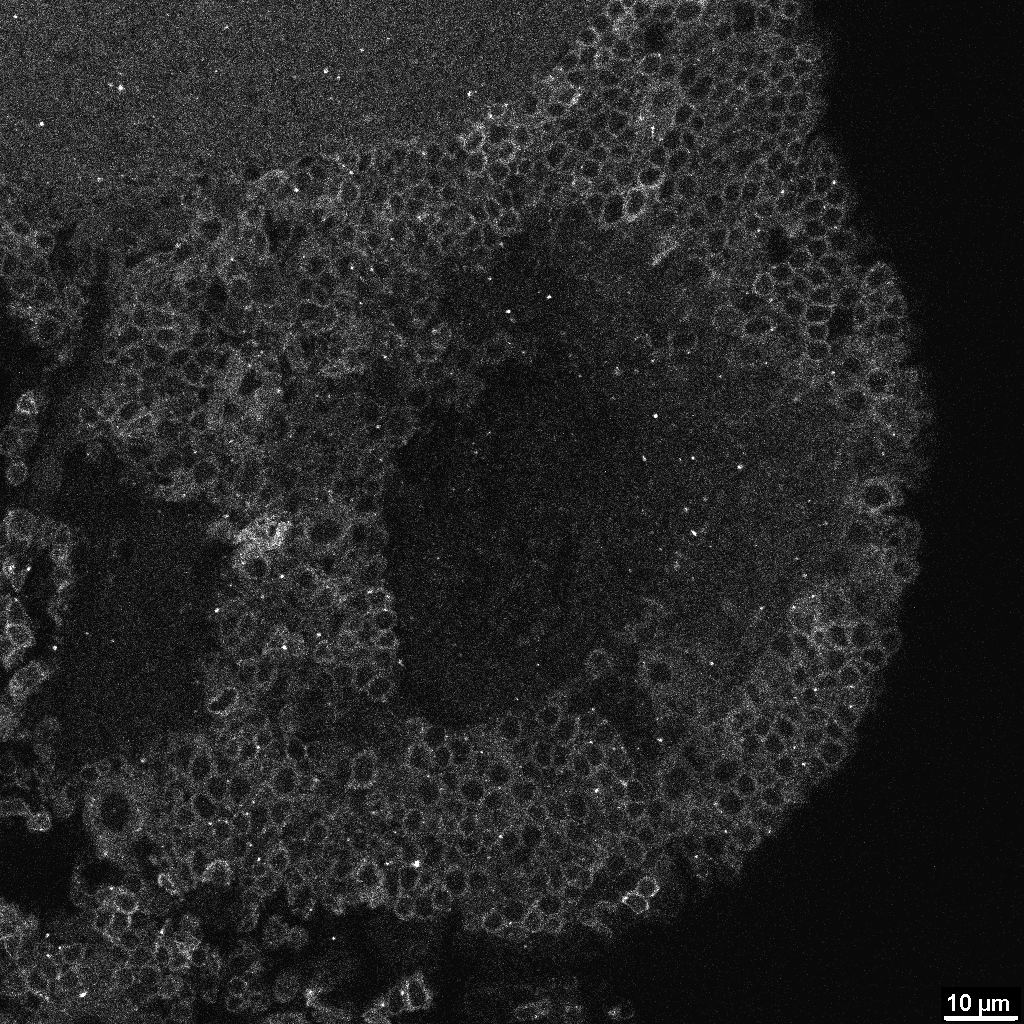

Supplement: Supplementary file 10 — Source data Fig. 7 [file 44319_2025_632_MOESM10_ESM.zip › Figure 7/7B/repo-GAL4 LUC-IR_inset.tif]
