# Supplementary figures and images for: Functional profiling and visualization of the sphingolipid metabolic network in vivo (part 2 of 2)
Source: EMBO Rep. 2025 Nov 10;26(24):6380–417. doi: 10.1038/s44319-025-00632-0 (PMC12714868; doi:10.1038/s44319-025-00632-0)

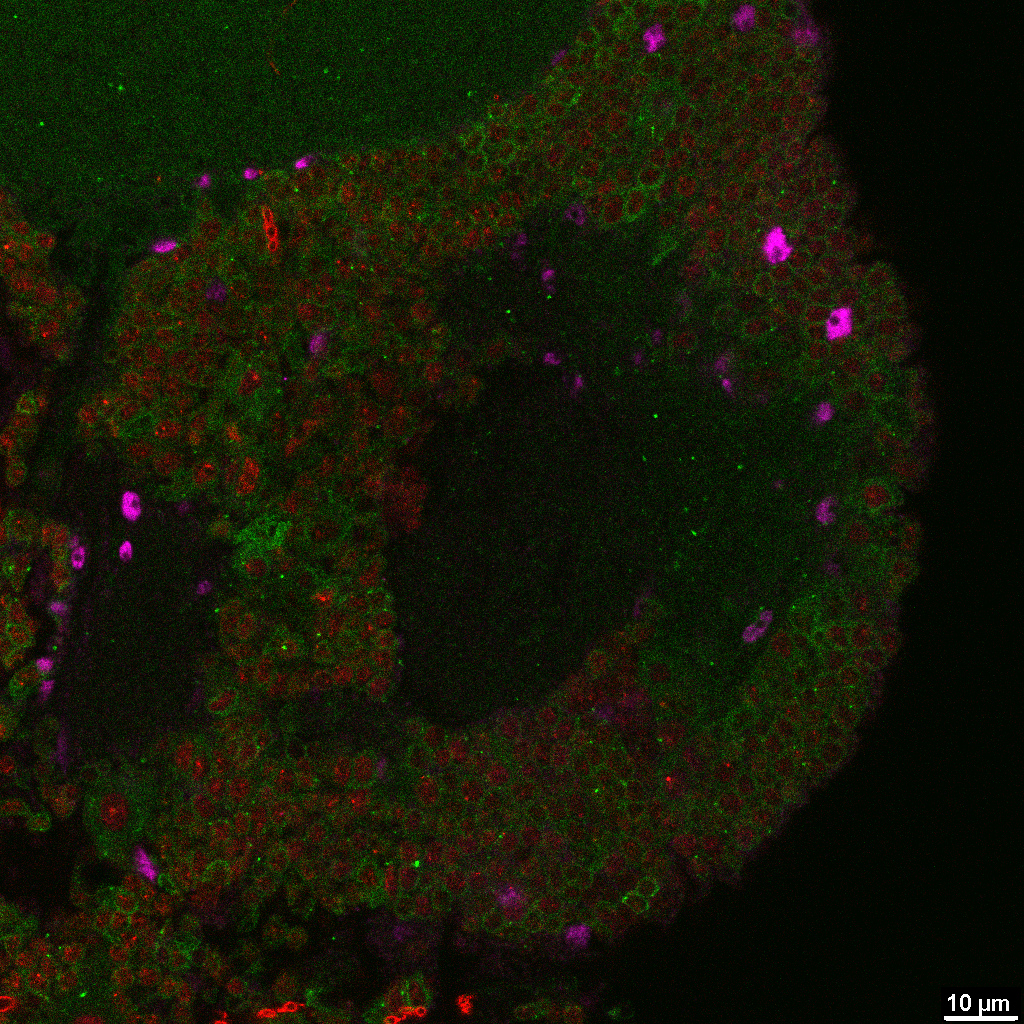

Supplement: Supplementary file 10 — Source data Fig. 7 [file 44319_2025_632_MOESM10_ESM.zip › Figure 7/7B/repo-GAL4 LUC-IR_Merge.tif]

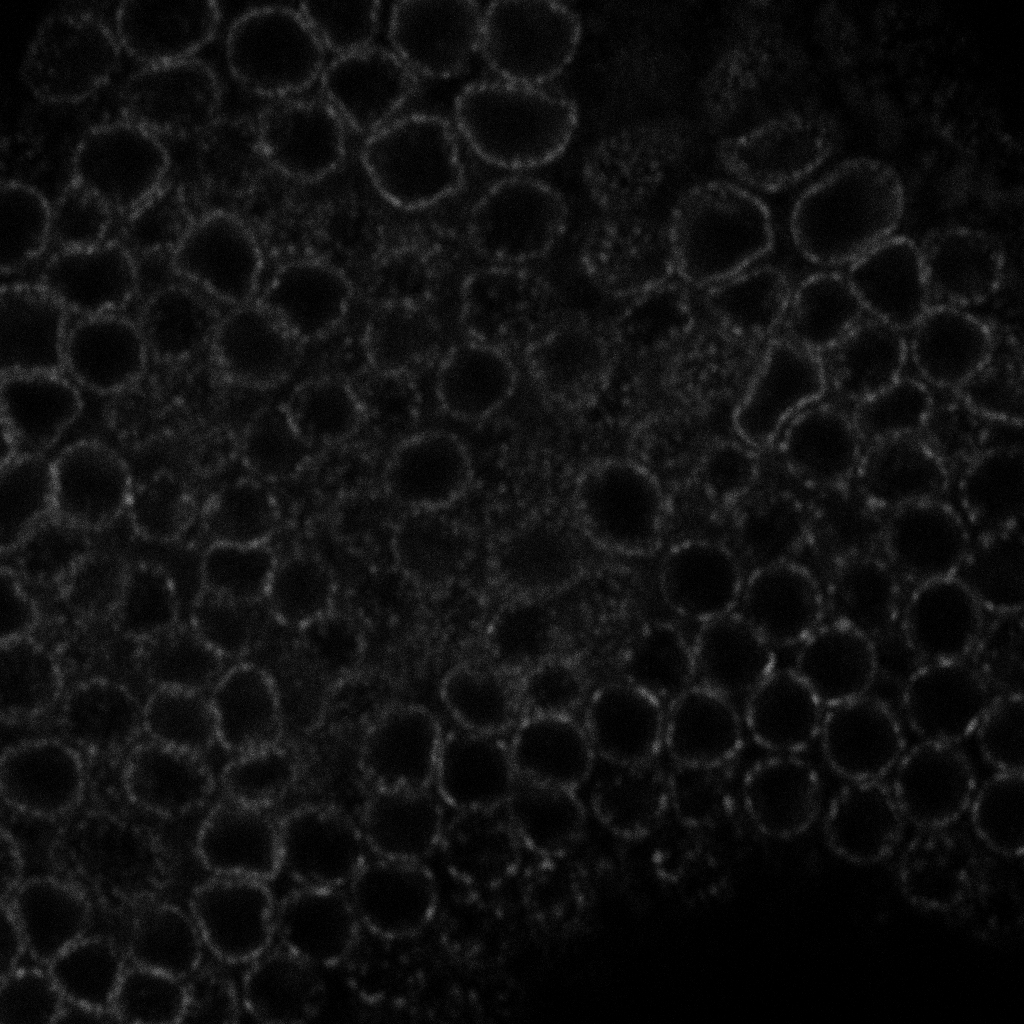

Supplement: Supplementary file 10 — Source data Fig. 7 [file 44319_2025_632_MOESM10_ESM.zip › Figure 7/7F/anti-HA.tif]

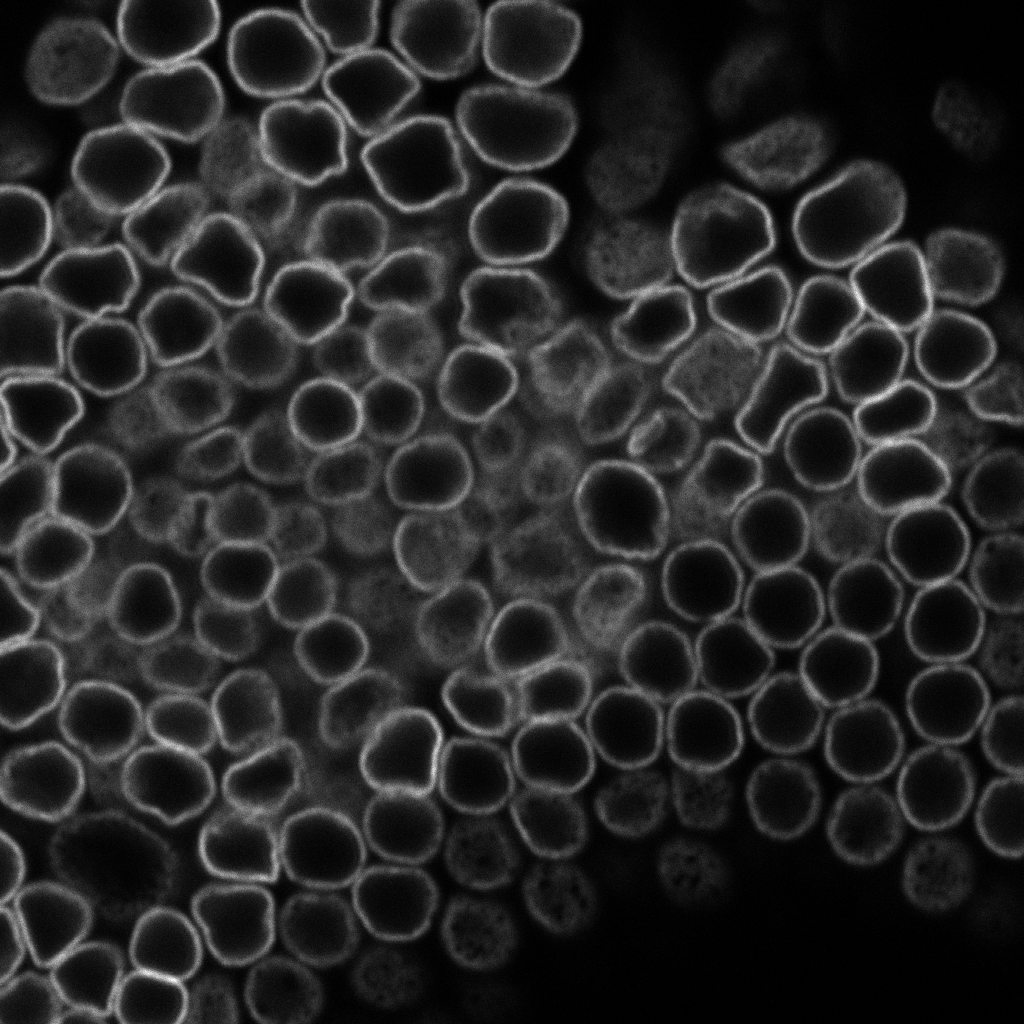

Supplement: Supplementary file 10 — Source data Fig. 7 [file 44319_2025_632_MOESM10_ESM.zip › Figure 7/7F/anti-Lamin.tif]

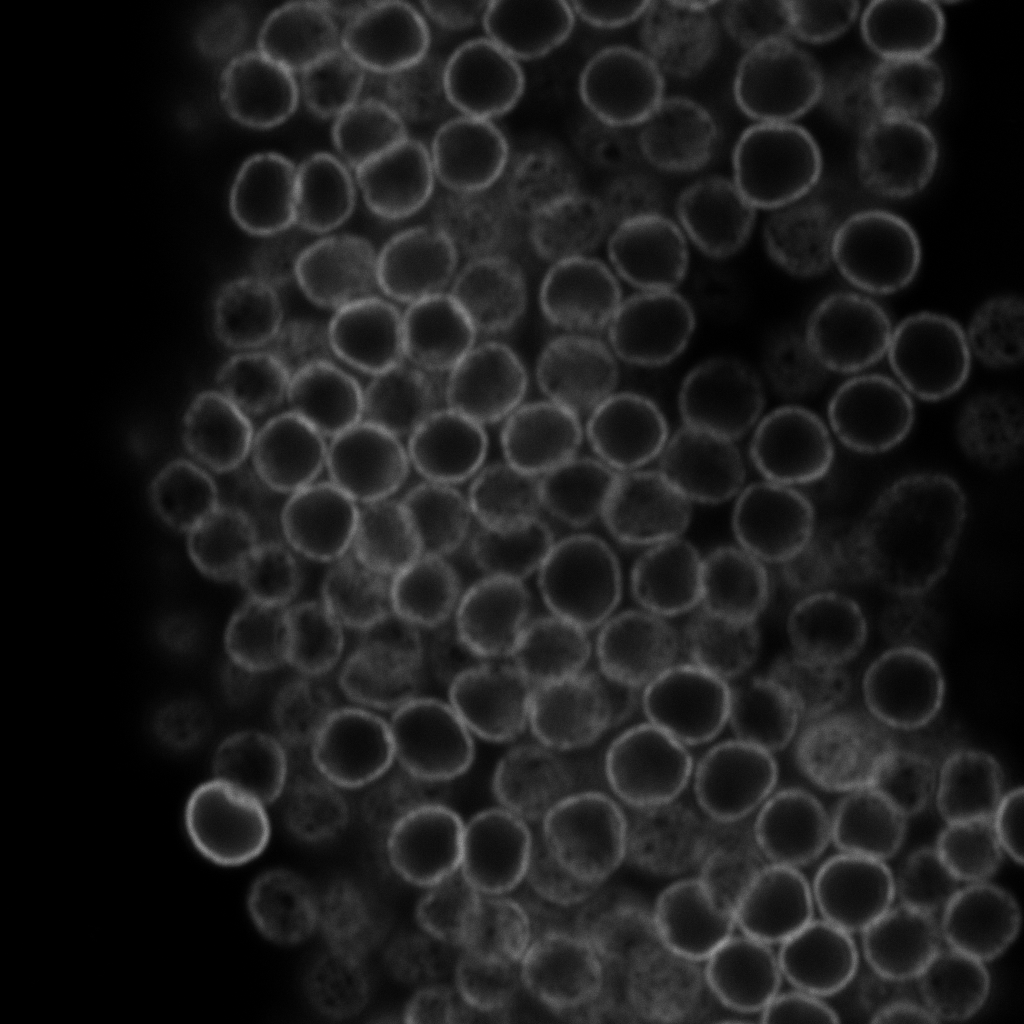

Supplement: Supplementary file 10 — Source data Fig. 7 [file 44319_2025_632_MOESM10_ESM.zip › Figure 7/7H/nSMaseKO Lamin.tif]

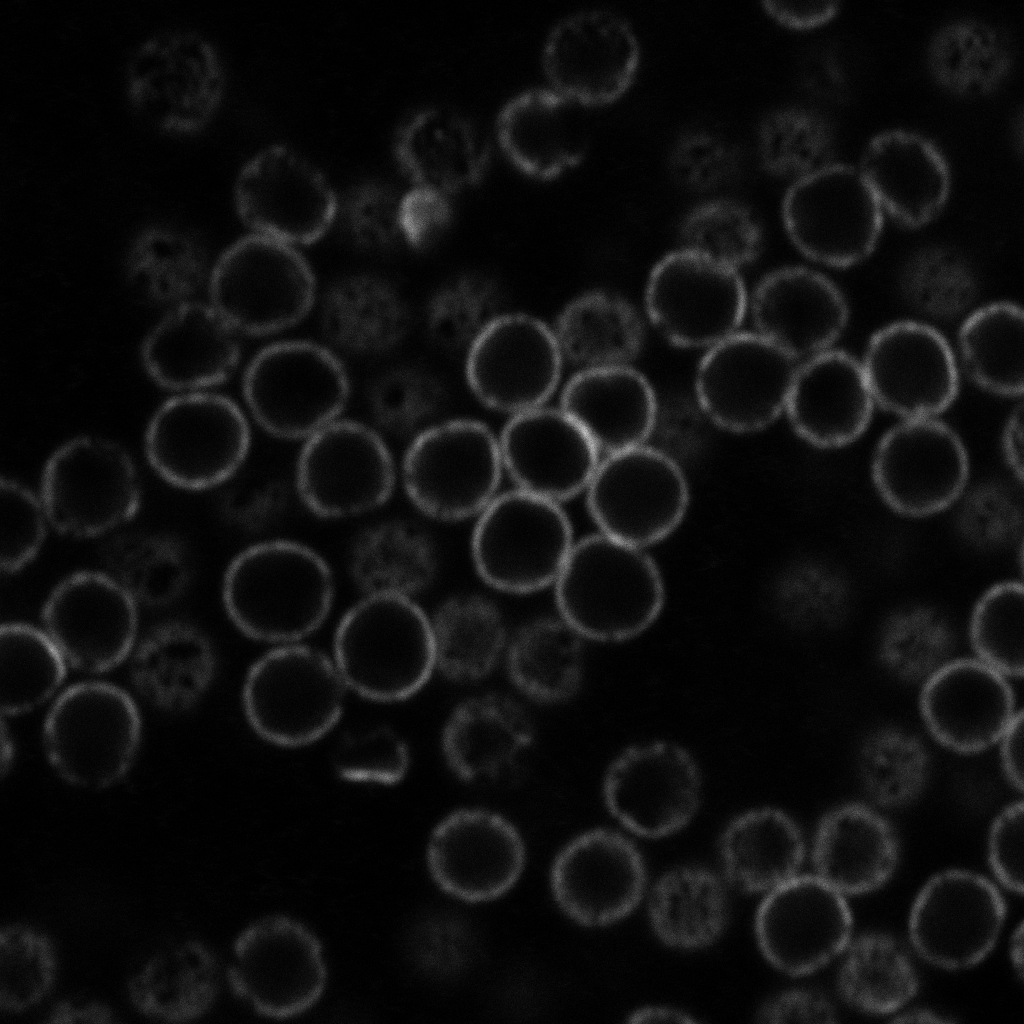

Supplement: Supplementary file 10 — Source data Fig. 7 [file 44319_2025_632_MOESM10_ESM.zip › Figure 7/7H/w1118 Lamin.tif]

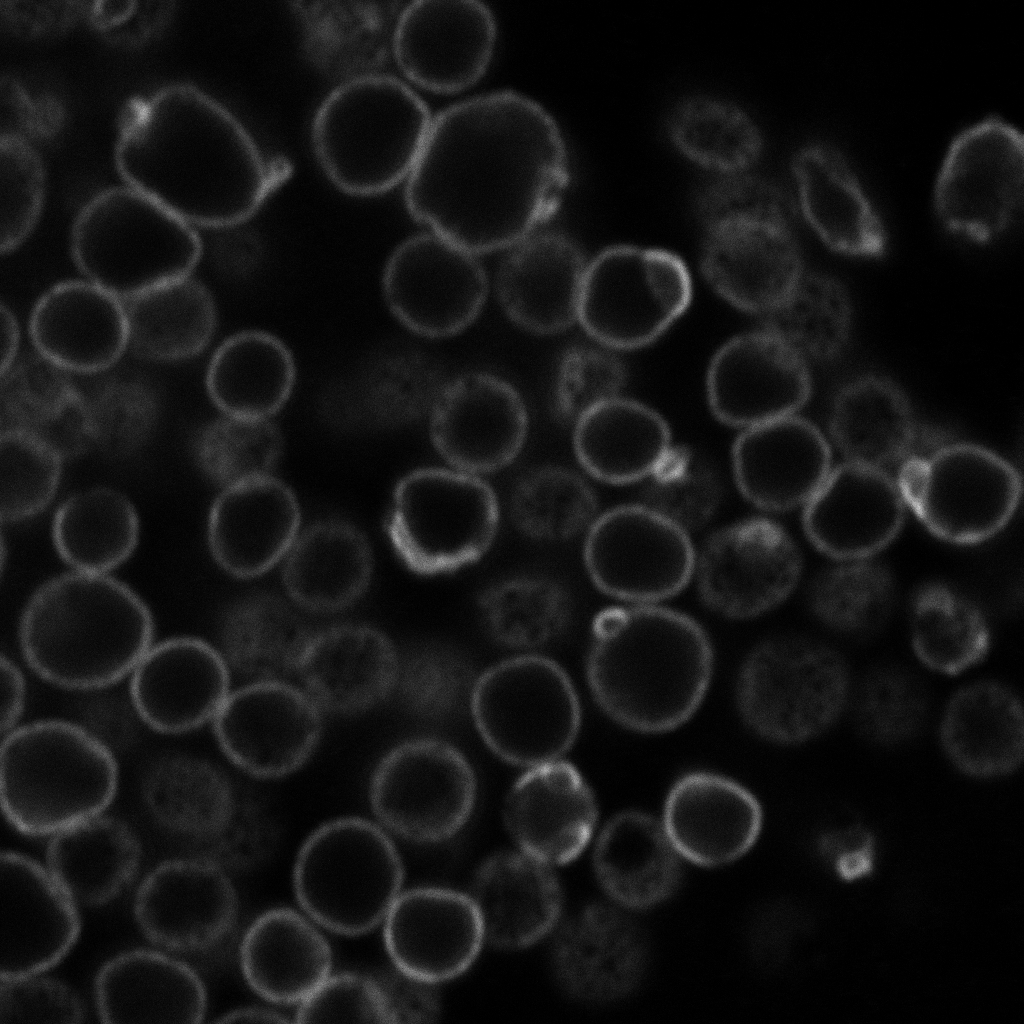

Supplement: Supplementary file 10 — Source data Fig. 7 [file 44319_2025_632_MOESM10_ESM.zip › Figure 7/7H/dSMPD4KO Lamin.tif]

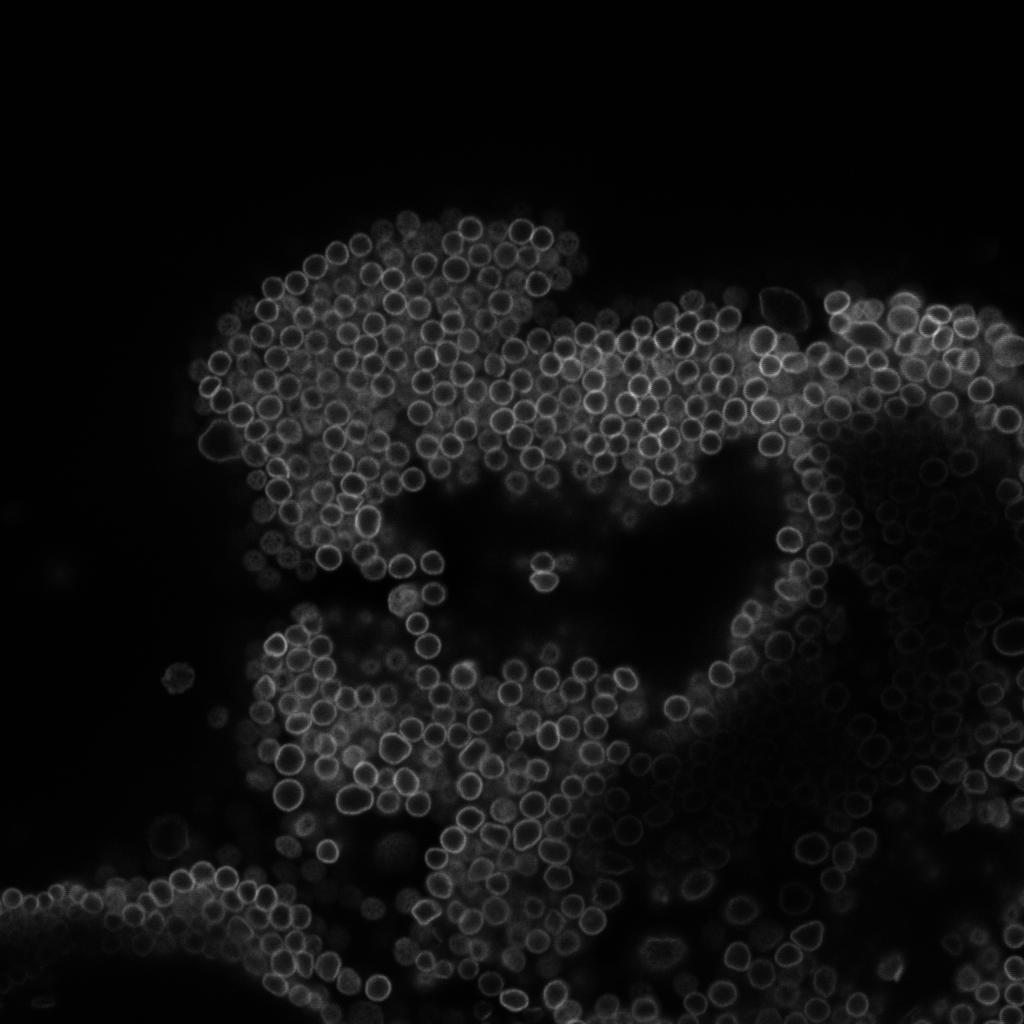

Supplement: Supplementary file 10 — Source data Fig. 7 [file 44319_2025_632_MOESM10_ESM.zip › Figure 7/7H/dSMPD4KO actin-dSMPD4-myc Lamin.tif]
