# Supplementary material for: Functional profiling and visualization of the sphingolipid metabolic network in vivo
Source: EMBO Rep. 2025 Nov 10;26(24):6380–417. doi: 10.1038/s44319-025-00632-0 (PMC12714868; doi:10.1038/s44319-025-00632-0)
Supplement: Supplementary file 11 — Expanded View Figures [file 44319_2025_632_MOESM11_ESM.pdf]

Expanded View Figures

Figure EV1. Validation of GAL4 expression in 3XHA-T2A-GAL4 (HG) knock-in flies (related to Fig. 2).

(**A**) Anti-HA and anti-Cnx99A (ER membrane marker) co-stainings of heterozygous *schlank*-HG L3 salivary glands. (Left) The colocalization of anti-HA and anti-Cnx99A indicated ER localization of the Schlank-3XHA protein. (Right) Punctate anti-HA signals in the nucleus (circled by dashed line) indicate nuclear localization of Schlank-3XHA protein. (**B**) Comparison of *gba1b*-HG and *gba1b* CRIMIC-GAL4 expressions. The GAL4 expressions in the adult brain of 1-week-old flies are visualized with UAS-nls-mCherry, and their colocalization with a glial marker (anti-Repo; Cyan) indicates glia-enriched expression of *gba1b*. Quantification and statistics of cell-type expressions are presented in Fig. EV2B,C. (**C**) Quantification and statistical analyses of numbers of nls-mCherry-positive cells. Bar graphs showed the number of nls-mCherry-positive cells in the central brains (1-week-old adult flies) of HG lines before and after Cre-mediated excision and CRIMIC-GAL4s of *CDase*, *gba1b*, and *sk1*. Data were representative of at least 2 independent experiments. Dots represent individual brains. Data are represented as mean ± SEM ($n \geq 3$). P values (CDase-HG Before Cre vs. CDase-HG After Cre, $P = 0.852$; CDase-HG Before Cre vs. CDase-CRIMIC, $P = 0.034$; CDase-HG After Cre vs. CDase-CRIMIC, $P = 0.007$; gba1b-HG Before Cre vs. gba1b-HG After Cre, $P = 0.869$; gba1b-HG Before Cre vs. gba1b-CRIMIC, $P = 0.004$; gba1b-HG After Cre vs. gba1b-CRIMIC, $P = 0.008$; sk1-HG Before Cre vs. sk1-HG After Cre, $P = 0.189$; sk1-HG Before Cre vs. sk1-HG CRIMIC, $P = 0.002$; sk1-HG After Cre vs. sk1-CRIMIC, $P = 0.006$) were calculated using one-way ANOVA with Dunnett's multiple comparisons. (**D**) Quantification and statistical analyses of nls-mCherry colocalized to neuronal and glia markers. Bar graphs showed the percentage of nls-mCherry spots colocalizing to neuronal nuclei (anti-Elav; black bars) and glial nuclei (anti-Repo; white bars) in the total number of labeled nls-mCherry (colocalizing with Elav or Repo). The percentage of anti-Elav (88.21%) or anti-Repo (11.79%) spots in the total number of labeled nuclei (the sum of anti-Elav and anti-Repo spots) is shown on the left, and the red dashed line indicates the percentage of anti-Elav spots. Data were presented as mean percentages of at least two independent experiments. P values (sk1-HG Before Cre, $P < 0.001$; sk1-HG After Cre, $P < 0.001$; sk1-CRIMIC, $P < 0.001$; gba1b-HG before Cre, $P < 0.001$; gba1b-HG After Cre, $P < 0.001$; gba1b-CRIMIC, $P < 0.001$; CDase-HG Before Cre, $P < 0.001$; CDase-HG After Cre, $P < 0.001$; CDase-CRIMIC, $P = 0.009$) were calculated using two-tailed unpaired Student's t-test, comparing the means between the percentage of nls-mCherry colocalizing with Elav in the total number of labeled nls-mCherry (the sum of Elav- and Repo-colocalizing nls-mCherry spots) and the percentage of Elav spots in the total number of labeled nuclei (the sum of Elav and Repo spots).

© The Author(s)

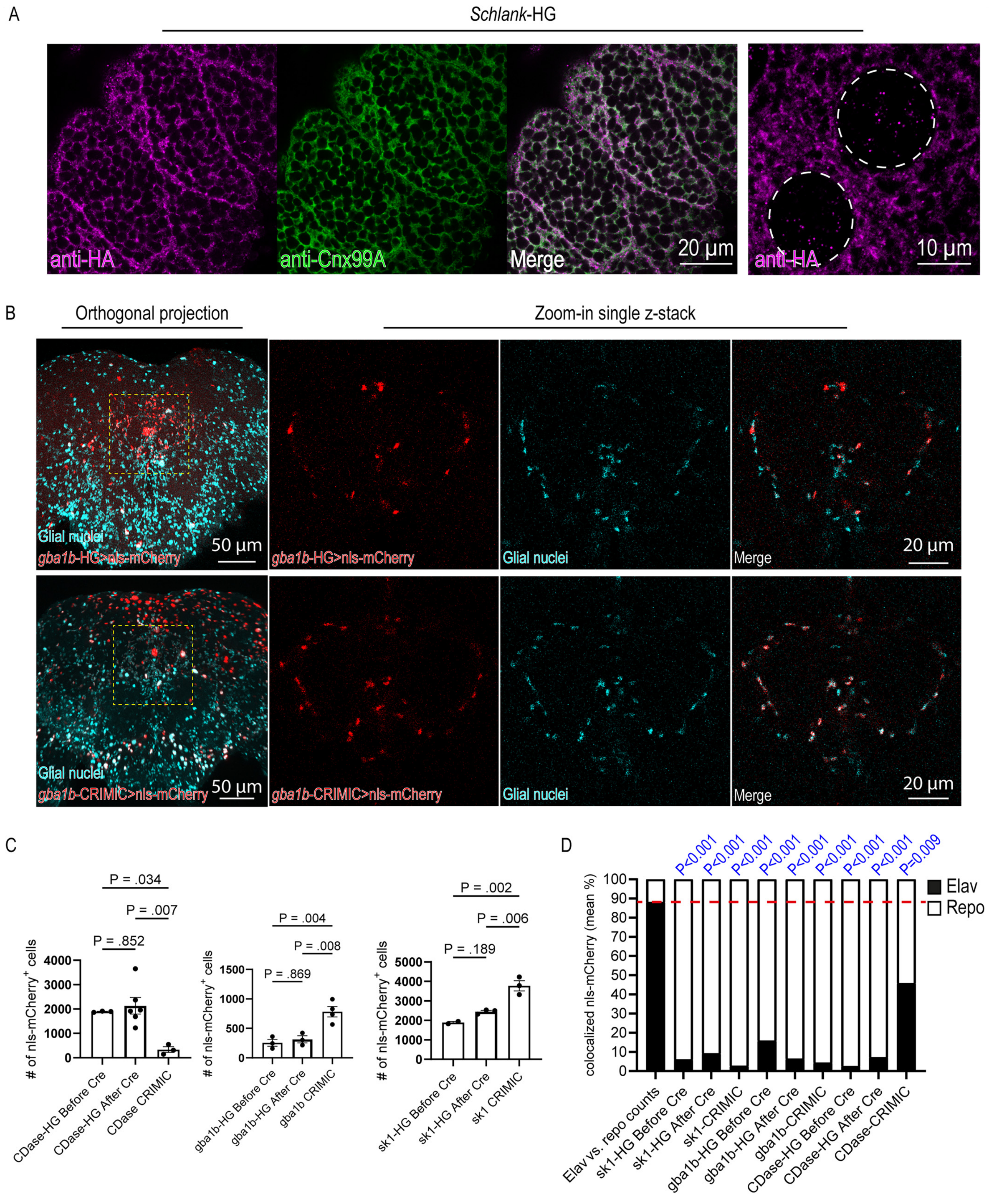
A
Schlank-HG
anti-HA
anti-Cnx99A
Merge
20 µm
anti-HA
10 µm
B
Orthogonal projection
Zoom-in single z-stack
Glial nuclei
gba1b-HG>nls-mCherry
50 µm
gba1b-HG>nls-mCherry
Glial nuclei
Merge
20 µm
Glial nuclei
gba1b-CRIMIC>nls-mCherry
50 µm
gba1b-CRIMIC>nls-mCherry
Glial nuclei
Merge
20 µm
C
P = .034
P = .007
P = .852
# of nls-mCherry+ cells
CDase-HG Before Cre
CDase-HG After Cre
CDase CRIMIC
P = .004
P = .008
P = .869
gba1b-HG Before Cre
gba1b-HG After Cre
gba1b CRIMIC
P = .002
P = .006
P = .189
sk1-HG Before Cre
sk1-HG After Cre
sk1 CRIMIC
D
P<0.001
P<0.001
P<0.001
P<0.001
P<0.001
P<0.001
P<0.001
P<0.001
P=0.009
Elav
Repo
colocalized nls-mCherry (mean %)
Elav vs. repo counts
sk1-HG Before Cre
sk1-HG After Cre
sk1-CRIMIC
gba1b-HG Before Cre
gba1b-HG After Cre
gba1b-CRIMIC
CDase-HG Before Cre
CDase-HG After Cre
CDase-CRIMIC

 © The Author(s)

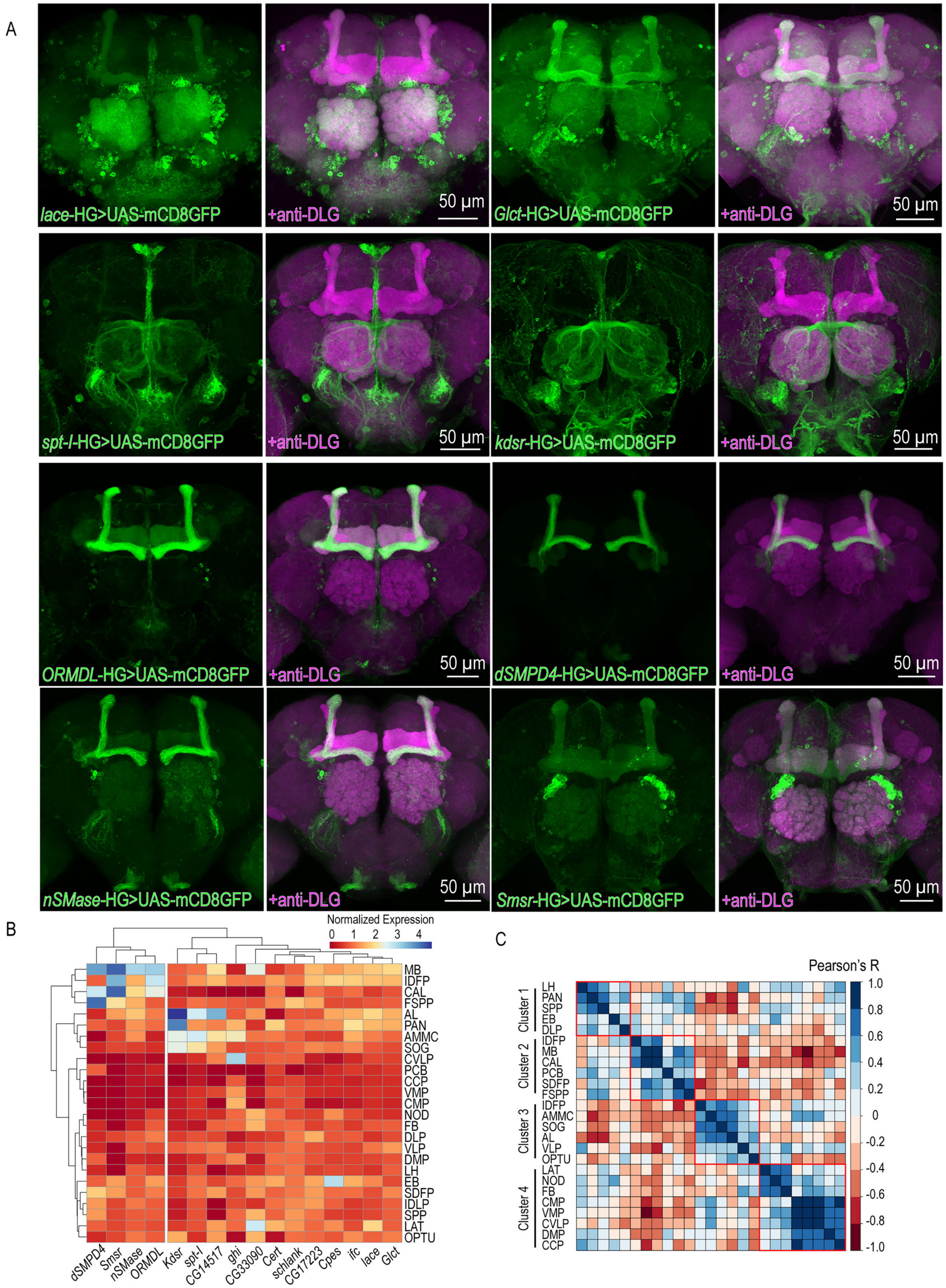
A
lace-HG>UAS-mCD8GFP
+anti-DLG
50 μm
Glct-HG>UAS-mCD8GFP
+anti-DLG
50 μm
spt-I-HG>UAS-mCD8GFP
+anti-DLG
50 μm
kdsr-HG>UAS-mCD8GFP
+anti-DLG
50 μm
ORMDL-HG>UAS-mCD8GFP
+anti-DLG
50 μm
dSMPD4-HG>UAS-mCD8GFP
+anti-DLG
50 μm
nSMase-HG>UAS-mCD8GFP
+anti-DLG
50 μm
Smsr-HG>UAS-mCD8GFP
+anti-DLG
50 μm
B
Normalized Expression
0 1 2 3 4
MB
IDFP
CAL
FSPP
AL
PAN
AMMC
SOG
CVLP
PCB
CCP
VMP
CMP
NOD
FB
DLP
VLP
DMP
LH
EB
SDFP
IDLP
SPP
LAT
OPTU
dSMPD4
Smsr
nSMase
ORMDL
Kdsr
spt-I
CG14517
ghi
CG33090
Cert
schlank
CG17223
Cpes
ifc
lace
Glct
C
Pearson's R
1.0
0.8
0.6
0.4
0.2
0
-0.2
-0.4
-0.6
-0.8
-1.0
Cluster 1
LH
PAN
SPP
EB
DLP
Cluster 2
IDFP
MB
CAL
PCB
SDFP
FSPP
Cluster 3
IDFP
AMMC
SOG
AL
VLP
OPTU
Cluster 4
LAT
NOD
FB
CMP
VMP
CVLP
DMP
CCP

Figure EV2. Transcriptional profiling with HG revealed highly diverse expression patterns of SPL regulators in different brain regions (related to Fig. 3).

(**A**) The expression pattern of targeted genes in the central brain of young adult flies (1-week-old) is visualized with UAS-mCD8GFP (green) and neuropil staining (magenta; anti-DLG). (**B**) A hierarchical heatmap of SPL metabolism gene expression by genes (columns) and neuropils (rows). The mean value of mCD8GFP intensity of each gene in each neuropil was normalized to the mean value of mCD8GFP of each gene in all neuropils. Red indicates high expression, and blue indicates low expression. Rows and columns are clustered based on gene expression similarity. (**C**) Correlation matrix displays Pearson correlation coefficients between gene expression profiles of different neuropil. Blue indicates high positive correlation, Red indicates high negative correlation, and white indicates no correlation. Representative figures of mCD8GFP images of all SPL regulators are presented in Appendix Figs. S22–30. The data in (**B**, **C**) represent the mCD8GFP image of one brain for individual genes. The representative figure of the brain alignment is presented in Appendix Fig. S31.

 © The Author(s)

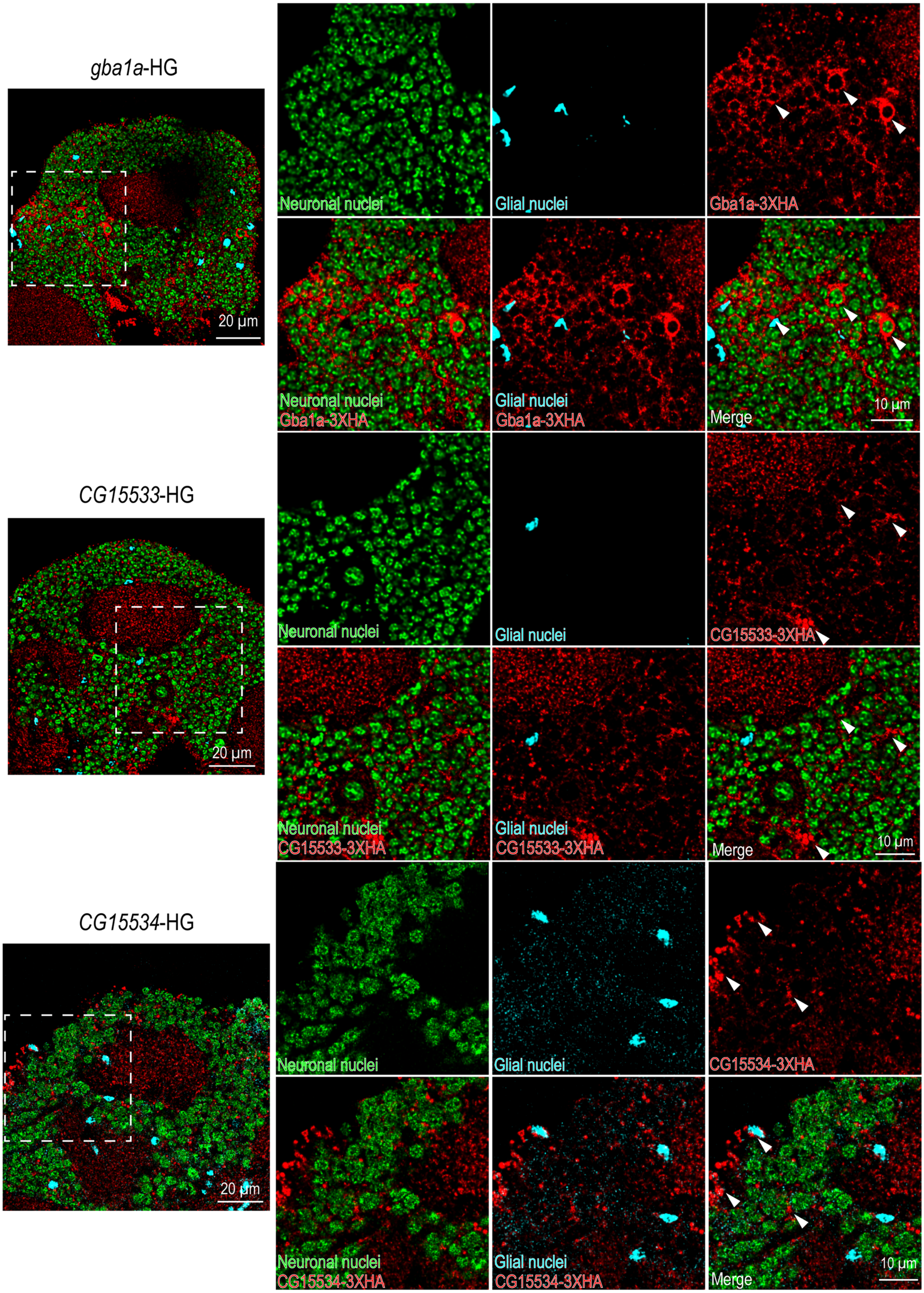
gba1a-HG
20 μm
Neuronal nuclei
Glial nuclei
Gba1a-3XHA
Neuronal nuclei
Gba1a-3XHA
Glial nuclei
Gba1a-3XHA
Merge
10 μm
CG15533-HG
20 μm
Neuronal nuclei
Glial nuclei
CG15533-3XHA
Neuronal nuclei
CG15533-3XHA
Glial nuclei
CG15533-3XHA
Merge
10 μm
CG15534-HG
20 μm
Neuronal nuclei
Glial nuclei
CG15534-3XHA
Neuronal nuclei
CG15534-3XHA
Glial nuclei
CG15534-3XHA
Merge
10 μm

◀ **Figure EV3. Anti-HA immunostainings of adult brains from HG lines *gba1a*, *CG15533*, *CG15534* (related to Fig. 4).**

The protein distribution of Gba1a-3XHA, CG15533-3XHA, and CG15534-3XHA are visualized by anti-HA immunostaining (red) with co-stainings of neuronal (green; anti-Elav) and glial (cyan; anti-Repo) nuclei of young adult brains (1-week-old). Arrowheads indicate cells showing positive anti-HA signals.

 © The Author(s)

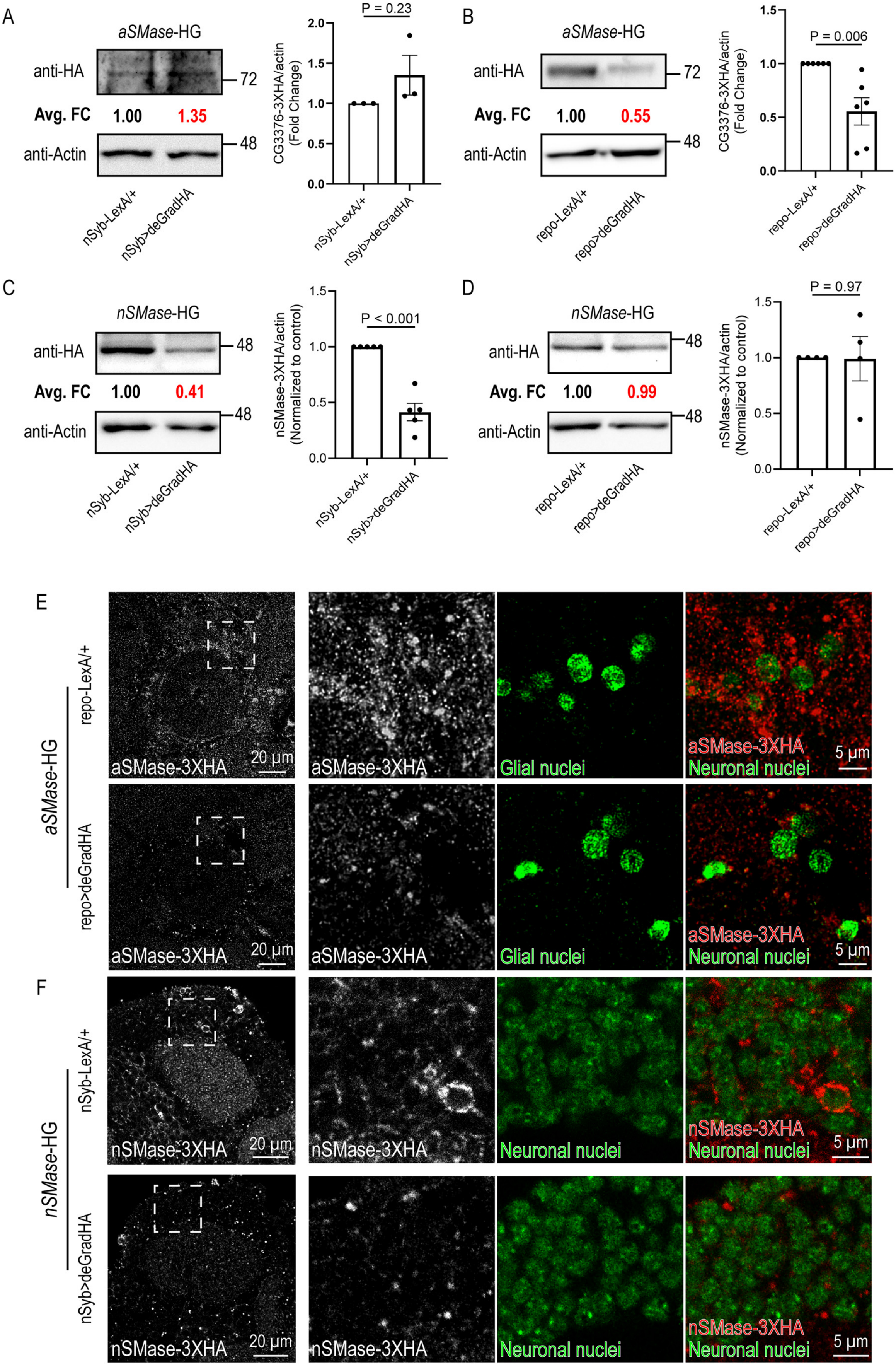
A
aSMase-HG
anti-HA
72
Avg. FC 1.00 1.35
anti-Actin
48
nSyb-LexA/+
nSyb>deGradHA
P = 0.23
CG3376-3XHA/actin (Fold Change)
B
aSMase-HG
anti-HA
72
Avg. FC 1.00 0.55
anti-Actin
48
repo-LexA/+
repo>deGradHA
P = 0.006
CG3376-3XHA/actin (Fold Change)
C
nSMase-HG
anti-HA
48
Avg. FC 1.00 0.41
anti-Actin
48
nSyb-LexA/+
nSyb>deGradHA
P < 0.001
nSMase-3XHA/actin (Normalized to control)
D
nSMase-HG
anti-HA
48
Avg. FC 1.00 0.99
anti-Actin
48
repo-LexA/+
repo>deGradHA
P = 0.97
nSMase-3XHA/actin (Normalized to control)
E
aSMase-HG
repo-LexA/+
repo>deGradHA
aSMase-3XHA
Glial nuclei
Neuronal nuclei
20 µm
5 µm
F
nSMase-HG
nSyb-LexA/+
nSyb>deGradHA
nSMase-3XHA
Neuronal nuclei
20 µm
5 µm

Figure EV4. The deGradHA induced cell type-specific protein degradation of aSMase-3XHA and nSMase-3XHA in the brain (related to Fig. 5).

(**A**) Representative immunoblot of the adult head extract of *aSMase*-HG line with (*aSMase*-HG/+; *nSyb*-LexA/*LexAop-deGradHA*) or without (*aSMase*-HG/+; *nSyb*-LexA/+) neuronal expression of deGradHA. The average fold change (Avg. FC) of aSMase-3XHA immunoreactivity normalized to loading control (anti-Actin) is shown in the figure. (Right) Quantification of the deGradHA protein degradation efficiency are shown on the right. Data are represented as mean ± SEM of 3 independent experiments. The *P* value ($P = 0.23$) was calculated using two-tailed unpaired Student's *t* test. (**B**) Representative immunoblot of the adult head extract of *aSMase*-HG line with (*Repo*-LexA/+ or y; *aSMase*-HG/+; *LexAop-deGradHA/+*) or without (*Repo*-LexA/+ or y; *aSMase*-HG/+) glial expression of deGradHA. (Right) Quantification of the deGradHA protein degradation efficiency are shown on the right. Data are represented as mean ± SEM of 6 independent experiments. The *P* value ($P = 0.006$) was calculated using two-tailed unpaired Student's *t*-test. (**C**) Representative immunoblot of the adult brain extract of *nSMase*-HG line with (*nSyb*-LexA/+; *nSMase*-HG/*LexAop-deGradHA*) or without (*nSyb*-LexA/+; *nSMase*-HG/+) neuronal expression of deGradHA. The average fold change (Avg. FC) of nSMase-3XHA immunoreactivity normalized to loading control (anti-Actin) is shown in the figure. (Right) Quantification of the deGradHA protein degradation efficiency are shown on the right. Data are represented as mean ± SEM of 5 independent experiments. The *P* value ($P < 0.001$) was calculated using two-tailed unpaired Student's *t* test. (**D**) Representative immunoblot of the adult brain extract of *nSMase*-HG line with (*Repo*-LexA/+ or y;; *nSMase*-HG/*LexAop-deGradHA*) or without (*Repo*-LexA/+ or y;; *nSMase*-HG/+) glial expression of deGradHA. Quantification of the deGradHA protein degradation efficiency are shown on the right. Data are represented as mean ± SEM of 4 independent experiments. The *P* value ($P = 0.97$) was calculated using two-tailed unpaired Student's *t* test. (**E**) Glial-specific degradation of aSMase-3XHA protein in the adult brain. The protein distribution of aSMase-3XHA is visualized by anti-HA immunostaining with co-stainings of glial nuclei (green; anti-Repo) of young adult brains (1-week-old). (Left) The representative image is taken from the posterior view of the Calyx, and the dashed-line square indicates the region of zoom-in images shown on the right. (Right) Zoom-in images show anti-HA signals (gray in the single-channel image; red in the overlay image) surrounding glial nuclei (green). (**F**) Neuron-specific degradation of nSMase-3XHA protein in the adult brain. The protein distribution of nSMase-3XHA is visualized by anti-HA immunostaining with co-stainings of neuronal nuclei (green; anti-Elav) of young adult brains (1-week-old). (Left) The representative image is taken from the posterior view of the Calyx, and the dashed-line square indicates the region of zoom-in images shown on the right. (Right) Zoom-in images show anti-HA signals (gray in the single-channel image; red in the overlay image) surrounding neuronal nuclei (green).

 © The Author(s)

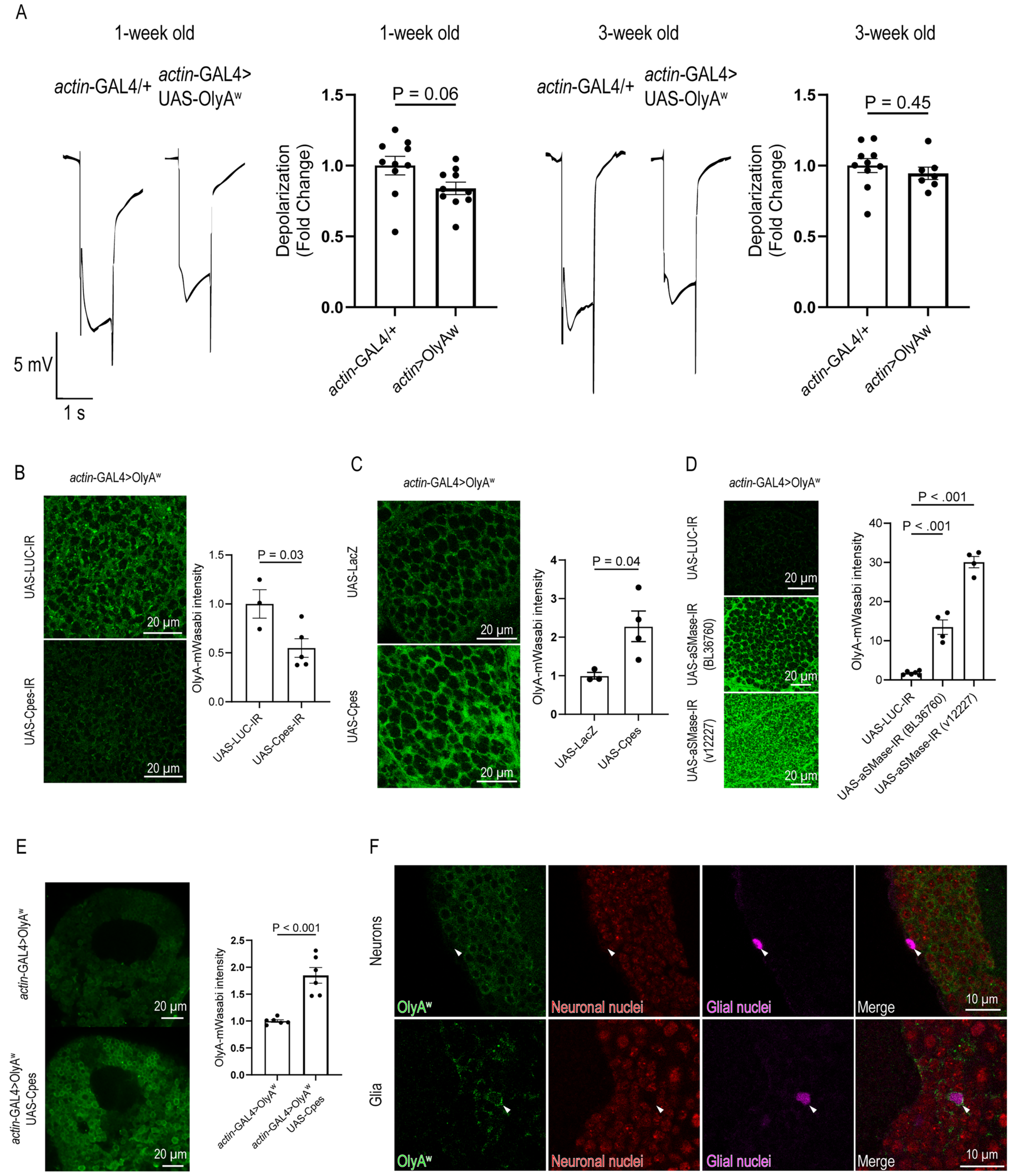
A
1-week old
actin-GAL4/+
actin-GAL4>
UAS-OlyAw
1-week old
P = 0.06
Depolarization
(Fold Change)
actin-GAL4/+
actin>OlyAw
3-week old
actin-GAL4/+
actin-GAL4>
UAS-OlyAw
3-week old
P = 0.45
5 mV
1 s
B
actin-GAL4>OlyAw
UAS-LUC-IR
UAS-Cpes-IR
20 µm
P = 0.03
OlyA-mWasabi intensity
C
actin-GAL4>OlyAw
UAS-LacZ
UAS-Cpes
P = 0.04
D
actin-GAL4>OlyAw
UAS-LUC-IR
UAS-aSMase-IR (BL36760)
UAS-aSMase-IR (v12227)
P < .001
E
actin-GAL4>OlyAw
actin-GAL4>OlyAw UAS-Cpes
P < 0.001
F
Neurons
Glia
OlyAw
Neuronal nuclei
Glial nuclei
Merge
10 µm

Figure EV5. Correlation between OlyA^{w} intensity and CerPE levels; Cell type-specific expression of OlyA^{w} in the brain; the effect of OlyA^{w} on neuronal function (related to Fig. 6).

(**A**) Representative electroretinogram (ERG) traces of control (*Actin*-GAL4/+) and OlyA^{w}-overexpressing (*Actin*-GAL4/ + ; *UAS-OlyA^{w}/+*) flies at 1 week and 3 weeks of age. Bar graphs show the quantification of the depolarization (normalized to the control). Data are representative of 3 independent experiments. Data are represented as mean ± SEM. The *P* value (1-week old, $P = 0.06$; 3-week old, $P = 0.45$) was calculated using two-tailed unpaired Student's *t* test. (**B**) OlyA^{w} expression in the L3 salivary glands upon control (*Actin*-GAL4 > UAS-OlyA^{w} + *UAS-LUC-IR*) and *Cpes* (*Actin*-GAL4 > *UAS-OlyA^{w}* + *UAS-Cpes-IR*) RNAi knockdowns. Data are representative of at least 2 independent experiments. Data are represented as mean ± SEM ($n \geq 3$). The *P* value ($P = 0.03$) were calculated using two-tailed unpaired Student's *t* test. (**C**) OlyA^{w} expression in the L3 salivary glands upon control (*Actin*-GAL4 > *UAS-OlyA^{w}* + *UAS-LacZ*) and *Cpes* (*Actin*-GAL4 > *UAS-OlyA^{w}* + *UAS-Cpes*) overexpression. Data are representative of at least 2 independent experiments. Data are represented as mean ± SEM ($n \geq 3$). The *P* value ($P = 0.04$) was calculated using two-tailed unpaired Student's *t* test. (**D**) OlyA^{w} expression in the L3 salivary glands upon control (*Actin*-GAL4 > *UAS-OlyA^{w}* + *UAS-LUC-IR*) and *aSMase* (*Actin*-GAL4 > *UAS-OlyA^{w}* + *UAS-aSMase-IR*) RNAi knockdowns. Data are representative of at least 2 independent experiments. Data are represented as mean ± SEM ($n \geq 4$). *P* values [aSMase-IR (BDSC 36760), $P < 0.001$; aSMase-IR (VDRC 12227), $P < 0.001$] were calculated using two-tailed unpaired Student's *t* test. (**E**) OlyA^{w} expression in the adult brain in control (*Actin*-GAL4 > *UAS-OlyA^{w}*) and *Cpes*-overexpressing (*Actin*-GAL4 > *UAS-OlyA^{w}* + *UAS-Cpes*) flies. Data are representative of at least 2 independent experiments. Data are represented as mean ± SEM ($n \geq 3$). The *P* value ($P < 0.001$) was calculated using two-tailed unpaired Student's *t* test. (**F**) (top) OlyA^{w} expression driven by a pan-neuron driver (*nSyb*-GAL4) in the adult brains. Neuronal nuclei are labeled by anti-Elav (red), and the glial nucleus is labeled by anti-Repo (magenta; arrowhead). (Bottom) OlyA^{w} expression driven by a pan-glia driver (*repo*-GAL4) in the adult brains. Neuronal nuclei are labeled by anti-Elav (red), and the glial nucleus is labeled by anti-Repo (magenta; arrowhead).

 © The Author(s)

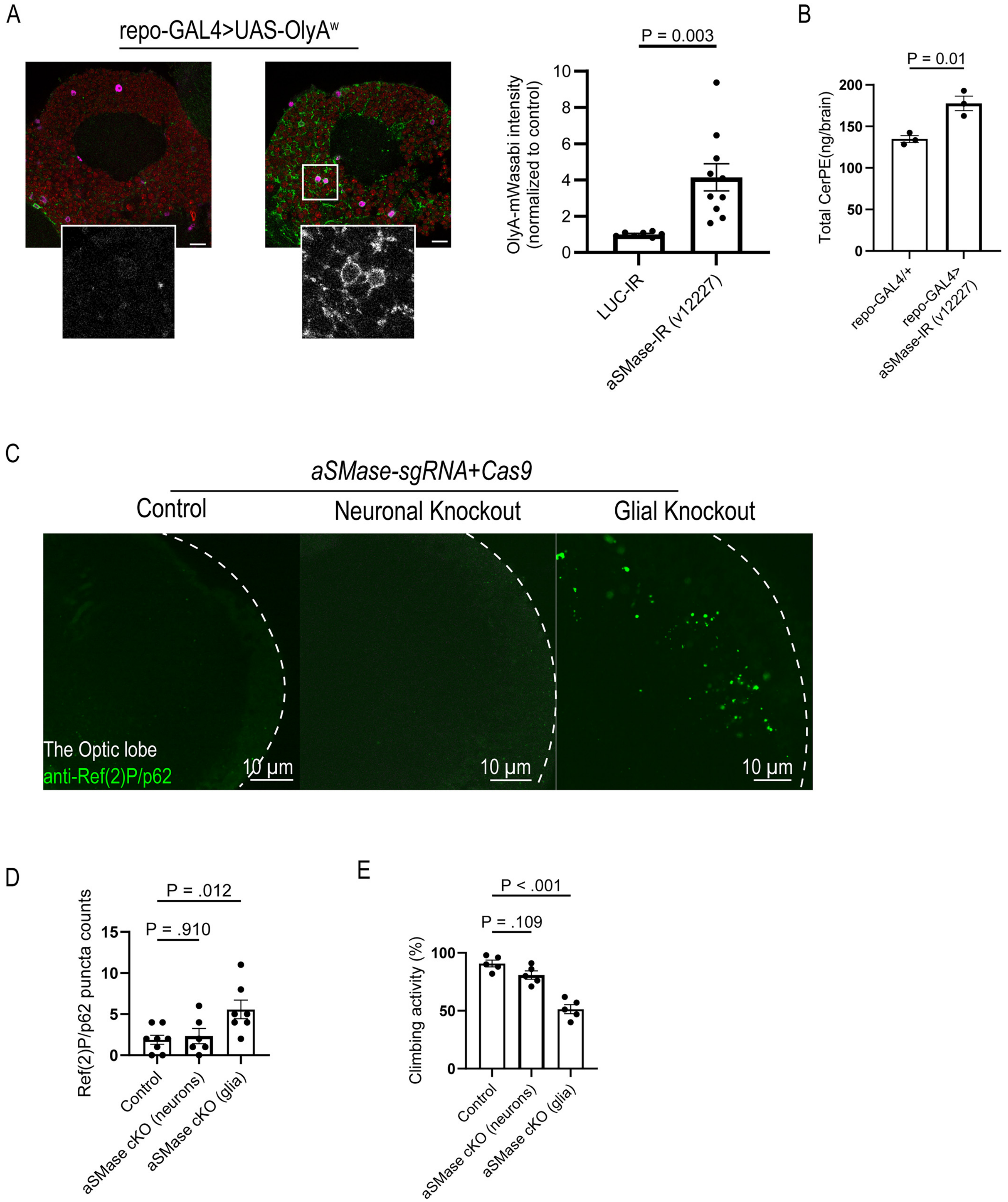
A
repo-GAL4>UAS-OlyA^w
P = 0.003
OlyA-mWasabi intensity (normalized to control)
LUC-IR
aSMase-IR (v12227)
B
P = 0.01
Total CerPE(ng/brain)
repo-GAL4/+
repo-GAL4> aSMase-IR (v12227)
C
aSMase-sgRNA+Cas9
Control
Neuronal Knockout
Glial Knockout
The Optic lobe
anti-Ref(2)P/p62
10 μm
D
P = .012
P = .910
Ref(2)P/p62 puncta counts
Control
aSMase cKO (neurons)
aSMase cKO (glia)
E
P < .001
P = .109
Climbing activity (%)

Figure EV6. aSMase loss-of-function phenotypes (related to Fig. 7).

(**A**) OlyA^w expression and the *aSMases RNAi* knockdown in glia. UAS-OlyA^w expression and aSMases RNAi were driven by *repo*-GAL4 (*repo*-GAL4 > *UAS-OlyA^w+aSMase-RNAi*). Confocal images were captured from the posterior view of the Calyx in the adult brain of young adult flies (1 week old). (Right) Quantifications of OlyA^w intensity in the cortical region. The *P* value ($P = 0.003$) was calculated using two-tailed unpaired Student's *t* test. Data are represented as mean ± SEM ($n \geq 7$). Data are representative of 4 independent experiments. (**B**) Quantification of the total CerPE levels in control (*repo*-GAL4/+) and *aSMase* knockdown (*repo*-GAL4/UAS-aSMase-RNAi) flies (day 10 female). The *P* value ($P = 0.01$) were calculated using two-tailed unpaired Student's *t* test. Data are represented as mean ± SEM ($n > 3$). Data are representative of 3 independent experiments. (**C**) Ref(2)P/p62 immunostainings in the optic lobe of adult brains from control or *aSMase* conditional knockout flies (1-week-old). Cell type-specific *aSMase* knockout was achieved by using small guide RNA targeting *aSMase* and Cas9 driven by neuronal (*nSyb*-GAL4) or glial (*repo*-GAL4) drivers. Control: *UAS-Cas9/+*; *UAS-aSMase-sgRNA/+*; neuronal cKO: *nSyb>Cas9+aSMase-sgRNA;* glial cKO: *repo>Cas9+aSMase-sgRNA*. (**D**) Quantification of Ref(2)P/p62 puncta counts. Data are represented as mean ± SEM ($n \geq 6$). *P* values [Control vs. aSMase cKO (neuron), $P = 0.910$; Control vs. aSMase cKO (glia), $P = 0.012$] were calculated using one-way ANOVA with Tukey's multiple comparisons. Data are representative of >5 independent experiments. (**E**) Climbing assay of control or *aSMase* conditional knockout flies (1-week-old). Cell type-specific *aSMase* knockout was achieved by using small guide RNA targeting *aSMase* and Cas9 driven by neuronal (*nSyb*-GAL4) or glial (*repo*-GAL4) drivers. Data are represented as mean ± SEM ($n = 5$). *P* values [Control vs. aSMase cKO (neuron), $P = 0.109$; Control vs. aSMase cKO (glia), $P < 0.001$] were calculated using one-way ANOVA with Tukey's multiple comparisons. Data are representative of 5 independent experiments. Control: *UAS-Cas9/+*; *UAS-aSMase-sgRNA/+*; neuronal cKO: *nSyb>Cas9+aSMase-sgRNA;* glial cKO: *repo>Cas9+aSMase-sgRNA*.

 © The Author(s)
